# Fidelity Mechanics: Analogues of the Four Thermodynamic Laws and Landauer’s Principle

**DOI:** 10.3390/e24091306

**Published:** 2022-09-15

**Authors:** Huan-Qiang Zhou, Qian-Qian Shi, Yan-Wei Dai

**Affiliations:** Centre for Modern Physics, Chongqing University, Chongqing 400044, China

**Keywords:** quantum critical phenomena, tensor network algorithms, symmetry-breaking order, topological order, an analogue of Landauer’s principle, analogues of the four thermodynamic laws, fidelity flows, renormalization group flows, arrows of time

## Abstract

Fidelity mechanics is formalized as a framework for investigating critical phenomena in quantum many-body systems. Fidelity temperature is introduced for quantifying quantum fluctuations, which, together with fidelity entropy and fidelity internal energy, constitute three basic state functions in fidelity mechanics, thus enabling us to formulate analogues of the four thermodynamic laws and Landauer’s principle at zero temperature. Fidelity flows, which are irreversible, are defined and may be interpreted as an alternative form of renormalization group flows. Thus, fidelity mechanics offers a means to characterize both stable and unstable fixed points: divergent fidelity temperature for unstable fixed points and zero-fidelity temperature and (locally) maximal fidelity entropy for stable fixed points. In addition, fidelity entropy behaves differently at an unstable fixed point for topological phase transitions and at a stable fixed point for topological quantum states of matter. A detailed analysis of fidelity mechanical-state functions is presented for six fundamental models—the quantum spin-1/2 XY model, the transverse-field quantum Ising model in a longitudinal field, the quantum spin-1/2 XYZ model, the quantum spin-1/2 XXZ model in a magnetic field, the quantum spin-1 XYZ model, and the spin-1/2 Kitaev model on a honeycomb lattice for illustrative purposes. We also present an argument to justify why the thermodynamic, psychological/computational, and cosmological arrows of time should align with each other, with the psychological/computational arrow of time being singled out as a master arrow of time.

## 1. Introduction

Quantum critical phenomena [1,2,3] arise from cooperative behavior in quantum many-body systems. Conventionally, there are two categories of theories for describing these fascinating physical phenomena. One is adapted from Landau’s spontaneous symmetry-breaking (SSB) theory [4], with a symmetry-broken phase characterized by nonzero values of a local order parameter. SSB occurs in a system when its Hamiltonian enjoys a certain symmetry, whereas the ground-state wave functions do not preserve it [5,6]. The implication of an SSB phenomenon is twofold: first, a system has stable and degenerate ground states, each of which breaks the symmetry of the system; second, the symmetry breakdown results from random perturbations. The other is Wilson’s renormalization group (RG) theory [7,8,9,10,11,12], originated from the field-theoretic approach to classical many-body systems. However, this so-called Landau–Ginzburg–Wilson paradigm suffers from a few fundamental problems: first, ubiquitous topologically ordered states occur beyond the SSB order [13,14,15]; second, even for SSB-ordered states, using only local-order parameters is insufficient to quantify quantum fluctuations; third, the proliferation of an unlimited number of irrelevant coupling constants occurs in various RG schemes, which makes it impractical to work out RG flows from unstable fixed points to stable fixed points; fourth, intrinsic irreversibility, i.e., information loss, along RG flows is baffling due to the fact that a number of high-energy degrees of freedom are discarded during the construction of the effective Hamiltonian. As such, a full characterization of quantum critical phenomena is still lacking.

The latest advances in our understanding of quantum critical phenomena originate from a perspective of fidelity [16,17,18,19,20,21,22,23,24,25,26,27,28], a basic notion in quantum information science. In Refs. [26,27,28], it has been argued that the ground-state fidelity per lattice site is fundamental in the sense that it may be used to characterize quantum phase transitions (QPTs) regardless of what type of internal order is present in quantum many-body states. In other words, the ground-state fidelity per lattice site is able to describe QPTs arising from symmetry-breaking order and/or topological order. This has been further confirmed in Refs. [29,30], where topologically ordered states in the spin-1/2 Kitaev model on a honeycomb lattice [31,32,33] and the Kosterlitz–Thouless (KT) transitions [34,35] are investigated. The argument is solely based on the basic postulate of quantum mechanics on quantum measurements, which implies that two nonorthogonal quantum states are not reliably distinguishable [36]. Moreover, even for quantum many-body systems with a symmetry-breaking order, it is advantageous to adopt the ground-state fidelity per lattice site instead of using conventional local order parameters due to the fact that it is model-independent, although one may systematically derive local order parameters from tensor network representations [37,38,39,40,41,42,43,44,45,46,47,48] of ground-state wave functions by investigating reduced density matrices for local areas on an infinite-size lattice [49,50]. In fact, a systematic scheme to study quantum critical phenomena in the context of the fidelity approach consists of three steps, as advocated in [49,50]: first, map out the ground-state phase diagram by evaluating the ground-state fidelity per lattice site; second, derive local order parameters (if any) from the reduced density matrices for a representative ground-state wave function in a given phase; third, characterize any phase without any long-range order in terms of non-local order parameters. We remark that this is even valid for thermal phase transitions, if we extend the notion of fidelity from pure states to mixed states to accommodate thermal fluctuations [26]. In fact, the logarithmic function of finite temperature fidelity per lattice site for two thermal mixed states corresponding to two different temperatures reduces to nothing but the free energy if other non-thermal control parameters are kept fixed. This implies that the singularities in finite temperature fidelity per lattice site coincide with those in the free energy, thus showing the equivalence between the fidelity approach and the conventional one to thermal phase transitions.

An intriguing question is to ask whether or not the fidelity approach provides a full characterization of quantum critical phenomena in the sense that it is not only able to signal critical points/unstable fixed points but also offers a method for locating stable fixed points. Moreover, it has to clarify in what sense a quantum many-body system flows from unstable fixed points to stable fixed points in the control parameter space, which may be understood as a flow discarding irrelevant information along the way. Given this as our main goal, our study is motivated by a few specific questions:

(i) There is long-standing folklore pointing towards a similarity between critical points and black holes, which usually refers to the fact that the effects from a critical point at zero temperature may be observed in a critical regime at low but finite temperature [51]. Given that both critical points and black holes originate from singularities, there should be a method for clarifying a formal similarity between QPTs and black holes.
(ii) RG flows from an unstable fixed point to a stable fixed point are irreversible. This is relevant to Zamolodchikov’s *c*-theorem [52,53,54] and Cardy’s *a*-theorem [55,56], which may be regarded as the adaptation of the renowned Boltzmann’s H theorem to the RG setting. In real space RG theories, such as Kadanoff block spins as well as other coarse-graining RG schemes, high-energy degrees of freedom are discarded. Therefore, RG flows seem irreversible in a similar sense to the situations described by Boltzmann’s H theorem, where physical time is replaced by an RG parameter [57]. Thus, it is desirable to see if there are any intrinsic explanations for the irreversibility from the perspective of fidelity.
(iii) As Landauer first noted [58], at finite temperature *T*, in order to erase one bit of information, we need to perform minimum work *w*: $w=k_{B}T\ln2$, with $k_{B}$ being the Boltzmann constant. At zero temperature, do we still need to perform any minimum work to erase one bit of information?
(iv) During the construction of an effective Hamiltonian along any RG flow, an unlimited number of irrelevant coupling constants proliferate. In practice, this prevents access to a stable fixed point. It is tempting to see if there is any deep reason underlying this observation.
(v) A proper definition of QPTs is still lacking. Traditionally, the ground-state energy density is used as an indicator to signal a critical point, but it fails in many situations [59]. Instead, some exotic indicators, such as entanglement entropy [60], topological entanglement entropy [61,62], and the ground-state fidelity per lattice site [26,27,28], are introduced to signal QPTs due to recent progress in our understanding of quantum critical phenomena. Therefore, it is important to find a proper criterion to define QPTs.

In this study, we aim to answer these questions. This is achieved by introducing fidelity temperature to quantify quantum fluctuations present in a given ground-state wave function for a quantum many-body system, which exhibits QPTs. As it turns out, fidelity temperature, together with fidelity entropy and fidelity internal energy, offer us a proper basis to describe QPTs that are both continuous and discontinuous. As a consequence, this allows us to formulate analogues of the four thermodynamic laws and Landauer’s principle. As illustrations, we discuss six fundamental models. These are the quantum spin-1/2 XY model, the transverse-field quantum Ising model in a longitudinal field, the quantum spin-1/2 XYZ model, the quantum spin-1/2 XXZ model in a magnetic field, the quantum spin-1 XYZ model, and the spin-1/2 Kitaev model on a honeycomb lattice. Rich physics is unveiled even for these well-studied models. First, for the quantum spin-1/2 XY model, the disordered circle is interpreted as a separation line between two different types of fidelity flows, with one type of fidelity flows starting from unstable fixed points with central charge c=1, and the other type of fidelity flows starts from unstable fixed points with central charge c=1/2. Both types of fidelity flows end at the same stable fixed point (0,1), at which fidelity entropy reaches its local maximum. Another remarkable feature is that fidelity temperatures are zero on the disordered circle, as it should be, since no quantum fluctuations exist in a factorized state. However, fidelity temperature is not well-defined at the Pokrovsky–Talapov (PT) transition point [63,64], ranging from 0 to ∞, depending on how it is approached. Second, for the quantum Ising model with transverse field λ and longitudinal field *h*, there are stable fixed points in the (λ,h) plane at (0,0), (0,∞), (∞,0), and (1,∞). The existence of stable fixed points (0,0) and (∞,0) is protected by the $Z_{2}$ symmetry when h=0, whereas the existence of stable fixed points (0,∞) and (1,∞) may be interpreted as a consequence of the variation of the symmetry group with λ: U(1) for λ=0 and none for λ≠0, when h≠0, although they usually are identified as the same point. Third, for the quantum spin-1/2 XYZ model, five different dualities have been identified, which enable us to reproduce the ground-state phase diagram for the quantum XYZ model. At the ferromagnetic (FM) transition point, fidelity temperature is not well-defined, ranging from 0 to infinity depending on how the FM transition point is approached. This is very much similar to the PT transition point for the quantum spin-1/2 XY model. Furthermore, KT transitions are characterized as topological, since fidelity entropy is not single-valued at the transition point. Fourth, for the XXZ model in a magnetic field, at the phase boundary between the critical XY phase and the antiferromagnetic (AF) phase, fidelity temperatures are not well-defined, ranging from a finite value to *∞*. That is, a QPT at this phase boundary is an intermediate case (IC) interpolating between a KT transition and a PT transition, which represents a new universality class that is different from both the KT transitions and PT transitions. Fifth, fidelity mechanical-state functions for the quantum spin-1 XYZ model, which exhibits the symmetry-protected topological (SPT) phase—the Haldane phase [65,66]—are evaluated. It was observed that fidelity entropy takes double values on the characteristic line (γ=0) in the Haldane phase, reflecting its topological nature in the control parameter space. Sixth, the spin-1/2 Kitaev model on a honeycomb lattice is discussed, with a detailed analysis of fidelity mechanical-state functions being carried out. The topological nature of the gapped and gapless spin liquid phases is reflected in the fact that fidelity entropy takes multiple values at the characteristic points.

The layout of this study is as follows. Section 1 is an introduction, describing our motivations to formalize a full characterization of quantum critical phenomena in the context of fidelity mechanics. In particular, five questions are raised regarding the current status of theoretical investigations into quantum critical phenomena. In Section 2, we first define a fidelity mechanical system and its environment, thus attaching a physical meaning to the present, the past, and the future, with information storage as a key ingredient, and then we introduce three fidelity mechanical-state functions, i.e., fidelity entropy, fidelity temperature, and fidelity internal energy, with an analogue of Landauer’s principle at zero temperature as a basic requirement from the internal logical consistency. Here, we emphasize that a key relation between an unknown function, as a defining factor for fidelity internal energy, and fidelity temperature is established in Section 2.5. In Section 3, a canonical form of the Hamiltonian in fidelity mechanics is discussed, thus unveiling an inherently fundamental role of duality in fidelity mechanics. In particular, the meaning of a canonical form of the Hamiltonian is clarified by relating duality with a shift operation in the Hamiltonian. Therefore, any artificial choice of the definition of duality is irrelevant as long as the identification of unstable and stable fixed points is concerned for fidelity flows, introduced later on in Section 13. Moreover, the consequences ensuing from a shift operation in the Hamiltonian is elaborated. In Section 4, a fictitious parameter σ is introduced to address different choices of a dominant control parameter in a given regime for quantum many-body systems. As demonstrated, information encoded in this fictitious parameter σ arising from different choices of a dominant control parameter is irrelevant in fidelity mechanics. In Section 5, fidelity mechanical-state functions are discussed under a shift operation in the Hamiltonian with respect to a reference benchmark. In Section 6, distinct features of different types of QPTs and quantum states of matter in fidelity mechanics are described, thus offering a means to characterize topological QPTs and topological quantum states of matter in the control parameter space. In Section 7, we present fidelity mechanical-state functions for the quantum spin-1/2 XY model, which is a typical example for continuous QPTs. In Section 8, fidelity mechanics is discussed for the transverse-field quantum Ising model in a longitudinal field, which exhibits a discontinuous QPT. In Section 9, fidelity mechanics are discussed for the quantum spin-1/2 XYZ model, thus offering a prototype for the role of dualities in fidelity mechanics. In Section 10, an analysis of fidelity mechanical-state functions is presented for the quantum spin-1/2 XXZ model in a magnetic field, which enables us to unveil an IC transition point. In Section 11, fidelity mechanics are discussed for the quantum spin-1 XYZ model, which exhibits the SPT phase—the Haldane phase [65,66]. In Section 12, a detailed analysis of fidelity mechanical-state functions is carried out for the spin-1/2 Kitaev model on a honeycomb lattice, which exhibits a topological phase transition (TPT). Here, we stress that, apart from the quantum spin-1/2 XY model and the spin-1/2 Kitaev model on a honeycomb lattice, a tensor network algorithm [39,40,41,46,47,48,67,68,69] in a matrix-product state representation has been exploited to simulate quantum many-body systems in these illustrative examples, thus making it possible to numerically evaluate the ground-state fidelity per lattice site and, in turn, fidelity mechanical-state functions. In Section 13, we answer the questions raised in the Introduction and define fidelity flows as an alternative form of RG flows. Moreover, an argument is presented to justify why the thermodynamic, psychological/computational and cosmological arrows of time should align with each other in the context of fidelity mechanics, with the psychological/computational arrow of time being singled out as a master arrow of time. Section 14 explains what insight fidelity mechanics might provide into our search for a classification of quantum states of matter and QPTs. The last Section 15 is devoted to concluding remarks.

Some supplementary materials are also presented in the Appendices, which are intended for readers interested in technical details. In Appendix A, we introduce the ground-state fidelity per lattice site and define a pinch point as an intersection point between two singular lines on a fidelity surface. As typical examples, the transverse-field quantum Ising model and the spin-1/2 Kitaev model on a honeycomb lattice are presented to illustrate QPTs arising from symmetry-breaking and topological orders, respectively, based on the exact expressions for the ground-state fidelity per lattice site, which in turn are derived from the exact solutions for the quantum spin-1/2 XY model and the spin-1/2 Kitaev model on a honeycomb lattice. In Appendix B, we summarize the infinite time-evolving block decimation algorithm (iTEBD) [46,47,48], which is efficient for generating ground-state wave functions in the matrix-product state representation for quantum many-body systems in one spatial dimension. Thus, it offers an efficient scheme to evaluate the ground-state fidelity per lattice site and to identify (unentangled) separable states [70,71] numerically. In Appendix C, dualities are presented for the quantum spin-*s* XYZ model and the spin-1/2 Kitaev model on a honeycomb lattice, respectively. In Appendix D, arrows of time are discussed, with a focus on the thermodynamic, psychological/computational, and cosmological arrows of time. It is argued that fidelity mechanics may be regarded as an attempt to understand the psychological/computational arrow of time in the context of quantum many-body systems. Appendix E recalls three theorems in quantum information science, which are used to justify our definition of a fidelity mechanical system and its environment. In Appendix F, three extensions of fidelity mechanics are made to adapt it to other situations: (1) when the ground-state energy density e(x) is always positive; (2) quantum many-body systems are not translation-invariant; (3) thermal phase transitions occur at finite temperature. In Appendix G, scaling entropy is discussed for the quantum spin-1/2 XYZ model, the quantum spin-1 XYZ model, and the spin-1/2 Kitaev model on a honeycomb lattice. In Appendix H, a scaling behavior of fidelity entropy in the vicinity of a critical point is performed, and a scaling analysis is presented for the quantum spin-1/2 XY model near a line of the Gaussian critical points. In Appendix I, a universal logarithmic scaling behavior of the block entanglement entropy is summarized for scale-invariant states arising from SSB with type-B Goldstone modes (GMs), which is relevant to a characterization of QPTs in fidelity mechanics. In Appendix J, the bond-centered and site-centered non-local order parameters are defined, which are used to characterize the SPT phases and the symmetry-protected trivial (SPt) phases, respectively. An efficient method for evaluating the bond-centered and site-centered non-local order parameters in the matrix-product state representation is described. In Appendix K, mathematical details are discussed about fidelity entropy, fidelity temperature, and fidelity internal energy for the quantum spin-1/2 XY model. In Appendix L, explicit expressions for fidelity entropy, fidelity temperature, and fidelity internal energy are presented for the transverse-field quantum Ising model in a longitudinal field. In Appendix M, mathematical details for fidelity entropy, fidelity temperature, and fidelity internal energy are discussed for the quantum spin-1/2 XYZ model. In Appendix N, mathematical details for fidelity entropy, fidelity temperature, and fidelity internal energy are discussed for the quantum spin-1/2 XXZ model in a magnetic field. In Appendix O, mathematical details for fidelity entropy, fidelity temperature, and fidelity internal energy are discussed for the quantum spin-1 XYZ model and for the quantum spin-1 bilinear–biquadratic model. In Appendix P, mathematical details for fidelity entropy, fidelity temperature, and fidelity internal energy are discussed for the spin-1/2 Kitaev model on a honeycomb lattice. In Appendix Q, a distinction between fidelity flows mimicking Zamolodchikov RG flows and fidelity flows mimicking real space RG flows is made, with the quantum spin-1/2 XY model as an illustrative example.

Although fidelity mechanics is formalized as a unifying framework for quantum critical phenomena, its ramifications are well beyond this specific research area. Indeed, its relevance to physical information is deeply rooted in the fact that fidelity itself is a basic notion in quantum information science. Moreover, information storage plays a fundamental role in both fidelity mechanics and computer science in addition to the supposition that fidelity work might be exploited to quantify computational costs in solving a mathematical problem from a temporal perspective, thus establishing a link to computational complexity [72].

The layout is arranged in such a way that a reader, who is only interested in the main ideas, may choose to focus on Section 2, Section 3, Section 4 and Section 5 and Section 7, Section 8, Section 9, Section 10, Section 11, Section 12, Section 13, Section 14 and Section 15, which contain the basic ingredients in our formalism. In addition, one may choose to peruse any of Section 7, Section 8, Section 9, Section 10, Section 11 and Section 12 to see how the formalism is implemented for a specific model, and these sections offer typical examples for distinct types of quantum states of matter and QPTs, including continuous QPTs, discontinuous QPTs, the KT and PT transitions, the SPT phases, and topologically ordered states, and other sections can be skipped without any effect on further reading. The Appendices are intended for a reader who is interested in various technical details involved, which also act as a pointer to further references, although Appendix A, Appendix B, Appendix C, Appendix D, Appendix E, Appendix F, Appendix G and Appendix H contain some background materials.

## 2. Fidelity Mechanics: Basic State Functions

### 2.1. Preliminaries

Consider a quantum many-body system on a lattice described by a Hamiltonian $H(x_{1},x_{2})$, with $x_{1}$ and $x_{2}$ being two control (coupling) parameters. However, it is straightforward to extend our discussion to a Hamiltonian with an arbitrary number of coupling parameters. For our purpose, it is necessary to determine its ground-state phase diagram, in addition to symmetries, dualities, and factorizing fields. As is well known, lines of critical/transition points divide the control parameter space into different phases, which may be characterized in terms of local-order parameters for symmetry-breaking ordered phases and non-local order parameters for topologically ordered phases. In contrast, symmetries, dualities, and factorizing fields furnish characteristic lines in the control parameter space, which separate a given phase into different regimes. Note that a multi-critical point arises if two lines of QPT points, both continuous and discontinuous, meet each other. As a consequence, a peculiar type of characteristic lines may appear, originating from a multi-critical point, if no characteristic line arising from symmetries, dualities, and factorizing fields exists. We introduce six fundamental models to be investigated for illustrative purposes and explain the relevant terminologies to set a stage for our formalism.

#### 2.1.1. Quantum Many-Body Systems: Six Illustrative Models

The first model is the quantum spin-1/2 XY model, described by the following Hamiltonian:

(1) $$H\left(\lambda ,\gamma \right)=-\sum_{i}\left(\frac{1+\gamma }{2}\sigma_{i}^{x}\sigma_{i+1}^{x}+\frac{1-\gamma }{2}\sigma_{i}^{y}\sigma_{i+1}^{y}+\lambda \sigma_{i}^{z}\right),$$

where $\sigma_{i}^{x}$, $\sigma_{i}^{y}$, and $\sigma_{i}^{z}$ are the spin-1/2 matrices at site *i*, γ is the anisotropic parameter, and λ is the transverse field. The second model is the transverse-field quantum Ising model in a longitudinal field, described by the following Hamiltonian:

(2) $$H\left(\lambda ,h\right)=-\sum_{i}\left(\sigma_{i}^{x}\sigma_{i+1}^{x}+\lambda \sigma_{i}^{z}+h\sigma_{i}^{x}\right),$$

where λ is the transverse field, and *h* is the longitudinal field. The third model is the quantum spin-1/2 XYZ model described by the following Hamiltonian:

(3) $$H\left(\gamma ,\Delta \right)=\sum_{i}\left(\frac{1+\gamma }{2}\sigma_{i}^{x}\sigma_{i+1}^{x}+\frac{1-\gamma }{2}\sigma_{i}^{y}\sigma_{i+1}^{y}+\frac{\Delta }{2}\sigma_{i}^{z}\sigma_{i+1}^{z}\right),$$

where γ and Δ are the anisotropic coupling constants. The fourth model is the quantum spin-1/2 XXZ model in a magnetic field, described by the following Hamiltonian:

(4) $$H\left(\Delta ,h\right)=\sum_{i}\left(\sigma_{i}^{x}\sigma_{i+1}^{x}+\sigma_{i}^{y}\sigma_{i+1}^{y}+\Delta \sigma_{i}^{z}\sigma_{i+1}^{z}+2h\sigma_{i}^{z}\right),$$

where Δ is the anisotropic coupling constant, and *h* is the magnetic field strength. The fifth model is the quantum spin-1 XYZ model, for which its Hamiltonian takes the following form:

(5) $$H\left(\gamma ,\Delta \right)=\sum_{i}\left(\frac{1+\gamma }{2}S_{i}^{x}S_{i+1}^{x}+\frac{1-\gamma }{2}S_{i}^{y}S_{i+1}^{y}+\frac{\Delta }{2}S_{i}^{z}S_{i+1}^{z}\right),$$

where $S_{i}^{x}$, $S_{i}^{y}$, and $S_{i}^{z}$ are the spin-1 matrices at site *i*, and γ and Δ are anisotropic coupling constants. The sixth model is the spin-1/2 Kitaev model [31] on a honeycomb lattice, with the following Hamiltonian:

(6) $$H\left(J_{x},J_{y},J_{z}\right)=-J_{x}\sum_{x-\mathrm{bonds}}\sigma_{i}^{x}\sigma_{j}^{x}-J_{y}\sum_{y-\mathrm{bonds}}\sigma_{i}^{y}\sigma_{j}^{y}-J_{z}\sum_{z-\mathrm{bonds}}\sigma_{i}^{z}\sigma_{j}^{z}.$$

Here, *i* and *j* label a pair of the nearest-neighbor sites, which hosts an Ising-like coupling on the three distinct types of bonds, labelled as the *x*-bonds, the *y*-bonds, and the *z*-bonds, on a honeycomb lattice, and $J_{x}$, $J_{y}$, and $J_{z}$ denote coupling constants on the three distinct types of the bonds, respectively.

#### 2.1.2. Ground-State Fidelity per Lattice Site

The ground-state phase diagram may be mapped out by evaluating the ground-state fidelity per lattice site. As demonstrated in [26,27,28,29,30,49,50,73,74,75], the ground-state fidelity per lattice site is able to signal QPTs arising from symmetry-breaking order and/or topological order. Here, we restrict ourselves to briefly recall the definition of the ground-state fidelity per lattice site (also cf. Appendix A for more details).

For two ground states $|\psi_{N}(x_{1},x_{2})\rangle$ and $|\psi_{N}(y_{1},y_{2})\rangle$, the ground-state fidelity is a measure of the similarity between them, with *N* being the system size. Mathematically, it is defined as the absolute value of their overlap

(7) $$F_{N}\left(x_{1},x_{2};y_{1},y_{2}\right)=\left|\langle \psi_{N}\left(y_{1},y_{2}\right)|\psi_{N}\left(x_{1},x_{2}\right)\rangle \right|.$$

Here, we stress that $y_{1}$ and $y_{2}$ should be understood as different values of the *same* control parameters as $x_{1}$ and $x_{2}$, respectively. In the thermodynamic limit, any two ground states are always distinguishable (orthogonal). That is, the fidelity between these two states vanishes. However, for a large but finite lattice size *N*, $F_{N}\left(x_{1},x_{2};y_{1},y_{2}\right)$ scales as $d^{N}\left(x_{1},x_{2};y_{1},y_{2}\right)$, with $d(x_{1},x_{2};y_{1},y_{2})$ being a scaling parameter. In the thermodynamic limit, one may introduce the ground-state fidelity per lattice site as follows

(8) $$d\left(x_{1},x_{2};y_{1},y_{2}\right)=\lim_{N\to \infty }F_{N}^{1/N}\left(x_{1},x_{2};y_{1},y_{2}\right).$$

Note that $d(x_{1},x_{2};y_{1},y_{2})$ is well-defined in the thermodynamic limit. An efficient scheme to evaluate the ground-state fidelity per lattice site is described in Appendix B for ground-state wave functions in the matrix-product state representation, which in turn are generated from a tensor network algorithm—the iTEBD algorithm [46,47,48].

#### 2.1.3. A Characteristic Line and a Characteristic Point

We introduce a concept—a characteristic line—which turns out to be a key ingredient in fidelity mechanics. We itemize four types of characteristic lines, each with an illustrative example.

(i) Generically, the symmetry group of the Hamiltonian varies with coupling parameters $x_{1}$ and $x_{2}$. If the Hamiltonian possesses a distinct symmetry group when the coupling constants take special values on a characteristic line in a given phase, then it separates this phase into different regimes in the control parameter space. An example to illustrate this observation is the quantum spin-1/2 XYZ model (3). For this model, on the line (γ=1−Δ), the Hamiltonian possesses U(1) symmetry, which is lost when coupling parameters move away from this characteristic line. In particular, a U(1) symmetry occurs when one coupling parameter is infinite in value. This type of characteristic line is referred to as a symmetric line.
(ii) Characteristic lines also arise from dualities [3,76], which are defined via a local or nonlocal unitary transformation, and they separate a given phase into different regimes in the control parameter space. An example to illustrate this observation is the quantum spin-1/2 XY model (1) [3,76]. Dualities exist along the two lines (λ=0 and γ=1). This type of characteristic line is referred to as a dual line. Caveat: Sometimes it is a bit tricky to recognize a dual line as a type of characteristic line. Suppose dualities exist on a plane. Then, the plane itself is a characteristic plane. Generically, a line in this plane is not a characteristic line, unless this line is self-dual in nature. However, if a line turns out to be a dual line for a sub-model, with one of the two control parameters being zero, then it is also recognized as a characteristic line for the full model. Mathematically, this amounts to stating that such a characteristic line is semi-self-dual in the sense that only one of the two coupling parameters remains to be the same. This is seen in the quantum spin-1/2 XYZ model (3) and the quantum spin-1 XYZ model (5).
(iii) Another type of characteristic line comes from factorizing fields [77,78,79,80,81,82]. Indeed, apart from various analytical approaches, there is an efficient numerical means for identifying factorizing fields for quantum many-body systems in the context of tensor networks [70,71] (also cf. Appendix B). A line of factorizing fields divides a specific phase into different regimes. Examples to illustrate this observation are the quantum spin-1/2 XY model (1) and the quantum spin-1/2 XYZ model (3) [77,78,79,80,81,82]. An interesting feature for a line of factorizing fields is that they frequently occur in a symmetry-broken phase and, in turn, are frequently associated with the PT transitions and FM transitions. We remark that factorizing fields also occur when one coupling parameter takes infinity in value or when more than one coupling parameters are infinite in value. This type of characteristic line is referred to as a factorizing-field line.
(iv) There is a peculiar type of characteristic line, originating from an isolated critical or a multi-critical point and ending at a point on a symmetric line, a dual line, or a factorizing-field line. This type of characteristic line is needed, if any other type of characteristic line is absent at such an isolated critical point or a multi-critical point. An illustrative example is a characteristic line (λ=1) for the transverse-field quantum Ising model in a longitudinal field (2). This type of characteristic line is referred to as a soft line due to the fact that this type of characteristic line does not impose any *rigid* constraints in a sense that its location in the control parameter space is not fixed in contrast to the constraints imposed by a symmetric line, a dual line, and a factorizing-field line.

In addition, it is useful to introduce a characteristic point, which is defined as an intersection point between two or more characteristic lines, including a symmetric line, a dual line, and a factorizing-field line, in the control parameter space.

#### 2.1.4. A Principal Regime

Given that characteristic lines separate a given quantum phase into different regimes, we need to clarify physical reasons underlying this separation. In our scenario, all ground states in a given phase share the same relevant information, with their distinguishability fully attributed to the fact that irrelevant information encoded in different ground states is different (cf. Appendix A for more details). However, this does not categorize any different types of irrelevant information that are possible in a given phase, which in turn may be traced back to critical points belonging to different universality classes. Actually, it is the four different types of characteristic lines that separate a given phase into different regimes, making it possible to attach a certain type of irrelevant information to each regime. That is, there is a one-to-one correspondence between a regime and the type of irrelevant information in a given phase. In addition, this also applies to characteristic lines themselves: different types of irrelevant information are attached to different characteristic lines, if they are present simultaneously in a given phase.

As a result of dualities, not all regimes are independent; we refer to all independent regimes as principal regimes, which actually represent the underlying physics for a given quantum many-body system. Accordingly, all other regimes, dual to a principal regime, are referred to as non-principal regimes. As a rule, a principal regime always includes an isolated critical point, a line of discontinuous QPT points ending at an isolated critical point or a line of critical points as a boundary. In addition, non-principal regimes are symmetric or dual image regimes that are symmetrical or dual in nature relative to a principal regime.

#### 2.1.5. A Dominant Control Parameter *x* and an Auxiliary Control Parameter τ

Now we are ready to introduce a *dominant* control parameter *x* and an *auxiliary* control parameter τ to replace the original coupling parameters $x_{1}$ and $x_{2}$ such that there is a one-to-one correspondence between (*x*, τ) and ($x_{1}$, $x_{2}$) in a specific regime. Therefore, the Hamiltonian $H(x_{1},x_{2})$ is re-parametrized as H(x,τ). As a dominant control parameter, *x* has to satisfy three conditions. First, as a function of *x*, the ground-state energy density e(x,τ) is monotonic with increasing *x* for a fixed τ. Second, the range of *x* is *finite*. Third, the ground-state fidelity per lattice site d(x,τ;y,τ) is nonzero.

An auxiliary control parameter τ is certainly needed for a principal regime, given a one-to-one correspondence between (*x*, τ) and ($x_{1}$, $x_{2}$). However, we only need to define a dominant control parameter *x* on a characteristic line, which appears as a boundary in a principal regime. In addition, once a dominant control parameter *x* and an auxiliary control parameter τ are defined for a principal regime, their symmetric or dual images act as a dominant control parameter and an auxiliary control parameter for a non-principal regime that is symmetrical or dual in nature relative to the principal regime. Generically, *x* starts from a critical point $x_{c}$ or a discontinuous QPT point $x_{d}$ and ends at a point on a characteristic line, including a characteristic point. Such a characteristic line itself may start from a multi-critical point and describes a QPT belonging to a universality class different from what $x_{c}$ or $x_{d}$ belongs to. As a consequence, our emphasis is on irrelevant information instead of relevant information encoded in ground-state wave functions for a quantum many-body system. This is in contrast to local order parameters in Landau’s SSB theory, but it resembles real-space RG theories that merely manipulate high-energy degrees of freedom.

A few remarks are in order. (1) Two characteristic lines, as the boundaries in a principal regime, originate from two critical/transition points belonging to two different universality classes. (2) A critical point at infinity arises when one of the two coupling parameters $x_{1}$ and $x_{2}$ in a given regime is infinite in value, with an extra U(1) symmetry at this point. This appears to be a result of duality, if a self-dual point does not describe a critical point. (3) A characteristic point at infinity arises when one of the two coupling parameters $x_{1}$ and $x_{2}$ in a given regime is infinite in value, with an extra U(1) symmetry at this characteristic point. In particular, a factorized ground state occurs at this characteristic point. (4) A characteristic line at infinity arises when two coupling parameters $x_{1}$ and $x_{2}$ in a given regime are infinite in value in proportionality, with an extra U(1) symmetry on this characteristic line, if a factorized state occurs as a ground state. Here, we emphasize that, for a quantum many-body system, if one of the coupling parameters $x_{1}$ and $x_{2}$ is infinite in value, then there are two possibilities: It accommodates either a trivial factorized ground state or a critical point—a fact that remains unnoticed in the conventional theories. A point that deserves to be mentioned is that when we speak of a critical point at infinity or a characteristic point at infinity, we are referring to the original coupling parameters, $x_{1}$ and $x_{2}$, instead of a dominant control parameter and an auxiliary control parameter given the extent of a dominant control parameter *x* is, by definition, finite. Related to this is that a characteristic point at infinity is occasionally referred to as a characteristic line, since such a characteristic line at infinity should be regarded as a point, given that the Hamiltonian is essentially identical on a characteristic line located at infinity (at most up to a local unitary transformation).

#### 2.1.6. Nineteen Principal Regimes for the Six Illustrative Models

Here, we collect all nineteen principal regimes for the six illustrative models.

**Figure 1:**
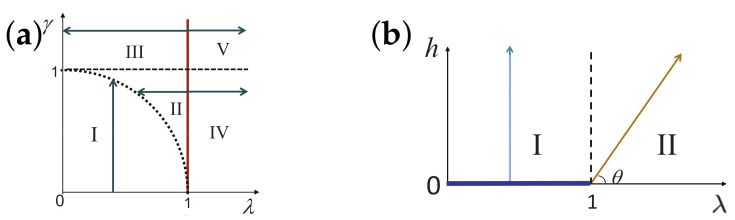
(**a**) Five principal regimes in the region λ≥0 and γ≥0 for the quantum spin-1/2 XY model. Here, arrows are used to indicate a dominant control parameter *x* for a fixed value of an auxiliary control parameter τ in the five principal regimes. (**b**) Two principal regimes for the transverse-field quantum Ising model in a longitudinal field, with h≥0 and λ≥0. Here, arrows are used to indicate a dominant control parameter *x* for a fixed value of an auxiliary control parameter τ in the two principal regimes.

**Figure 2:**
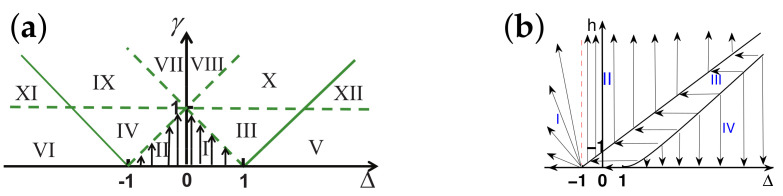
(**a**) Two principal regimes for the quantum spin-1/2 XYZ model with γ≥0. Here, arrows are used to indicate a dominant control parameter *x* for a fixed value of an auxiliary control parameter τ in the two principal regimes. We remark that regime I, regime III, regime V, regime VII, regime IX, and regime XI are dual in nature relative to each other, whereas regime II, regime IV, regime VI, regime VIII, regime X, and regime XII are dual relative to each other. Therefore, there are only two principal regimes with regime I and regime II as our choice. (**b**) Four principal regimes for the quantum spin-1/2 XXZ model in a magnetic field, with h≥0. Here, arrows are used to indicate a dominant control parameter *x* for a fixed value of an auxiliary control parameter τ in the four principal regimes, labelled as I, II, III, and IV, respectively.

**Figure 3:**
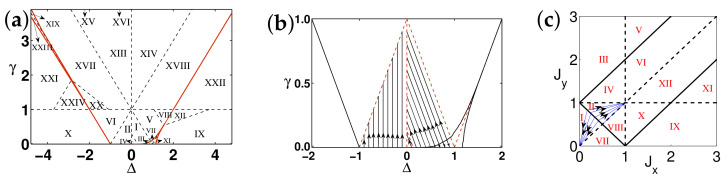
(**a**) Four principal regimes for the quantum spin-1 XYZ model, with γ≥0. Here, regime I, regime V, regime IX, regime XIII, regime XVII, and regime XXI are dual relative to each other; regime II, regime VI, regime X, regime XIV, regime XVIII, and regime XXII are dual relative to each other; regime III, regime VII, regime XI, regime XV, regime XIX, and regime XXIII are dual relative to each other, whereas regime IV, regime VIII, regime XII, regime XVI, regime XX, and regime XXIV are dual relative to each other. Therefore, there are only four principal regimes, with regime I, regime II, regime III, and regime IV as our choices. Note that regime XV and regime XVI only appear when γ is large, and they do not exist in the current parameter region when γ varies from 0 to 4. Here, their presence is only indicative. (**b**) Arrows are used to indicate a dominant control parameter *x* for a fixed value of an auxiliary control parameter τ in the four principal regimes for the quantum spin-1 XYZ model. (**c**) Two principal regimes in the region $J_{x}\geq 0$ and $J_{y}\geq 0$ for the spin-1/2 Kitaev model on a honeycomb lattice. Here, arrows are used to indicate a choice of a dominant control parameter *x* for a fixed value of an auxiliary control parameter τ in regime I and regime II. Here, regime I, regime III, regime V, regime VII, regime IX, and regime XI are dual relative to each other, whereas regime II, regime IV, regime VI, regime VIII, regime X, and regime XII are dual relative to each other. Therefore, there are only two principal regimes, with regime I and regime II as our choices.

There are five principal regimes in the quantum spin-1/2 XY model (1), labelled as I, II, III, IV, and V, which are shown in Figure 1a. There are two principal regimes in the transverse-field quantum Ising model in a longitudinal field (2), labelled as I and II, which are shown in Figure 1b. There are two principal regimes in the quantum spin-1/2 XYZ model (3), labelled as I and II, which are shown in Figure 2a. There are four principal regimes in the quantum spin-1/2 XXZ model in a magnetic field (4), labelled as I, II, III, and IV, which are shown in Figure 2b. There are four principal regimes in the quantum spin-1 XYZ model (5), labelled as I, II, III, and IV, which are shown in Figure 3a,b. There are two principal regimes in the spin-1/2 Kitaev model on a honeycomb lattice (6), labelled as I and II, which are shown in Figure 3c.

Meanwhile, a choice for a dominant control parameter *x* and an auxiliary control parameter τ is made in each of the nineteen principal regimes for the six illustrative models.

For the quantum spin-1/2 XY model (1), a dominant control parameter *x* is chosen to be γ, starting from $\gamma =\gamma_{c}=0$ up to the disordered circle, and an auxiliary control parameter τ is chosen to be λ in regime I. A dominant control parameter is chosen to be 1−λ, starting from $\lambda =\lambda_{c}=1$ up to the disordered circle: $\lambda^{2}+\gamma^{2}=1$, or λ=0; an auxiliary control parameter τ is chosen to be γ in regime II or regime III; a dominant control parameter is chosen to be 1−1/λ, starting from $\lambda =\lambda_{c}=1$ up to λ=∞, and an auxiliary control parameter τ is chosen to be γ in regime IV or regime V. This choice is to retain consistency with duality for the transverse-field quantum Ising model, corresponding to the quantum spin-1/2 XY model with γ=1.

For the transverse-field quantum Ising model in a longitudinal field (2), a dominant control parameter *x* is chosen to be x=h/(1+h), starting from x=0 up to x=∞, and an auxiliary control parameter τ is chosen to be λ in regime I. A dominant control parameter *x* is chosen to be x=r/(1+r), starting from x=0 up to x=1, and an auxiliary control parameter τ is chosen to be θ in regime II. Here, we have defined a radius *r* and an azimuthal angle θ: $r=\sqrt{{\left(\lambda -1\right)}^{2}+h^{2}}$ and θ=arctanh/(λ−1), with *r* ranging from $r=r_{c}=0$ to r=∞, but *x* ranges from $x_{c}=0$ to x=1. This choice is consistent with the requirement from the Kramers–Wannier duality when θ=0.

For the quantum spin-1/2 XYZ model (3), a dominant control parameter *x* is chosen to be γ, and an auxiliary control parameter τ is chosen to be Δ in regime I and regime II. Here, γ ranges from $\gamma =\gamma_{c}=0$ to γ=1−Δ for a fixed Δ∈(0,1) in regime I and to γ=1+Δ for a fixed Δ∈(−1,0) in regime II.

For the quantum spin-1/2 XXZ model in a magnetic field (4), a dominant control parameter *x* is chosen to be $x=\sqrt{{\left(\Delta +1\right)}^{2}+h^{2}}/\left(1+\sqrt{{\left(\Delta +1\right)}^{2}+h^{2}}\right)$, starting from the point (−1,0) up to the point (−∞,−∞), and an auxiliary control parameter τ is chosen to be τ=arctan(h/(Δ+1))∈(π/2,π] in regime I. A dominant control parameter *x* is chosen to be x=1−1/(h−Δ), starting from h=Δ+1 up to h=∞, and an auxiliary control parameter τ is chosen to be τ=Δ∈[−1,∞) in regime II. A dominant control parameter *x* is chosen to be x=−Δ, starting from $\Delta =\Delta_{c}$ up to Δ=h−1, and an auxiliary control parameter τ is chosen to be τ=h∈(0,∞) in regime III, with $\Delta_{c}$ being a QPT point on the line of the IC transition points for a fixed τ. A dominant control parameter *x* is chosen to be $x=h_{c}-h$, starting from $h=h_{c}$ up to h=0, and an auxiliary control parameter τ is chosen to be τ=Δ∈(1,∞) in regime IV. Here, an IC transition point represents an intermediate case interpolating between a KT transition and a PT transition.

For the quantum spin-1 XYZ model (5), a dominant control parameter *x* is chosen to be $x=\Delta_{c}/\left(2-\Delta_{c}\right)-\Delta /\left(2-\Delta \right)$, starting from the point $\left(\Delta_{c},\tau -\Delta_{c}\right)$, up to (0,τ), and an auxiliary control parameter τ is chosen to be τ=γ+Δ, ranging from $\Delta_{c1}$ to 1, in regime I, with ($\Delta_{c},\tau -\Delta_{c}$) denoting the Ising transition point between the Haldane phase and the ${\mathrm{AF}}_{x}$ phase, which is located on the straight line (γ=τ−Δ), for a fixed $\tau \in [\Delta_{c1},1]$, and $\Delta_{c1}$ denoting the critical value of Δ for the KT transition from the critical XY phase to the Haldane phase on the U(1)-symmetric line γ=0. A dominant control parameter *x* is chosen to be x=γ, starting from γ=0 up to γ=1+Δ, and an auxiliary control parameter τ is chosen to be τ=Δ∈(−1,0) in regime II. A dominant control parameter *x* is chosen to be $x=\Delta /\left(2-\Delta \right)-\Delta_{c}/\left(2-\Delta_{c}\right)$, starting from $\left(\Delta_{c},\tau -\Delta_{c}\right)$ up to (τ,0), and an auxiliary control parameter τ is chosen to be τ=γ+Δ, ranging from $\Delta_{c1}$ to 1, in regime III. A dominant control parameter *x* is chosen to be x=(γ+Δ)/(2−γ−Δ)−Δ/(2−Δ), and an auxiliary control parameter τ is chosen to be τ=γ+Δ in regime IV.

For the spin-1/2 Kitaev model on a honeycomb lattice (6), a dominant control parameter *x* is chosen to be $x=\sqrt{J_{x}^{2}+J_{y}^{2}}\left(1-J_{x}-J_{y}\right)/\left(J_{x}+J_{y}\right)$, starting from the critical point $\left(J_{x}/\left(J_{x}+J_{y}\right),\;J_{y}/\left(J_{x}+J_{y}\right)\right)$ up to the U(1)-symmetric point (0,0), and an auxiliary control parameter τ is chosen to be $\tau =J_{y}/J_{x}\in \left(1,\infty \right)$ in regime I; a dominant control parameter *x* is chosen to be $x=\sqrt{{\left(J_{x}-1\right)}^{2}+{\left(J_{y}-1\right)}^{2}}\left(J_{x}+J_{y}-1\right)/\left(2-J_{x}-J_{y}\right)$ starting from the critical point $\left(\left(J_{y}-1\right)/\left(J_{x}+J_{y}-2\right),\;\left(J_{x}-1\right)/\left(J_{x}+J_{y}-2\right)\right)$ up to the $S_{3}$-symmetric point (1,1), and an auxiliary control parameter τ is chosen to be $\tau =\left(J_{y}-1\right)/\left(J_{x}-1\right)\in \left(0,\;1\right)$ in regime II.

We remark that all the non-principal regimes are either symmetrical or dual to the principal regimes for the quantum spin-1/2 XYZ model (3), the quantum spin-1 XYZ model (5), and the spin-1/2 Kitaev model on a honeycomb lattice (6), which are subject to duality transformations (cf. Appendix C). A salient feature is that a principal regime and its dual image regimes share the same dominant control parameter *x* and the same auxiliary control parameter τ, though their mathematical expressions depend on a specific regime.

Throughout this study, if the chosen auxiliary control parameter τ is fixed, then we shall drop τ in the Hamiltonian H(x,τ) and fidelity mechanical-state functions to keep the notation simple unless otherwise stated.

### 2.2. A Fidelity Mechanical System and Its Environment

For a quantum many-body system described by a Hamiltonian H(x), if we treat *x* as a parameter varying with time *t*, then the time evolution is subject to the time-dependent Schrödinger equation, which is invariant under the time-reversal symmetry operation. In particular, as the adiabatic theorem [83] tells, if *x* slowly varies, then the system remains in a ground state if it is initially in a ground state, unless a critical point is crossed. We remark that an adiabatic shortcut to drive through a critical point for a quantum many-body system is possible, with an original idea from transitionless quantum driving [84], as discussed in Ref. [85]. However, everyday experience teaches us that we remember the past but not the future. This so-called psychological/computational arrow of time distinguishes the past from the future (for a brief summary about arrows of time, cf. Appendix D and for a list of arrows of time, see, e.g., [86]). A fundamental issue is to understand the ensuing consequences resulted from information storage, i.e., recording information encoded in the past states in media. As it turns out, information storage is a key ingredient in fidelity mechanics.

An outside observer, as an information processor, is equipped with a quantum copier tailored to a collection of mutually orthogonal states generated via a time evolution. Note that the no-cloning theorem does not rule out the possibility for copying a set of mutually orthogonal states (cf. Appendix E for more details about the no-cloning, no-deleting, and no-hiding theorems). For a (translation-invariant) quantum many-body system, the orthogonality follows from the translational invariance, combining with the fact that any quantum state may be represented in terms of a matrix-product state in one spatial dimension and a projected-entangled pair state in two and higher spatial dimensions, as long as the bond dimension is large enough [39,40,41,46,47,48]. In fact, as shown in [28], this is valid for any two translation-invariant states in the thermodynamic limit. Physically, this is related to the fact that more degrees of freedom are involved in a quantum system, more distinguishable its states are—a point relevant to the observation that “more is different” [87]. This enables us to turn quantum states at different instants, which arise from a time evolution, into quantum states at the same instant, recorded in media, via quantum copying. This is in sharp contrast to the case when one considers a quantum few-body system. For the latter, it is impossible to set up such a quantum copier. In a sense, one may regard time itself as a fictitious information eraser, which constantly deletes quantum states from a system during a quantum evolution. For quantum many-body systems, the states might be recovered from the environment, according to the no-cloning theorem and the no-hiding theorem (cf. Appendix E), since the states generated from a quantum evolution are orthogonal if the thermodynamic limit is approached, which makes them distinguishable. In contrast, for quantum few-body systems, the states generated from a quantum evolution are not orthogonal, and thus are not reliably distinguishable.

**Figure 4:**
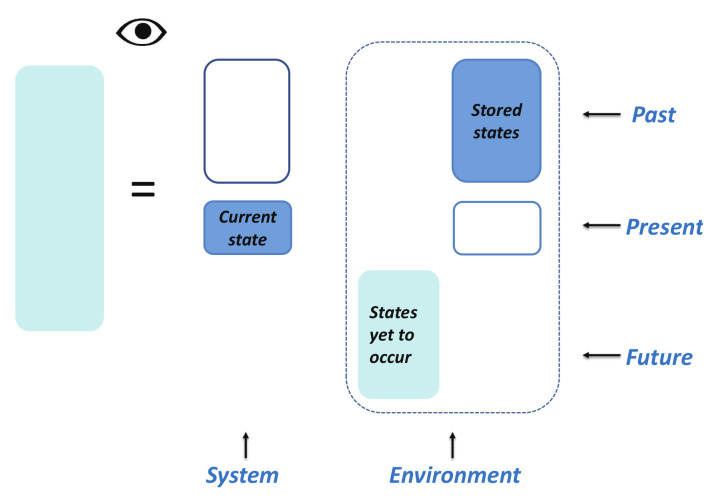
A fidelity mechanical system and its environment. A fidelity mechanical system is defined to be the current state stored in a medium. An environment consists of past states, which are stored in other media, and any possible states yet to occur in the future, which are simply left blank in media. Here, the current state, the past states, and the future states are associated with a quantum many-body system described by the Hamiltonian H(x), with *x* being a dominant control parameter, meaning that the ground-state energy density e(x) is monotonic with *x* and the range of *x* is finite. The present lies exactly at the intersection of the past and the future. Note that an outside observer, as an information processor, is equipped with a quantum copier tailored to a collection of mutually orthogonal states generated via a time evolution. Thus, a certain amount of information is extractable by comparing the current state with the past states, both of which are stored in media.

Now, we are ready to define a fidelity mechanical system and its environment. A fidelity mechanical system is defined to be the current state stored in a medium. An environment consists of the past states, which are stored in other media, and any possible states yet to occur in the future, which are simply left blank in media. The present lies exactly at the intersection of the past and the future. A pictorial representation for a fidelity mechanical system and its environment is presented in Figure 4.

Now, we turn to a description of a state for a given fidelity mechanical system. For this purpose, we introduce a quantum mechanical equivalent of the relaxation time scale in thermodynamics [88], which tells how much time a non-equilibrium state needs to adjust to an equilibrium state. From the adiabatic theorem, one knows that as long as the inverse of the gap is small enough, a quantum system starting its evolution from one ground state remains in another ground state. However, if it is driven at finite rate, then it will be excited. In fact, the inverse of the gap acts as a quantum mechanical equivalent of the relaxation time scale. Here, we remark that a relaxation time scale has been introduced to account for the Kibble-Zurek mechanism [89,90,91,92] describing the non-equilibrium dynamics and the formation of topological defects in a quantum many-body system, which is driven through a continuous QPT at finite rate [93,94,95]. Therefore, it is plausible to regard an adiabatic evolution as an analogue of a quasi-static process in thermodynamics. In fact, a fidelity mechanical system, with the current state stored in a medium being a ground state, is in equilibrium with its environment, with all the past states being ground states. Accordingly, a fidelity mechanical system, with the current state stored in a medium being a low-lying state, takes at least as much time as required by a quantum mechanical equivalent of the relaxation time scale in order to return to an equilibrium state with its environment. This allows us to define basic state functions, e.g., fidelity entropy, fidelity temperature, and fidelity internal energy, for a fidelity mechanical system.

### 2.3. Fidelity Entropy, Fidelity Temperature, and Fidelity Internal Energy: Continuous Quantum Phase Transitions

For a given fidelity mechanical system, which is in equilibrium with its environment, an important question is to quantify what amount of information may be recovered from the environment due to information storage that makes information encoded in the past states available. Here, it is proper to clarify what type of information we are trying to extract. In fact, we may categorize information into two different types: (i) information encoded in a given state, which may be quantified in terms of, e.g., entanglement entropy [60,96,97]. In this case, *only* one state is concerned, with quantum correlations at different spatial locations involved. Thus, this type of information is *spatial*; (ii) information extractable by comparing the current state with the past states, both of which are stored in media. Thus, different states at different temporal instants are involved. Therefore, this type of information is *temporal*. In fidelity mechanics, we solely deal with information of the second type.

For a continuous QPT, fidelity entropy S(x) is defined to characterize the uncertainty accumulated from a critical point $x_{c}$ to *x*

(9) $$S\left(x\right)=-2\int_{x_{c}}^{x}\ln d(x,y)dy+S_{0}.$$

Here, d(x,y) is the ground-state fidelity per lattice site for two ground states |ψ(x)〉 and |ψ(y)〉, and $S_{0}$ is an additive constant, representing residual fidelity entropy at a critical point. Fidelity entropy S(x) quantifies the amount of information that is extractable from comparing the current state at *x* with the stored states at *y* in the past. Actually, there is an interpretation for the first term in the definition of fidelity entropy S(x) in terms of Shannon entropy [36], if one regards the squared fidelity between two quantum states as a probability.

We assume that e(x) is always negative for any *x* (also cf. Appendix F, if e(x) is always positive for any *x*). Given fidelity entropy S(x), we need to define, in a consistent manner, fidelity internal energy U(x) and fidelity temperature T(x). Indeed, we define fidelity temperature T(x) as T(x)=∂U(x)/∂S(x), which implies that no fidelity work is involved when *x* is varied. This amounts to stipulating a rule that separates an increment of fidelity internal energy ΔU(x) into an increment of fidelity heat ΔQ(x) and an increment of fidelity work ΔW(x), with ΔQ(x)=T(x)ΔS(x). As defined, fidelity temperature is associated with a variation of a chosen dominant control parameter, whereas fidelity work is associated with a variation of an auxiliary control parameter. Here, we remark that we simply denote fidelity temperature by T(x), without concern about any confusion with physical temperature *T*, which is zero for QPTs. However, we note that, even at a finite temperature *T*, fidelity temperature T(x) may also be defined to quantify fluctuations (cf. Appendix F).

Suppose the Hamiltonian H(x) is defined by the Hamiltonian density h(x) acting locally on the Hilbert space for a translation-invariant quantum many-body system (for an extension to a non-translation-invariant quantum many-body system, cf. Appendix F). With the translational invariance in mind, we have 〈ψ(y)|h(x)|ψ(x)〉=e(x)〈ψ(y)|ψ(x)〉, with e(x) being the ground-state energy density. Given that 〈ψ(y)|ψ(x)〉 scales exponentially with *N*, it is reasonable to postulate that the dependence of fidelity internal energy U(x) on the ground-state energy density e(x) should be logarithmic. Hence, we define fidelity internal energy U(x) as follows:

(10) $$U\left(x\right)=\mp \ln\left(\frac{e(x)}{e(x_{c})}\right)V\left(x\right)+U_{0},$$

where V(x) is a quantity as a function of *x* that has yet to be determined consistently, and $U_{0}$ is an additive constant. Here, ∓ is introduced to ensure that V(x) is positive: −/+ corresponds to e(x) that is monotonically increasing/decreasing with *x*, respectively. Throughout this study, a statement that a function g(x) is monotonically increasing/decreasing with *x* should be understood in the sense that it monotonically increases/decreases as *x* increases. Here, g(x) represents any function of *x*. We remark that $e\left(x\right)/e\left(x_{c}\right)$ is monotonically decreasing with *x*, if e(x) is monotonically increasing with *x*, whereas $e\left(x\right)/e\left(x_{c}\right)$ is monotonically increasing with *x*, if e(x) is monotonically decreasing with *x*. It is proper to remark that fidelity entropy S(x) and fidelity internal energy U(x), as defined, should be understood as fidelity entropy per lattice site and fidelity internal energy per lattice site, respectively.

Given two undefined quantities V(x) and fidelity temperature T(x), we really need another constraint. As it turns out, such a constraint occurs in the guise of the analogue of Landauer’s principle at zero temperature: a certain amount of fidelity work needs to be performed to erase any information at zero temperature, due to quantum fluctuations. Logically, the internal consistency ascertains that the minimum fidelity work to be performed to erase one bit of information must be w(x)=∓T(x)ln2, with T(x) being fidelity temperature quantifying quantum fluctuations and −/+ corresponding to increasing/decreasing e(x) with *x*, respectively.

In Section 2.5, a key relation between fidelity temperature T(x) and V(x) is established from the analogue of Landauer’s principle at zero temperature

(11) $$T\left(x\right)=-\frac{\partial V(x)}{\partial x}\equiv -V_{x}\left(x\right).$$

Here, V(x) must be monotonically decreasing with *x* in order to guarantee that fidelity temperature T(x) is always positive. Combining this relation with the definition of fidelity temperature T(x):

(12) $$T\left(x\right)=\frac{\partial U(x)/\partial x}{\partial S(x)/\partial x},$$

we have the following

(13) $$T\left(x\right)=\mp \frac{{\left(\ln\left(e\left(x\right)/e\left(x_{c}\right)\right)\right)}_{x}V\left(x\right)+\ln e\left(x\right)/e\left(x_{c}\right)V_{x}\left(x\right)}{S_{x}\left(x\right)}.$$

Here, $S_{x}\left(x\right)\equiv \partial S(x)/\partial x$ and ${\left(\ln\left(e\left(x\right)/e\left(x_{c}\right)\right)\right)}_{x}\equiv \partial \ln\left(e\left(x\right)/e\left(x_{c}\right)\right)/\partial x$. This implies the following:

(14) $$V_{x}\left(x\right)=\alpha \left(x\right)\;V\left(x\right),$$

where α(x) is defined as follows

(15) $$\alpha \left(x\right)=\pm \frac{{\left(\ln\left(e\left(x\right)/e\left(x_{c}\right)\right)\right)}_{x}}{S_{x}\left(x\right)\mp \ln\left(e\left(x\right)/e\left(x_{c}\right)\right)}.$$

Here, α(x) is always negative for any *x*, consistent with the fact that V(x) monotonically decreases with *x*. We emphasize that α(x) is singular when a critical point $x_{c}$ is approached (cf. Appendix H for a scaling analysis). Therefore, Equation (14) is a singular first-order differential equation. It plays a fundamental role in fidelity mechanics. In fact, once it is solved, we will be able to determine fidelity internal energy U(x) and fidelity temperature T(x).

### 2.4. Fidelity Entropy, Fidelity Temperature, and Fidelity Internal Energy: Discontinuous Quantum Phase Transitions

For discontinuous QPTs, some modifications are needed. For a quantum many-body system undergoing a first-order QPT at a transition point $x_{d}$, fidelity entropy S(x) is defined as follows:

(16) $$S\left(x\right)=-2\int_{x_{d}}^{x}\ln d(x,y)dy+S_{0},$$

where $S_{0}$ is residual fidelity entropy at a discontinuous QPT point $x_{d}$. However, in order to retain consistency with the fact that fidelity temperature T(x) is finite at a discontinuous QPT point $x_{d}$, an additional parameter κ is introduced in fidelity internal energy U(x)

(17) $$U\left(x\right)=\mp \left[\ln\kappa +\ln\left(\frac{e(x)}{e(x_{d})}\right)\right]V\left(x\right)+U_{0}.$$

Here, V(x)>0 is an undetermined function of *x*, $U_{0}$ is an additive constant, and −/+ corresponds to monotonically increasing/decreasing e(x) with *x*, respectively. Note that fidelity temperature T(x) is again determined by $T\left(x\right)=-V_{x}$, since the same argument still applies to discontinuous QPTs. In fact, V(x) must be monotonically decreasing with *x*, in order to guarantee positive fidelity temperature T(x). Combining the definition of fidelity temperature T(x)=∂U(x)/∂S(x)=∂U(x)/∂x/∂S(x)/∂x with $T\left(x\right)=-V_{x}\left(x\right)$, we have the following:

(18) $$V_{x}\left(x\right)=\alpha_{d}\left(x\right)\;V\left(x\right),$$

where $\alpha_{d}\left(x\right)$ is defined as follows

(19) $$\alpha_{d}\left(x\right)=\pm \frac{(\ln{e\left(x\right)/e(x_{d}))}_{x}}{S_{x}\left(x\right)\mp \left(\ln\kappa +\ln\left(e\left(x\right)/e\left(x_{d}\right)\right)\right)}.$$

Note that, in contrast to continuous QPTs, $\alpha_{d}\left(x\right)$ is regular when a transition point $x_{d}$ is approached. Therefore, Equation (18) is a regular first-order differential equation. Once it is solved, we will be able to determine fidelity internal energy U(x) and fidelity temperature T(x).

### 2.5. Relation between an Unknown Function V(x) and Fidelity Temperature T(x)

Now we turn to the relation between an unknown function V(x) and fidelity temperature T(x), which has been exploited to derive differential equations for V(x) in the preceding subsections.

Consider a quantum many-body system described by the Hamiltonian H(x), with *x* being a dominant control parameter. The analogue of Landauer’s principle at zero temperature states that, in a fidelity mechanical system, to erase one bit of information at zero temperature, we need to perform the minimum fidelity work, w(x)=∓T(x)ln2, which quantifies the computational costs involved. Here, T(x) characterizes quantum fluctuations at zero temperature, and −/+ corresponds to monotonically increasing/decreasing ground-state energy density e(x) with *x*, respectively. Our task is to establish a relationship between an unknown function V(x) and fidelity temperature T(x).

Assume that the Hamiltonian H(x) is chosen to ensure that the ground-state energy density e(x) is negative. We prepare a composite system consisting of two identical copies. That is, the two copies share an identical Hamiltonian, but they are expressed in terms of different degrees of freedom ($H_{d}\left(x\right)$ and $H_{u}\left(x\right)$); then, one bit of information is encoded for each value of *x*. Therefore, the composite Hamiltonian $H_{c}\left(x\right)$ is $H_{c}\left(x\right)=H_{u}\left(x\right)+H_{d}\left(x\right)$. If we denote the ground-state energy density by e(x) for $H_{u}\left(x\right)$ and $H_{d}\left(x\right)$, then the ground-state energy density $e_{c}\left(x\right)$ for the composite Hamiltonian $H_{c}\left(x\right)$ is $e_{c}\left(x\right)=2\;e\left(x\right)$.

**Figure 5:**
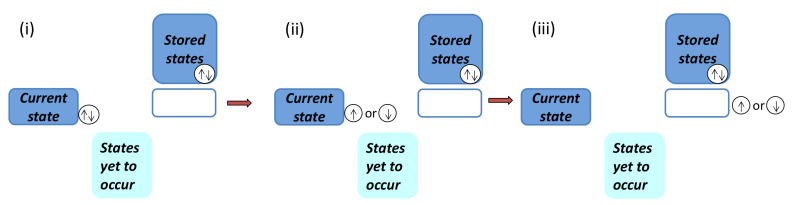
(**i**) A composite fidelity mechanical system consisting of two identical copies. Fidelity mechanical-state functions remain the same as a single-copy system. (**ii**) One copy is deleted from a composite fidelity mechanical system for a value of a dominant control parameter between *x* and x+Δx. To perform the deletion, a certain amount of fidelity work, quantifying the computational costs, ΔW needs to be performed, as required by the analogue of Landauer’s principle at zero temperature. (**iii**) The information about the retained copy is removed from the current state media and recorded in the information storage media.

To proceed further, we distinguish two cases:

(A) For a single-copy system, if the ground-state energy density e(x) monotonically decreases from a critical point $x_{c}$ to *x*, then fidelity internal energy U(x) takes the following form: $U\left(x\right)=\ln\left(e\left(x\right)/e\left(x_{c}\right)\right)V\left(x\right)+U_{0}$, with $U_{0}$ being an additive constant, and V(x) being positive. For a composite system consisting of two identical copies, fidelity mechanical-state functions remain the same as a single-copy system. This is illustrated in Figure 5i. Now, one copy is deleted from a composite fidelity mechanical system for a value of a dominant control parameter between *x* and x+Δx. To perform the deletion, a certain amount of fidelity work, quantifying the computational costs, ΔU(x)ΔW(x)=T(x)ln2Δx, needs to be performed, as required by the analogue of Landauer’s principle at zero temperature, to compensate for the increment of fidelity internal energy ΔU(x), as illustrated in Figure 5ii
  (20) $$\Delta \left(\ln\frac{e(x)}{2e(x_{c})}V\left(x\right)\right)=T\left(x\right)\Delta S\left(x\right)+\Delta W\left(x\right).$$
  As the last step, which is illustrated in Figure 5iii, the information about the retained copy is removed from the current state media and recorded in the information storage media. This amounts to extracting one bit of information for each value of a dominant control parameter between *x* and x+Δx, thus leading to a change in fidelity entropy: ΔS(x)→ΔS(x)−ln2Δx
  (21) $$\Delta \left(\ln\frac{e(x)}{e(x_{c})}V\left(x\right)\right)=T\left(x\right)\Delta \left(S\left(x\right)-\ln2\;x\right)+\Delta W\left(x\right).$$
  That is, T(x) is required to be related with V(x) as follows
  (22) $$T\left(x\right)=-\frac{\Delta V(x)}{\Delta x}.$$
  If Δx→0, then we have the following
  (23) $$T\left(x\right)=-V_{x}\left(x\right).$$
(B) For a single-copy system, if the ground-state energy density e(x) monotonically increases from a critical point $x_{c}$ to *x*, then fidelity internal energy U(x) takes the following form: $U\left(x\right)=-\ln\left(e\left(x\right)/e\left(x_{c}\right)\right)V\left(x\right)+U_{0}$, with $U_{0}$ being an additive constant, and V(x) being positive. For a composite system consisting of two identical copies, fidelity mechanical-state functions remain the same as a single-copy system. This is illustrated in Figure 5i. Now, one copy is deleted from a composite fidelity mechanical system for a value of a dominant control parameter between *x* and x+Δx. To perform this deletion, a certain amount of fidelity work, quantifying the computational costs, ΔU(x)ΔW(x)=−T(x)ln2Δx needs to be performed, as required by the analogue of Landauer’s principle at zero temperature, to compensate for the increment of fidelity internal energy ΔU(x), as illustrated in Figure 5ii:
  (24) $$-\Delta \left(\ln\frac{e(x)}{2e(x_{c})}V\left(x\right)\right)=T\left(x\right)\Delta S\left(x\right)+\Delta W\left(x\right).$$
  As the last step, which is illustrated in Figure 5iii, the information about the retained copy is removed from the current state media and recorded in the information storage media. This amounts to extracting one bit of information for each value of a dominant control parameter between *x* and x+Δx, thus leading to a change in fidelity entropy—ΔS(x)→ΔS(x)+ln2Δx:
  (25) $$-\Delta \left(\ln\frac{e(x)}{e(x_{c})}V\left(x\right)\right)=T\left(x\right)\Delta \left(S\left(x\right)+\ln2\;x\right)+\Delta W\left(x\right).$$
  That is, T(x) is required to be related with V(x) as follows
  (26) $$T\left(x\right)=-\frac{\Delta V(x)}{\Delta x}.$$
  If Δx→0, then we have the following
  (27) $$T\left(x\right)=-V_{x}\left(x\right).$$

### 2.6. A Contribution to Fidelity Entropy from Rescaling in the Ground-State Energy Density

For a quantum many-body system, the ground-state phase diagram exhibits distinct phases, each of which in turn is divided into different regimes as a result of the presence of characteristic lines. If the system admits dualities, then some regimes are dual in nature relative to each other. The implication for this fact is that one only needs to introduce a dominant control parameter *x* and an auxiliary control parameter τ in a few chosen regimes—the so-called principal regimes, with all the other regimes, dual relative to one of the principal regimes, being referred to as their dual image regimes under duality transformations. Occasionally, extra efforts have to be made to choose a proper dominant control parameter *x*, since the ground-state energy density e(x,τ) must be monotonic as a function of *x* for a fixed τ, or the range of *x* must be finite. That is, performing a re-parametrization operation in the Hamiltonian, $H\left(x_{1},x_{2}\right)\to H^{\omega }\left(x,\tau \right)$, is necessary to ensure the existence of a dominant control parameter *x*, where ω labels distinct principal regimes, and *x* and τ are functions of $x_{1}$ and $x_{2}$. In fact, there are two different types of rescaling operations: One originates from duality and the other originates from re-parametrization. Indeed, sometimes, a re-parametrization operation in the Hamiltonian is introduced to retain consistency with dualities if dualities only exist on a characteristic line in the control parameter space.

A re-parametrization operation in the Hamiltonian results in the introduction of a multiplying factor $m^{\omega }\left(x,\tau \right)>0$ such that the ground-state energy density $e(x_{1},x_{2})$ becomes $e^{\omega }\left(x,\tau \right)$: $e\left(x_{1},x_{2}\right)=m^{\omega }\left(x,\tau \right)\;e^{\omega }\left(x,\tau \right)$. Here, $e^{\omega }\left(x,\tau \right)$ and $m^{\omega }\left(x,\tau \right)$ must be monotonic as a function of *x* for a fixed τ, and there should be an $x_{0}$ such that $k(x_{0},\tau )=1$. In particular, if a multiplying factor $m^{\omega }\left(x,\tau \right)$ is simply equal to 1, then such a re-parametrization operation is trivial, with its meaning to be explained below. On the other hand, duality arises from a unitary transformation: $H\left(x_{1},x_{2}\right)=k^{\prime }\left(x_{1}^{\prime },x_{2}^{\prime }\right)UH\left(x_{1}^{\prime },x_{2}^{\prime }\right)U^{†}$, with *U* being a unitary transformation and $k^{\prime }\left(x_{1}^{\prime },x_{2}^{\prime }\right)>0$ being a function of $x_{1}^{\prime }$ and $x_{2}^{\prime }$. This implies that the ground-state energy density $e(x_{1},x_{2})$ becomes $e(x_{1}^{\prime },x_{2}^{\prime })$: $e\left(x_{1},x_{2}\right)=k\left(x_{1}^{\prime },x_{2}^{\prime }\right)e\left(x_{1}^{\prime },x_{2}^{\prime }\right)$. For convenience, we introduce $k\left(x_{1},x_{2}\right)\equiv k^{\prime }\left(x_{1}^{\prime },x_{2}^{\prime }\right)$. However, there exists a special type of duality transformations with $k(x_{1},x_{2})$ being equal to 1, which we refer to as a symmetric transformation. A detailed discussion about a duality transformation and its role in fidelity mechanics is deferred to Section 3.

**Figure 6:**
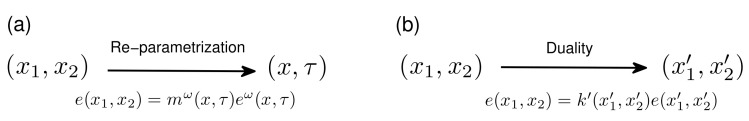
(**a**) A re-parametrization operation in the Hamiltonian $H(x_{1},x_{2})$ is performed, which induces rescaling in the ground-state energy density $e(x_{1},x_{2})$: $e\left(x_{1},x_{2}\right)=m^{\omega }\left(x,\tau \right)\;e^{\omega }\left(x,\tau \right)$. Here, $e^{\omega }\left(x,\tau \right)$ and $m^{\omega }\left(x,\tau \right)$ must be monotonic as a function of *x* for a fixed τ, and there should be an $x_{0}$ such that $k(x_{0},\tau )=1$. (**b**) Duality arises from a unitary transformation: $H(x_{1},x_{2})$$=k^{\prime }\left(x_{1}^{\prime },x_{2}^{\prime }\right)UH\left(x_{1}^{\prime },x_{2}^{\prime }\right)U^{†}$, with *U* being a unitary transformation and $k^{\prime }\left(x_{1}^{\prime },x_{2}^{\prime }\right)>0$ being a function of $x_{1}^{\prime }$ and $x_{2}^{\prime }$. This induces rescaling in the ground-state energy density $e(x_{1},x_{2})$: $e\left(x_{1},x_{2}\right)=k\left(x_{1}^{\prime },x_{2}^{\prime }\right)e\left(x_{1}^{\prime },x_{2}^{\prime }\right)$. For convenience, we introduce $k\left(x_{1},x_{2}\right)\equiv k^{\prime }\left(x_{1}^{\prime },x_{2}^{\prime }\right)$. There is a marked difference between the two types of rescaling operations: For a re-parametrization operation, (x,τ) and $\left(x_{1},\;x_{2}\right)$ represent two different ways of re-parametrization for the same Hamiltonian, representing the same point in the control parameter space, in contrast to a duality transformation, which connects two different points, denoted as $\left(x_{1},\;x_{2}\right)$ and $\left(x_{1}^{\prime },\;x_{2}^{\prime }\right)$, in the control parameter space.

There is a marked difference between the two types of rescaling operations, as demonstrated in Figure 6: For a re-parametrization operation, (x,τ) and $\left(x_{1},x_{2}\right)$ represent two different ways for parametrizing the coupling parameters for the same Hamiltonian, representing the same point in the control parameter space. In contrast, a duality transformation connects two different points $\left(x_{1},x_{2}\right)$ and $\left(x_{1}^{\prime },x_{2}^{\prime }\right)$, located in two dual regimes in the control parameter space. As a convention, we always assign $\left(x_{1}^{\prime },x_{2}^{\prime }\right)$ to represent a point in a principal regime, labelled as ω. Thus, $\left(x_{1},x_{2}\right)$ represents an image point of $\left(x_{1}^{\prime },x_{2}^{\prime }\right)$ in a dual image regime, labelled as α. Hence, we are able to introduce a dominant control parameter *x* and an auxiliary control parameter τ in the principal regime ω. That is, a one-to-one correspondence $\left(x_{1}^{\prime },x_{2}^{\prime }\right)\Leftrightarrow \left(x,\tau \right)$ is established in the principal regime ω, which may be regarded as a re-parametrization operation in the Hamiltonian $H(x_{1}^{\prime },x_{2}^{\prime })$, with $H\left(x_{1}^{\prime },x_{2}^{\prime }\right)=H^{\omega }\left(x,\tau \right)$ and $e^{\omega }\left(x_{1}^{\prime },x_{2}^{\prime }\right)=e^{\omega }\left(x,\tau \right)$. Here, we have assumed that the re-parametrization in the Hamiltonian $H(x_{1}^{\prime },x_{2}^{\prime })$ is trivial, when *x* and τ are chosen in the principal regime ω. However, this is not necessarily true. That is, it is possible to perform both types of rescaling operations in a non-principal regime. For the sake of simplicity, we restrict our considerations to a situation in which a re-parametrization operation in the Hamiltonian $H(x_{1}^{\prime },x_{2}^{\prime })$ is trivial when *x* and τ are chosen, since the extension is straightforward. By compiling everything, we have $H^{\varphi }\left(x_{1},x_{2}\right)=k_{\varphi \omega }\left(x,\tau \right)UH^{\omega }\left(x,\tau \right)U^{†}$ and $e^{\varphi }\left(x_{1},x_{2}\right)=k_{\varphi \omega }\left(x,\tau \right)e^{\omega }\left(x,\tau \right)$. Here, $k_{\varphi \omega }\left(x,\tau \right)$ is introduced via $k_{\varphi \omega }\left(x,\tau \right)\equiv k^{\prime }\left(x_{1}^{\prime },x_{2}^{\prime }\right)$, if $x_{1}^{\prime }$ and $x_{2}^{\prime }$ are regarded as functions of *x* and τ, respectively.

In addition, encountering a situation that a duality transformation only exists on a dual line in a principal regime is common. Then, this dual line may be divided into two different parts that are dual relative to each other. Similarly to a principal regime, one may define a principal part with its dual part being a non-principal part. With this in mind, our discussion is also applicable to a principal part on a dual line.

As a convention, we introduce “ω” to label the Hamiltonian and the ground-state energy density in distinct principal regimes: $H^{\omega }\left(x,\tau \right)$ and $e^{\omega }\left(x,\tau \right)$, with a dominant control parameter *x* and an auxiliary control parameter τ. Therefore, fidelity entropy $S^{\omega }\left(x,\tau \right)$, fidelity temperature $T^{\omega }\left(x,\tau \right)$, and fidelity internal energy $U^{\omega }\left(x,\tau \right)$ for the Hamiltonian $H^{\omega }\left(x,\tau \right)$ follow from our discussions above, with replacements $e\left(x\right)\to e^{\omega }\left(x,\tau \right)$ and $V\left(x\right)\to V^{\omega }\left(x,\tau \right)$. For continuous QPTs, we have the following:

(28) $$S^{\omega }\left(x,\tau \right)=-2\int_{x_{c}}^{x}\ln d^{\omega }\left(x,\tau ;y,\tau \right)dy+S_{0}^{\omega }\left(\tau \right),$$

(29) $$T^{\omega }\left(x,\tau \right)=-V_{x}^{\omega }\left(x,\tau \right),$$

and

(30) $$U^{\omega }\left(x,\tau \right)=\mp \ln\frac{e^{\omega }\left(x,\tau \right)}{e^{\omega }\left(x_{c},\tau \right)}V^{\omega }\left(x,\tau \right)+U_{0}^{\omega }\left(\tau \right),$$

where

(31) $$V_{x}^{\omega }\left(x,\tau \right)=\alpha^{\omega }\left(x,\tau \right)\;V^{\omega }\left(x,\tau \right),$$

with

(32) $$\alpha^{\omega }\left(x,\tau \right)=\pm \frac{\ln{\left(e^{\omega }\left(x,\tau \right)/e^{\omega }\left(x_{c},\tau \right)\right)}_{x}}{S_{x}^{\omega }\left(x,\tau \right)\mp \ln e^{\omega }\left(x,\tau \right)/e^{\omega }\left(x_{c},\tau \right)}.$$

For discontinuous QPTs, we have the following:

(33) $$S^{\omega }\left(x,\tau \right)=-2\int_{x_{d}}^{x}\ln d^{\omega }\left(x,\tau ;y,\tau \right)dy+S_{0}^{\omega }\left(\tau \right),$$

(34) $$T^{\omega }\left(x,\tau \right)=-V_{x}^{\omega }\left(x,\tau \right),$$

and

(35) $$U^{\omega }\left(x,\tau \right)=\mp \left(\ln\kappa +\ln\frac{e^{\omega }\left(x,\tau \right)}{e^{\omega }\left(x_{d},\tau \right)}\right)V^{\omega }\left(x,\tau \right)+U_{0}^{\omega }\left(\tau \right),$$

where

(36) $$V_{x}^{\omega }\left(x,\tau \right)=\alpha^{\omega }\left(x,\tau \right)\;V^{\omega }\left(x,\tau \right),$$

with

(37) $$\alpha^{\omega }\left(x,\tau \right)=\pm \frac{\ln{\left(e^{\omega }\left(x,\tau \right)/e^{\omega }\left(x_{d},\tau \right)\right)}_{x}}{S_{x}^{\omega }\left(x,\tau \right)\mp \left(\ln\kappa +\ln\left(e^{\omega }\left(x,\tau \right)/e^{\omega }\left(x_{d},\tau \right)\right)\right)}.$$

That is, an auxiliary control parameter τ, which has been dropped off for brevity, is reinserted into our formalism presented in Section 2.6.

Now, we are ready to introduce scaling entropy $S_{\sigma }^{\omega }\left(x,\tau \right)$ for a principal regime, labelled as ω, due to a re-parametrization operation, or scaling entropy $S_{\sigma }^{\varphi \omega }\left(x,\tau \right)$ for a dual regime, labelled as φ, due to a duality transformation. Physically, the presence of a multiplying factor $m^{\omega }\left(x,\tau \right)$ or $k_{\varphi \omega }\left(x,\tau \right)$, arising from a re-parametrization operation or a duality transformation, amounts to a variation of an energy scale, which undergoes updating in the information storage media, as *x* varies for a fixed τ. Therefore, it induces a fidelity heat exchange between a fidelity mechanical system and its environment, implying that information is recorded concerning an energy scale. This makes a contribution to fidelity entropy, with the variation of scaling entropy $S_{\sigma }^{\omega }\left(x,\tau \right)$ or $S_{\sigma }^{\varphi \omega }\left(x,\tau \right)$ being proportional to $\Delta m^{\omega }\left(x,\tau \right)/m^{\omega }\left(x,\tau \right)$ or $\Delta k_{\varphi \omega }\left(x,\tau \right)/k_{\varphi \omega }\left(x,\tau \right)$, respectively. The latter represents uncertainties due to variations in an energy scale. Here, we remark that the variation in an energy scale needs to be recorded in the information storage media, thus requiring the performance of a certain amount of fidelity work, quantifying the computational costs, to compensate for a variation of scaling entropy $S_{\sigma }^{\omega }\left(x,\tau \right)$ or $S_{\sigma }^{\varphi \omega }\left(x,\tau \right)$, according to the analogue of Landauer’s principle at zero temperature.

For a re-parametrization operation in the Hamiltonian, we have $e\left(x_{1},x_{2}\right)=m^{\omega }\left(x,\tau \right)$$e^{\omega }\left(x,\tau \right)$. Then, $S_{\sigma }^{\omega }\left(x,\tau \right)$ is defined as $S_{\sigma }^{\omega }\left(x,\tau \right)\equiv \pm \ln m^{\omega }\left(x,\tau \right)$, with the signs ± determined to retain consistency with the analogue of Landauer’s principle at zero temperature. As a result, fidelity entropy $S_{f}^{\omega }\left(x,\tau \right)$ consists of two parts: $S_{f}^{\omega }\left(x,\tau \right)=S^{\omega }\left(x,\tau \right)+S_{\sigma }^{\omega }\left(x,\tau \right)$, where $S^{\omega }\left(x,\tau \right)$ is the contribution to fidelity entropy from the ground-state fidelity per lattice site. Once fidelity entropy $S_{f}^{\omega }\left(x,\tau \right)$ is determined, fidelity entropy $S_{f}^{\omega }\left(x_{1},x_{2}\right)$ follows, since it takes the same value as $S_{f}^{\omega }\left(x,\tau \right)$. That is, $S_{f}^{\omega }\left(x_{1},x_{2}\right)\equiv S_{f}^{\omega }\left(x,\tau \right)$, when we move from *x* and τ to $x_{1}$ and $x_{2}$, meaning that *x* and τ are regarded as functions of $x_{1}$ and $x_{2}$. This is due to the fact that $\left(x_{1},x_{2}\right)$ and (x,τ) label the same point in a principal regime under a re-parametrization operation. We remark that if a multiplying factor $m^{\omega }\left(x,\tau \right)$ is equal to 1, then scaling entropy $S_{\sigma }^{\omega }\left(x,\tau \right)$ vanishes. This explains why such a re-parametrization operation is trivial.

For a duality transformation connecting a principal regime, labelled as ω, to a dual image regime, labelled as φ, we have $S_{\sigma }^{\varphi \omega }\left(x,\tau \right)\equiv \pm \ln k_{\varphi \omega }\left(x,\tau \right)$, with sign ± that is determined to retain consistency with the analogue of Landauer’s principle at zero temperature. As a result, scaling entropy $S_{\sigma }^{\varphi }\left(x,\tau \right)$ in a dual image regime, labelled as φ, consists of two parts as contributions from two types of rescaling operations—$S_{\sigma }^{\varphi }\left(x,\tau \right)=S_{\sigma }^{\omega }\left(x,\tau \right)+S_{\sigma }^{\varphi \omega }\left(x,\tau \right)$—with $S_{\sigma }^{\omega }\left(x,\tau \right)$ denoting scaling entropy from a re-parametrization operation in a principal regime, labelled as ω, and $S_{\sigma }^{\varphi \omega }\left(x,\tau \right)$ denoting scaling entropy from a duality transformation connecting a principal regime, labelled as ω, to a dual image regime, labelled as φ, if both a re-parametrization operation in a principal regime and a duality transformation connecting a principal regime to a dual image regime are present.

However, it is a bit involved to determine the signs ±. Mathematically, a duality transformation is induced from a (discrete) group, e.g., $Z_{2}$ or $S_{3}$, depending on a specific model under investigation. For a $Z_{2}$ group, there is only one generator; thus, there is no ambiguity to define a primary duality transformation. In contrast, for a duality transformation induced from the symmetric group $S_{3}$, ambiguities arise. For the models under investigation, we only need to consider the situation in which there is a subgroup $Z_{2}^{+}$ that induces a symmetric transformation. However, the subgroup $Z_{2}^{+}$ is not normal; therefore, we have to cope with a left or right coset. That is, when a modulo operation is performed on subgroup $Z_{2}^{+}$, there are still two nontrivial elements in the left or right coset, each of which generates a primary duality transformation in the sense that it is impossible for a primary duality transformation to be decomposed into two other primary duality transformations.

Needless to say, a choice of two primary duality transformations depends not only on the subgroup $Z_{2}^{+}$ but also on our choice of a dominant control parameter *x* and an auxiliary control parameter τ, which in turn depends on what regime we choose as a principal regime. Once the choice is made, we may then perform one primary duality transformation that connects a principal regime, labelled as ω, with the first dual image regime, labelled as φ, such that the signs in this dual image regime are determined: +/− corresponds to $e^{\omega }\left(x,\tau \right)$ that is monotonically increasing/decreasing with *x* for a fixed τ in a principal regime. Here, it is proper to make a generic remark that, in principle, one only needs to determine the sign ± from one *single* point in this dual image regime, since the discreteness of the values of the sign guarantees that the sign remains to be identical in the entire dual image regime. Therefore, restricting our consideration to the characteristic lines in a principal regime or its first dual image regime is legitimate as far as the determination of the sign is concerned. As a convention, we exploit “ω” to label a principal regime, with its first dual image regime and the second dual image regime being labelled as “φ” and “ρ”, respectively, if dualities are induced from the symmetric group $S_{3}$.

To proceed, we distinguish two distinct situations. First, a principal regime, labelled as ω, with the ground-state energy density being monotonically increasing/decreasing on one of its characteristic lines, shares a characteristic line with its first dual image regime, labelled as φ, with the ground-state energy density being monotonically decreasing/increasing on one of its characteristic lines, respectively. Two possibilities arise: (i) The ground-state energy density is not monotonic on the shared characteristic line. Then it is necessary to perform a re-parametrization operation on this characteristic line to ensure that the rescaled ground-state energy density is monotonically increasing/decreasing with a chosen dominant control parameter *x*, thus ensuring that the monotonicity is consistent in the principal regime. Meanwhile, an alternative re-parametrization operation is needed to ensure that the rescaled ground-state energy density is monotonically decreasing/increasing with the same dominant control parameter *x*, thus ensuring that the monotonicity is consistent in the first dual image regime. (ii) The shared characteristic line is a factorizing-field line, on which the rescaled ground-state energy density is a constant, after a re-parametrization operation is performed. As it turns out, the extent of the second dual image regime is not finite for the first situation. Hence, one may perform the first primary duality transformation to connect the principal regime, labelled as ω, with the first dual image regime, labelled as φ, and the second primary duality transformation to connect the first dual image regime, labelled as φ, with the second dual image regime, labelled as ρ, as if the first dual image regime, labelled as φ, was a principal regime. Hence, the signs of scaling entropy in the first and second dual image regimes are *solely* determined from the monotonicities of their respective (rescaled) ground-state energy densities with the respective dominant control parameters on their characteristic lines, according to the analogue of Landauer’s principle at zero temperature. That is, if the (rescaled) ground-state energy densities are *simultaneously* increasing or decreasing with their respective dominant control parameters on the characteristic lines in a principal regime, then a plus or minus sign arises in the first dual image regime, respectively. The same procedure may be repeated for the second primary duality transformation connecting the first dual image regime and the second dual image regime, as if the first dual image regime, labelled as φ, was a principal regime. Second, the extent of the control parameters $\left(x_{1},x_{2}\right)$ is finite and the monotonicity of the ground-state energy density with a dominant control parameter *x* is consistent on the two characteristic lines in a principal regime, labelled as ω. Then, one may perform the first primary duality transformation to connect the principal regime, labelled as ω, with the first dual image regime, labelled as φ. Hence, the sign of scaling entropy in the first dual image regime is determined from the monotonicity of the ground-state energy density with the chosen dominant control parameters on the characteristic lines in the principal regime, according to the analogue of Landauer’s principle at zero temperature. However, the extent of control parameters $\left(x_{1},x_{2}\right)$ is not finite or the monotonicity of the ground-state energy density with a dominant control parameter *x* is not consistent on the two characteristic lines in its first dual image regime, labelled as φ. Then, we have to return to the principal regime, labelled as ω, and perform the second primary duality transformation to connect the principal regime with the second dual image regime, such that the sign of scaling entropy in the second dual image regime is determined, according to the analogue of Landauer’s principle at zero temperature. In the first situation, the signs ± from the two primary duality transformations are not necessarily the same, since the sign ± in the first dual image regime is determined from the monotonicity of the (rescaled) ground-state energy density on the characteristic lines in the principal regime, labelled as ω, and the sign ± in the second dual image regime is determined from the monotonicity of the (rescaled) ground-state energy density on the characteristic lines in the first dual image regime, labelled as φ. In the second situation, the signs ± from the two primary duality transformations must be identical, since both signs ± are determined from the monotonicity of the ground-state energy density with a dominant control parameter *x* on the characteristic lines in the principal regime, labelled as ω.

In practice, this leads to a rule of thumb that scaling entropy $S_{\sigma }^{\omega }\left(x,\tau \right)$, $S_{\sigma }^{\varphi \omega }\left(x,\tau \right)$, $S_{\sigma }^{\rho \omega }\left(x,\tau \right)$, or $S_{\sigma }^{\rho \varphi }\left(x,\tau \right)$ monotonically increases with a dominant control parameter *x* for a fixed τ, since a principal regime and its dual image regimes share the same dominant control parameter *x* and the same auxiliary control parameter τ, though their mathematical expressions depend on a specific regime.

A supplementary rule is needed if the (rescaled) ground-state energy density is a constant, since the analogue of Landauer’s principle at zero temperature does not point to a specific sign. In this case, the signs are determined to ensure that scaling entropy $S_{\sigma }^{\omega }\left(x,\tau \right)$, $S_{\sigma }^{\varphi \omega }\left(x,\tau \right)$, $S_{\sigma }^{\rho \omega }\left(x,\tau \right)$, or $S_{\sigma }^{\rho \varphi }\left(x,\tau \right)$ monotonically increases with a dominant control parameter *x* for a fixed τ. In fact, this supplementary rule is not independent from the analogue of Landauer’s principle at zero temperature, since it is the only choice that is consistent with the rule of thumb.

A few remarks are in order. First, we have assumed that only two characteristic lines are involved in a given regime in the above discussion. This is not necessarily the case, since more than two characteristic lines are allowed in a regime. However, our discussion still applies, with the only change being that the monotonicity of the (rescaled) ground-state energy density with a dominant control parameter *x* is consistent on all characteristic lines in a given regime. Second, a soft line, as a peculiar type of characteristic line, is special in the sense that any two regimes separated by a soft line must share the same sign since the values of the signs are discrete, but a soft line may be continuously deformed. Therefore, one may treat the two regimes separated by a soft line as a composite regime as far as the determination of the signs is concerned. With this fact in mind, a principal composite regime shares a self-dual line with its first dual image composite regime, and the first dual image composite regime shares a self-dual line with the second dual image composite regime, for the models under investigation, if dualities are induced from the symmetric group $S_{3}$. Third, the discreteness of the values of the signs guarantees that the signs do not vary with a varying fictitious parameter σ connecting different choices of a dominant control parameter *x* and an auxiliary control parameter τ, which will be introduced in Section 4. Fourth, if the ground-state energy density is not monotonic on a characteristic line in a given regime, then it is always possible to perform a re-parametrization operation on this characteristic line such that the rescaled ground-state energy density monotonically increases or decreases with a properly chosen dominant control parameter *x* depending on a choice of a multiplying factor $m^{\omega }\left(x\right)$. We stress that such a choice is not unique, thus resulting in different fidelity mechanical-state functions. However, the underlying physics remains the same as far as the locations of both stable and unstable fixed points are concerned (cf. Section 4). Fifth, not all regimes, dual relative to each other under a duality transformation induced from the symmetric group $S_{3}$, are on the same footing, meaning that a regime may not be qualified as a principal regime. This happens if the monotonicity is not consistent for the ground-state energy densities with the chosen dominant control parameters on two characteristic lines, or if a one-to-one correspondence between the auxiliary control parameter in a regime and the dominant control parameter on one of its characteristic lines is not retained after a duality transformation induced from $Z_{2}$ is performed, if duality transformations induced from both $Z_{2}$ and $S_{3}$ are involved. Sixth, when duality transformations, induced from $Z_{2}$ and $S_{3}$, occur simultaneously in a specific model, we should first treat those induced from $S_{3}$. Once the signs for the regimes dual relative to each other under a duality transformation induced from $S_{3}$ are determined, one may treat them as a composite regime to determine the signs for the dual image regimes under a duality transformation induced from $Z_{2}$. Alternatively, one may focus on a principal regime and resort to the commutativity between a duality transformation induced from $S_{3}$ and a duality transformation induced from $Z_{2}$, to produce the same signs. Here, it is crucial to choose a proper principal regime that retains a one-to-one correspondence between an auxiliary control parameter in its dual image regime and a dominant control parameter on one of its characteristic lines under a duality transformation induced from $Z_{2}$. As it turns out, the rule of thumb is still valid in this generic case. Last but not least, our discussion about scaling entropy is also applicable to a dual image part, which is connected with a principal part via a duality transformation on a dual line.

Illustrative examples may be found in Appendix G for the quantum spin-1/2 XYZ model, the quantum spin-1 XYZ model and the spin-1/2 Kitaev model on a honeycomb lattice.

### 2.7. Shifts in Fidelity Temperature and Fidelity Internal Energy

Assume that fidelity entropy is determined in a principal part on a characteristic line and in a principal regime, with a proper choice of a dominant control parameter *x* and an auxiliary control parameter τ. Then, in order to determine fidelity temperature and fidelity internal energy, we need to solve a singular first-order differential equation, Equation (14), for continuous QPTs and a regular first-order differential equation, Equation (18), for discontinuous QPTs. This results in an integration constant $V_{0}^{\omega }\left(\tau \right)$ that has yet to be determined. Once this is performed, both fidelity temperature $T^{\omega }\left(x,\tau \right)$ and fidelity internal energy $U^{\omega }\left(x,\tau \right)$ follow, with an additive constant $U_{0}^{\omega }\left(\tau \right)$ that has yet to be determined.

On the other hand, it is necessary to introduce a shift in fidelity temperature $T^{\omega }\left(x,\tau \right)$→$T^{\omega }\left(x,\tau \right)-T_{0}^{\omega }\left(\tau \right)$, which in turn induces a shift in fidelity internal energy $U^{\omega }\left(x,\tau \right)$→$U^{\omega }\left(x,\tau \right)-T_{0}^{\omega }\left(\tau \right)S^{\omega }\left(x,\tau \right)$. This is due to the fact that fidelity temperature must be zero for an unentangled (factorized) state, since fidelity temperature is introduced to quantify quantum fluctuations. In addition, fidelity temperature must diverge, given strong quantum fluctuations present in a ground-state wave function, at a critical point. In fact, the divergence of fidelity temperature at a critical point is guaranteed from an observation that $\alpha^{\omega }\left(x,\tau \right)$ is singular when *x* approaches $x_{c}$ for a fixed τ (for a scaling analysis of fidelity entropy in the vicinity of a critical point/transition point, cf. Appendix H). In other words, a shift in fidelity temperature, accompanied by a shift in fidelity internal energy, emerges as a result of the internal logical consistency in fidelity mechanics.

A few remarks are in order. First, a shift in fidelity temperature $T^{q}\left(x\right)\to T^{q}\left(x\right)-T_{0}^{q}$, accompanied by a shift in fidelity internal energy $U^{q}\left(x\right)-T_{0}^{q}S^{q}\left(x\right)$, needs to be carried out for a principal part, labelled as *q*, on a characteristic line. Note that there is no need to introduce an auxiliary control parameter τ for a characteristic line. Therefore, when we refer to our prescription for a principal part on a characteristic line, it is proper to use mathematical equations from Equations (9)–(19). Second, a shift in fidelity temperature $T^{\omega }\left(x,\tau \right)\to T^{\omega }\left(x,\tau \right)-T_{0}^{\omega }\left(\tau \right)$, accompanied by a shift in fidelity internal energy $U^{\omega }\left(x,\tau \right)\to U^{\omega }\left(x,\tau \right)-T_{0}^{\omega }\left(\tau \right)S^{\omega }\left(x,\tau \right)$, needs to be carried out for a principal regime, with a proper choice of *x* and τ. Therefore, when we refer to our prescription for a principal regime, it is proper to use mathematical equations from Equations (28)–(37). Third, $T_{0}$ simply represents fidelity temperature at a characteristic point, evaluated from a dominant control parameter on a characteristic line originating from a chosen point on a line of critical points. Fourth, fidelity temperature is zero for an unentangled (factorized) state, and fidelity internal energy must be a constant for a factorizing-field line. Fifth, both fidelity temperature and fidelity internal energy are left intact if a duality transformation is performed, since they are determined *solely* from a choice of a dominant control parameter *x*.

### 2.8. Piecing Together All Regimes: The Continuity Requirements

Up until now, we have focused on a dominant control parameter *x* for a quantum many-body system described by the Hamiltonian $H^{\omega }\left(x,\tau \right)$ in a principal regime, labelled as ω. Now we turn to an auxiliary control parameter τ, which plays an important role, when we piece together all regimes. This leads to the continuity requirements for fidelity mechanical-state functions. Here, we stress that all regimes include principal regimes and non-principal regimes, since a duality transformation connects a principal regime with its dual image regimes—non-principal regimes—in the control parameter space.

#### 2.8.1. The Continuity Requirements for Fidelity Entropy: A Characteristic Point, a Characteristic Line, and a Principal Regime

Consider a principal regime, with a proper choice of a dominant control parameter *x* and an auxiliary control parameter τ. Then, fidelity entropy follows from definition (28). Suppose the principal regime is enclosed by the boundaries consisting of a line of critical points, with one endpoint being a multi-critical point and the other a characteristic point, and two characteristic lines: One originates from the multi-critical point, and the other originates from the characteristic point on the line of critical points. Note that the two characteristic lines meet each other at another characteristic point away from the line of critical points. Once a proper choice of a dominant control parameter *x* is made in a principal part on a characteristic line, we are able to determine fidelity entropy according to definition (9). Since fidelity entropy is *relative*, in a sense that it is only determined up to an additive constant, one may set the residual fidelity entropy to zero at a chosen critical point. One preferred choice is the characteristic point located on the line of critical points, meaning that fidelity entropy is zero at this characteristic point. Then, fidelity entropy on the characteristic line originating from this characteristic point is determined, according to definition (9). With this in mind, fidelity entropy on a characteristic line originating from a multi-critical point follows from the continuity requirement for fidelity entropy at the characteristic point away from the line of critical points. In particular, the residual fidelity entropy at the multi-critical point is determined. As such, one may determine the residual fidelity entropy on the line of critical points from the continuity requirement for fidelity entropy on the characteristic line originating from a multi-critical point. In other words, fidelity entropy is determined in this principal regime.

Once this is performed, we move to an adjacent principal regime and repeat the procedure to determine the residual fidelity entropy on a line of critical points in this principal regime. The procedure is repeated until all lines of critical points are exhausted.

This ensures *continuity* for fidelity entropy on the boundaries between any two principal regimes or a principal regime and its dual image regime in the control parameter space. Note that fidelity entropy may not be single-valued at a characteristic point or on a characteristic line, which will be discussed in Section 6.

#### 2.8.2. The Continuity Requirements for Fidelity Temperature and Fidelity Internal Energy: A Characteristic Line

Let us now discuss fidelity temperature and fidelity internal energy for continuous QPTs in a principal part, labelled as *k*, on a characteristic line originating from a multi-critical point. The latter itself appears to be an endpoint on a line of critical points. Here, *k* labels different principal parts on characteristic lines for a given model. A shift in fidelity temperature $T^{k}\left(x\right)\to T^{k}\left(x\right)-T_{0}^{k}$ is performed to ensure that fidelity temperature is at zero or minimum at a characteristic point, which is an intersection point between two characteristic lines depending on whether the ground-state wave function is in an unentangled (factorized) state or an entangled state. This in turn induces a shift in fidelity internal energy $U^{k}\left(x\right)\to U^{k}\left(x\right)-T_{0}^{k}S\left(x\right)$, where $T_{0}^{k}$ represents fidelity temperature at the characteristic point evaluated from the multi-critical point, with *x* being a dominant control parameter on the characteristic line.

#### 2.8.3. The Continuity Requirements for Fidelity Temperature and Fidelity Internal Energy: A Principal Regime

We turn to fidelity temperature and fidelity internal energy for continuous QPTs in a principal regime. Suppose that we solved a singular first-order differential equation, Equation (14), with *x* being a chosen dominant control parameter in the principal regime. This results in an integration constant $V_{0}^{\omega }\left(\tau \right)$.

In order to ensure that the continuity requirement for fidelity temperature is satisfied, a shift in fidelity temperature $T^{\omega }\left(x,\tau \right)\to T^{\omega }\left(x,\tau \right)-T_{0}^{\omega }\left(\tau \right)$ is performed, accompanied by a shift in fidelity internal energy $U^{\omega }\left(x,\tau \right)\to U^{\omega }\left(x,\tau \right)-T_{0}^{\omega }\left(\tau \right)S^{\omega }\left(x,\tau \right)$. Generically, $T_{0}^{\omega }\left(\tau \right)\equiv T_{m}^{\omega }\left(\tau \right)-T_{t}^{\omega }$, where $T_{m}^{\omega }\left(\tau \right)$ represents fidelity temperature at a chosen point on a characteristic line originating from a multi-critical point, evaluated from a dominant control parameter *x* in this principal regime, whereas $T_{t}^{\omega }$ represents fidelity temperature at the same point, which is determined from a dominant control parameter *x* on this characteristic line itself. We emphasize that the chosen point is now parametrized as $\left(x_{1},x_{2}\right)$ in the control parameter space, (x,τ) in a principal regime, and *x* in a principal part on a characteristic line. A convenient method for addressing this ambiguity is to choose τ to label the chosen point on a characteristic line originating from a multi-critical point, when the continuity requirements for fidelity temperature and fidelity internal energy in a principal regime is implemented. To implement this, establishing a relation between the auxiliary control parameter τ in the principal regime and the dominant control parameter in the principal part on the characteristic line is necessary, which is a one-to-one correspondence.

Once this is performed, an additive constant $U_{0}^{\omega }\left(\tau \right)$ in fidelity internal energy and an integration constant $V_{0}^{\omega }\left(\tau \right)$ are determined from the continuity requirements for fidelity temperature $T^{\omega }\left(x,\tau \right)$ and fidelity internal energy $U^{\omega }\left(x,\tau \right)$ on a characteristic line originating from a multi-critical point, with an extra condition that fidelity internal energy $U^{\omega }\left(x,\tau \right)$ is zero at a critical point. This extra condition will be justified in the next Subsection.

After shifts in fidelity temperature and fidelity internal energy are implemented, and $V_{0}^{\omega }\left(\tau \right)$ and $U_{0}^{\omega }\left(\tau \right)$ are determined, we are able to arrive at the final results for fidelity temperature $T_{f}^{\omega }\left(x,\tau \right)$ and fidelity internal energy $U_{f}^{\omega }\left(x,\tau \right)$. We introduce $T_{f}^{\omega }\left(x_{1},x_{2}\right)\equiv T_{f}^{\omega }\left(x,\tau \right)$ and $U_{f}^{\omega }\left(x_{1},x_{2}\right)\equiv U_{f}^{\omega }\left(x,\tau \right)$ when we move from *x* and τ to $x_{1}$ and $x_{2}$, meaning that *x* and τ are regarded as functions of $x_{1}$ and $x_{2}$, respectively.

#### 2.8.4. The Continuity Requirements for Fidelity Temperature and Fidelity Internal Energy: Discontinuous Phase Transitions

The above discussion also applies to discontinuous QPTs, with a modification that an additive constant $U_{0}^{\omega }\left(\tau \right)$ in fidelity internal energy, an integration constant $V_{0}^{\omega }\left(\tau \right)$, and an extra parameter κ are determined from the continuity requirements for fidelity temperature and fidelity internal energy at a transition point $x_{d}$ on a line of discontinuous QPT points, which ends at an isolated critical point. Here, the line of the discontinuous QPT points itself is a part of a symmetric line, with the symmetry group being discrete. With this fact in mind, we remark that a line of discontinuous QPT points results from SSB. That is, there is a continuous QPT at a critical point $x_{c}$, due to SSB, on the symmetric line with a discrete symmetry group. In other words, this continuous QPT at a critical point $x_{c}$ is protected by the discrete symmetry group. Away from the characteristic line, the model under investigation does not enjoy any symmetry except for a characteristic line, which either ends or is located at infinity.

#### 2.8.5. Piecing Together Principal Regimes and Non-Principal Regimes (If Any)

Once fidelity mechanical-state functions in all the principal regimes as well as on the characteristic lines are determined, we are able to determine fidelity mechanical-state functions in all non-principal regimes as dual image regimes by taking into account the contribution from scaling entropies arising from re-parametrization operations or dualities. This allows us to piece together all regimes to visualize fidelity mechanical-state functions in the entire control parameter space.

Specific examples to illustrate how our prescription is implemented for quantum many-body systems may be found in Section 7 for the quantum spin-1/2 XY model, in Section 8 for the transverse-field quantum Ising model in a longitudinal field, in Section 9 for the quantum spin-1/2 XYZ model, in Section 10 for the quantum spin-1/2 XXZ model in a magnetic field, in Section 11 for the quantum spin-1 XYZ model, and in Section 12 for the spin-1/2 Kitaev model on a honeycomb lattice.

### 2.9. Generic Remarks

We are able to draw some consequences from our argument above, combining the discussions about duality in Section 3, about an interior point of view vs exterior point of view in Section 6 and about fidelity flows in Section 13. First, the residual fidelity entropy $S_{0}$ depends on a specific choice of a dominant control parameter *x* in a principal regime; thus, it does not reflect information encoded in the ground-state wave function at a critical point. That is, it is *extrinsic* in the sense that it is impossible to determine it from the Hamiltonian itself at a critical point. Actually, this observation leads us to an interior point of view vs. an exterior point of view in Section 6. Loosely speaking, it also makes sense to speak of the residual fidelity entropy in a principal part or a principal regime, though it is defined at a critical point. Second, there are lower and upper bounds for fidelity internal energy U(x), although this statement is only valid for fidelity internal energy, determined from a specific Hamiltonian with the ground-state energy density being negative. This means that the Hamiltonian is in a canonical form with respect to a specific definition of duality (cf. Section 3). This is due to the fact that the range of a dominant control parameter *x* is finite. As a convention, we always choose the lower bound to be zero. However, as discussed in Section 3, for a Hamiltonian with the ground-state energy density changing its sign, fidelity internal energy diverges when the ground-state energy density is zero. Third, at a critical point, fidelity internal energy U(x) must be zero, thus leading to the requirement that $U_{0}=T_{0}\;S_{0}$. Physically, this is a consequence of the fact that, at a critical point, it is impossible to extract any relevant information by discarding irrelevant information, since any relevant information is covered up by irrelevant information. Mathematically, at a critical point, fidelity internal energy becomes $U_{0}-T_{0}\;S_{0}$, which has to satisfy $U_{0}-T_{0}\;S_{0}\geq 0$, due to the convention that the lower bound is zero. Indeed, if it takes a positive value, then it is impossible to guarantee that fidelity internal energy U(x) monotonically increases with *x*. That is, the internal logical consistency demands that fidelity internal energy U(x) must be zero at a critical point. Fourth, for a given quantum many-body system, fidelity internal energy U(x) takes the same value at all stable fixed points. This reflects the fact that the Hamiltonians are unitarily equivalent at all stable fixed points. Fifth, fidelity temperature is zero on a factorizing-field line given that no quantum fluctuations are present.

As mentioned, two characteristic lines intersect with each other at a characteristic point in the control parameter space. Such a characteristic point may occur on or away from a line of critical points. If it occurs away from a line of critical points, then it is identified as a stable or metastable fixed point from an exterior point of view; if it appears on a line of critical points, then it is identified as a stable fixed point from an interior point of view, as follows from our discussion about fidelity flows in Section 13: A stable fixed point is characterized by a zero-fidelity temperature and (local) maximal fidelity entropy; a metastable fixed point is characterized by the minimum fidelity temperature and (local) maximal fidelity entropy. Instead, any critical point, located on a line of critical points, is identified as an unstable fixed point from an exterior point of view. On the other hand, a multi-critical/multi-transition point, located at one endpoint of a line of critical points, is identified as an unstable fixed point from an interior point of view, characterized in terms of divergent fidelity temperature, as a result of the fact that α(x), defined in Equation (15), diverges at such a multi-critical/multi-transition point. Here, we remark that a characteristic point at infinity is labelled in terms of the two original coupling parameters, with one of them being infinite in value. As it turns out, keeping the other finite coupling parameters in labelling a characteristic point at infinity is necessary given that the symmetry group varies with the two coupling parameters.

Note that different choices are allowed for a dominant control parameter *x* in a principal regime. However, different choices lead to different fidelity mechanical-state functions. Therefore, one may raise a concern whether or not it is possible to extract any sensible physics from our formalism. This concern has been addressed in Section 4. As argued, any two different sets of fidelity entropy, fidelity temperature, and fidelity internal energy resulting from two different choices are related to each other via introducing a fictitious parameter σ. Actually, information encoded in σ arising from different choices of a dominant control parameter for a given regime is *irrelevant* in the sense that both stable and unstable fixed points remain the same. Physically, this is due to the fact that the constraints imposed by symmetries, dualities and factorizing fields are *rigid*, meaning that there is no flexibility in choosing a dominant control parameter *x* on such a characteristic line, although it is still allowed to perform a re-parametrization operation in the ground-state energy density on a characteristic line subject to the condition that, for any two re-parametrization operations, a dominant control parameter from one re-parametrization operation must be monotonically increasing with that from the other re-parametrization operation and vice versa. Although this does change fidelity mechanical-state functions, it does not change where fidelity temperature diverges or becomes zero and does not change where fidelity entropy takes a (local) maximum. In practice, we may take advantage of this freedom to properly choose a dominant control parameter, *x*, such that the numerical simulation is more efficient when we exploit a tensor network algorithm [37,38,39,40,41,46,47,48] to simulate quantum many-body systems. As an illustrative example, we choose a dominant control parameter *x* in different ways for the quantum spin-1/2 and spin-1 XYZ models to demonstrate different features in various aspects.

We emphasize that, once fidelity mechanical-state functions are determined in all principal regimes and their symmetric or dual image regimes, we have to transform back to the original coupling parameters, $x_{1}$ and $x_{2}$, according to a one-to-one correspondence between ($x_{1},x_{2}$) and (x,τ). As a convention, we use a subscript *f* to indicate fidelity mechanical-state functions, with the original coupling parameters as their arguments, for a specific quantum many-body system. Note that the subscript, *f*, is also exploited to indicate the final outcome for fidelity mechanical-state functions, with a dominant control parameter *x* and an auxiliary control parameter τ as their arguments.

## 3. A Shift Operation in the Hamiltonian: Duality and a Canonical Form of the Hamiltonian

Given that ground-state energy density $e(x_{1},x_{2})$ is involved in our formalism, an important question remains: What form of the Hamiltonian should be chosen given the Hamiltonian is mathematically determined up to a constant multiplying factor and an additive constant? As is well known, for a given Hamiltonian $H(x_{1},x_{2})$, the physics itself does not change under two operations: One is a constant multiplying operation and the other is a shift operation, although Hamiltonian $H(x_{1},x_{2})$ becomes $H^{*}\left(x_{1},x_{2}\right)=gH\left(x_{1},x_{2}\right)+b$, with g>0 and *b* being real numbers. The operations in the Hamiltonian $H(x_{1},x_{2})$ induce a change in the ground-state energy density $e(x_{1},x_{2})$: $e^{*}\left(x_{1},x_{2}\right)=ge\left(x_{1},x_{2}\right)+b$. Therefore, our question may be reshaped as follows. What is a canonical form of the Hamiltonian *H* in fidelity mechanics? The answer rests on a well-known notion: duality.

Generically, duality is nothing but a unitary mapping between quantum Hamiltonians that preserves the quasi-local character of their interaction terms (see, e.g., [3,76]). Mathematically, this corresponds to $H\left(x_{1},x_{2}\right)=k^{\prime }\left(x_{1}^{\prime },x_{2}^{\prime }\right)UH^{\prime }\left(x_{1}^{\prime },x_{2}^{\prime }\right)U^{†}$, where $H^{\prime }\left(x_{1}^{\prime },x_{2}^{\prime }\right)$ is the Hamiltonian unitarily equivalent to $H(x_{1},x_{2})$, *U* is a unitary operator, and $k^{\prime }\left(x_{1}^{\prime },x_{2}^{\prime }\right)>0$. Dualities are of special interest, which are unitary mappings conserving the form of Hamiltonian operator $H(x_{1},x_{2})$. That is, $H\left(x_{1},x_{2}\right)=k^{\prime }\left(x_{1}^{\prime },x_{2}^{\prime }\right)UH\left(x_{1}^{\prime },x_{2}^{\prime }\right)U^{†}$, with $x_{1}^{\prime }$ and $x_{2}^{\prime }$ being functions of $x_{1}$ and $x_{2}$. In this study, we only refer to this form of unitary mapping as dualities. In other words, duality is one of the two types of rescaling operations in the Hamiltonian, with the only difference that duality always involves a nontrivial unitary transformation. A prototypical example is the Kramers–Wannier duality for the transverse-field quantum Ising model [3,76]. Physically, this duality allows us to relate the weak-coupling regime to the strong-coupling regime.

It is important to note that duality leaves no room for the Hamiltonian $H^{*}\left(x_{1},x_{2}\right)$ but a constant multiplying factor. That is, for a fixed *g*, there is only one value of *b* such that duality exists in the corresponding Hamiltonian. Therefore, one may choose a specific form of the Hamiltonian *H* among equivalent Hamiltonians, related via $H^{*}\left(x_{1},x_{2}\right)$$=gH(x_{1},x_{2})+b$, up to a constant multiplying factor. This form is a *canonical form* of the Hamiltonian in fidelity mechanics, meaning that it only makes sense to adopt the ground-state energy density $e(x_{1},x_{2})$ from a canonical form of the Hamiltonian in Equations (15) and (19) to determine fidelity mechanical-state functions. We remark that, generically, duality is lacking in a given Hamiltonian. However, we are still able to define a canonical form in such a case; there are three ways to do so. First, for a Hamiltonian depending on at least one coupling parameter, we may find a special case that hosts duality, thus enabling us to determine a specific value of *b*, as is the case for the quantum spin-1/2 XY model. For this model, the transverse-field quantum Ising model as a special case does host duality. Second, for a Hamiltonian without any coupling parameter (except for a constant multiplying factor as an energy scale), we may introduce more coupling constants by embedding a given Hamiltonian into a more general Hamiltonian with more than two coupling parameters and try to see if there is any special case that can host duality. This happens to the quantum spin-1/2 XXX model and the quantum spin-1/2 XXZ model, which may be extended to the quantum spin-1/2 XYZ model. The latter hosts duality, as discussed in Appendix C. Third, an established canonical form of a given Hamiltonian may be exploited to justify a canonical form of a related Hamiltonian, which reduces to the given Hamiltonian in some limit. This happens to the t-J model, as it reduces to the quantum spin-1/2 XXX model at half filling.

We stress that the presence of a constant multiplying factor does not change fidelity entropy $S_{f}\left(x_{1},x_{2}\right)$ (up to an additive constant), fidelity temperature $T_{f}\left(x_{1},x_{2}\right)$, and fidelity internal energy $U_{f}\left(x_{1},x_{2}\right)$ as long as it is kept constant. In contrast, extra attention needs to be paid to the shift operation, $H^{*}\left(x_{1},x_{2}\right)=H\left(x_{1},x_{2}\right)+b$. Suppose $H(x_{1},x_{2})$ is in a canonical form, then, generically, we have $e(x_{1},x_{2})<0$. Thus, fidelity entropy $S_{f}\left(x_{1},x_{2}\right)$, fidelity temperature $T_{f}\left(x_{1},x_{2}\right)$, and fidelity internal energy $U_{f}\left(x_{1},x_{2}\right)$ follow accordingly, with $V(x_{1},x_{2})$ determined from the singular first-order differential equation, Equation (14), for continuous QPTs and the regular first-order differential equation, Equation (18), for discontinuous QPTs. Therefore, we focus on a shift operation in the Hamiltonian, $H^{*}\left(x_{1},x_{2}\right)=H\left(x_{1},x_{2}\right)+b$, which induces a change in the ground-state energy density $e(x_{1},x_{2})$: $e^{*}\left(x_{1},x_{2}\right)=e\left(x_{1},x_{2}\right)+b$.

Our discussion up until this point leaves an impression that a canonical form of a given Hamiltonian seems to occupy a unique position in fidelity mechanics. However, this is not true, since the definition of a canonical form of the Hamiltonian depends on the definition of duality, which in turn depends on a shift operation in the Hamiltonian.

To see this point, we assume $H(x_{1},x_{2})$ is in a canonical form. Our definition for duality, which has been adopted, is conventional (see, e.g., [3]). If the definition of duality is modified, then a canonical form of the Hamiltonian follows. Suppose the definition of duality is modified to be $H^{*}\left(x_{1},x_{2}\right)=k^{\prime }\left(x_{1}^{\prime },x_{2}^{\prime }\right)UH^{*}\left(x_{1}^{\prime },x_{2}^{\prime }\right)U^{†}+\mu \left(x_{1}^{\prime },x_{2}^{\prime }\right)$, with an extra parameter $\mu (x_{1}^{\prime },x_{2}^{\prime })$. The task is to find out a proper $\mu (x_{1}^{\prime },x_{2}^{\prime })$ to ensure that $H^{*}\left(x\right)$ is in a canonical form according to this modified definition for duality. As it turns out, we have $\mu \left(x_{1}^{\prime },x_{2}^{\prime }\right)=b\left(1-k^{\prime }\left(x_{1}^{\prime },x_{2}^{\prime }\right)\right)$.

Following our prescription in Section 2, fidelity entropy $S_{f}^{*}\left(x_{1},x_{2}\right)$, fidelity temperature $T_{f}^{*}\left(x_{1},x_{2}\right)$, and fidelity internal energy $U_{f}^{*}\left(x_{1},x_{2}\right)$ are equally well-defined as long as $e^{*}\left(x_{1},x_{2}\right)$ is negative. Here, we have introduced a superscript, *, to indicate the dependence of fidelity mechanical-state functions on *b*. Given that ground-state wave functions remain the same, we have $S_{f}^{*}\left(x_{1},x_{2}\right)=S_{f}\left(x_{1},x_{2}\right)$. However, fidelity temperature $T_{f}^{*}\left(x_{1},x_{2}\right)$ and fidelity internal energy $U_{f}^{*}\left(x_{1},x_{2}\right)$ depend on *b*. Here, we have retained the same choice of a dominant control parameter, *x*, in a given regime.

Physically, the dependence of both fidelity temperature $T_{f}^{*}\left(x_{1},x_{2}\right)$ and fidelity internal energy $U_{f}^{*}\left(x_{1},x_{2}\right)$ on *b* simply means that information about *b* is updated in the information storage media as *b* varies. As a consequence, a certain amount of fidelity work $W_{f}^{*}\left(x_{1},x_{2}\right)$ is required to compensate for changes in both fidelity temperature $T_{f}^{*}\left(x_{1},x_{2}\right)$ and fidelity internal energy $U_{f}^{*}\left(x_{1},x_{2}\right)$:

(38) $$W_{f}^{*}\left(x_{1},x_{2}\right)=\int \left(dU_{f}^{*}\left(x_{1},x_{2}\right)-T_{f}^{*}\left(x_{1},x_{2}\right)dS_{f}^{*}\left(x_{1},x_{2}\right)\right)=U_{f}^{*}\left(x_{1},x_{2}\right)-U_{f}\left(x_{1},x_{2}\right),$$

since fidelity entropy $S_{f}^{*}\left(x_{1},x_{2}\right)$ remains the same during the shift operation.

This means that fidelity mechanical-state functions depend on a canonical form of the Hamiltonian, which in turn depend on the definition of duality. Therefore, what really matters in fidelity mechanics is not the absolute values of fidelity mechanical-state functions. Instead, the underlying physics is fully captured by fidelity flows, introduced in Section 13. That is, we are only concerned about where stable and unstable fixed points are located in the control parameter space, which are identified as characteristic points and critical points, respectively, in fidelity mechanics. Since a characteristic point appears to be an intersection point between characteristic lines, the constraints imposed by symmetries, dualities, and factorizing fields are *rigid* in the sense that stable fixed points remain the same for any different definitions of a canonical form of the Hamiltonian, resulting from different definitions of duality given that all characteristic lines remain the same as a shift operation in the Hamiltonian is performed. In addition, unstable fixed points also remain the same since the ground- state phase diagram does not change, as a shift operation in the Hamiltonian is performed. Indeed, the behaviors of fidelity mechanical-state functions at stable and unstable fixed points never change: At a stable fixed point, fidelity entropy $S_{f}^{*}\left(x_{1},x_{2}\right)$ reaches its (local) maximum, fidelity temperature $T_{f}^{*}\left(x_{1},x_{2}\right)$ is zero, and fidelity internal energy $U_{f}^{*}\left(x_{1},x_{2}\right)$ takes the maximum value; at an unstable fixed point, fidelity entropy $S_{f}^{*}\left(x_{1},x_{2}\right)$ becomes the residual fidelity entropy, fidelity temperature $T_{f}^{*}\left(x_{1},x_{2}\right)$ diverges, and fidelity internal energy $U_{f}^{*}\left(x_{1},x_{2}\right)$ is zero. This implies that a canonical form of the Hamiltonian, as defined, does not occupy any *unique* position in fidelity mechanics.

In passing, we remark that duality is ubiquitous for quantum many-body systems, as shown in Appendix C, respectively, for the quantum spin-*s* XYZ model and for the spin-1/2 Kitaev model on a honeycomb lattice.

## 4. A Fictitious Parameter σ Connecting Different Choices for a Dominant Control Parameter in a Principal Regime

In a principal regime, there are many different choices of a dominant control parameter *x*, as long as such a choice is consistent with the rigid constraints imposed by symmetries, dualities, and factorizing fields. Different choices result in different fidelity mechanical-state functions. Therefore, two points need to be addressed: first, it is necessary to connect different choices of a dominant control parameter *x* in a given principal regime; second, different choices of a dominant control parameter *x* should not change where stable and unstable fixed points are located.

**Figure 7:**
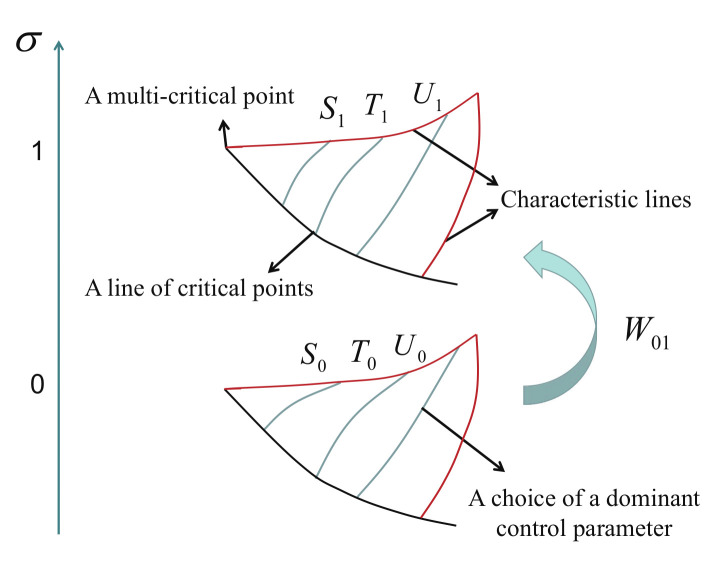
A fictitious parameter σ connecting different choices of a dominant control parameter *x* in a principal regime.

Let us start from the first point. Suppose that we have made two different choices of a dominant control parameter *x* in a given principal regime: One yields fidelity mechanical-state functions $U_{0}$, $S_{0}$, and $T_{0}$, and the other yields $U_{1}$, $S_{1}$, and $T_{1}$. Then, we may introduce a fictitious parameter σ ranging from 0 to 1. Now, it is legitimate to resort to a new set of fidelity mechanical-state functions $U_{\sigma }$, $S_{\sigma }$, and $T_{\sigma }$, which are some smooth functions of σ such that $U_{\sigma }$, $S_{\sigma }$, and $T_{\sigma }$ interpolate between $U_{0}$, $S_{0}$, $T_{0}$, and $U_{1}$, $S_{1}$, $T_{1}$ when σ varies from 0 to 1. This amounts to stating that we may smoothly deform one choice to the other, as depicted in Figure 7. Therefore, we are able to connect one choice to the other by performing a certain amount of fidelity work $W_{01}$:

(39) $$W_{01}=\int \left(dU-T_{\sigma }dS\right)=\Delta U-T_{a}\Delta S,$$

where $\Delta U=U_{1}-U_{0}$, $\Delta S=S_{1}-S_{0}$, and $T_{a}$ may be determined from the mean value theorem for a definite integral. Needless to say, whether fidelity work needs to be performed, $W_{01}$, depends on how we deform our choices into each other. Suppose that $T_{0}<T_{1}$, then we have $T_{0}<T_{a}<T_{1}$. By simply establishing $T_{a}=T_{0}$ or $T_{1}$, we may estimate an upper bound and a lower bound for the amount of fidelity work, quantifying the computational costs, which needs to be performed.

Now, we turn to the second point. Recall that a choice of a dominant control parameter *x* has to be subject to the constraints imposed by symmetries, dualities, and factorizing fields. The constraints are *rigid* in the sense that such a fictitious parameter σ does not exist on any characteristic line. In other words, there is no flexibility in choosing a dominant control parameter *x* on a characteristic line, arising from symmetries, dualities and factorizing fields, apart from a re-parametrization operation. The latter is subject to the condition that for any two choices connected via a re-parametrization operation, one must be a monotonically increasing function of the other and vice versa. However, this does not change where fidelity temperature diverges or becomes zero and does not change where fidelity entropy takes a (local) maximum. Therefore, both stable and unstable fixed points remain the same for any different choices. In this sense, information encoded in a fictitious parameter σ arising from different choices of a dominant control parameter *x* in a principal regime is *irrelevant*.

## 5. Fidelity Mechanical-State Functions under a Shift Operation in the Hamiltonian with Respect to a Reference Benchmark

**Figure 8:**
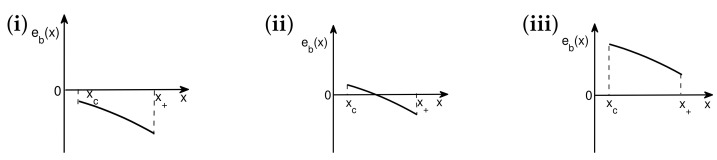
The shifted ground-state energy density $e_{b}\left(x\right)$ as a function of a dominant control parameter *x*, with *x* ranging from $x_{c}$ at a critical point to its value at a point on a characteristic line: $x_{+}$. Here, $e_{b}\left(x\right)=e\left(x\right)+b$, as a result of a shift in the Hamiltonian $H\left(x\right)\to H_{b}\left(x\right)=H\left(x\right)+b$, with e(x) being the ground-state energy density for the Hamiltonian H(x). It is necessary to distinguish three distinct regimes: (**i**) $e_{b}\left(x\right)$ is always negative for any *x*; (**ii**) $e_{b}\left(x\right)$ changes its sign at $x_{r}$: $e_{b}\left(x_{r}\right)=0$; (**iii**) $e_{b}\left(x\right)$ is always positive for any *x*. Here, we assume that the ground-state energy density e(x) monotonically decreases with *x*.

We assume that a dominant control parameter *x* has been chosen. As a convention, *x* ranges from a critical point $x_{c}$ to its value at a point on a characteristic line: $x_{+}$. Suppose that the ground-state energy density e(x) monotonically decreases with *x* and is always negative. Then, fidelity internal energy U(x), fidelity entropy S(x), and fidelity temperature T(x) are well-defined, which simply follow from our prescription in Section 2. Now, we perform a shift in the Hamiltonian *H*: $H\to H_{b}=H+b$, with *b* being a positive constant. Note that we have introduced subscript *b* to replace superscript * in the preceding section, since we move from $\left(x_{1},x_{2}\right)$ to (x,τ), with τ being dropped off for the sake of brevity. Hence, the ground-state energy density e(x) becomes $e_{b}\left(x\right)=e\left(x\right)+b$. As *b* increases, one encounters three distinct regimes for *b*, as shown in Figure 8. First, $e_{b}\left(x\right)$ remains negative for any *x*. Second, $e_{b}\left(x\right)$ is positive for $x<x_{r}$ and negative for $x>x_{r}$, with $x_{r}$ being a unique solution to an algebraic equation $e_{b}\left(x\right)=0$: $e_{b}\left(x_{r}\right)=0$. Third, $e_{b}\left(x\right)$ is always positive for any *x*. As it turns out, the change from one regime to another may be characterized as a “phase transition” in fidelity mechanics. That is, two successive fidelity mechanical phase transitions occur when *b* varies.

For this purpose, let us elaborate on the first regime, when $e_{b}\left(x\right)$ remains negative for any *x*. Obviously, we are allowed to define fidelity mechanical-state functions for any value of *b* in this regime, as follows from the prescription in Section 2. As a result, both fidelity temperature and fidelity internal energy depend on *b*, although fidelity entropy remains the same up to an additive constant, since ground-state wave functions remain the same under the shift. This vividly illustrates the fact that fidelity mechanical-state functions depend *not only* on our choices of a dominant control parameter *x* (cf. Section 4) *but also* on a canonical form of the Hamiltonian, which in turn is related to the definition of duality (cf. Section 3). As argued there, any difference arising from either distinct choices of a dominant control parameter *x* or modified definitions of a canonical form of the Hamiltonian is *irrelevant*, as far as stable and unstable fixed points are concerned. As a consequence, one may choose any value of *b* in this regime to define fidelity mechanical-state functions.

Therefore, a question arises concerning what amount of fidelity work needs to be performed when *b* varies from 0 to a nonzero value, if we adopt fidelity internal energy U(x), fidelity entropy S(x), and fidelity temperature T(x) for b=0 as our reference benchmark. Physically, this means that information about an energy scale at the reference benchmark b=0, recorded in the information storage media, is never updated, as *b* varies, although information about the variation of an energy scale is also recorded. This renders it possible to make a comparison between b≠0 and b=0. When b=0, we have the following

(40) ΔU(x)=T(x)ΔS(x).

Here, we remark that the explicit expressions for fidelity internal energy U(x), fidelity entropy S(x), and fidelity temperature T(x) have been presented in Section 2. For convenience, we reproduce them here: fidelity internal energy $U\left(x\right)=\ln\left(e\left(x\right)/e\left(x_{c}\right)\right)V\left(x\right)+U_{0}$, with $U_{0}$ being an additive constant, and fidelity entropy $S\left(x\right)=-2\int_{x_{c}}^{x}\ln d(x,y)dy+S_{0}$, with $S_{0}$ being an additive constant. Since the ground-state wave functions remain the same under the shift, fidelity entropy, S(x), is left intact: $S_{b}\left(x\right)=S\left(x\right)$. In addition, since a dominant control parameter *x* remains the same and an energy scale at the reference benchmark b=0 is retained, fidelity temperature, T(x), is also left intact: $T_{b}\left(x\right)=T\left(x\right)$. In contrast, fidelity internal energy U(x) undergoes a change: $U\left(x\right)\to U_{b}\left(x\right)$, where $U_{b}\left(x\right)$ takes the form $U_{b}\left(x\right)=\ln\left(e_{b}\left(x\right)/e_{b}\left(x_{c}\right)\right)V\left(x\right)+U_{0}$. Here, we assume that an energy scale $e_{b}\left(x_{c}\right)$ has been adopted for a nonzero value of *b*. Combining with Equation (40), we have the following

(41) $$\Delta U_{b}\left(x\right)=T_{b}\left(x\right)\Delta S_{b}\left(x\right)+\Delta W_{b}\left(x\right).$$

Here, $\Delta W_{b}\left(x\right)$ denotes fidelity work that needs to be performed, with $W_{b}\left(x\right)=\left[\ln\left(e_{b}\left(x\right)/e\left(x\right)\right)-\ln\left(e_{b}\left(x_{c}\right)/e\left(x_{c}\right)\right)\right]V\left(x\right)$.

In order to compare with our reference benchmark b=0, we have to return to the original energy scale $e(x_{c})$ instead of $e_{b}\left(x_{c}\right)$, thus leading to an increment of fidelity entropy: $-\ln(e_{b}\left(x_{c}\right)/e\left(x_{c}\right))\Delta x$. That is, fidelity entropy $S_{b}\left(x\right)$ entails a change due to the fact that different energy scales are exploited when *b* varies: $\left|e(x_{c})\right|$ for b=0 and $|e_{b}\left(x_{c}\right)|$ for nonzero *b*. This variation in energy scales amounts to information erasure (cf. Section 2). As such, fidelity entropy $S_{b}\left(x\right)$ becomes the following:

(42) $$S_{b}\left(x\right)=-2\int_{x_{c}}^{x}\ln d(x,y)dy-\ln\left(e_{b}\left(x_{c}\right)/e\left(x_{c}\right)\right)\Delta x+S_{0},$$

whereas fidelity internal energy $U_{b}\left(x\right)$ becomes the following

(43) $$U_{b}\left(x\right)=\ln\frac{e_{b}\left(x\right)}{e(x_{c})}V\left(x\right)+U_{0}.$$

It follows that fidelity internal energy $U_{b}\left(x\right)$ and, thus, fidelity work $W_{b}\left(x\right)$ diverges if $e_{b}\left(x_{c}\right)\to 0$, as observed in Equation (43). This is anticipated, since this amounts to extracting information about a value of *b* to ensure that $e_{b}\left(x_{c}\right)=0$. More precisely, the amount of fidelity work that needs to be performed depends on the accuracies that we are trying to achieve. If the error scales as $N^{-\zeta }$, the amount of fidelity work needed to be performed scales as lnN. If the error scales as exp(−ηN), the amount of fidelity work needed to be performed scales as *N*. Here, *N* is an integer, and ζ and η are positive real numbers. In fact, this is consistent with the analogue of Landauer’s principle at zero temperature (cf. Section 2), since lnN and *N* bits of information are recorded in the information storage media, when the error scales as $N^{-\mu }$ or exp(−νN), respectively.

Now we turn to the second regime. We expect that fidelity temperature T(x) and fidelity entropy S(x) are left intact, $T_{b}\left(x\right)=T\left(x\right)$ and $S_{b}\left(x\right)=S\left(x\right)$, but fidelity internal energy U(x) undergoes a change: $U\left(x\right)\to U_{b}\left(x\right)$. However, extra complications arise, since $e_{b}\left(x\right)$ changes sign in this regime. A choice for $U_{b}\left(x\right)$, consistent with the analogue of Landauer’s principle at zero temperature (cf. Section 2), takes the following $U_{b}\left(x\right)$$=\mp \ln(|e_{b}\left(x\right)|/|e_{b}\left(x_{c}\right)\left|\right)V\left(x\right)+U_{0}$. Here, the sign − is taken for $x\in (x_{c},x_{r})$, in which $e_{b}\left(x\right)$ is positive, and the sign + is taken for $x\in (x_{r},x_{+})$, in which $e_{b}\left(x\right)$ is negative. As a consequence, $W_{b}\left(x\right)$ takes the following form

(44) $$\begin{array}{cc} W_{b}\left(x\right)= & \left\{\begin{array}{c} \left[-\ln\frac{e_{b}\left(x\right)}{e_{b}\left(x_{c}\right)}-\ln\frac{e(x)}{e(x_{c})}\right]V\left(x\right),\;x\in \left(x_{c},x_{r}\right);\\ \left[\ln\frac{|e_{b}\left(x\right)|}{e_{b}\left(x_{c}\right)}-\ln\frac{e(x)}{e(x_{c})}\right]V\left(x\right),\;x\in \left(x_{r},x_{+}\right). \end{array}\right. \end{array}$$

In order to compare with our reference benchmark b=0, we have to return back to the original energy scale $e(x_{c})$ instead of $e_{b}\left(x_{c}\right)$, thus leading to a change in fidelity entropy S(x): $S\left(x\right)\to S_{b}\left(x\right)$ due to the fact that different energy scales are exploited as *b* varies: $\left|e(x_{c})\right|$ for b=0 and $e_{b}\left(x_{c}\right)$ for nonzero *b*. We have the following

(45) $$\begin{array}{cc} S_{b}\left(x\right)= & \left\{\begin{array}{c} -2\int_{x_{c}}^{x}\ln d(x,y)dy+\ln e_{b}\left(x_{c}\right)+\ln\left|e\left(x_{c}\right)\right|+S_{0},\;x\in \left(x_{c},x_{r}\right);\\ -2\int_{x_{c}}^{x}\ln d(x,y)dy-\ln e_{b}\left(x_{c}\right)+\ln\left|e\left(x_{c}\right)\right|+S_{0},\;x\in \left(x_{r},x_{+}\right). \end{array}\right. \end{array}$$

Accordingly, fidelity internal energy $U_{b}\left(x\right)$ becomes the following

(46) $$\begin{array}{cc} U_{b}\left(x\right)= & \left\{\begin{array}{c} -[\ln e_{b}\left(x\right)+\ln|e\left(x_{c}\right)\left|\right]V\left(x\right)+U_{0},\;x\in \left(x_{c},x_{r}\right);\\ \left[\ln\right|e_{b}\left(x\right)|-\ln|e\left(x_{c}\right)\left|\right]V\left(x\right)+U_{0},\;x\in \left(x_{r},x_{+}\right). \end{array}\right. \end{array}$$

It follows that the amount of fidelity work needed to be performed depends on the accuracies that we are trying to achieve in locating a solution $x_{r}$ to an algebraic equation e(x)+b=0. More precisely, if the error scales as $N^{-\zeta }$, the amount of fidelity work needed to be performed scales as lnN. If the error scales as exp(−ηN), the amount of fidelity work needed to be performed scales as *N*.

The third regime is similar to the first regime, with the difference being that $e_{b}\left(x\right)$ remains positive for any *x*. Fidelity temperature T(x) and fidelity entropy S(x) are left intact: $T_{b}\left(x\right)=T\left(x\right)$ and $S_{b}\left(x\right)=S\left(x\right)$. However, fidelity internal energy U(x) undergoes a change: $U\left(x\right)\to U_{b}\left(x\right)$. Following the argument in Appendix F, fidelity internal energy $U_{b}\left(x\right)$ takes the following form: $U_{b}\left(x\right)=-\ln\left(e_{b}\left(x\right)/e_{b}\left(x_{c}\right)\right)V\left(x\right)+U_{0}$. As a consequence, we have $W_{b}\left(x\right)=\left[-\ln\left(e_{b}\left(x\right)/e_{b}\left(x_{c}\right)\right)-\ln\left(e\left(x\right)/e\left(x_{c}\right)\right)\right]V\left(x\right)$.

In order to compare with our reference benchmark b=0, we have to return to the original energy scale $e(x_{c})$ instead of $e_{b}\left(x_{c}\right)$, thus leading to a change in fidelity entropy S(x): $S\left(x\right)\to S_{b}\left(x\right)$ due to the fact that different energy scales are exploited when *b* varies: $\left|e(x_{c})\right|$ for b=0 and $e_{b}\left(x_{c}\right)$ for nonzero *b*. That is, fidelity entropy $S_{b}\left(x\right)$ becomes the following

(47) $$S_{b}\left(x\right)=-2\int_{x_{c}}^{x}\ln d(x,y)dy+\ln e_{b}\left(x_{c}\right)+\ln\left|e\left(x_{c}\right)\right|+S_{0}.$$

Accordingly, fidelity internal energy $U_{b}\left(x\right)$ becomes the following

(48) $$U_{b}\left(x\right)=-[\ln e_{b}\left(x\right)+\ln|e\left(x_{c}\right)\left|\right]V\left(x\right)+U_{0}.$$

The same argument also works if the ground-state energy density e(x) monotonically increases with *x* and is always negative.

We remark that our discussion about the amount of fidelity work that needs to be performed to achieve a preset accuracy leads to the supposition that fidelity work might be exploited to quantify computational costs in solving a mathematical problem.

Now, we are ready to justify our assumption about the ground-state energy density e(x) that it is negative for all *x*, which was discussed in Section 2. For a given Hamiltonian H(x), if e(x) is not always negative, then it should be shifted to ensure that it is negative. Hence, fidelity entropy, fidelity temperature, and fidelity internal energy can be determined following from our formalism in Section 2. As argued in Section 3, we are able to assign the shifted Hamiltonian to be a canonical form of the Hamiltonian according to a specific definition of duality. In order to shift it back to the original Hamiltonian, we resort to our discussions above in case (ii) or case (iii): if e(x) changes its sign, then it is case (ii); if e(x) is positive for all *x*, then it is case (iii). This allows the determination of fidelity internal energy, with fidelity entropy being subject to a change due to different energy scales as *b* varies, and with fidelity temperature being left intact. Therefore, our assumption that e(x) is negative for all *x*, made in Section 2, does not prevent us from investigating any quantum many-body system in fidelity mechanics.

## 6. A Characterization of Quantum Phase Transitions and Quantum States of Matter in Fidelity Mechanics

As already mentioned in Section 6, when we piece together all regimes through continuity requirements to visualize fidelity mechanical-state functions in the entire control parameter space, a question arises regarding whether or not fidelity mechanical-state functions are single-valued, which is relevant to the characterization of QPTs and quantum states of matter in fidelity mechanics. In practice, fidelity mechanical-state functions are first determined for a principal part on a characteristic line, and then an extension is carried out to a principal regime, which accommodates a line of critical points as a boundary. Recall that a principal regime always accommodates an isolated critical point, a line of discontinuous QPT points ending at an isolated critical point or a line of critical points, which acts as a boundary for a given principal regime. However, for our purpose, we only need to focus on a principal regime involving a line of critical points, given that fidelity mechanical-state functions are always single-valued, if a principal regime *only* involves an isolated critical point or a line of discontinuous QPT points ending at an isolated critical point, as follows from our prescriptions for a characteristic line and for a principal regime in Section 2.

**Figure 9:**
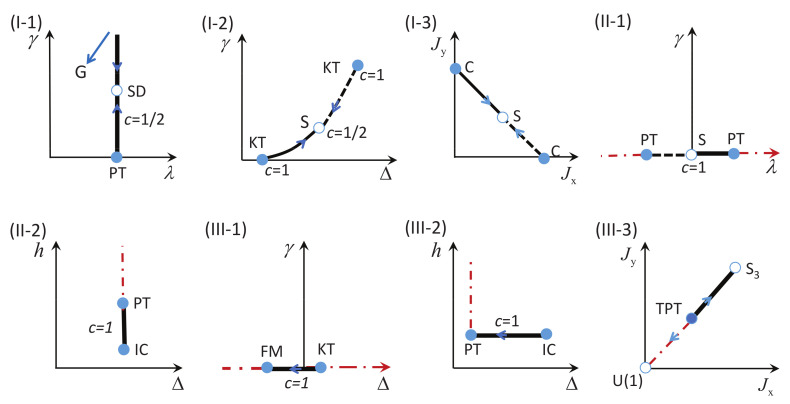
For all six illustrative models, there are eight scenarios for a complete line of critical points that admit an interior point of view and an exterior point of view simultaneously, which in turn fall into the three categories. They are labelled as **I-1**, **I-2**, **I-3**, **II-1**, **II-2**, **III-1**, **III-2**, and **III-3**. Here, $J_{z}$ is set to be an energy scale: $J_{z}=1$ in scenario I-3 and scenario III-3, and *c* denotes central charge. A complete line of critical points or a principal part on a complete line of critical points is depicted as a solid line; a critical/transition point, located at an endpoint, is depicted as a solid dot; a characteristic point is depicted as an empty dot; a symmetric or dual image part on a complete line is depicted as a dash line. In particular, a dash-dot line emanating from an endpoint on a complete line was used to indicate an analogue of the Hawking radiation, and an arrow on a complete line indicates a possible choice of a dominant control parameter *x*. In addition,“SD”, “PT”, “KT”, “S”, “C”, “IC”, “FM”, “TPT”, “U(1)”, and “$S_{3}$” represent a self-dual point, a PT transition point, a KT transition point, a symmetric point, a phase transition point from a gapped $Z_{2}$ spin liquid phase to a gapped $Z_{2}$ spin liquid phase, an IC transition point, an FM transition point, a TPT point, a U(1)-symmetric point, and an $S_{3}$-symmetric point, respectively. We remark that, for scenario I-1, there is a critical point with central charge c=1, labelled as “G”, which is located at infinity, as indicated by an arrow. Here, PT, KT, IC, FM and TPT are the shorthands for Pokrovsky–Talapov, Kosterlitz–Thouless, intermediate case, ferromagnetic and topological phase transition, respectively.

Among the nineteen principal regimes, there are fourteen principal regimes that involve a line of critical points. We remark that, generically, it is necessary to include their image regimes under a symmetric or duality transformation to form a full ground-state phase diagram. In fact, a typical scenario for a complete line of critical points emerges when one principal regime is adjacent to another principal regime or a non-principal regime, which is either symmetrical or dual in nature relative to the principal regime. As it turns out, there are eight distinct scenarios for a complete line of critical points for quantum many-body systems under investigation, which fall into the three categories, as listed in Figure 9. Here, by a complete line of critical points, we mean a line of critical points, which ends at two transition points or at two characteristic points or at one transition point and one characteristic point. Note that it is straightforward to extend this notion to a high-dimensional object, if quantum many-body systems with more than two control parameters are considered.

**Figure 10:**
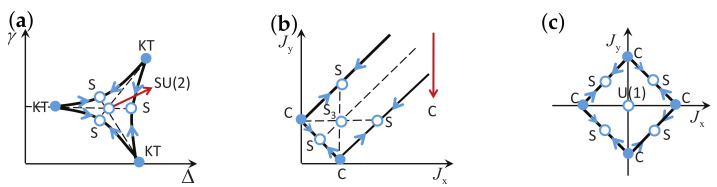
Three types of cycles are formed from a few complete lines of critical points, some of which are symmetrical or dual relative to each other. (**a**) A cycle is formed from the three complete lines of critical points in scenario I-2, which are dual relative to each other. Here, a KT transition point, with central charge being c=1, is located at each of the three intersection points between two of the three complete lines of critical points. Note that central charge *c* is equal to 1/2 at an interior point on the cycle away from the three KT transition point, and a U(1)-symmetric point is located at the middle on each of the three complete lines of critical points. (**b**) A cycle is formed from the three complete lines of critical points in scenario I-3, which are symmetrical or dual relative to each other. (**c**) A cycle is formed from the two complete lines of critical points in scenario I-3, which are symmetrical relative to each other under a symmetric transformation: $J_{x}↔-J_{x}$ and $J_{y}↔-J_{y}$, together with their counterparts when $J_{x}$ becomes $-J_{x}$, with $J_{y}$ left intact. Here, a complete line of critical points is depicted as a solid line; a critical point located at the endpoints of a critical line is depicted as a solid dot; a characteristic point is depicted as an empty dot; a characteristic line is depicted as a dash line, with an arrow indicating a choice of a dominant control parameter *x* on a principal part and its dual images. In addition, “KT”, “S”, “C”, “SU(2)”, “$S_{3}$”, and “U(1)” represent a KT transition point, a symmetric point, a phase transition point from a gapped $Z_{2}$ spin liquid phase to a gapped $Z_{2}$ spin liquid phase, an SU(2)-symmetric point, an $S_{3}$-symmetric point, and a U(1)-symmetric point, respectively. In addition, there is a phase transition from a gapless $Z_{2}$ spin liquid phase to a gapped $Z_{2}$ spin liquid phase at an interior point on a complete line of critical points. For the spin-1/2 Kitaev model on a honeycomb lattice, we set $J_{z}$ as an energy scale: $J_{z}=1$. Then, a critical point, dual in nature relative to two transition points labelled as *C*, is located at infinity, when $J_{x}$ and $J_{y}$, which are proportional to each other, are infinite in value, as indicated in terms of an arrow.

Our task is to characterize distinct types of QPTs by conducting an investigation into the behaviors of fidelity mechanical-state functions at QPT points, which act as the endpoints of a complete line of critical points. This leads us to an interior point of view vs. an exterior point of view, introduced in the next subsection. In addition, a cycle, formed from a complete line of critical points with its symmetric or dual images, also plays an important role in characterizing quantum states of matter. To this end, one needs to focus on the behaviors of fidelity entropy on a characteristic line or at a characteristic point, located inside a cycle. For the six illustrative models, three cycles emerge, as shown in Figure 10: one for the quantum spin-1 XYZ model and the other two for the spin-1/2 Kitaev model on a honeycomb lattice.

### 6.1. An Interior Point of View vs. an Exterior Point of View

The similarity between a critical point and a black hole arises from the observation that both are relevant to singularities: Critical points result from a singularity in the control parameter space, whereas black holes result from a singularity in space-time. This is even valid for an isolated critical point, which acts as an endpoint on a line of discontinuous QPT points. However, a complete line of critical points appears to be more relevant in this regard. In fact, one may bring this analogy one step further.

As is well-known, it is impossible to communicate between two observers, with one located inside a black hole and the other located outside a black hole, except for the so-called Hawking radiation [98,99]. Although a complete line of critical points is itself not necessarily a characteristic line, our prescription for a principal part on a characteristic line still applies. If a characteristic line consists of two parts that are symmetrical or dual to one another, then we only need to work out our prescription for a principal part, with fidelity mechanical-state functions on the other part—a symmetric or dual image part—simply deduced from a symmetric or duality transformation. As a consequence, we encounter two sets of fidelity mechanical-state functions on a complete line of critical points: One set arises when our prescription for a characteristic line is implemented for a principal part on a complete line of critical points, and the other set arises when our prescription for a principal regime is implemented for a principal regime, with a principal part on a complete line of critical points as its boundary. In other words, the subtleties concerning the single valuedness of fidelity mechanical-state functions originate from two distinct viewpoints: an interior point of view vs. an exterior point of view. This is analogous to a black hole, with the event horizon separating the interior and the exterior regions of a black hole.

It is remarkable to observe that, in some cases, one may turn the two distinct viewpoints into two distinct perspectives, both of which only concern an exterior point of view. This is due to the fact that, if a complete line of critical points itself is located on, but it is *only* a part of, a symmetric line, then a QPT occurs at an endpoint. Such a QPT point is protected by the symmetry group on this symmetric line, in addition to a possible discrete symmetry group. That amounts to stating that one is able to speak of an interior point of view vs. an exterior point of view with respect to the symmetric line itself: the interior and the exterior are separated by such a QPT point, which acts as an endpoint on the complete line of critical points. In certain circumstances, fidelity mechanical-state functions from an interior point of view match that from an exterior point of view at such a QPT point, as a result of the continuity requirements for fidelity mechanical-state functions, if one is restricted to the symmetric line. In other words, it is possible to access information encoded in the interior of a complete line of critical points from the exterior if one is restricted to the symmetric line itself. This is more or less analogous to the Hawking radiation [98,99].

Therefore, we have to deal with two distinct perspectives from an exterior point of view on a symmetric line, if an analogue of the Hawking radiation occurs on a symmetric line, which in turn accommodates a complete line of critical points, with its extent being finite. In fact, this happens for the five scenarios in the second and third categories, and it is indicated in terms of a dash-dot line emanating from an endpoint, representing a QPT point, on a complete line in Figure 9. For the six illustrative models, a QPT point involved is a PT transition point, an FM transition point, a KT transition point, an IC transition point, and a TPT transition point. Note that a PT transition point, an FM transition point, a KT transition point, and an IC transition point separate a line of critical points from a gapped part on a U(1)-symmetric line for scenario II-1, scenario II-2, scenario III-1, and scenario III-2, and a TPT transition point separates a line of critical points from a gapped part on the $Z_{2}$-symmetric line for scenario III-3, respectively. However, the two perspectives do not necessarily match with each other, as detailed in the next subsection. As it turns out, this offers a novel characterization of QPT points in fidelity mechanics.

Here, it is proper to stress that it only makes sense to speak of an interior point of view vs. an exterior point of view with respect to an isolated critical point, a complete line of critical points or a two-dimensional critical regime, since both are *relative*. For example, an interior point of view with respect to a two-dimensional critical regime is simultaneously an exterior point of view with respect to a complete line of critical points located on its boundary, which forms a cycle, together with its symmetric or dual images.

### 6.2. A Characterization of Quantum Phase Transitions in Fidelity Mechanics

In this subsection, we elaborate on the behaviors of fidelity mechanical-state functions on a complete line of critical points.

To begin with, let us make it clear that fidelity mechanical-state functions from an interior point of view at any point away from the endpoints on a complete line of critical points always do not match those from an exterior point of view, as follows from our prescriptions for a characteristic line and for a principal regime in Section 2. Therefore, we only need to focus on the endpoints on a complete line of critical points. Here, we remark that fidelity mechanical-state functions from an exterior point of view at the endpoints are defined as a limit of those from our prescription for a principal regime, when the endpoints are approached along a complete line of critical points, and fidelity mechanical-state functions from an interior point of view at the endpoints follow from our prescription for a principal part on a characteristic line.

The first category contains three scenarios, as shown in Figure 9. Here, a complete line of critical points, labelled as I-1, ends at one PT transition point and at one critical point with central charge c=1 at infinity. This scenario emerges, when a principal regime, labelled as II, and a principal regime, labelled as III, are adjacent to each other, or a principal regime, labelled as IV, and a principal regime, labelled as V, are adjacent to each other for the quantum spin-1/2 XY model (cf. Section 2). Here, central charge *c* is equal to 1/2 on the complete line of critical points away from the two endpoints, which are labelled as “PT” and “G”, respectively, although it only makes sense to speak of a PT transition when it is approached along the U(1)-symmetric line (γ=0). A complete line of critical points, labelled as I-2, ends at two KT transition points, with the U(1)-symmetric point in the middle. This scenario emerges when a principal regime, labelled as I, is adjacent to its dual image, or a principal regime, labelled as III, is adjacent to its dual image (cf. Section 2). It appears as the line of the Ising critical points for the quantum spin-1 XYZ model (5). Here, central charge *c* is equal to 1/2 at an interior point on a complete line of critical points, and central charge *c* is equal to 1 at the endpoints, labelled as “KT”, although it only makes sense to speak of a KT transition when it is approached along the U(1)-symmetric line: γ=0 and its dual images. A complete line of critical points, labelled as I-3, ends at two transition points, with a $Z_{2}$-symmetric point in the middle. This scenario emerges, when a principal regime, labelled as I, is adjacent to its symmetric image, or a principal regime, labelled as II, is adjacent to its symmetric image (cf. Section 2). It appears as the line of the TPT transition points for the spin-1/2 Kitaev model on a honeycomb lattice (6). The transition points, labelled as C, describe a QPT from a gapped $Z_{2}$ spin liquid to a gapped $Z_{2}$ spin liquid in contrast to a TPT transition point at an interior point on the complete line of critical points, which describes a QPT from a gapped $Z_{2}$ spin liquid to a gapless $Z_{2}$ spin liquid.

A commonality among the three scenarios is that a complete line of critical points, together with a symmetric or dual image, either extends to a characteristic point located at infinity, as it happens in scenario I-1, or forms a cycle, as it happens in scenario I-2 and scenario I-3. As a consequence, our prescriptions imply that, according to our convention, fidelity internal energy is zero at the endpoints from an exterior point of view and from an interior point of view. Therefore, fidelity internal energy is single-valued at the endpoints. Fidelity temperatures diverge at endpoints from an exterior point of view and from an interior point of view, although fidelity temperature from an interior point of view is finite at any point away from the endpoints on a complete line and even become zero at a self-dual or a symmetric point, labelled as “SD” or “S” in Figure 9. Although fidelity entropy from an interior point of view does not match that from an exterior point of view, at any point away from the endpoints on a complete line of critical points, it is always possible to adjust an additive constant at a self-dual or a symmetric point, labelled as “SD” or “S”, to ensure that fidelity entropy from an interior point of view matches that from an exterior point of view at the endpoints. That is, fidelity entropy is single-valued at the endpoints, labelled as “PT”, “KT”, and “C”, and it diverges at the endpoint, labelled as “G”, located at infinity. That is, it becomes minus infinity due to the presence of scaling entropy.

The second category contains two scenarios, as shown in Figure 9. A complete line of critical points, labelled as II-1, ends at two PT transition points, with a $Z_{2}$-symmetric point in the middle. This scenario emerges when a principal regime, labelled as I, is adjacent to its symmetric image (cf. Section 2). It appears as the line of the Gaussian critical points for the quantum spin-1/2 XY model (1). Here, the transition points, labelled as “PT”, represent PT transitions protected by the symmetry group U(1). A complete line of critical points, labelled as II-2, ends at one PT transition point and one IC transition point. This scenario emerges as a vertical line, with a fixed value of Δ, in the two-dimensional critical XY regime—a principal regime—labelled as III for the quantum spin-1/2 XXZ model in a magnetic field (cf. Section 2). We remark that this principal regime involves a line of the PT transition points and a line of the IC transition points, which meet each other at infinity, with the two-dimensional critical XY regime in between.

A commonality among the two scenarios is that a complete line of critical points itself results from the level crossings due to the fact that the model Hamiltonian, i.e., Equation (1) when γ=0 or Equation (4), is split into two commuting parts, since $\sum \sigma_{i}^{z}$ is conserved. As a consequence, the ground-state fidelity per lattice site vanishes; therefore, no dominant control parameter *x* is, by definition, available. Hence, it does not make sense to speak of fidelity mechanical-state functions from an interior point of view for scenario II-1 and scenario II-2. However, this does not mean that it is impossible to determine fidelity mechanical-state functions for a critical point located on such a complete line of critical points. Instead, we have to embed it into the two-dimensional critical XY regime and choose a dominant control parameter *x* to avoid the level crossings such that the ground-state fidelity per lattice site does not vanish. This leads us to scenario III-1 and scenario III-2 in the third category. Indeed, the difference between the two scenarios in the second category and the two scenarios, i.e., scenario III-1 and scenario III-2, in the third category lies in the fact that the model Hamiltonian at one point commutes with the model Hamiltonian at another point on a complete line in scenario II-1 and scenario II-2, with their difference being proportional to $\sum \sigma_{i}^{z}$, but that is not true for scenario III-1 and scenario III-2, although all four complete lines lie in the two-dimensional critical XY regime, with the symmetry group being U(1). In other words, it is necessary to combine scenario II-1 and scenario II-2 with scenario III-2 to determine fidelity mechanical-state functions at the endpoints. For this purpose, it is convenient to make a distinction between two types of U(1)-symmetric lines. We refer to a U(1)-symmetric line in scenario II-1 and scenario II-2 as a commuting U(1)-symmetric line in order to distinguish from a non-commuting U(1)-symmetric line in scenario III-1 and scenario III-2.

A complete line of critical points in the two scenarios is located on a symmetric line—the commuting U(1)-symmetric line. Here, we stress that, for a critical point on a complete line in scenario II-1 and scenario II-2, an interior point of view comes from scenario III-2, as already argued above. That is, two perspectives from an exterior point of view are involved for scenario II-1 and scenario II-2, combined with scenario III-2: The first perspective is restricted to the commuting U(1)-symmetric line, and the second perspective requires an introduction of the quantum spin-1/2 XYZ model in an external magnetic field such that the quantum spin-1/2 XXZ model in an external magnetic field itself becomes a two-dimensional U(1) characteristic plane, into which both a commuting U(1)-symmetric line and a non-commuting U(1)-symmetric line are embedded. We stress that a commuting U(1)-symmetric line may be regarded as a characteristic line on this two-dimensional U(1) characteristic plane due to the commutativity mentioned above in contrast to a non-commuting U(1)-symmetric line, which is generically not a characteristic line, if one is restricted to this two-dimensional U(1) characteristic plane, unless it involves an additional discrete symmetry group. Indeed, it occurs on the non-commuting U(1)-symmetric line in scenario III-1—the dihedral symmetry group $Z_{2}\times Z_{2}$, generated from any two of the three π-rotations around the *x*-, *y*- and *z*-axes, and the symmetry group $Z_{2}$, generated from the time-reversal symmetry operation. However, such a discrete symmetry group does not occur for the non-commuting U(1)-symmetric line in scenario III-2.

We remark that an exterior point of view with respect to a complete line of critical points in scenario II-1 and scenario II-2 is part of an exterior point of view with respect to the two-dimensional critical XY regime for the quantum spin-1/2 XXZ model in a magnetic field (4). Recall that the quantum spin-1/2 XYZ model in a magnetic field accommodates a factorizing-field cone surface [77,78,79,80,81,82], which divides a given phase into three-dimensional principal regimes if symmetries and dualities are taken into account. That is, we have to cope with a three-dimensional principal regime, with one dominant control parameter *x* and two auxiliary control parameters $\tau_{1}$ and $\tau_{2}$.

We turn to fidelity mechanical-state functions at an endpoint, labelled as “PT”, for scenario II-1 and scenario II-2 and at an endpoint labelled as “IC” for scenario II-2. For an endpoint, labelled as “PT”, fidelity internal energy from the first perspective takes the maximum value, but it is zero from the second perspective according to our convention. Fidelity temperature is zero from the first perspective, but it diverges from the second perspective. Meanwhile, fidelity entropy from the first perspective may be adjusted to be identical to that of the second perspective, since the commuting U(1)-symmetric line ends at a characteristic point located at infinity. Indeed, fidelity entropy diverges due to the presence of scaling entropy at the characteristic point. For the endpoint labelled as “IC”, fidelity internal energy is zero, according to our convention, whereas fidelity temperature diverges from an exterior point of view. Meanwhile, fidelity entropy at an IC transition point may be determined from that at a PT transition point from an exterior point of view. However, no interior point of view is available for scenario II-2, as already argued above. Instead, a combination with scenario III-2 is necessary to capture the full picture for fidelity mechanical-state functions at an IC transition point, as is performed below. This will also clarify why no analogue of the Hawking radiation occurs at an IC transition point.

The third category contains three scenarios, as shown in Figure 9. A complete line of critical points, labelled as III-1, ends at one FM transition point and one KT transition point (cf. Section 2). This scenario emerges when a principal regime, labelled as I, is adjacent to a principal regime, labelled as II, for the quantum spin-1/2 XYZ model (3), or it emerges when a principal regime labelled as II is adjacent to a principal regime, labelled as IV, for the quantum spin-1 XYZ model (5). It appears as a line of the Gaussian critical points. Note that the complete line acts as one principal part, since no characteristic point exists between the KT transition point and the FM transition point. Here, a subtlety arises as to whether or not we should regard the point (0,0) as a characteristic point, since it is located on the semi-self-dual line (Δ=0) intersecting with the U(1)-symmetric line (γ=0). However, we stress that the duality transformation involved maps the complete line of critical points to a critical point located at infinity. In other words, the point (0,0) is featureless on the complete line. That is, the point (0,0) is not a characteristic point from an interior point of view, although it is a characteristic point from an exterior point of view. Here, we recall that a characteristic point is defined to be an intersection point between two characteristic lines. Actually, there are two types of characteristic points: one type is located on a line of critical points, and the other type is away from a line of critical points. This is due to the fact that a line of critical points may be located on a symmetric or dual line. Occasionally, subtlety arises for the first type of characteristic points, since it only makes sense to recognize them from an exterior point of view. That is, it may happen that such a characteristic point from an exterior point of view is not a characteristic point anymore from an interior point of view. In addition, for the quantum spin-1 XYZ model, we are unable to locate the KT transition point exactly. If it is located at the origin, then the complete line constitutes a boundary in a principal regime, labelled as II, since a principal regime, labelled as IV, vanishes. A complete line of critical points, labelled as III-2, ends at one PT transition point and one IC transition point. This scenario emerges as a horizontal line, with a value of *h* being fixed, in a principal regime, labelled as III, for the quantum spin-1/2 XXZ model in a magnetic field (4) (cf. Section 2). It appears as a horizontal line of the Gaussian critical points. A complete line of critical points, labelled as III-3, ends at one TPT transition point and one $S_{3}$-symmetric point. This scenario emerges, as a boundary in a principal regime, labelled as II, for the spin-1/2 Kitaev model on a honeycomb lattice (6) (cf. Section 2). It appears as a typical line representing the TPT transition points between the gapped and gapless spin liquids. In order to evaluate fidelity mechanical-state functions from an exterior point of view with respect to the complete line of critical points in scenario III-3, it is necessary to extend to the two-dimensional critical regime, representing a gapless $Z_{2}$ spin liquid—a principal regime, labelled as II, into which the complete line of critical points is embedded as a boundary. The situation is similar to scenario II-2 and scenario III-2. That is, we have to cope with a three-dimensional principal regime, with one dominant control parameter *x* and two auxiliary control parameters $\tau_{1}$ and $\tau_{2}$. In other words, we have to introduce an extra coupling parameter. One way to perform this is to consider the spin-1/2 Kitaev model on a honeycomb lattice coupled to an external magnetic field along the [111]-axis [100,101], with this principal regime located on a $Z_{2}$-symmetric plane. The advantage to consider this coupling parameter is that the field couples the spins in a symmetric way, in a sense that the field does not prefer any particular bond labelled as the *x*-bonds, *y*-bonds, and *z*-bonds. That is, the $S_{3}$-symmetric point is extended to an $S_{3}$-symmetric line. A numerical simulation has been performed for this $S_{3}$-symmetric line in terms of the infinite density matrix renormalization group (iDMRG) algorithm [101], which reveals two successive QPTs at finite field strengths, depending on the sign of the Kitaev exchange coupling parameters: the (non-Abelian) gapless $Z_{2}$ spin liquid at low fields, an intermediate regime only present for the AF Kitaev exchange, and a field-polarized phase at large fields.

A commonality among the three scenarios is that a complete line of critical points is located on a symmetric line: the non-commuting U(1)-symmetric line for scenario III-1 and scenario III-2 and the $Z_{2}$-symmetric line for scenario III-3, with a possible additional discrete symmetry group; the dihedral symmetry group $Z_{2}\times Z_{2}$ and the symmetry group $Z_{2}$ for scenario III-1 and scenario III-3, but none for scenario III-2, respectively.

We are now ready to describe the behaviors of fidelity mechanical-state functions at a QPT point as follows.

At the PT transition point, the situation is a bit cumbersome since it involves either scenario II-1 and scenario III-2 or scenario II-2 and scenario III-2 simultaneously. Fidelity internal energy is the maximum and fidelity temperature is zero from the first perspective, according to scenario II-1 or scenario II-2, and fidelity internal energy is zero and fidelity temperature diverges from the second perspective, according to scenario III-2. Meanwhile, fidelity entropy from the first perspective, according to scenario II-1 or scenario II-2, may be adjusted to be identical to that from the second perspective, according to scenario III-2. Hence, fidelity entropy is single-valued.

At the FM transition point, fidelity internal energy from the first perspective is at the maximum, but it is zero from the second perspective. Fidelity temperature is zero from the first perspective, but it diverges from the second perspective. In contrast, fidelity entropy from the first perspective may be adjusted to be identical to that from the second perspective, since a characteristic point is located at infinity, Δ=−∞, on the non-commuting U(1)-symmetric line. Indeed, fidelity entropy diverges at the characteristic point due to the presence of scaling entropy. Hence, fidelity entropy is single-valued at the FM transition point. Physically, this is consistent with the fact that an FM transition point accommodates highly degenerate and highly entangled ground states arising from SSB with one type-B GM, which are scale-invariant [102] (cf. Appendix I).

At the KT transition point, fidelity internal energy is zero and fidelity temperature diverges from both the first perspective and the second perspective. However, it is impossible to adjust fidelity entropy from the first perspective to be identical to that from the second perspective. Therefore, fidelity entropy is double-valued at the KT transition point.

At the IC transition point, fidelity internal energy is zero and fidelity temperature diverges from both an exterior point of view and an interior point of view, according to scenario III-2. However, it is impossible to adjust fidelity entropy from an interior point of view to be identical to that from an exterior point of view, and so fidelity entropy is double-valued at the IC transition point, if it is approached from inside the two-dimensional critical XY regime. In addition, fidelity mechanical-state functions remain the same as those for the quantum spin-1/2 XXZ model (3), with the same value of Δ, if an IC transition point is approached along a vertical line inside the AF phase—the commuting U(1)-symmetric line. As a consequence, fidelity entropy is triple-valued at an IC transition point. We remark that fidelity temperature is nonzero at an IC transition point, if it is approached along a vertical line inside the AF phase.

At the TPT transition point, fidelity internal energy is zero and fidelity temperature diverges from both the first perspective and the second perspective. In contrast, fidelity entropy is double-valued at the transition point, which is labelled as TPT. That is, fidelity entropy from the first perspective does not match that from the second perspective at the TPT transition point. In addition, fidelity internal energy is the maximum and fidelity temperature is zero at the $S_{3}$-symmetric point, labelled as $S_{3}$.

We are led to a remarkable observation that an analogue of the Hawking radiation occurs if and only if fidelity temperature, which is zero or diverges from an interior point of view at a QPT point, matches that from an exterior point of view when it is approached along a symmetric line. Here, a QPT point acts as an endpoint on a complete line of critical points, and an extra distinct feature is necessary if a symmetric line itself lies in a symmetric plane, as exemplified in the non-commuting U(1)-symmetric line for scenario III-1. Physically, this originates from the fact that fidelity temperature is zero at a stable fixed point and diverges at an unstable fixed point. This observation may be regarded as a criterion for the occurrence of an analogue of the Hawking radiation at a QPT point.

According to our criterion, an analogue of the Hawking radiation occurs at the endpoints labelled as “FM” and “KT” for scenario III-1, at the endpoint, labelled as “PT” for scenario III-2, and at the endpoint, labelled as “TPT” for scenario III-3 in the sense that it is possible to access information encoded in the interior of a complete line of critical points from the exterior, if one is restricted to a symmetric line itself. However, no analogue of the Hawking radiation occurs at the endpoint, labelled as “IC” in scenario III-2, since fidelity temperature is nonzero but finite when it is approached along the commuting U(1)-symmetric line in contrast to other cases. Here, we stress that an analogue of the Hawking radiation at the endpoint labelled as “PT” for scenario III-2 is restricted to the vertical line, with the value of Δ being fixed. Indeed, this is nothing but an analogue of the Hawking radiation in scenario II-2.

Now, we are in a position to characterize distinct types of QPTs in fidelity mechanics. Generically speaking, if one is not able to discard the double-valuedness of fidelity entropy at a QPT point, then it must be characterized as *topological*. As a consequence, the KT transitions and TPT transitions are topological. In contrast, the PT transitions and FM transitions are not topological. In particular, the IC transitions are peculiar in the sense that fidelity entropy is double-valued, as follows from scenario III-2, if it is approached along a horizontal line inside the two-dimensional critical XY regime. However, fidelity entropy is single-valued, as follows from scenario II-2, if it is approached along a vertical line inside the AF phase. This reflects the fact that an IC transition interpolates between the KT transitions and PT transitions.

In addition, there are peculiarities in the characterization of the PT transitions, FM transitions, and IC transitions in fidelity mechanics. For the PT transitions and FM transitions, fidelity internal energy ranges from zero to the maximum and fidelity temperature ranges from zero to infinity. However, there is a marked difference between a PT transition point and an FM transition point: The ground state is nondegenerate for the former, whereas ground states are highly degenerate for the latter. For the IC transitions, fidelity internal energy ranges from zero to a finite value, which is less than the maximum, and fidelity temperature ranges from a nonzero value to infinity.

Actually, our argument unveils an important role of the dihedral symmetry group $Z_{2}\times Z_{2}$ and the time-reversal symmetry group $Z_{2}$ in characterizing the FM transitions and the KT transitions in fidelity mechanics. In fact, both the FM transitions and the KT transitions are protected by the dihedral symmetry group $Z_{2}\times Z_{2}$ and the time-reversal symmetry group $Z_{2}$ in addition to the symmetry group U(1) on the non-commuting U(1)-symmetric line. Once a term, such as $\sum \sigma_{i}^{z}$, is introduced into the model Hamiltonian, both symmetry groups are explicitly broken. Hence, the FM transitions and KT transitions are turned into the PT transitions and IC transitions, respectively. In this sense, one may regard the PT transitions and IC transitions as variants of the FM transitions and KT transitions when the dihedral symmetry and the time-reversal symmetry are explicitly broken, respectively. In other words, the PT transitions are to the FM transitions as the IC transitions are to the KT transitions. The same reasoning also applies to the TPT transitions. In fact, the presence of an external magnetic field along the [111]-axis explicitly breaks the dihedral symmetry group $Z_{2}\times Z_{2}$ and the time-reversal symmetry group $Z_{2}$, in contrast to the Hamiltonian for the spin-1/2 Kitaev model on a honeycomb lattice (6). As a result, the first of the two successive QPTs, unveiled in [101], is the variant of the TPT transitions, when the dihedral symmetry group $Z_{2}\times Z_{2}$ and the time-reversal symmetry group $Z_{2}$ are explicitly broken.

This fact is echoed in conventional characterizations. Indeed, the FM transitions arise from SSB, with one type-B GM, when SU(2) is spontaneously broken to U(1). Hence, the ground-state subspace accommodates both the highest weight state and the lowest weight state, which are symmetrical with respect to the time-reversal symmetry operation. That is, the time-reversal symmetry group $Z_{2}$ is broken spontaneously at the FM transition point. Therefore, if the time-reversal symmetry group $Z_{2}$ is explicitly broken, then either the highest weight state or the lowest weight state is selected as a ground state depending on the sign of the longitudinal field strength *h*. Since both the highest and the lowest weight states are unentangled factorized ground states, they may be characterized as a trivial scale-invariant state. Actually, the entanglement entropy is simply zero, which is consistent with the fact that the number of the type-A GMs is zero according to the Mermin–Wagner–Coleman theorem [103,104], and the number of the type-B GMs is zero, as follows from the counting rule [105,106]. This is due to the fact that at least two broken generators are needed for one type-B GM according to the counting rule. Hence, the number of type-B GMs must be zero if the symmetry group is U(1) (cf. Appendix I for more details). In addition, there is an *emergent* symmetry group $Z_{2}$ generated by the time-reversal symmetry operation in the AF phase for the quantum spin-1/2 XXZ model in a magnetic field (4) if the longitudinal field strength *h* is nonzero. This means that the time-reversal symmetry is recovered in the two-fold degenerate ground-state subspace, although the model Hamiltonian is not invariant under the time-reversal symmetry operation. The same argument also applies for the dihedral symmetry group $Z_{2}\times Z_{2}$. As a consequence, the entire AF phase is controlled by the KT transition point, instead of a line of the IC transition points. This explains why no analogue of the Hawking radiation arises at an IC transition point. If an IC transition point is approached along a vertical line inside the AF phase, then the correlation length remains finite or, equivalently, the entanglement entropy is finite, which is consistent with the observation that fidelity temperature is finite in our characterization.

Therefore, a smoking-gun signature is unveiled for distinct types of QPTs in fidelity mechanics. In this sense, a characterization of QPTs in the context of fidelity mechanics elaborates on different scenarios for a complete line of critical points, which manifest themselves in the ground-state phase diagram for a quantum many-body system.

As a sideline, we mention that a shift in fidelity temperature is performed to ensure that it is zero or minimum at a characteristic point. This requires the existence of a nonlocal unitary transformation that turns a highly entangled ground-state wave function at this point into an unentangled (factorized) state. In fact, the same nonlocal unitary transformation needs to be performed for any ground-state wave function at any interior point on a complete line of critical points such that our evaluation of the ground-state fidelity per lattice site remains intact. As a result, we even do not need to know the explicit form of the nonlocal unitary transformation in order to evaluate fidelity mechanical-state functions from an interior point of view. Physically, this amounts to stating that ground-state wave functions at all stable fixed points are unitarily equivalent, regardless of symmetry-breaking orders or topological orders given that fidelity temperatures are zero and fidelity internal energies take the maximum values at all stable fixed points. The only difference between symmetry-breaking orders or topological orders is reflected in the fact that such a unitary transformation is local or nonlocal. A specific point at which a unitary transformation needs to be performed is located at the self-dual point for scenario I-1, the U(1)-symmetric point for scenario I-2, the $Z_{2}$-symmetric point for scenario I-3, the FM point for scenario III-1, the PT point for scenario III-2, and the $S_{3}$-symmetric point for scenario III-3, respectively.

However, extra care must be exercised since there is a singularity at an FM transition point, an IC transition, and a PT transition point when they are approached along a complete line of critical points. The singularities arise because the two limiting procedures do not commute [107]: one is the thermodynamic limit N→∞ and the other is the limiting procedure representing how such a transition point is approached along a line of critical points.

### 6.3. A Characterization of Topological Quantum States of Matter in Fidelity Mechanics

It is natural to expect that the behaviors of fidelity entropy at a characteristic point or on a characteristic line play a crucial role in the characterization of quantum states of matter. An important observation is that a characteristic point located at infinity leads to an unentangled factorized ground state. That is, it results in a trivial stable fixed point, given that a characteristic point may be identified as either a stable fixed point or a metastable fixed point. Hence, it is crucial to track a characteristic point, which is located inside a cycle, as exemplified in Figure 10 for the quantum spin-1 XYZ model and the spin-1/2 Kitaev model on a honeycomb lattice. This is due to the fact that a nontrivial entangled ground state *only* occurs at a characteristic point, which must not be located at infinity. Indeed, the emergence of a cycle implies either the multiple-valuedness of fidelity entropy at a characteristic point or the double-valuedness of fidelity entropy on a characteristic line, both of which are located inside a cycle. Generically, if one is not able to discard the multi-valuedness of fidelity entropy at a characteristic point or the double-valuedness of fidelity entropy on a characteristic line in a given phase, then it must be a *topologically* ordered phase.

This happens in three situations for the six models under investigation when a cycle is formed from a few complete lines of critical points, some of which are symmetrical or dual relative to each other, as demonstrated in Figure 10. Specifically, for the quantum spin-1 XYZ model, a cycle is formed from the three complete lines of critical points in scenario I-2, which are dual in nature relative to each other. Here, a KT transition point, with central charge being c=1, is located at each of the three intersection points between two of the three complete lines of critical points. Note that central charge *c* is equal to 1/2 at an interior point on the cycle away from the three KT transition points, and a U(1)-symmetric point is located at the middle on each of the three complete lines of critical points. As it turns out, fidelity entropy is double-valued on the U(1) characteristic line in the Haldane phase (cf. Section 11). For the spin-1/2 Kitaev model on a honeycomb lattice, there are two cycles: One cycle is formed from the three complete lines of critical points in scenario I-3, which are dual to each other, and the other is formed from the two complete lines of critical points in scenario I-3, which are symmetrical to each other under a symmetric transformation: $J_{x}↔-J_{x}$, and $J_{y}↔-J_{y}$, together with their counterparts when $J_{x}$ becomes $-J_{x}$, with $J_{y}$ left intact. We stress that a critical point, dual to two symmetric QPT points, labelled as *C*, is located at infinity, when $J_{x}$ and $J_{y}$, proportional to each other, are infinite in value, as indicated in terms of an arrow in Figure 10b. This transition is from a gapped $Z_{2}$ spin liquid to a gapped $Z_{2}$ spin liquid, in contrast to a phase transition, labelled as TPT, from a gapless $Z_{2}$ spin liquid phase to a gapped $Z_{2}$ spin liquid phase at an interior point on the cycle. As it turns out, fidelity entropy is multiple-valued at the $S_{3}$ characteristic point or the U(1) characteristic point (cf. Section 12).

We emphasize that the double-valuedness of fidelity entropy on a characteristic line may be turned into the multiple-valuedness of fidelity entropy at a characteristic point via a smooth deformation of a dominant control parameter *x* and an auxiliary control parameter τ, and vice versa. Therefore, there is no essential difference between the two situations. We anticipate that more patterns for such a cycle show up in other quantum many-body systems (cf. Section 14). Here, by *topological*, we mean that fidelity entropy is not single-valued either at a QPT point or at a (meta)stable fixed point in the control parameter space. That is, the topological nature directly concerns the control parameter space—a novel feature that is not reflected in the conventional characterization. We stress that its possible ramifications for the conventional classifications of QPTs and quantum states of matter remain to be elaborated.

However, as far as the six illustrative models are concerned, our characterization of both topological QPTs and topological quantum states of matter in fidelity mechanics is consistent with the conventional classifications. A typical example is the conventional topological QPT–KT transitions, which appear also to be topological in the control parameter space. This will be illustrated in Section 9, Section 10 and Section 11, when we discuss fidelity mechanical-state functions for the quantum spin-1/2 XXZ model in an external magnetic field, the quantum spin-1/2 XYZ model, and the quantum spin-1 XYZ model. In addition, the SPT phases [108,109,110,111,112], the SPt phases [113,114], and the gapless and gapped $Z_{2}$ spin liquids [31] appear to be *topological* in the control parameter space. As it turns out, a nonlocal order parameter is essential for characterizing a topologically ordered state (cf. Appendix J about the bond-centered nonlocal order parameter for the SPT phases and the site-centered nonlocal order parameter for the SPt phases). This will be illustrated in Section 11 and Section 12, when we discuss fidelity mechanical-state functions for the quantum spin-1 XYZ model and the spin-1/2 Kitaev model on a honeycomb lattice in fidelity mechanics. Here, we remark that the Haldane phase for the quantum spin-*s* XYZ model is topological as a result of our characterization in the control parameter space. This is consistent with the original characterization by Haldane [65,66], who developed the nonlinear σ model approach to the spin-*s* AF Heisenberg model. In this approach, there is no essential difference between even integer *s* and odd integer *s*. In contrast, recent developments unveiled an intriguing difference between the two cases. Indeed, the Haldane phase for the spin-2 AF Heisenberg model is argued to be adiabatically connected to the large-*D* phase [115]—a typical example for the SPt phases [113], in contrast to the spin-1 case. One might take advantage of this argument to justify that the SPt phases are topological, although they are trivial in cohomological classifications [108,109,110,111] in contrast to the SPT phases.

## 7. Quantum Spin-1/2 XY Model—A Typical Example for Continuous Quantum Phase Transitions

**Figure 11:**
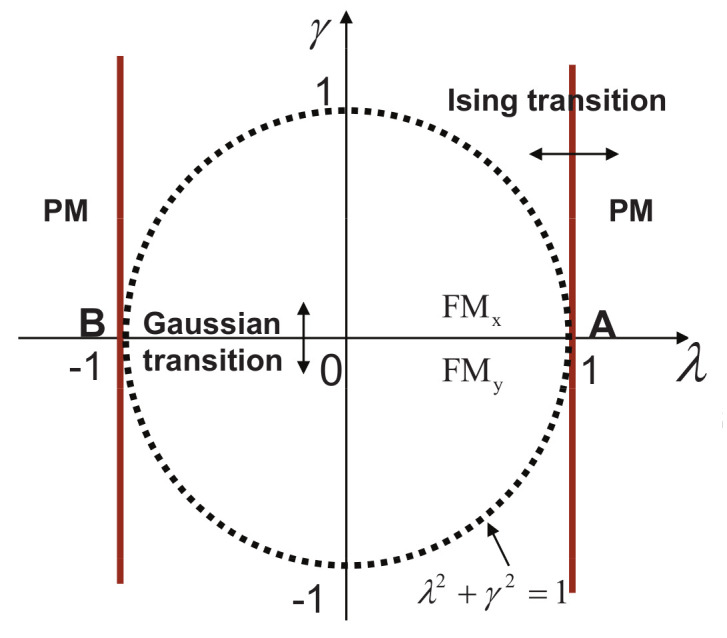
Ground-state phase diagram for the quantum spin-1/2 XY model. There is a marked difference between the regimes inside and outside the disordered circle. Indeed, as claimed [119], a long-range entanglement-driven order exists inside the disordered circle. However, we stress that the same order must also exist on the dual line (λ=0 with γ≥1) due to the presence of dualities between λ=0 with γ≥1 and λ=0 with γ≤1.

The model Hamiltonian (1) is in a canonical form, which is exactly solvable [116,117,118], with its ground-state phase diagram shown in Figure 11. For γ≠0, the Hamiltonian (1) possesses a $Z_{2}$ symmetry group, defined by $\sigma_{i}^{x}↔-\sigma_{i}^{x}$, $\sigma_{i}^{y}↔-\sigma_{i}^{y}$ and $\sigma_{i}^{z}↔\sigma_{i}^{z}$. For γ=0, the symmetry group becomes U(1). In addition, a U(1) symmetry occurs at two isolated points, λ=0 and γ=±1, as well as at a critical point when γ is infinite in value and at a characteristic point when λ is infinite in value.

The ground-state phase diagram may be read off from the singularities exhibited in the ground-state fidelity per lattice site (cf. Appendix A for the transverse-field quantum Ising model as a special case). The system undergoes QPTs, when the two lines of critical points (λ=1 with γ≠0, and γ=0 with −1<λ<1) are crossed in the thermodynamic limit. For a fixed γ, the model is driven to cross a critical point at λ=1 from an ordered FM phase to a disordered paramagnetic (PM) phase, which is a QPT belonging to the Ising universality class and characterized in terms of central charge c=1/2 in conformal field theories. Specifically, for γ>0 (γ<0), when λ∈(−1,1), the system is in the FM order along the x(y) direction, which is labelled as ${\mathrm{FM}}_{x}/{\mathrm{FM}}_{y}$ in Figure 11. For a fixed λ∈(−1,1), the system is driven through a Gaussian critical point at γ=0, with central charge c=1. For γ=0, a PT transition from a critical phase to a fully polarized phase occurs at λ=±1, protected by the symmetry group U(1). Note that, at two multi-critical points (±1,0), denoted as A and B in Figure 11, dynamic critical exponent *z* is z=2. Hence, the underlying field theories are not conformally invariant. As follows from our argument in Appendix I, the PT transition point is categorized as a trivial example for scale-invariant states, with the block entanglement entropy S(n) being zero when it is approached along the U(1)-symmetric line (γ=0), thus implying that the fractal dimension $d_{f}$ is zero. Here, we stress that the transition points (±1,0), labelled as PT, do not necessarily mean a PT transition point unless they are approached along the U(1)-symmetric line (γ=0).

The Hamiltonian (1) exhibits dualities for γ=1 and λ=0. If γ=1, it becomes the transverse-field quantum Ising model, which enjoys the Kramers–Wannier dualities [3,76]. If λ=0, a duality transformation emerges between the two regimes, γ>1 and 0<γ<1, implying that the model is critical, with central charge c=1, when γ is infinite in value. More details may be found in Appendix K.

An interesting feature of the Hamiltonian (1) is the disordered circle: $\lambda^{2}+\gamma^{2}=1$, characterized by the fact that ground states on the circle are factorized states [77,78,79,80,81,82]. As demonstrated in Ref. [59], the model on the disordered circle is unitarily equivalent to a spin-1/2 model with three-body interactions, for which its ground states are expressed in terms of matrix-product states with the bond dimension χ being equal to two. Therefore, we have to treat the transition points at (±1,0) as an exotic QPT given that the ground-state energy density e(λ,γ) is a constant on the disordered circle. In addition, there is a marked difference between the regimes inside and outside the disordered circle, away from the vertical axis λ=0. Indeed, as claimed [119], long-range entanglement-driven orders exist in the disordered regime. However, we stress that the same order must also exist on the dual line (λ=0 with γ≥1) due to the presence of dualities between λ=0 with γ≥1 and λ=0 with 0<γ≤1.

We may restrict ourselves to the region, defined by λ≥0 and γ≥0, since the Hamiltonian (1) is symmetrical with respect to γ↔−γ and λ↔−λ. Meanwhile, the consideration of the dualities and factorizing fields allows us to separate the entire region with γ>0 and λ>0 into five principal regimes (cf. Section 2): (I) the regime inside the disordered circle, with 0<λ<1 and $0<\gamma <\sqrt{1-\lambda^{2}}$; (II) the regime outside the disordered circle, with 0<λ<1 and $\sqrt{1-\lambda^{2}}<\gamma <1$; (III) the regime with 0<λ<1 and γ>1; (IV) the regime with λ>1 and 0<γ<1; (V) the regime with λ>1 and γ>1.

In each regime, we may choose a dominant control parameter *x* as long as such a choice is consistent with the constraints imposed by the symmetry groups, dualities, and factorizing fields, meaning that any choice has to respect all the boundaries between different regimes subject to a re-parametrization operation. Here, our choice is as follows: (1) for regime I, a dominant control parameter *x* was chosen to be γ, starting from $\gamma =\gamma_{c}=0$ up to the disordered circle, and an auxiliary control parameter τ is chosen to be λ; (2) for regime II or regime III, a dominant control parameter *x* was chosen to be 1−λ, starting from $\lambda =\lambda_{c}=1$ up to the disordered circle or λ=0, and an auxiliary control parameter τ is chosen to be γ; (3) for regime IV or regime V, a dominant control parameter *x* was chosen to be 1−1/λ starting from $\lambda =\lambda_{c}=1$ up to λ=∞, and an auxiliary control parameter τ is chosen to be γ. This choice was made to retain consistency with the Kramers–Wannier duality for the transverse-field quantum Ising model.

It is numerically confirmed that fidelity entropy S(λ,γ) scales as $\gamma^{\nu +1}$ near the line of the Gaussian critical points: γ=0 with −1<λ<1, and scales as ${\left|1-\lambda \right|}^{\nu +1}$ near the line of the Ising critical points: λ=1 with γ≠0. Here, ν is the critical exponent for the correlation length. In both cases, we have ν=1 (cf. Appendix H).

**Figure 12:**
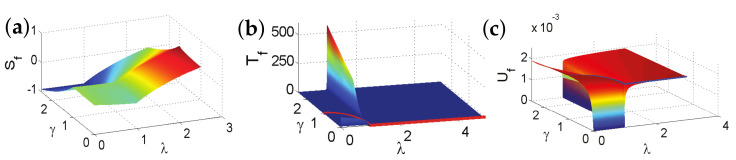
Fidelity entropy $S_{f}\left(\lambda ,\gamma \right)$, fidelity temperature $T_{f}\left(\lambda ,\gamma \right)$, and fidelity internal energy $U_{f}\left(\lambda ,\gamma \right)$ for the quantum spin-1/2 XY model. Here, we restrict ourselves to the region λ≥0 and γ≥0 due to the symmetry of the Hamiltonian (1) with respect to γ↔−γ and λ↔−λ. (**a**) Fidelity entropy $S_{f}\left(\lambda ,\gamma \right)$ exhibits singularities on two dual lines (γ=1 and λ=0), and on the disordered circle: $\lambda^{2}+\gamma^{2}=1$, in addition to two lines of critical points at γ=0 with 0<λ<1 and λ=1 with γ≠0. One might view such singularities as “phase transitions” in fidelity mechanics. Note also that fidelity entropy $S_{f}\left(\lambda ,\gamma \right)$ reaches its local maximum at (0,1). (**b**) Fidelity temperature $T_{f}\left(\lambda ,\gamma \right)$ diverges at two lines of critical points (γ=0 with 0<λ<1 and λ=1 with γ≠0), but it is zero on the disordered circle: $\lambda^{2}+\gamma^{2}=1$, as well as at a characteristic point, representing a factorizing field when λ is infinite in value. (**c**) Fidelity internal energy $U_{f}\left(\lambda ,\gamma \right)$ takes the same value at all stable fixed points and on the disordered circle: $\lambda^{2}+\gamma^{2}=1$, including a characteristic point at infinity representing a factorizing field when λ is infinite in value.

Once a dominant control parameter *x* and an auxiliary control parameter τ are chosen, fidelity entropy $S_{f}\left(\lambda ,\gamma \right)$ from an exterior point of view may be determined straightforwardly in the five principal regimes as well as on the characteristic lines, which appear as the boundaries between different principal regimes. Accordingly, fidelity temperature $T_{f}\left(\lambda ,\gamma \right)$ and fidelity internal energy $U_{f}\left(\lambda ,\gamma \right)$ are determined by solving a singular first-order differential equation for V(x,τ). The explicit expressions for fidelity entropy $S_{f}\left(\lambda ,\gamma \right)$, fidelity temperature $T_{f}\left(\lambda ,\gamma \right)$, and fidelity internal energy $U_{f}\left(\lambda ,\gamma \right)$ may be derived, following our prescription in Section 2 (also cf. scenario I-1 and scenario II-1 in Section 6). The details are presented in Appendix K. As a result, we plot fidelity entropy $S_{f}\left(\lambda ,\gamma \right)$, fidelity temperature $T_{f}\left(\lambda ,\gamma \right)$, and fidelity internal energy $U_{f}\left(\lambda ,\gamma \right)$ as a function of λ and γ in Figure 12a–c, respectively. Here, a contribution to fidelity entropy from rescaling due to dualities has been taken into account.

In addition to unstable fixed points, which are identified as critical points, there are three stable fixed points, identified as characteristic points located at (0,1), (∞,1), and (∞,0). Note that, at an unstable fixed point, fidelity temperature $T_{f}\left(\lambda ,\gamma \right)$ diverges, indicating strong quantum fluctuations, whereas at a stable fixed point, fidelity temperature $T_{f}\left(\lambda ,\gamma \right)$ is zero, indicating the absence of quantum fluctuations. This also happens on the disordered circle: $\lambda^{2}+\gamma^{2}=1$, with a factorized state as a ground state. That is, a factorizing-field line features zero-fidelity temperature in fidelity mechanics. However, at the PT transition point (1,0), fidelity temperature $T_{f}\left(\lambda ,\gamma \right)$ is not well-defined, and fidelity internal energy $U_{f}\left(\lambda ,\gamma \right)$ ranges from 0 to the maximum. In fact, fidelity temperature $T_{f}\left(\lambda ,\gamma \right)$ takes any value, ranging from 0 to ∞, depending on how it is approached, since all fidelity isothermal lines, defined as a line with the same constant values of fidelity temperature, converge at the PT transition point (cf. scenario I-1, scenario II-1, and scenario III-2 in Section 6). This bears a resemblance to a previous result [120] that entanglement entropy is not well-defined at the PT transition point (1, 0); its value depends on how the PT transition point (1,0) is approached.

We remark that, apart from QPTs detected through singularities in the ground-state fidelity per lattice site, fidelity mechanical-state functions exhibit singularities on the two dual lines (γ=1 and λ=0) and on the disordered circle: $\lambda^{2}+\gamma^{2}=1$. Note that the dual line (γ=1) consists of a principal part and its dual image part, and the dual line (λ=0) consists of a principal part and its dual image part in addition to the disordered circle. One might view such singularities as “phase transitions” in fidelity mechanics. This interpretation resolves a confusing point raised in [119]; as claimed, long-range entanglement-driven orders exist inside the disordered circle, suggesting that a QPT occurs on the disordered circle. However, the same long-range entanglement-driven order also exists on the dual line (λ=0 with γ≥1) due to the presence of duality between λ=0 with γ≥1 and λ=0 with 0<γ≤1. This indicates that no QPT occurs on the disordered circle. Otherwise, QPTs should also occur on the dual line (λ=0 with γ≥1).

We have to bear in mind that there are different choices of a dominant control parameter *x* in each regime yielding different fidelity mechanical-state functions. However, a connection exists between different choices, as discussed in Section 4. A crucial point is that both stable and unstable fixed points remain the same regardless of choices of a dominant control parameter *x* in a given regime.

Now we turn to fidelity mechanical-state functions on the two lines of critical points from an interior point of view: one is the line of the Gaussian critical points (γ=0 with −1<λ<1), and the other is the line of the Ising critical points (λ=1 with γ≠0).

**Figure 13:**
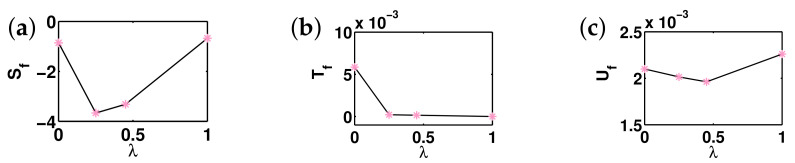
Fidelity entropy $S_{f}\left(\lambda ,0\right)$, fidelity temperature $T_{f}\left(\lambda ,0\right)$, and fidelity internal energy $U_{f}\left(\lambda ,0\right)$ for the quantum spin-1/2 XY model from an interior point of view on the line of the Gaussian critical points (γ=0 with 0<λ<1). This follows from an interior point of view in the two-dimensional critical XY regime for the quantum spin-1/2 XXZ model in a magnetic field (cf. scenario III-2 in Section 6). We remark that dot lines are only a guide for eye, with empty dots indicating our simulation results for λ=0, 0.25, 0.45, and 1. Here, the iTEBD algorithm is exploited to simulate the quantum spin-1/2 XXZ model in a magnetic field, with the bond dimension χ up to χ=60. (**a**) Fidelity entropy $S_{f}\left(\lambda ,0\right)$ is not monotonic as λ varies. (**b**) Fidelity temperature $T_{f}\left(\lambda ,0\right)$ is monotonic as λ varies. (**c**) Fidelity internal energy $U_{f}\left(\lambda ,0\right)$ is not monotonic as λ varies.

For the line of the Gaussian critical points (γ=0 with −1<λ<1), no dominant control parameter *x* is available, since the ground-state fidelity per lattice site $d(\lambda_{1},\lambda_{2})$ is simply zero, reflecting the fact that this line of critical points originates from the level crossings due to the presence of a U(1) generator, $\sum_{i}\sigma_{i}^{z}$, commuting with the Hamiltonian (1) when γ=0. As a result, any two ground states with different values of λ are orthogonal to each other (cf. scenario II-1 in Section 6). Instead, we have to embed the line of the Gaussian critical points (γ=0 with −1<λ<1) into the two-dimensional critical XY regime for the quantum spin-1/2 XXZ model in a magnetic field (4), to determine fidelity mechanical-state functions on the line of the Gaussian critical points (γ=0 with −1<λ<1) (cf. scenario III-2 in Section 6). As a consequence, fidelity entropy $S_{f}\left(\lambda ,0\right)$, fidelity temperature $T_{f}\left(\lambda ,0\right)$, and fidelity internal energy $U_{f}\left(\lambda ,0\right)$ as a function of *λ* are determined, with their numerical simulation results for λ=0, λ=0.25, λ=0.45, and λ=1 when γ=0 being plotted in Figure 13a–c, respectively. This indicates that fidelity entropy $S_{f}\left(\lambda ,0\right)$ and fidelity internal energy $U_{f}\left(\lambda ,0\right)$ are not monotonic, although fidelity temperature $T_{f}\left(\lambda ,0\right)$ seems to be monotonic as λ varies. However, we remark that the iTEBD algorithm is exploited to simulate the model (4) in the two-dimensional critical XY regime, with the bond dimension χ being only up to χ=60. Therefore, the results are less reliable, with a typical relative error for fidelity internal energy being around 5%.

**Figure 14:**
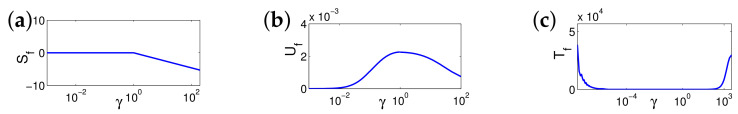
Fidelity entropy $S_{f}\left(1,\gamma \right)$, fidelity temperature $T_{f}\left(1,\gamma \right)$, and fidelity internal energy $U_{f}\left(1,\gamma \right)$ for the quantum spin-1/2 XY model from an interior point of view on the line of the Ising critical points (λ=1 with γ≠0). (**a**) Fidelity entropy $S_{f}\left(1,\gamma \right)$ monotonically increases when γ varies from γ=0 to γ=1 and from γ=∞ to γ=1, and it reaches its maximum at the characteristic point (1, 1). (**b**) Fidelity temperature $T_{f}\left(1,\gamma \right)$ diverges at (1, 0) and (1, ∞), but it is zero at the characteristic point (1, 1). (**c**) Fidelity internal energy $U_{f}\left(1,\gamma \right)$ monotonically increases when γ varies from γ=0 to γ=1 and from γ=∞ to γ=1, and it reaches its maximum at the characteristic point (1, 1).

As for the line of the Ising critical points (λ=1 with γ≠0), central charge *c* is 1 when γ is infinite in value, and *c* is 1/2 when γ is finite and non-zero. The line of the Ising critical points (λ=1 with γ≠0) may be divided into two principal parts: One is from γ=∞ to γ=1, and the other is from γ=0 to γ=1, since the self-dual point (1,1) acts as a characteristic point. Given the extent of γ is not finite in the principal part from ∞ to 1, a re-parametrization operation in the ground-state energy density e(λ,γ), as a function of γ, for a fixed λ=1, is necessary. Once this is performed, evaluating fidelity entropy $S_{f}\left(1,\gamma \right)$, fidelity temperature $T_{f}\left(1,\gamma \right)$, and fidelity internal energy $U_{f}\left(1,\gamma \right)$, as a function of γ, in the two principal parts is straightforward, with γ from ∞ to 1 and from 0 to 1, respectively. Mathematical details about their explicit expressions are presented in Appendix K. Here, we stress that shifts in fidelity temperature and fidelity internal energy are performed to ensure that fidelity temperature $T_{f}\left(1,\gamma \right)$ is zero at the characteristic point (1,1). This amounts to performing a nonlocal unitary transformation to remove entanglement from the ground-state wave function at the characteristic point (1,1), turning it into an unentangled (factorized) state. Note that it is necessary to perform this nonlocal unitary transformation for all ground states on the line of the Ising critical points (λ=1 with γ≠0) in order to evaluate fidelity entropy $S_{f}\left(1,\gamma \right)$ (cf. scenario I-1 in Section 6). We plot fidelity entropy $S_{f}\left(1,\gamma \right)$, fidelity temperature $T_{f}\left(1,\gamma \right)$, and fidelity internal energy $U_{f}\left(1,\gamma \right)$ as a function of γ in Figure 14a–c, respectively. As it turns out, fidelity entropy $S_{f}\left(1,\gamma \right)$ is single-valued at the transition point (1,0), labelled as PT. In fact, fidelity mechanical state functions from an interior point of view do not match that from an exterior point of view *only* if γ is nonzero and finite, when λ=1.

The single-valuedness of fidelity entropy at the PT transition point implies that it is not topological, consistent with the conventional characterization. We remark that an analogue of the Hawking radiation occurs at the PT transition point since fidelity temperature from an interior point of view is zero, which matches that from an exterior point of view when it is approached from the commuting U(1)-symmetric line (cf. Section 6).

## 8. Transverse-Field Quantum Ising Model in a Longitudinal Field—A Typical Example for Discontinuous Quantum Phase Transitions

The model Hamiltonian (2) is in a canonical form. When h=0, the Hamiltonian (2) becomes the transverse-field quantum Ising model and possesses $Z_{2}$ symmetry. It exhibits a second-order QPT at $\lambda_{c}=1$, characterized by the $Z_{2}$ symmetry-breaking order for λ<1. When h≠0 and λ≠0, no symmetry exists in the Hamiltonian. However, a U(1) symmetry occurs when λ=0, as well as when λ or *h* is infinite in value, or λ and *h*, proportional to each other, are infinite in value, with a factorized state as a ground state.

**Figure 15:**
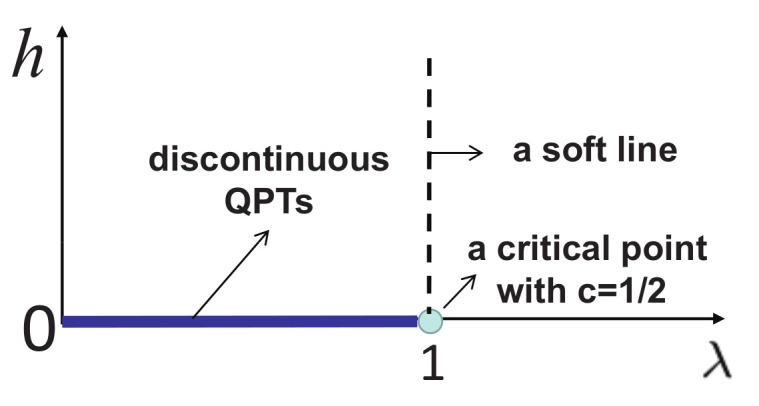
Ground-state phase diagram for the transverse-field quantum Ising model in a longitudinal field, with λ≥0 and h≥0. Here, a solid line represents a line of discontinuous QPT points (h=0 with 0≤λ<1), which ends at a critical point (1,0), and a dash line indicates a soft line. The discontinuous QPTs occur from a phase with spin polarization in −x to a phase with spin polarization in *x*, when *h* changes its sign.

As shown in Figure 15, the ground-state phase diagram is simple: There exists a line of discontinuous QPT points (h=0 with 0≤λ<1), which ends at the critical point (1,0). The discontinuous QPTs occur from a phase with spin polarization in −x to a phase with spin polarization in *x* when *h* changes its sign. As already mentioned, duality occurs in the transverse-field quantum Ising model. In addition, the Hamiltonian (2) is symmetrical with respect to h↔−h. Taking symmetries and dualities into account, we may divide the control parameter space into two principal regimes (cf. Section 2): regime I, defined as 0≤λ<1 and h≥0, and regime II, defined as λ≥1 and h≥0. Here, we remark that there are two principal regimes and three characteristic lines: one $Z_{2}$-symmetric line, one U(1)-symmetric line, and one soft line, together with factorizing fields located at infinity when *h* is infinite in value or when λ and *h* are infinite in value in proportionality, with the characteristic lines being the boundaries of the two principal regimes.

For the transverse-field quantum Ising model in a longitudinal field, we resort to the iTEBD algorithm [46,47,48] to generate the ground-state wave functions, with the bond dimension χ=60. It is numerically confirmed that fidelity entropy S(r,θ) scales as $r^{3/2}$ near the critical point (1,0) for θ≠0 and as $r^{2}$ for θ=0. This is consistent with the fact that the critical exponent ν for the correlation length is ν=1/2 for θ≠0 and ν=1 for θ=0 (cf. Appendix H).

In regime I, a dominant control parameter *x* is chosen to be x=h/(1+h), starting from h=0 up to h=∞, and an auxiliary control parameter τ is chosen to be τ=λ∈(0,1). Here, a re-parametrization operation in the ground-state energy density e(λ,h): $e\left(\lambda ,h\right)=m^{I}\left(x,\tau \right)e^{I}\left(x,\tau \right)$, with $m^{I}\left(x,\tau \right)=1/\left(1-x\right)$, is performed to ensure that $e^{I}\left(x,\tau \right)$ is monotonic with *x*. In regime II, a dominant control parameter *x* is chosen to be $x=\sqrt{{\left(\lambda -1\right)}^{2}+h^{2}}/\left(1+\sqrt{{\left(\lambda -1\right)}^{2}+h^{2}}\right)$, starting from the point (1,0) up to the point (∞,∞), and an auxiliary control parameter τ is chosen to be τ=arctanh/(λ−1)∈(0,π/2). Here, a re-parametrization operation in the ground-state energy density e(λ,h): $e\left(\lambda ,h\right)=m^{\mathrm{II}}\left(x,\tau \right)\;e^{\mathrm{II}}\left(x,\tau \right)$, with $m^{\mathrm{II}}\left(x,\tau \right)=1/\left(1-x\right)$, is performed to ensure that $e^{\mathrm{II}}\left(x,\tau \right)$ is monotonic with *x*. This choice is consistent with the Kramers–Wannier duality when τ=0.

Once a dominant control parameter *x* and an auxiliary control parameter τ are chosen, fidelity entropy $S_{f}\left(\lambda ,h\right)$ may be determined straightforwardly in the two principal regimes as well as on the characteristic lines, which appear as the boundaries between the two principal regimes or their symmetric image regimes. Accordingly, fidelity temperature $T_{f}\left(\lambda ,\gamma \right)$ and fidelity internal energy $U_{f}\left(\lambda ,\gamma \right)$ are determined by solving a singular or regular first-order differential equation for V(x,τ). The explicit expressions for fidelity entropy $S_{f}\left(\lambda ,h\right)$, fidelity temperature $T_{f}\left(\lambda ,h\right)$, and fidelity internal energy $U_{f}\left(\lambda ,h\right)$ may be derived, following from our prescription in Section 2. The details are presented in Appendix L.

**Figure 16:**
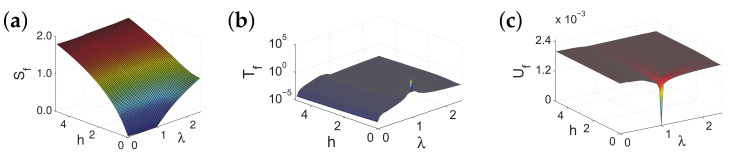
Fidelity entropy $S_{f}\left(\lambda ,h\right)$, fidelity temperature $T_{f}\left(\lambda ,h\right)$, and fidelity internal energy $U_{f}\left(\lambda ,h\right)$ as a function of λ and *h* for the transverse-field quantum Ising model in a longitudinal field, with λ≥0 and h≥0. (**a**) There exist two singular lines (h=0 and λ=1), in fidelity entropy $S_{f}\left(\lambda ,h\right)$. Note that singularities on the dual line (h=0) arise from duality and should be attributed to the $Z_{2}$ symmetry, whereas singularities on λ=1 reflects the fact that spins point towards the +x direction for 0≤λ<1 when *h* is infinite in value and towards other directions for λ>1 when λ and *h*, proportional to each other, are infinite in value. (**b**) Fidelity temperature $T_{f}\left(\lambda ,h\right)$ diverges at the critical point (1, 0) but vanishes when λ or *h* is infinite in value or λ and *h*, proportional to each other, are infinite in value. (**c**) Fidelity internal energy $U_{f}\left(\lambda ,h\right)$ takes the maximum at all stable fixed points, as well as at factorizing fields, when λ or *h* is infinite in value or λ and *h*, proportional to each other, are infinite in value.

We plot fidelity entropy $S_{f}\left(\lambda ,h\right)$, fidelity temperature $T_{f}\left(\lambda ,h\right)$, and fidelity internal energy $U_{f}\left(\lambda ,h\right)$ as a function of λ and *h* in Figure 16. Here, a contribution to fidelity entropy from a re-parametrization operation has been taken into account. Fidelity entropy $S_{f}\left(\lambda ,h\right)$ reaches a local maximum when λ=1 and reaches the maximum when λ=0, if scaling entropy ln(1+|h|) is excluded. This is consistent with the existence of stable fixed points at (0,0), (0,∞), (∞,0), and (1,∞), which are seen as characteristic points in the control parameter space. We remark that (1,∞) should be identified with (0,∞), although the existence of soft line (λ=1) might be related to a well-known fact that, at nonzero *h*, a massive excitation spectrum involves eight massive particles, which shows a deep relation with the algebraic structure represented by the exceptional Lie algebra $E_{8}$, as predicted in perturbed conformal field theories [121]. The existence of stable fixed points (0,0) and (∞,0) is protected by the $Z_{2}$ symmetry when h=0, whereas the existence of stable fixed point (0,∞) may be interpreted as a consequence of the variation of the symmetry group with λ: U(1) for λ=0. Fidelity temperature $T_{f}\left(\lambda ,h\right)$ diverges at the critical point (1,0) and reaches zero at stable fixed points, as well as when λ or *h* is infinite in value or λ and *h*, proportional to each other, are infinite in value.

We remark that, in addition to the critical point (1,0) and the line of discontinuous QPT points (h=0 with 0≤λ<1), detected through singularities in the ground-state fidelity per lattice site, fidelity mechanical-state functions exhibit singularities on the dual line (h=0), the U(1)-symmetric line (λ=0) and the soft line (λ=1). One might view such singularities as “phase transitions” in fidelity mechanics. Note that singularities on the dual line (h=0) arise from duality and should be attributed to the $Z_{2}$ symmetry, whereas singularities on λ=1 reflects the fact that spins point towards the +x direction for λ<1 when *h* is infinite in value and towards other directions for λ>1 when λ and *h* are infinite in value and in proportionality. Thus, it is different from characteristic lines arising from symmetries, dualities, and factorizing fields (cf. Section 4). Furthermore, fidelity internal energy $U_{f}\left(\lambda ,h\right)$ takes the same maximum value at all stable fixed points, as well as at factorizing fields, when λ or *h* is infinite in value or λ and *h*, proportional to each other, are infinite in value.

The Hamiltonian (2) does not entail an interior point of view for a complete line of critical points. Instead, it hosts a line of discontinuous QPT points, ending at an isolated critical point. As it turns out, the critical point (1,0) is not topological since fidelity entropy $S_{f}\left(\lambda ,h\right)$ is single-valued.

## 9. Quantum Spin-1/2 XYZ Model—A Typical Example for Dualities

**Figure 17:**
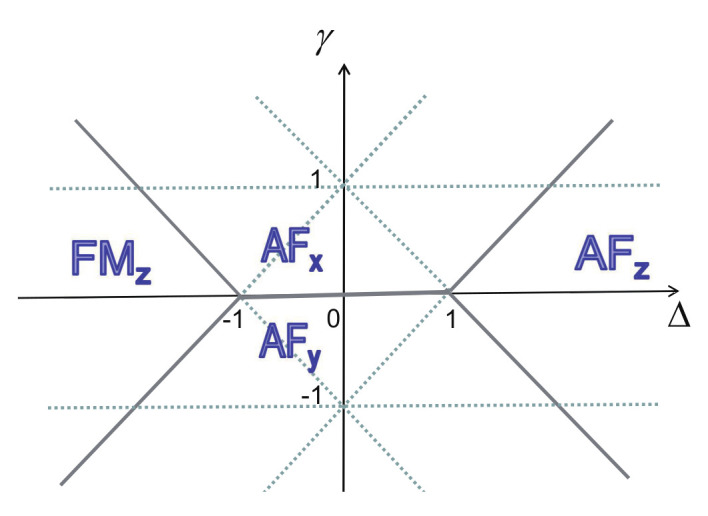
Ground-state phase diagram for the quantum spin-1/2 XYZ model. There are four different phases, labelled as ${\mathrm{AF}}_{x}$, ${\mathrm{AF}}_{y}$, ${\mathrm{AF}}_{z}$, and ${\mathrm{FM}}_{z}$, representing an AF phase in the *x* direction, an AF phase in the *y* direction, an AF phase in the *z* direction, and an FM phase in the *z* direction, respectively. Solid lines (γ=0 with −1<Δ≤1, γ=1+Δ with Δ<−1, γ=1−Δ with Δ≥1, γ=−1−Δ with Δ<−1, and γ=−1+Δ with Δ≥1), denote its phase boundaries between different phases. In particular, FM points are located at (−1, 0) and its dual images and the KT transition point are located at (1, 0) and its dual image. In addition, factorizing fields occur on two lines: γ=1+Δ and γ=−1−Δ, with Δ>−1, as well as when |Δ| is infinite in value. Moreover, a critical point at infinity occurs when |γ| is infinite in value.

The model Hamiltonian (3) is in a canonical form, which is exactly solvable by means of the Bethe ansatz via its equivalence to the eight-vertex model [122,123,124,125,126]. Its ground-state phase diagram is shown in Figure 17. There are four different phases, labelled as ${\mathrm{AF}}_{x}$, ${\mathrm{AF}}_{y}$, ${\mathrm{AF}}_{z}$, and ${\mathrm{FM}}_{z}$, representing an AF phase in the *x* direction, an AF phase in the *y* direction, an AF phase in the *z* direction, and an FM phase in the *z* direction, respectively. In addition, there are five lines of critical points, depicted as five solid lines in Figure 17. For γ=0, a line of critical points exists between −1<Δ≤1, which is the critical XY phase, with central charge c=1. The KT transition point is located at Δ=1 and γ=0, protected by a U(1) symmetry, from the critical XY phase to the ${\mathrm{AF}}_{z}$ phase for Δ>1. For γ≠0, four lines of critical points exist, two of which are γ=−1+Δ with Δ≥1 and γ=−1−Δ with Δ<−1 that are symmetrical to γ=1−Δ with Δ≥1 and γ=1+Δ with Δ<−1, respectively. They represent dual images of the critical XY regime, located on γ=0, with −1<Δ≤1. Note that, along the U(1)-symmetric lines (γ=−1+Δ and γ=1−Δ), the KT transitions occur at Δ=1, protected by a U(1) symmetry, from a critical phase to the ${\mathrm{AF}}_{y}$ phase and the ${\mathrm{AF}}_{x}$ phase for Δ<1, respectively. When Δ is minus infinite in value, it yields a factorized ground state in the ${\mathrm{FM}}_{z}$ phase. When Δ is plus infinite in value, it yields a factorized ground state in the ${\mathrm{AF}}_{z}$ phase. In addition, γ=1+Δ and γ=−1−Δ, with Δ>−1, represent two factorizing-field lines [119]. Moreover, a critical point, with central charge c=1, occurs when γ is infinite in value for any fixed Δ.

The Hamiltonian (3) possesses the dihedral symmetry group $Z_{2}\times Z_{2}$, generated from any two of the three π-rotations around the *x*-, *y*- and *z*-axes, e.g., the *x*- and *z*-axes: $\sigma_{i}^{x}↔\sigma_{i}^{x}$, $\sigma_{i}^{y}↔-\sigma_{i}^{y}$ and $\sigma_{i}^{z}↔-\sigma_{i}^{z}$ and $\sigma_{i}^{x}↔-\sigma_{i}^{x}$, $\sigma_{i}^{y}↔-\sigma_{i}^{y}$ and $\sigma_{i}^{z}↔\sigma_{i}^{z}$, respectively. In addition to γ=0, there are four U(1)-symmetric lines, which are located at γ=1−Δ, γ=1+Δ, γ=−1+Δ, and γ=−1−Δ. Meanwhile, a U(1) symmetry occurs when |Δ| or |γ| is infinite in value. Furthermore, a SU(2) symmetry emerges at the two characteristic points (±1,0) and their dual image points.

As demonstrated in Appendix C, there are five dualities for the Hamiltonian (3) arising from the symmetric group $S_{3}$ with respect to *x*, *y*, and *z*, thus leading to five symmetric or self-dual lines, which are identical to five U(1)-symmetric lines in addition to three semi-self-dual lines located at γ=±1 and Δ=0.

The Hamiltonian (3) is symmetrical with respect to γ↔−γ. Therefore, we may restrict ourselves to the region γ≥0. Taking into account the symmetries, dualities, and factorizing fields [77,78,79], we may divide the region γ≥0 into twelve different regimes, with five lines defined by γ=1 and γ=±1±Δ as the boundaries. The twelve regimes are separated into two groups, with six regimes in each group that are dual in nature relative to each other. As shown in Section 2, regime I, regime III, regime V, regime VII, regime IX, and regime XI are dual relative to each other, whereas regime II, regime IV, regime VI, regime VIII, regime X, and regime XII are dual relative to each other. Therefore, there are only two principal regimes, which represent the physics underlying the quantum spin-1/2 XYZ model. Here, we chose regime I and regime II as the two principal regimes.

A choice of a dominant control parameter *x* and an auxiliary control parameter τ has been made for each of the two principal regimes (cf. Section 2). In regime I and regime II, we chose a dominant control parameter *x* to be x=γ and an auxiliary control parameter τ to be τ=Δ. In regime I, γ ranges from $\gamma =\gamma_{c}$, with $\gamma_{c}=0$, to a point on the U(1)-symmetric line: γ=1−Δ, for a fixed Δ∈(0,1). In regime II, γ ranges from $\gamma =\gamma_{c}$, with $\gamma_{c}=0$, to a point on the U(1)-symmetric line: γ=1+Δ, for a fixed Δ∈(−1,0). However, other choices are possible, as long as such a choice is consistent with the constraints imposed by the symmetries, dualities, and factorizing fields (cf. Section 4).

It is numerically confirmed that fidelity entropy S(Δ,γ) scales as $\gamma^{\nu (\Delta )+1}$ near the line of the Gaussian critical points (γ=0 with −1<Δ≤1). Here, ν(Δ) is the critical exponent for the correlation length, which varies with Δ. This is consistent with the fact that the transition belongs to the Gaussian universality class (cf. Appendix H). Note that the iTEBD algorithm is exploited to generate the ground-state wave functions, with the bond dimension χ=60.

Once a dominant control parameter *x* and an auxiliary control parameter τ are chosen in regime I and regime II, fidelity entropy $S_{f}\left(\Delta ,\gamma \right)$ may be determined straightforwardly in the two principal regimes as well as on the characteristic lines, which appear as the boundaries between the two principal regimes or their dual image regimes. Accordingly, fidelity temperature $T_{f}\left(\Delta ,\gamma \right)$ and fidelity internal energy $U_{f}\left(\Delta ,\gamma \right)$ are determined by solving a singular first-order differential equation for V(x,τ). Then, fidelity mechanical-state functions in the non-principal regimes, which are dual image regimes, simply follow from their respective dualities to regime I and regime II. However, the ground-state energy density e(Δ,γ) is not monotonic on the U(1)-symmetric line (γ=1−Δ with 0≤Δ≤1). Therefore, it is necessary to perform a re-parametrization operation in the ground-state energy density e(Δ,1−Δ): $e\left(\Delta ,1-\Delta \right)=m^{\mathrm{ii}}\left(x\right)e^{\mathrm{ii}}\left(x\right)$, with x=1−Δ/(2−Δ) and $m^{\mathrm{ii}}\left(x\right)=2/\left(1+x\right)$. In addition, there is an alternative choice for a re-parametrization operation in the ground-state energy density e(Δ,1−Δ): $e\left(\Delta ,1-\Delta \right)=m_{A}^{\mathrm{ii}}\left(x\right)e_{A}^{\mathrm{ii}}\left(x\right)$, with $m_{A}^{\mathrm{ii}}\left(x\right)=1/\left(2-x\right)$. The explicit expressions for fidelity entropy $S_{f}\left(\Delta ,\gamma \right)$, fidelity temperature $T_{f}\left(\Delta ,\gamma \right)$, and fidelity internal energy $U_{f}\left(\Delta ,\gamma \right)$ may be derived following from our prescription in Section 2 (also cf. scenario III-1 in Section 6). The details are presented in Appendix M.

**Figure 18:**
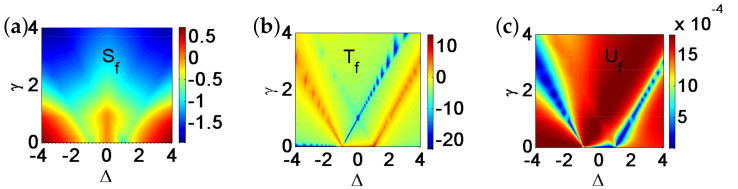
Fidelity entropy $S_{f}\left(\Delta ,\gamma \right)$, fidelity temperature $T_{f}\left(\Delta ,\gamma \right)$, and fidelity internal energy $U_{f}\left(\Delta ,\gamma \right)$ for the quantum spin-1/2 XYZ model, with γ≥0, from an exterior point of view. (**a**) Fidelity entropy $S_{f}\left(\Delta ,\gamma \right)$ exhibits a local maximum at a stable fixed point (1,0). Singularities occur on the three lines of the Gaussian critical points: γ=0 with −1<Δ≤1, γ=−1+Δ with Δ≥1, and γ=−1−Δ with Δ<−1, on the factorizing-field line (γ=1+Δ with Δ>−1), and on the three characteristic lines: One is a U(1)-symmetric and self-dual line (γ=1−Δ with Δ≤1) and the other two are semi-self-dual lines (γ=1 and Δ=0). (**b**) Fidelity temperature $T_{f}\left(\Delta ,\gamma \right)$ diverges at the three lines of the Gaussian critical points: γ=0 with −1<Δ≤1, γ=−1+Δ with Δ≥1, and γ=−1−Δ with Δ<−1; it is zero on the factorizing-field line (γ=1+Δ with Δ>−1), as well as when |Δ| is infinite in value. (**c**) Fidelity internal energy $U_{f}\left(\Delta ,\gamma \right)$ takes the same maximum value at all stable fixed points ((0,1), (±∞,0), and (±∞,1)); on the factorizing-field line (γ=1+Δ with Δ>−1; and at factorizing fields when |Δ| is infinite in value. Note that at the FM transition point (−1, 0), fidelity temperature $T_{f}$, and fidelity internal energy $U_{f}$ are not well-defined; fidelity temperature $T_{f}$ ranges from 0 to infinity and fidelity internal energy $U_{f}$ ranges from 0 to the maximum depending on how the FM transition point (−1, 0) is approached.

A contour plot is depicted in Figure 18a for fidelity entropy $S_{f}\left(\Delta ,\gamma \right)$ in the control parameter space, with γ≥0. Here, a contribution to fidelity entropy from rescaling in the ground-state energy density, due to a re-parametrization operation and dualities, has been taken into account (cf. Appendix G). Fidelity entropy $S_{f}\left(\Delta ,\gamma \right)$ takes a local maximum at the U(1)-symmetric point (0,1) and its dual images. Fidelity temperature $T_{f}\left(\Delta ,\gamma \right)$ and fidelity internal energy $U_{f}\left(\Delta ,\gamma \right)$ are shown as contour plots in Figure 18b,c, respectively. As observed, fidelity temperature $T_{f}\left(\Delta ,\gamma \right)$ diverges at the three lines of the Gaussian critical points: γ=0 with −1<Δ≤1, γ=−1+Δ with Δ≥1, and γ=−1−Δ with Δ<−1 and vanishes on the factorizing-field line (γ=1+Δ with Δ>−1), in addition to two characteristic lines at infinity when |Δ| is infinite in value. We remark that there are five stable fixed points, identified as characteristic points (away from the three lines of the Gaussian critical points), in the region γ≥0: (0,1), (±∞,0), and (±∞,1).

Fidelity entropy $S_{f}\left(\Delta ,\gamma \right)$, fidelity temperature $T_{f}\left(\Delta ,\gamma \right)$, and fidelity internal energy $U_{f}\left(\Delta ,\gamma \right)$ exhibit singular behaviors on the three lines of the Gaussian critical points: γ=0 with −1<Δ≤1, γ=−1+Δ with Δ≥1, and γ=−1−Δ with Δ<−1; on the factorizing-field line (γ=1+Δ with Δ>−1); and on the three characteristic lines: one is the U(1)-symmetric and self-dual line (γ=1−Δ with Δ≤1), and the other two are semi-self-dual lines (γ=1 and Δ=0). This singular behavior may be recognized as “phase transitions” in fidelity mechanics. In addition, fidelity internal energy $U_{f}\left(\Delta ,\gamma \right)$ takes the same maximum value at all stable fixed points (0,1), (±∞,0), and (±∞,1), as well as on the factorizing-field line. Physically, this amounts to stating that ground-state wave functions at all stable fixed points are unitarily equivalent. Note that at the FM transition point (−1, 0), fidelity temperature $T_{f}$ and fidelity internal energy $U_{f}$ are not well-defined; fidelity temperature $T_{f}$ ranges from 0 to infinity and fidelity internal energy $U_{f}$ ranges from 0 to the maximum depending on how the FM transition point (−1, 0) is approached. This is similar to the PT transition point for the quantum spin-1/2 XY model. However, the ground states are highly degenerate at the FM transition point (−1, 0), whereas the ground state at the PT transition point is non-degenerate. It is worth mentioning that this characterization of the FM transition point is consistent with the fact that, at the FM transition point (−1, 0), the Hamiltonian (3) admits highly degenerate and highly entangled ground states, which arise from SSB with one type-B GM when SU(2) is spontaneously broken to U(1) [102]. As it turns out, the ground states are scale-invariant, with the fractal dimension $d_{f}$ being identified as the number $N_{B}$ of type-B GMs: $d_{f}=N_{B}=1$ (also cf. Appendix I).

We turn to fidelity mechanical-state functions on the three lines of the Gaussian critical points from an interior point of view: γ=0 with −1<Δ≤1, γ=−1+Δ with Δ≥1, and γ=−1−Δ with Δ<−1. Since the three lines of the Gaussian critical points are dual relative to each other, we only need to consider the line of the Gaussian critical points (γ=0 with −1<Δ≤1)—a complete line of critical points (cf. scenario III-1 in Section 6).

Since no characteristic points exist between the two endpoints from an interior point of view, there is only one principal part. Once a dominant control parameter *x* is chosen in this principal part, evaluating fidelity entropy $S_{f}\left(\Delta ,0\right)$ with −1<Δ≤1 is straightforward. Then, fidelity temperature $T_{f}\left(\Delta ,0\right)$ and fidelity internal energy $U_{f}\left(\Delta ,0\right)$, as a function of Δ, with −1<Δ≤1, ffollow from solving a singular first-order differential equation for V(x). Mathematical details about their explicit expressions are presented in Appendix M.

**Figure 19:**
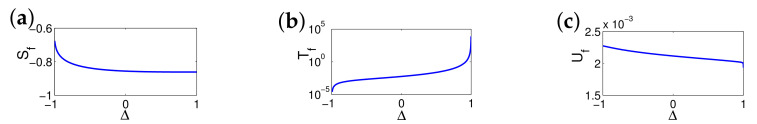
Fidelity entropy $S_{f}\left(\Delta ,0\right)$, fidelity temperature $T_{f}\left(\Delta ,0\right)$, and fidelity internal energy $U_{f}\left(\Delta ,0\right)$ on the complete line of critical points (γ=0 with −1<Δ<1) for the quantum spin-1/2 XYZ model from an interior point of view. (**a**) Fidelity entropy monotonically increases when Δ varies from Δ=1 to Δ=−1, and reaches its maximum at the FM transition point (−1, 0). (**b**) Fidelity temperature $T_{f}\left(\Delta ,0\right)$ diverges at Δ=1, but it is zero at the FM transition point (−1, 0). (**c**) Fidelity internal energy $U_{f}\left(\Delta ,0\right)$ monotonically increases when Δ varies from Δ=1 to Δ=−1, and it reaches its maximum at the FM transition point (−1, 0). Note that fidelity entropy $S_{f}\left(\Delta ,0\right)$, fidelity temperature $T_{f}\left(\Delta ,0\right)$, and fidelity internal energy $U_{f}\left(\Delta ,0\right)$ at Δ=−1 from an interior point of view only match those at Δ=−1 from an exterior point of view. Therefore, fidelity entropy $S_{f}\left(\Delta ,\gamma \right)$ is double-valued at the KT transition point (1, 0). Hence, a KT transition is topological in our characterization, consistent with the conventional classification.

We plot fidelity entropy $S_{f}\left(\Delta ,0\right)$, fidelity temperature $T_{f}\left(\Delta ,0\right)$, and fidelity internal energy $U_{f}\left(\Delta ,0\right)$, from an interior point of view, as a function of Δ, with −1<Δ≤1, in Figure 19a–c, respectively: (a) Fidelity entropy $S_{f}\left(\Delta ,0\right)$ monotonically increases when Δ varies from Δ=1 to Δ=−1, and it reaches its maximum at the FM transition point (−1, 0); (b) fidelity temperature $T_{f}\left(\Delta ,0\right)$ diverges at the KT transition point (1, 0), but it is zero at the FM transition point (−1, 0); (c) fidelity internal energy $U_{f}\left(\Delta ,0\right)$ monotonically increases when Δ varies from Δ=1 to Δ=−1, and it reaches its maximum at the FM transition point (−1, 0).

According to our prescription, shifts in fidelity temperature and fidelity internal energy are performed to ensure that $T_{f}\left(\Delta ,0\right)$ is zero and $U_{f}\left(\Delta ,0\right)$ is the maximum at the FM transition point (−1, 0). This demands the existence of a nonlocal unitary transformation that turns a highly entangled ground-state wave function at this transition point into an unentangled (factorized) state, as discussed in Section 6. Physically, this amounts to stating that ground-state wave functions at all stable fixed points are unitarily equivalent. Meanwhile, at the FM transition point, a singularity arises from the fact that the two limiting procedures do not commute [107]: one is the thermodynamic limit N→∞ and the other is the limiting procedure representing how such a transition point is approached along the complete line of critical points.

The single valuedness of fidelity entropy at the FM transition point implies that it is not topological, consistent with the conventional characterization. In contrast, the double valuedness of fidelity entropy at the KT transition point implies that it is topological in our characterization (cf. scenario III-1 in Section 6). We remark that an analogue of the Hawking radiation occurs at the FM transition point and the KT transition point. Indeed, fidelity temperature from an interior point of view is zero at the FM transition point and diverges at the KT transition point, matching that from an exterior point of view when they are approached along the non-commuting U(1)-symmetric line.

## 10. Quantum Spin-1/2 XXZ Model in a Magnetic Field—An Intermediate Case between the Kosterlitz–Thouless Transitions and the Pokrovsky–Talapov Transitions

**Figure 20:**
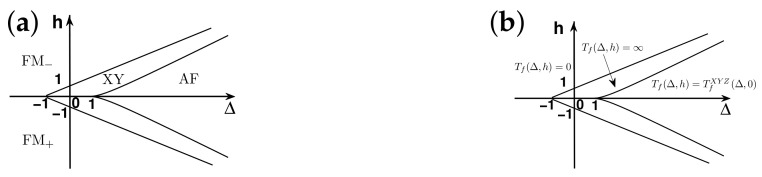
(**a**) Ground-state phase diagram for the quantum spin-1/2 XXZ model in a magnetic field. Note that the model is symmetrical with respect to h↔−h: $S_{i}^{x}↔S_{i}^{y}$ and $S_{i}^{z}↔-S_{i}^{z}$. There are four phases labelled as AF, ${\mathrm{FM}}_{-}$, ${\mathrm{FM}}_{+}$, and XY representing an AF phase, an FM phase with all spin down, an FM phase with all spin up, and the critical XY phase with central charge c=1, respectively. Here, the phase boundary between the critical XY phase and the AF phase asymptotically approaches a line of the PT transition points between the ${\mathrm{FM}}_{-}$/${\mathrm{FM}}_{+}$ phase and the critical XY phase for h>0/h<0, respectively. In particular, the FM transition point is located at (−1,0) and the KT transition point is located at (1,0). (**b**) Fidelity temperature $T_{f}\left(\Delta ,h\right)$ for the quantum spin-1/2 XXZ model in a magnetic field. In the phases ${\mathrm{FM}}_{-}$ and ${\mathrm{FM}}_{+}$, it is zero; in the critical XY phase, it diverges; in the AF phase, it takes the same value as fidelity temperature $T_{f}^{\mathrm{XYZ}}\left(\Delta ,0\right)$ for the quantum spin-1/2 XYZ model when Δ>1. Here, the (curved) line of the IC transition points is reproduced from the exact result by means of the Bethe ansatz [128].

The model Hamiltonian (4) is in a canonical form, which is exactly solvable by means of the Bethe ansatz [127,128,129,130,131,132], with its ground-state phase diagram shown in Figure 20. There are four phases, labelled as AF, ${\mathrm{FM}}_{-}$, ${\mathrm{FM}}_{+}$, and XY, representing an AF phase, an FM phase with all spin down, an FM phase with all spin up, and a critical XY phase with central charge c=1, respectively. We may restrict ourselves to the region h≥0, since the Hamiltonian (4) is symmetrical with respect to h↔−h: $\sigma_{i}^{x}↔\sigma_{i}^{y}$ and $\sigma_{i}^{z}↔-\sigma_{i}^{z}$. There are two lines of QPT points: One is a line of the PT transition points, defined by $h_{p}=1+\Delta_{p}$, and the other is a (curved) line of the IC transition points, defined by $h_{c}=\phi \left(\Delta_{c}\right)$, with $\Delta_{c}>1$. The latter has been worked out exactly [128], which is reproduced in Figure 20. Meanwhile, the consideration of the two lines of QPT points and the $Z_{2}$-symmetric line leads us to separate the entire region h≥0 into four principal regimes: regime I, regime II, regime III, and regime IV.

A choice of a dominant control parameter *x* and an auxiliary control parameter τ has been made for each of the four principal regimes (cf. Section 2). In regime I, a dominant control parameter *x* was chosen to be $x=\sqrt{{\left(\Delta +1\right)}^{2}+h^{2}}/\left(1+\sqrt{{\left(\Delta +1\right)}^{2}+h^{2}}\right)$, starting from the point (−1,0) up to the point (−∞,−∞), and an auxiliary control parameter τ was chosen to be τ=arctan(h/(Δ+1))∈(π/2,π]. Here, a re-parametrization operation in the ground-state energy density e(Δ,h): $e\left(\Delta ,h\right)=m^{I}\left(x,\tau \right)\;e^{I}\left(x,\tau \right)$, with $m^{I}\left(x,\tau \right)=\left(2\sin\tau -\cos\tau \right)x/\left(1-x\right)+1$, is performed to ensure that $e^{I}\left(x,\tau \right)$ is a constant: $e^{I}\left(x,\tau \right)=-1$. In regime II, a dominant control parameter *x* was chosen to be x=1−1/(h−Δ), starting from h=Δ+1 up to h=∞, and an auxiliary control parameter τ was chosen to be τ=Δ∈[−1,∞). Here, a re-parametrization operation in the ground-state energy density e(Δ,h): $e\left(\Delta ,h\right)=m^{\mathrm{II}}\left(x,\tau \right)\;e^{\mathrm{II}}\left(x,\tau \right)$, with $m^{\mathrm{II}}\left(x,\tau \right)=\tau +2/\left(1-x\right)$, is performed to ensure that $e^{\mathrm{II}}\left(x,\tau \right)$ is a constant: $e^{\mathrm{II}}\left(x,\tau \right)=-1$. In regime III, a dominant control parameter *x* was chosen to be x=−Δ, starting from $\Delta =\Delta_{c}$ up to Δ=h−1, and an auxiliary control parameter τ was chosen to be τ=h∈(0,∞), with $\Delta_{c}$ being a QPT point on the line of the IC transition points, for a fixed τ. Here, a re-parametrization operation in the ground-state energy density e(Δ,h): $e\left(\Delta ,h\right)=m^{\mathrm{III}}\left(x,\tau \right)\;e^{\mathrm{III}}\left(x,\tau \right)$, with $m^{\mathrm{III}}\left(x,\tau \right)=1/\left(\Delta_{c}+x+1\right)$, is performed to ensure that $e^{\mathrm{III}}\left(x,\tau \right)$ is monotonic with *x* for a fixed τ. Here, $\left(\Delta_{c},h_{c}\right)$ denotes an IC transition point. In regime IV, a dominant control parameter *x* was chosen to be $x=h_{c}-h$, starting from $h=h_{c}$ up to h=0, and an auxiliary control parameter τ was chosen to be τ=Δ∈(1,∞).

Once a dominant control parameter *x* is chosen, fidelity entropy $S_{f}\left(\Delta ,h\right)$ may be determined straightforwardly. Accordingly, fidelity temperature $T_{f}\left(\Delta ,h\right)$ and fidelity internal energy $U_{f}\left(\Delta ,h\right)$ are determined by solving a singular first-order differential equation for V(Δ,h). In the four principal regimes as well as on the characteristic lines as the boundaries between different principal regimes, the explicit expressions for fidelity entropy $S_{f}\left(\Delta ,h\right)$, fidelity temperature $T_{f}\left(\Delta ,h\right)$, and fidelity internal energy $U_{f}\left(\Delta ,h\right)$ may be derived, following from our prescription in Section 2 (also cf. scenario III-1 and scenario III-2 in Section 6).

In the entire ${\mathrm{FM}}_{-}$ and ${\mathrm{FM}}_{+}$ phases, up to the phase boundary between the FM phases and the XY phase, the ground state remains the same: a spin-polarized state with all spins down for h>0 and a spin-polarized state with all spins up for h<0. The two states coexist when h=0. Therefore, there is a discontinuous QPT if the transition line (h=0) is crossed, where the $Z_{2}$ symmetry, defined by $\sigma_{i}^{x}↔\sigma_{i}^{y}$ and $\sigma_{i}^{z}↔-\sigma_{i}^{z}$, is spontaneously broken. As shown in Figure 20, the soft line (Δ=−1) divides the ${\mathrm{FM}}_{-}$ phase into two distinct principal regimes: regime I and regime II. For the chosen dominant control parameter *x* and auxiliary control parameter τ, the ground-state energy density is a constant, i.e., $e^{I}\left(x,\tau \right)=e^{\mathrm{II}}\left(x,\tau \right)=-1$. Therefore, fidelity entropy $S_{f}\left(\Delta ,h\right)$ only includes a contribution from scaling entropy, which takes the following form: $S_{\sigma }\left(\Delta ,h\right)=\ln\left(2h-\Delta \right)$. Then, fidelity temperature $T_{f}\left(\Delta ,h\right)$ is zero, and fidelity internal energy $U_{f}\left(\Delta ,h\right)$ is the maximum.

In the critical XY phase, fidelity temperature $T_{f}\left(\Delta ,h\right)$ diverges from an exterior point of view, indicating strong quantum fluctuations. Fidelity internal energy is, by convention, zero, and the only contribution to fidelity entropy is the residual fidelity entropy $S_{0}$. Since the residual fidelity entropy is extrinsic in nature, it is only determined if the model is embedded into a more general model, such as the quantum XYZ model in a magnetic field (cf. scenario II-2 and scenario III-2 in Section 6), which accommodates a factorizing-field cone surface, on which the ground states are unentangled (factorized) states [77,78,79]. At the boundary between the ${\mathrm{FM}}_{-}$ phase and the XY phase, PT transitions occur when Δ>−1. Hence, fidelity temperature $T_{f}\left(\Delta ,h\right)$ is not well-defined, ranging from 0 to ∞, and fidelity internal energy $U_{f}\left(\Delta ,h\right)$ ranges from 0 to the maximum. In addition, the FM transition point appears to be a multi-critical point, located at (−1, 0). Indeed, fidelity temperature $T_{f}\left(\Delta ,h\right)$ ranges from 0 to ∞, and fidelity internal energy $U_{f}\left(\Delta ,h\right)$ ranges from 0 to the maximum at the FM transition point (cf. Section 9).

In the AF phase, the ground-state wave functions do not depend on *h*. Hence, fidelity entropy $S_{f}\left(\Delta ,h\right)$, fidelity temperature $T_{f}\left(\Delta ,h\right)$, and fidelity internal energy $T_{f}\left(\Delta ,h\right)$ do not depend on *h*. That is, we only need to determine fidelity entropy $S_{f}\left(\Delta ,0\right)$, fidelity temperature $T_{f}\left(\Delta ,0\right)$, and fidelity internal energy $U_{f}\left(\Delta ,0\right)$ on the non-commuting U(1)-symmetric line (h=0 with Δ>1). Note that the symmetry group $Z_{2}^{\sigma }\times Z_{2}^{\tau }/Z_{2}^{\sigma \tau }$ is spontaneously broken on the non-commuting U(1)-symmetric line (h=0 with Δ>1). Here, $Z_{2}^{\tau }$ is generated from a one-site translation, $Z_{2}^{\sigma }$ is generated from the local unitary operation, defined by $\sigma_{i}^{x}↔\sigma_{i}^{y}$ and $\sigma_{i}^{z}↔-\sigma_{i}^{z}$, and $Z_{2}^{\sigma \tau }$ is generated from the combined operation στ. In contrast, only the symmetry group $Z_{2}^{\tau }$ is spontaneously broken, when h≠0. Therefore, the non-commuting U(1)-symmetric line (h=0 with Δ>1) is peculiar in the AF phase: fidelity mechanical-state functions for h=0 with Δ>1 are identical to those for the quantum spin-1/2 XYZ model, when γ=0 with Δ>1, which have been evaluated in Section 9. Hence, we have $S_{f}\left(\Delta ,0\right)=S_{f}^{\mathrm{XYZ}}\left(\Delta ,0\right)$, $T_{f}\left(\Delta ,0\right)=T_{f}^{\mathrm{XYZ}}\left(\Delta ,0\right)$, and $U_{f}\left(\Delta ,0\right)=U_{f}^{\mathrm{XYZ}}\left(\Delta ,0\right)$. We stress that there is an *emergent* symmetry group $Z_{2}$ generated by the time-reversal symmetry operation, and an *emergent* dihedral symmetry group $Z_{2}\times Z_{2}$ in the AF phase if the longitudinal field strength *h* is nonzero. In fact, the time-reversal symmetry and the dihedral symmetry are recovered in the twofold degenerate ground states, although the model Hamiltonian (4) itself is not invariant. This is in sharp contrast to the ${\mathrm{AF}}_{z}$ phase for the quantum spin-1/2 XYZ model, when γ is nonzero. Physically, this results from the fact that the quantum spin-1/2 XXZ model in a magnetic field constitutes a U(1)-symmetric plane and the quantum spin-1/2 XYZ model constitutes a dual plane, when they are embedded into the quantum spin-1/2 XYZ model in a magnetic field. In fact, the entire AF phase is controlled by the KT transition point, located at (1, 0), instead of a line of the IC transition points.

**Figure 21:**
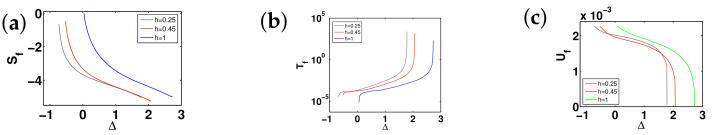
Fidelity entropy $S_{f}\left(\Delta \right)$, fidelity temperature $T_{f}\left(\Delta \right)$, and fidelity internal energy $U_{f}\left(\Delta \right)$ for the quantum spin-1/2 XXZ model in a magnetic field from an interior point of view with respect to the two-dimensional critical XY regime. Here, we have chosen h=0.25, 0.45, and 1. (**a**) Fidelity entropy $S_{f}\left(\Delta \right)$ monotonically increases when Δ varies from the IC transition points (1.80,0.25), (2.08,0.45), and (2.75,1) to the PT transition points (−0.75,0.25), (−0.55,0.45), and (0, 1) and reaches its maximum at the PT transition points: (−0.75,0.25), (−0.55,0.45), and (0, 1), respectively. (**b**) Fidelity temperature $T_{f}\left(\Delta \right)$ diverges at the IC transition points (1.80,0.25), (2.08,0.45), and (2.75,1), but it is zero at the PT transition points (−0.75,0.25), (−0.55,0.45), and (0, 1), respectively. (**c**) Fidelity internal energy $U_{f}\left(\Delta \right)$ monotonically increases from zero to the maximum, when Δ varies from the IC transition points (1.80,0.25), (2.08,0.45), and (2.75,1) to the PT transition points (−0.75,0.25), (−0.55,0.45), and (0, 1), respectively.

Mathematical details about the explicit expressions for fidelity entropy $S_{f}\left(\Delta ,h\right)$, fidelity temperature $T_{f}\left(\Delta ,h\right)$, and fidelity internal energy $U_{f}\left(\Delta ,h\right)$ are presented in Appendix N. We plot fidelity entropy $S_{f}\left(\Delta ,h\right)$, fidelity temperature $T_{f}\left(\Delta ,h\right)$, and fidelity internal energy $U_{f}\left(\Delta ,h\right)$ for h=0.25, 0.45, and 1, from an interior point of view, as a function of Δ with $h-1<\Delta \leq \Delta_{c}$ in Figure 21a–c, respectively.

Now we are ready to discuss the line of the IC transition points—the phase boundary between the XY phase and the AF phase. Along this line, fidelity temperature $T_{f}\left(\Delta_{c},h_{c}\right)$ monotonically decreases from ∞ at $\Delta_{c}=1$ to zero at $\Delta_{c}=\infty$. Therefore, at an IC transition point, $\left(\Delta_{c},h_{c}\right)$, fidelity temperature is not well-defined for $\Delta_{c}>1$, ranging from $T_{f}^{\mathrm{XYZ}}\left(\Delta_{c},0\right)$ to ∞. That is, an IC transition interpolates between the KT transitions and the PT transitions, which represents a new universality class. As pointed out in Section 6, the presence of $\sum \sigma_{i}^{z}$ in the Hamiltonian (4) explicitly breaks the dihedral symmetry group $Z_{2}\times Z_{2}$ and the time-reversal symmetry group $Z_{2}$. Hence, the IC transition may be regarded as a variant of the KT transition when the dihedral symmetry group $Z_{2}\times Z_{2}$ and the time-reversal symmetry group $Z_{2}$ are explicitly broken, similarly to the observation that the PT transition may be regarded as a variant of the FM transition (cf. Section 6).

As follows from our discussion about the quantum spin-1/2 XY model (cf. Section 7), no dominant control parameter *x* is defined on the line of critical points (Δ=0 with −1<h<1). Since this line of critical points results from the level crossings, implying that any two different ground states are orthogonal to each other. The same argument is applicable to any vertical line of critical points, with Δ being fixed, inside the critical XY regime—the two-dimensional critical XY regime (cf. scenario II-2 in Section 6). In contrast, a dominant control parameter *x* is well-defined on a horizontal line of critical points, with *h* being fixed, inside the two-dimensional critical XY regime (cf. scenario III-2 in Section 6). Actually, this choice is consistent with that for the complete line of critical points (cf. scenario III-1) for the quantum spin-1/2 XYZ model, when γ=0. That is, an interior point of view for the two-dimensional critical XY regime, or equivalently, an exterior point of view for the line of the IC transition points, implies that, for any horizontal line, with *h* being fixed to be $h_{c}$, an IC transition point at $\Delta_{c}$ is topological, since fidelity entropy is double-valued, when it is approached from inside the two-dimensional critical XY regime, similarly to the KT transitions. In contrast, an IC transition point at $\Delta_{c}$ and $h_{c}$ is not topological, since fidelity entropy is single-valued when it is approached vertically from inside the AF phase, similarly to the PT transitions. Therefore, fidelity entropy is triple-valued at an IC transition point (cf. Section 6).

We remark that an analogue of the Hawking radiation occurs at the FM transition point and the KT transition point. Indeed, fidelity temperature from an interior point of view is zero at the FM transition point and diverges at the KT transition point, matching that from an exterior point of view, when they are approached along the non-commuting U(1)-symmetric line, as already discussed in Section 9. In contrast, no analogue of the Hawking radiation occurs at the IC transition point, since fidelity temperature is nonzero but finite, when it is approached along the commuting U(1)-symmetric line inside the AF phase (cf. scenario III-2 in Section 6). Indeed, a dominant control parameter *x* in the two-dimensional critical XY regime does not extend to the AF phase, and a dominant control parameter *x* in the AF phase does not extend to the two-dimensional critical XY regime.

## 11. Quantum Spin-1 XYZ Model—A Typical Example for the Symmetry-Protected Topological Phases

**Figure 22:**
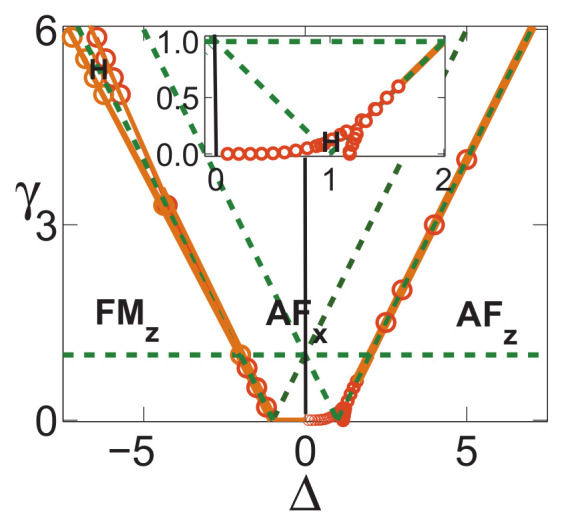
Ground-state phase diagram for the quantum spin-1 XYZ model, with γ≥0. In addition to distinct symmetry-breaking ordered phase, labelled as ${\mathrm{AF}}_{x}$, ${\mathrm{AF}}_{y}$, ${\mathrm{AF}}_{z}$, and ${\mathrm{FM}}_{z}$, the Haldane phase—a typical example for the SPT phases—emerges in the two dual regions, located in the vicinity of the SU(2)-symmetric point (1,0) and its dual image point at infinity. Here, ${\mathrm{AF}}_{x}$, ${\mathrm{AF}}_{y}$, ${\mathrm{AF}}_{z}$, and $F_{z}$ represent an AF phase in the *x* direction, an AF phase in the *y* direction, an AF phase in the *z* direction, and an FM phase in the *z* direction, respectively. Here, we restrict ourselves to the region: γ≥0, due to the symmetry of the Hamiltonian (5) with respect to γ→−γ. The solid lines denote the phase boundaries between the distinct phases. In particular, the FM transition points are located at (−1, 0) and its dual images, and the KT transition points are located at (0, 0) and its dual images. The U(1)-symmetric line (γ=1+Δ with Δ>−1), is a factorizing-field line in addition to other U(1)-symmetric lines: γ=0, γ=−1+Δ, γ=−1−Δ, and γ=1−Δ. Inset: A magnification of the Haldane phase, when 0≤γ≤1.

The model Hamiltonian (5) is in a canonical form, which is not exactly solvable. Hence, we resort to the iTEBD algorithm to map out the ground-state phase diagram, as shown in Figure 22. In addition to the four distinct symmetry-breaking ordered phases ${\mathrm{AF}}_{x}$, ${\mathrm{AF}}_{y}$, ${\mathrm{AF}}_{z}$, and $F_{z}$, the Haldane phase, a typical example for the SPT phases, emerges in the vicinity of the SU(2)-symmetric point (1,0) and its dual image points, as anticipated from the mapping to the nonlinear σ model [65,66]. Here, ${\mathrm{AF}}_{x}$, ${\mathrm{AF}}_{y}$, ${\mathrm{AF}}_{z}$, and $F_{z}$ represent an AF phase in the *x* direction, an AF phase in the *y* direction, an AF phase in the *z* direction, and an FM phase in the *z* direction, respectively.

The Hamiltonian (5) possesses the dihedral symmetry group $Z_{2}\times Z_{2}$, generated from any two of the three π-rotations around the *x*-, *y*- and *z*-axes, e.g., the *x*- and *z*-axes: $S_{i}^{x}↔S_{i}^{x}$, $S_{i}^{y}↔-S_{i}^{y}$ and $S_{i}^{z}↔-S_{i}^{z}$ and $S_{i}^{x}↔-S_{i}^{x}$, $S_{i}^{y}↔-S_{i}^{y}$ and $S_{i}^{z}↔S_{i}^{z}$, respectively. As demonstrated in Appendix C, there are five dualities for the Hamiltonian (5) arising from the symmetric group $S_{3}$ with respect to *x*, *y*, and *z*, thus leading to five symmetric or self-dual lines that are identical to five U(1)-symmetric lines in addition to three semi-self-dual lines located at γ=±1 and Δ=0. In addition to γ=0, there are four U(1)-symmetric lines, which are located at γ=1−Δ, γ=1+Δ, γ=−1+Δ, and γ=−1−Δ. Meanwhile, a U(1) symmetry occurs when |Δ| or |γ| is infinite in value. Furthermore, a SU(2) symmetry emerges at the two characteristic points (±1,0) and their dual image points.

The SU(2)-symmetric point, located at (1,0), is adiabatically connected to the Affleck–Kennedy–Lieb–Tasaki (AKLT) model, which admits an exact solution in terms of the valence bond solid [133,134]. Note that the AKLT model is a special case of the spin-1 bilinear–biquadratic model (also cf. Appendix O), which itself attracts much attention [135,136,137,138,139,140,141,142,143,144,145,146,147,148,149,150,151,152,153,154,155,156,157,158]. In addition, the so-called string order parameter [159] is introduced to characterize this exotic phase—a typical example for the SPT phases [108,109,110,111]. A recent development in characterizing an SPT phase is to exploit the so-called bond-centered non-local order parameter [112] (also cf. Appendix J). It is found that there is a hidden $Z_{2}\times Z_{2}$ symmetry-breaking order in the Haldane phase [160,161], which results in a four-fold degenerate ground states after a non-local unitary transformation is performed.

The Hamiltonian (5) is symmetrical with respect to γ↔−γ. Therefore, we may restrict ourselves to the region γ≥0. Taking into account the symmetries, dualities, factorizing fields [77,78,79] and a soft line, we may divide the entire region γ≥0 into twenty-four different regimes. The twenty-four regimes fall into four groups, with six regimes in each group being dual to each other (cf. Section 2): regime I, regime V, regime IX, regime XIII, regime XVII, and regime XXI are dual to each other; regime II, regime VI, regime X, regime XIV, regime XVIII, and regime XXII are dual to each other; regime III, regime VII, regime XI, regime XV, regime XIX, and regime XXIII are dual to each other, whereas regime IV, regime VIII, regime XII, regime XVI, regime XX, and regime XXIV are dual to each other. Therefore, there are only four principal regimes representing the physics underlying the quantum spin-1 XYZ model.

We chose regime I ($0<\Delta <\Delta_{c0}$ and 0<γ<1), regime II (−1<Δ<0 and 0<γ<1+Δ), regime III (0<Δ<1 and $\Delta_{c1}<\gamma <1-\Delta_{c0}$), and regime IV ($0<\Delta <\Delta_{c1}$ and $0<\gamma <1-\Delta_{c1}$) as the four principal regimes. Here, $\Delta_{c1}$ denotes the critical value of Δ for a KT transition from the critical XY phase to the Haldane phase on the U(1)-symmetric line (γ=0), as follows from our numerical simulation in terms of the iTEBD algorithm. For the bond dimension χ=60, we have $\Delta_{c0}\approx 0.915$ and $\Delta_{c1}\approx 0.29$. Note that, on the U(1)-symmetric line (γ=0), the KT transition point shifts towards (0,0) as the bond dimension χ increases. In fact, when χ=300, $\Delta_{c1}\approx 0.11$. However, there is no compelling evidence to demonstrate that $\Delta_{c1}$ becomes zero exactly. This observation is consistent with [162,163]. A point to be mentioned is that if $\Delta_{c1}$ becomes zero exactly, then the principal regime IV becomes a numerical artifact arising from the finiteness of the bond dimension χ. Here, we stress that a regime arising from the finiteness of the bond dimension χ, as a numerical artifact, is quite common as a result of the tensor network simulations [158]. In any case, whether or not principal regime IV arises from the finiteness of the bond dimension χ does not affect our discussion about fidelity mechanical-state functions.

A choice of a dominant control parameter *x* and an auxiliary control parameter τ has been made for each of the four principal regimes (cf. Section 2). In regime I, a dominant control parameter *x* was chosen to be $x=\Delta_{c}/\left(2-\Delta_{c}\right)-\Delta /\left(2-\Delta \right)$, starting from the point $\left(\Delta_{c},\tau -\Delta_{c}\right)$, up to (0,τ), and an auxiliary control parameter τ was chosen to be τ=γ+Δ, ranging from $\Delta_{c1}$ to 1. Here, a re-parametrization operation in the ground-state energy density e(Δ,τ−Δ): $e\left(\Delta ,\gamma \right)=m^{I}\left(x,\tau \right)e^{I}\left(x,\tau \right)$, with $m^{I}\left(x,\tau \right)=\left(\Delta_{c}/\left(2-\Delta_{c}\right)+1\right)/\left(x+1\right)$, is performed to ensure that $e^{I}\left(x,\tau \right)$ monotonically decreases with *x*. Here, ($\Delta_{c},\tau -\Delta_{c}$) denotes the Ising transition point between the Haldane phase and the ${\mathrm{AF}}_{x}$ phase, which is located on the straight line (γ=τ−Δ) for a fixed $\tau \in [\Delta_{c1},1]$. In regime II, a dominant control parameter *x* was chosen to be x=γ, starting from γ=0 up to γ=1+Δ, and an auxiliary control parameter τ was chosen to be τ=Δ∈(−1,0). In regime III, a dominant control parameter *x* was chosen to be $x=\Delta /\left(2-\Delta \right)-\Delta_{c}/\left(2-\Delta_{c}\right)$, starting from $\left(\Delta_{c},\tau -\Delta_{c}\right)$ up to (τ,0), and an auxiliary control parameter τ was chosen to be τ=γ+Δ, ranging from $\Delta_{c1}$ to 1. In regime IV, a dominant control parameter *x* was chosen to be x=(γ+Δ)/(2−γ−Δ)−Δ/(2−Δ) and an auxiliary control parameter τ was chosen to be τ=γ+Δ. A Gaussian transition occurs at $x_{c}=0$, for a fixed τ. Here, a re-parametrization operation in the ground-state energy density e(Δ,γ): $e\left(\Delta ,\gamma \right)=m^{\mathrm{IV}}\left(x,\tau \right)e^{\mathrm{IV}}\left(x,\tau \right)$, with $m^{\mathrm{IV}}\left(x,\tau \right)=\left(\tau /\left(2-\tau \right)+1\right)/\left(x+1\right)$, is performed to ensure that $m^{\mathrm{IV}}\left(x,\tau \right)$ and $e^{\mathrm{IV}}\left(x,\tau \right)$ monotonically decreases with *x*, for a fixed $\tau \in (0,\Delta_{c1}]$. However, other choices are possible, as long as such a choice is consistent with the constraints imposed by the symmetries, dualities, and factorizing fields (cf. Section 4).

It is numerically confirmed that fidelity entropy S(Δ,γ) scales as $\gamma^{\nu (\Delta )+1}$ near the line of the Gaussian critical points (γ=0 with $-1<\Delta \leq \Delta_{c1}$). Here, ν(Δ) is the critical exponent for the correlation length, which varies with Δ. This is consistent with the fact that the transition belongs to the Gaussian universality class (cf. Appendix H). In addition, fidelity entropy S(x,τ) scales as $x^{2}$ near the line of critical points between the Haldane phase and the ${\mathrm{AF}}_{x}$ phase, indicating that the critical exponent ν is ν=1, consistent with the fact that the transition belongs to the Ising universality class (cf. Appendix H).

If a dominant control parameter *x* and an auxiliary control parameter τ are chosen in regime I, regime II, regime III and regime IV, then fidelity entropy $S_{f}\left(\Delta ,\gamma \right)$ may be determined straightforwardly in the four principal regimes as well as on the characteristic lines, which appear as the boundaries between the four principal regimes or their dual image regimes. Accordingly, fidelity temperature $T_{f}\left(\Delta ,\gamma \right)$ and fidelity internal energy $U_{f}\left(\Delta ,\gamma \right)$ are determined by solving a singular first-order differential equation for V(x,τ). Then, fidelity mechanical-state functions in the non-principal regimes, which are dual image regimes, simply follow from their respective dualities to regime I, regime II, regime III, and regime IV. However, the ground-state energy density e(Δ,γ) is not monotonic along the characteristic line (γ=1−Δ with $\Delta_{c0}\leq \Delta \leq 1$), which is a principal part. In the principal part (γ=1−Δ with $\Delta_{c0}\leq \Delta \leq 1$), a dominant control parameter *x* is chosen to be $x=\Delta_{c0}/\left(2-\Delta_{c0}\right)-\Delta /\left(2-\Delta \right)$, starting from the transition point $\left(\Delta_{c0},\;1-\Delta_{c0}\right)$ up to the U(1)-symmetric point (0,1). Here, a re-parametrization operation in the ground-state energy density e(Δ,1−Δ): $e\left(\Delta ,1-\Delta \right)=m^{\mathrm{ii}}\left(x\right)e^{\mathrm{ii}}\left(x\right)$, with $m^{\mathrm{ii}}\left(x\right)=\left(\Delta_{c0}/\left(2-\Delta_{c0}\right)+1\right)/\left(x+1\right)$, is performed to ensure that $e^{\mathrm{ii}}\left(x\right)$ is monotonic with *x*. In addition, there is an alternative choice for a re-parametrization operation in the ground-state energy density e(Δ,1−Δ): $e\left(\Delta ,1-\Delta \right)=m_{A}^{\mathrm{ii}}\left(x\right)e_{A}^{\mathrm{ii}}\left(x\right)$, with $m_{A}^{\mathrm{ii}}\left(x\right)=\left(2-\Delta_{c0}\right)/\left(2-2x+\Delta_{c0}x\right)$. Note that $\Delta_{c0}$ denotes the Ising transition point between the Haldane phase and the ${\mathrm{AF}}_{x}$ phase on the U(1)-symmetric line (γ=1−Δ with 0<Δ<1).

The explicit expressions for fidelity entropy $S_{f}\left(\Delta ,\gamma \right)$, fidelity temperature $T_{f}\left(\Delta ,\gamma \right)$, and fidelity internal energy $U_{f}\left(\Delta ,\gamma \right)$ may be derived, following from our prescription in Section 2 (also cf. scenario III-1 in Section 6). The details are presented in Appendix O.

**Figure 23:**
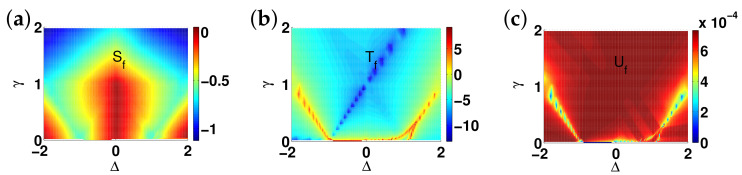
Fidelity entropy $S_{f}\left(\Delta ,\gamma \right)$, fidelity temperature $T_{f}\left(\Delta ,\gamma \right)$, and fidelity internal energy $U_{f}\left(\Delta ,\gamma \right)$ for the quantum spin-1 XYZ model, with γ≥0. (**a**) Fidelity entropy $S_{f}\left(\Delta ,\gamma \right)$ exhibits a local maximum at a stable fixed point (1,0). Singularities occur on the three lines of the Gaussian critical points: γ=0 with $-1<\Delta \leq \Delta_{c1}$ and its dual images, with central charge c=1, and on the lines of the Ising critical points in addition to the factorizing-field line: γ=1+Δ with Δ>−1 and the two U(1)-symmetric lines: γ=1−Δ with Δ≤1 and γ=1. (**b**) Fidelity temperature $T_{f}\left(\Delta ,\gamma \right)$ diverges at the three lines of the Gaussian critical points: γ=0 with $-1<\Delta \leq \Delta_{c1}$ and its dual images, with central charge c=1 and at the lines of the Ising critical points. It is zero on the factorizing-field line: γ=1+Δ with Δ>−1 and at the factorizing fields, when |Δ| is infinite in value. (**c**) Fidelity internal energy $U_{f}\left(\Delta ,\gamma \right)$ takes the maximum value at all stable fixed point; (0,1), (±∞,0), and (±∞,1); on the factorizing-field line: γ=1+Δ with Δ>−1; and at the factorizing fields, when |Δ| is infinite in value. Here, $\Delta_{c1}\approx 0.29$ and $\Delta_{c2}\approx 1.55$ follow from our numerical simulation by means of the iTEBD algorithm, with the bond dimension χ=60.

A contour plot is depicted in Figure 23a–c for fidelity entropy $S_{f}\left(\Delta ,\gamma \right)$, fidelity temperature $T_{f}\left(\Delta ,\gamma \right)$, and fidelity internal energy $U_{f}\left(\Delta ,\gamma \right)$ in the control parameter space for γ≥0. Here, a contribution to fidelity entropy from rescaling in the ground-state energy density, due to a re-parametrization operation and dualities, has been taken into account (cf. Appendix G). Fidelity entropy $S_{f}\left(\Delta ,\gamma \right)$ takes a local maximum at the U(1)-symmetric point (0,1) and its dual images. Fidelity temperature $T_{f}\left(\Delta ,\gamma \right)$ diverges at the lines of the Gaussian critical points (γ=0 with $-1<\Delta \leq \Delta_{c1}$), and its dual images and the lines of the Ising critical points, which appear as the phase boundaries in the Haldane phases, and vanishes on the factorizing-field line (γ=1+Δ with Δ>−1), in addition to the two characteristic lines at infinity—Δ=±∞. We remark that there are seven stable fixed points, identified as the characteristic points in the region γ≥0; the U(1)-symmetric point: (0,1); and its dual images and one metastable fixed point at the SU(2)-symmetric point (1,0) and its dual images. Fidelity entropy takes double values on the non-commuting U(1)-symmetric line (γ=0) in the Haldane phase, reflecting the fact that the Haldane phase is topological in the control parameter space (cf. scenario I-2 and the first type of cycle in Section 6).

Fidelity entropy $S_{f}\left(\Delta ,\gamma \right)$, fidelity temperature $T_{f}\left(\Delta ,\gamma \right)$, and fidelity internal energy $U_{f}\left(\Delta ,\gamma \right)$ exhibit singular behaviors on the lines of the Gaussian critical points (γ=0 with $-1<\Delta \leq \Delta_{c1}$) and its dual images at the phase boundaries in the Haldane phases and on the U(1)-symmetric line (γ=1+Δ with Δ>−1), which is also a factorizing-field line and on the two U(1)-symmetric lines: γ=1−Δ with Δ≤1 and γ=1, which are self-dual. This singular behavior may be recognized as “phase transitions” in fidelity mechanics. In addition, fidelity internal energy $U_{f}\left(\Delta ,\gamma \right)$ takes the same maximum value at all stable fixed points, as well as on the factorizing-field line. Note that at the FM transition point (−1, 0), fidelity temperature $T_{f}$ and fidelity internal energy $U_{f}$ are not well-defined; fidelity temperature $T_{f}$ ranges from 0 to infinity and fidelity internal energy $U_{f}$ ranges from 0 to the maximum depending on how the FM transition point (−1, 0) is approached. This is exactly the same as the FM transition point for the quantum spin-1/2 XYZ model. In fact, the Hamiltonian (5) at this transition point admits highly degenerate and highly entangled ground states, which arise from SSB with one type-B GM when SU(2) is spontaneously broken to U(1) [102]. As it turns out, the ground states are scale-invariant, with the fractal dimension $d_{f}$ being identified as the number $N_{B}$ of type-B GMs: $d_{f}=N_{B}=1$ (also cf. Appendix I).

We turn to fidelity mechanical-state functions on the three lines of critical points from an interior point of view: γ=0 with $-1<\Delta \leq \Delta_{c1}$ and its dual images. Given they are dual relative to each other, we only need to consider the line of critical points (γ=0 with $-1<\Delta \leq \Delta_{c1}$). This is a complete line of critical points, labelled as scenario III-1 in Section 6. Since no characteristic point exists between the two endpoints, there is only one principal part (cf. Section 9). Once a dominant control parameter *x* is chosen in this principal part, evaluating fidelity entropy $S_{f}\left(\Delta ,0\right)$, as a function of Δ, with $-1<\Delta \leq \Delta_{c1}$, is straightforward. Then, fidelity temperature $T_{f}\left(\Delta ,0\right)$ and fidelity internal energy $U_{f}\left(\Delta ,0\right)$ follow from solving a singular first-order differential equation for V(x). Mathematical details about their explicit expressions are presented in Appendix O.

**Figure 24:**
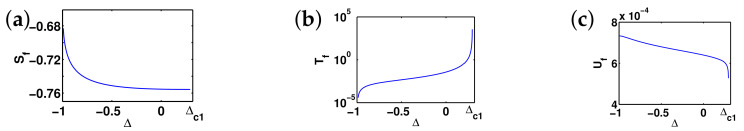
Fidelity entropy $S_{f}\left(\Delta \right)$, fidelity temperature $T_{f}\left(\Delta \right)$, and fidelity internal energy $U_{f}\left(\Delta \right)$ on the complete line of the Gaussian critical points (γ=0 with $-1<\Delta <\Delta_{c1}$) for the quantum spin-1 XYZ model. (**a**) Fidelity entropy monotonically increases from $\Delta =\Delta_{c1}$ to Δ=−1 and reaches its maximum at the FM transition point (−1, 0). (**b**) Fidelity temperature $T_{f}\left(\Delta \right)$ diverges at the KT transition point ($\Delta_{c1}$, 0), but it is zero at the FM transition point (−1, 0). (**c**) Fidelity internal energy $U_{f}\left(\Delta \right)$ monotonically increases and reaches its maximum at the FM transition point (−1, 0). Fidelity entropy $S_{f}\left(\Delta \right)$, fidelity temperature $T_{f}\left(\Delta \right)$, and fidelity internal energy $U_{f}\left(\Delta \right)$ at the FM transition point (−1, 0) are identified as those from an exterior point of view. Here, $\Delta_{c1}\approx 0.29$ follow from our numerical simulation by means of the iTEBD algorithm, with the bond dimension χ=60.

We plot fidelity entropy $S_{f}\left(\Delta ,0\right)$, fidelity temperature $T_{f}\left(\Delta ,0\right)$, and fidelity internal energy $U_{f}\left(\Delta ,0\right)$ as a function of Δ, with $-1<\Delta \leq \Delta_{c1}$, in Figure 24a–c, respectively, from an interior point of view. (a) Fidelity entropy $S_{f}\left(\Delta ,0\right)$ monotonically increases when Δ varies from $\Delta =\Delta_{c1}$ to Δ=−1 and reaches its maximum at the FM transition point (−1, 0). (b) Fidelity temperature $T_{f}\left(\Delta ,0\right)$ diverges at the KT transition point (Δ_*c*1_, 0), but it is zero at the FM transition point (−1, 0). (c) Fidelity internal energy $U_{f}\left(\Delta ,0\right)$ monotonically increases when Δ varies from $\Delta =\Delta_{c1}$ to Δ=−1 and reaches its maximum at the FM transition point (−1, 0).

In addition, we stress that, according to our prescription, shifts in fidelity temperature and fidelity internal energy are performed to ensure that $T_{f}\left(\Delta ,0\right)$ is zero and $U_{f}\left(\Delta ,0\right)$ is the maximum at the FM transition point (−1, 0). This demands the existence of a nonlocal unitary transformation that turns a highly entangled ground-state wave function at this transition point into an unentangled (factorized) state, as discussed in Section 6. Physically, this amounts to stating that ground-state wave functions at all stable fixed points are unitarily equivalent. Meanwhile, at the FM transition point, a singularity arises from the fact that the two limiting procedures do not commute [107]: one is the thermodynamic limit N→∞ and the other is the limiting procedure representing how such a transition point is approached along the complete line of critical points.

In principle, one may carry out a detailed analysis of fidelity mechanical-state functions on the cycle, i.e., the boundaries enclosing the Haldane phases, as shown in Figure 10a. However, our numerical simulation is not accurate enough to locate phase boundaries, which makes it less reliable when evaluating fidelity entropy. Instead, a heuristic physical argument implies that fidelity entropy is singled-valued at the QPT point $\left(\Delta_{c1},0\right)$, labelled as KT, and its dual images. Meanwhile, fidelity mechanical-state functions from an interior point of view do not match those from an exterior point of view (with respect to the complete line of critical points) for any interior point (cf. scenario I-2 in Section 6).

The single-valuedness of fidelity entropy at the FM transition point implies that it is not topological, consistent with the conventional characterization. In contrast, the double-valuedness of fidelity entropy at the KT transition point implies that it is topological in our characterization (cf. scenario III-1 in Section 6). We remark that an analogue of the Hawking radiation occurs at the FM transition point and the KT transition point, according to the criterion in Section 6. Indeed, fidelity temperature from an interior point of view is zero at the FM transition point and diverges at the KT transition point, matching those from an exterior point of view, when they are approached along the non-commuting U(1)-symmetric line.

## 12. The Spin-1/2 Kitaev Model on a Honeycomb Lattice—A Typical Example for Topologically Ordered States

**Figure 25:**
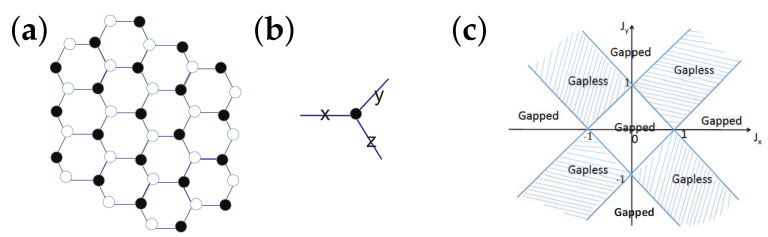
(**a**) The structure of the spin-1/2 Kitaev model on a honeycomb lattice, with filled and unfilled circles indicating two sublattices. (**b**) The interactions on the three distinct types of the bonds: *x*-type, *y*-type, and *z*-type. (**c**) Ground-state phase diagram for the spin-1/2 Kitaev model on a honeycomb lattice. It consists of nine distinct phases: five gapped $Z_{2}$ quantum spin liquid phases and four gapless $Z_{2}$ quantum spin liquid phases.

The model Hamiltonian (6) is in a canonical form. The structure of the spin-1/2 Kitaev model on a honeycomb lattice, with filled and unfilled circles indicating two sublattices, and the interactions on the three distinct types of the bonds, *x*-type, *y*-type, and *z*-type, are shown in Figure 25a,b. Its simplicity makes it likely to be the first model in which topologically ordered states are realized on an optical lattice [164].

Set $J_{z}=1$; we may use $H(J_{x},J_{y})$ to denote the Hamiltonian for brevity. The Hamiltonian (6) is exactly solvable [31,33]. This allows us to map out the ground-state phase diagram, as shown in Figure 25c. We note that the Hamiltonian (6) consists of four distinct phases, three gapped $Z_{2}$ quantum spin liquid phases and one gapless $Z_{2}$ quantum spin liquid phase, if we restrict ourselves to the following region: $J_{x}\geq 0$ and $J_{y}\geq 0$ [31]. For the gapless $Z_{2}$ quantum spin liquid, its ground state is spin-disordered and supports the emergent gapless excitations represented by Majorana fermions [31], whereas for the gapped $Z_{2}$ quantum spin liquid, spin correlations are short-ranged and confined to the nearest-neighbor pairs [32,33].

The Hamiltonian $H(J_{x},J_{y})$ is symmetrical with respect to $J_{x}↔J_{y}$ under a local unitary transformation, $\sigma_{i}^{x}↔\sigma_{i}^{y}$ and $\sigma_{i}^{z}↔-\sigma_{i}^{z}$, with two additional dualities arising from the symmetric group $S_{3}$ with respect to *x*, *y*, and *z*. It possesses the dihedral symmetry group $Z_{2}\times Z_{2}$, generated from any two of the three π-rotations around the *x*-, *y*- and *z*-axes, e.g., the *x*- and *z*-axes: $\sigma_{i}^{x}↔\sigma_{i}^{x}$, $\sigma_{i}^{y}↔-\sigma_{i}^{y}$ and $\sigma_{i}^{z}↔-\sigma_{i}^{z}$ and $\sigma_{i}^{x}↔-\sigma_{i}^{x}$, $\sigma_{i}^{y}↔-\sigma_{i}^{y}$ and $\sigma_{i}^{z}↔\sigma_{i}^{z}$, respectively.

The symmetries and dualities are discussed in detail for the region, defined by $J_{x}\geq 0$ and $J_{y}\geq 0$, in Appendix C. Taking the symmetries and dualities into account, we may divide the region, defined by $J_{x}\geq 0$ and $J_{y}\geq 0$, into twelve distinct regimes (cf. Section 2), with the $Z_{2}$-symmetric line ($J_{x}=J_{y}$) and the two self-dual lines being located at $J_{x}=1$ and $J_{y}=1$, respectively, together with the three lines of critical points ($J_{y}=1-J_{x}$ with $0\leq J_{x}\leq 1$, $J_{y}=1+J_{x}$ with $J_{x}\geq 0$, and $J_{y}=-1+J_{x}$ with $J_{x}\geq 1$) [165,166]. The twelve regimes are separated into two groups, with six regimes in each group being dual to each other. As shown in Section 2, the first group includes regime I, regime III, regime V, regime VII, regime IX, and regime XI, whereas the second group includes regime II, regime IV, regime VI, regime VIII, regime X, and regime XII. Therefore, there are only two principal regimes representing the physics underlying the spin-1/2 Kitaev model on a honeycomb lattice. Here, we chose regime I ($0<J_{x}<1/2$ and $J_{x}<J_{y}<1-J_{x}$) and regime II ($0<J_{x}<1$, $1-J_{x}<J_{y}<1$ and $J_{y}>J_{x}$) as the two principal regimes. Then, all the other regimes are symmetric or dual image regimes. In addition, the characteristic points, as an intersection of any two characteristic lines, are identified to be located at the $S_{3}$-symmetric point (1,1) and at the U(1)-symmetric point (0,0) and its dual image points.

A choice of a dominant control parameter *x* and an auxiliary control parameter τ has been made for each of the two principal regimes (cf. Section 2). In regime I, a dominant control parameter *x* was chosen to be $x=\sqrt{J_{x}^{2}+J_{y}^{2}}\left(1-J_{x}-J_{y}\right)/\left(J_{x}+J_{y}\right)$, starting from the critical point $\left(J_{x}/\left(J_{x}+J_{y}\right),J_{y}/\left(J_{x}+J_{y}\right)\right)$ up to the U(1)-symmetric point (0,0), and an auxiliary control parameter τ was chosen to be $\tau =J_{y}/J_{x}\in \left(1,\infty \right)$. Here, a re-parametrization operation in the ground-state energy density $e(J_{x},J_{y})$ is performed, which turns out to be trivial: $e\left(J_{x},J_{y}\right)=e^{I}\left(x,\tau \right)$. In regime II, a dominant control parameter *x* was chosen to be $x=\sqrt{{\left(J_{x}-1\right)}^{2}+{\left(J_{y}-1\right)}^{2}}\left(J_{x}+J_{y}-1\right)/\left(2-J_{x}-J_{y}\right)$, starting from the critical point $\left(\left(J_{y}-1\right)/\left(J_{x}+J_{y}-2\right),\left(J_{x}-1\right)/\left(J_{x}+J_{y}-2\right)\right)$ up to the $S_{3}$-symmetric point (1,1), and an auxiliary control parameter τ was chosen to be $\tau =\left(J_{y}-1\right)/\left(J_{x}-1\right)\in \left(0,1\right)$. Here, a re-parametrization operation in the ground-state energy density $e(J_{x},J_{y})$ is performed, which turns out to be trivial: $e\left(J_{x},J_{y}\right)=e^{\mathrm{II}}\left(x,\tau \right)$.

It is numerically confirmed that fidelity entropy S(x,τ) scales as $x^{5/2}$ near the line of critical points: $J_{y}=1-J_{x}$ with $0\leq J_{x}\leq 1$, consistent with the fact that d=2, m=1, $\nu_{\|}=1/2$, and $\nu_{⊥}=1$. Here, $\nu_{\|}$ and $\nu_{⊥}$ stand for the critical exponent for the correlation length in two perpendicular directions, with *m* and d−m being the effective dimensions, respectively (cf. Appendix H).

Once a dominant control parameter *x* and an auxiliary control parameter τ are chosen in regime I and regime II, evaluating fidelity entropy $S_{f}\left(J_{x},J_{y}\right)$, fidelity temperature $T_{f}\left(J_{x},J_{y}\right)$, and fidelity internal energy $U_{f}\left(J_{x},J_{y}\right)$ is straightforward in each principal regime. Mathematical details for their explicit expressions are demonstrated in Appendix P. In addition, fidelity entropy $S_{f}\left(J_{x},J_{y}\right)$, fidelity temperature $T_{f}\left(J_{x},J_{y}\right)$, and fidelity internal energy $U_{f}\left(J_{x},J_{y}\right)$ in the symmetric or dual image regimes simply follow from their respective symmetric or duality transformations to regime I and regime II, respectively.

**Figure 26:**
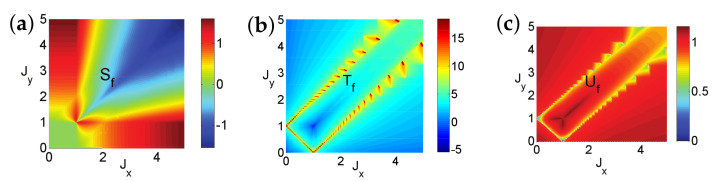
Fidelity entropy $S_{f}\left(J_{x},J_{y}\right)$, fidelity temperature $T_{f}\left(J_{x},J_{y}\right)$ and fidelity internal energy $U_{f}\left(J_{x},J_{y}\right)$ for the spin-1/2 Kitaev model on a honeycomb lattice in the region $J_{x}\geq 0$ and $J_{y}\geq 0$. (**a**) Fidelity entropy $S_{f}\left(J_{x},J_{y}\right)$ takes a local maximum at the U(1)-symmetric point (0,0) and the $S_{3}$-symmetric point (1,1). Singularities occur on the three lines of critical points ($J_{y}=1-J_{x}$ with $0\leq J_{x}\leq 1$, $J_{y}=1+J_{x}$ with $J_{x}\geq 0$, and $J_{y}=-1+J_{x}$ with $J_{x}>1$), and on the $Z_{2}$-symmetric lines, which are also self-dual: $J_{x}=1$, $J_{y}=1$, and $J_{x}=J_{y}$. QPTs on the three lines of critical points, apart from (0,1) and its dual images, are topological, reflected in the fact that fidelity entropy is double-valued. (**b**) Fidelity temperature $T_{f}\left(J_{x},J_{y}\right)$ diverges at the three lines of critical points ($J_{y}=1-J_{x}$ with $0\leq J_{x}\leq 1$, $J_{y}=1+J_{x}$ with $J_{x}\geq 0$, and $J_{y}=-1+J_{x}$ with $J_{x}>0$), and is zero at the U(1)-symmetric point (0,0) and the $S_{3}$-symmetric point (1,1) and their symmetric or dual image points. (**c**) Fidelity internal energy $U_{f}\left(J_{x},J_{y}\right)$ takes the maximum value at the $S_{3}$-symmetric point (1,1) and the U(1)-symmetric point (0,0) and its dual image points.

A contour plot is depicted in Figure 26a–c for fidelity entropy $S_{f}\left(J_{x},J_{y}\right)$, fidelity temperature $T_{f}\left(J_{x},J_{y}\right)$, and fidelity internal energy $U_{f}\left(J_{x},J_{y}\right)$ in the control parameter space, with $J_{x}\geq 0$ and $J_{y}\geq 0$, respectively. Here, a contribution to fidelity entropy from rescaling in the ground-state energy density, due to dualities, has been taken into account (cf. Appendix G). Fidelity entropy $S_{f}\left(J_{x},J_{y}\right)$ takes a local maximum at the $S_{3}$-symmetric point (1,1) and at the U(1)-symmetric point (0,0) and its dual image points; fidelity temperature $T_{f}\left(J_{x},J_{y}\right)$ diverges at the three lines of critical points ($J_{y}=1-J_{x}$ with $0\leq J_{x}\leq 1$, $J_{y}=1+J_{x}$ with $J_{x}\geq 0$, and $J_{y}=-1+J_{x}$ with $J_{x}\geq 1$), and vanishes at the U(1)-symmetric point (0,0) and its dual image points, and at the $S_{3}$-symmetric point (1,1). The four characteristic (symmetric) points are identified as four stable fixed points in the region, defined by $J_{x}\geq 0$ and $J_{y}\geq 0$, at which fidelity internal energy $U_{f}\left(J_{x},J_{y}\right)$ takes the same maximum value. Here, a non-local unitary transformation is needed, which is performed to remove entanglement from the ground-state wave function at the $S_{3}$ symmetric point (1,1) or at the U(1) symmetric point (0,0) and its dual image points, in order to turn it into an unentangled (factorized) state. In our choice, fidelity entropy $S_{f}\left(J_{x},J_{y}\right)$ at the U(1)-symmetric point (0,0) is single-valued; then, it is multiple-valued at the $S_{3}$-symmetric point (1,1). However, there is an alternative choice: fidelity entropy $S_{f}\left(J_{x},J_{y}\right)$ at the $S_{3}$-symmetric point (1,1) is single-valued; then, it is multiple-valued at the U(1)-symmetric point (0,0). Therefore, both the gapped and gapless spin liquid phases are topological, consistent with the emergence of the two cycles, as shown in Figure 10b,c, respectively (cf. scenario III-3 in Section 6).

In addition, fidelity entropy $S_{f}\left(J_{x},J_{y}\right)$, fidelity temperature $T_{f}\left(J_{x},J_{y}\right)$, and fidelity internal energy $U_{f}\left(J_{x},J_{y}\right)$ exhibit singular behaviors on the six lines, defined by $J_{x}=1$, $J_{y}=1$, $J_{y}=J_{x}$, $J_{y}=1-J_{x}$, and $J_{y}=\pm 1+J_{x}$, among which three are the lines of critical points ($J_{y}=1-J_{x}$ with $0\leq J_{x}\leq 1$, $J_{y}=1+J_{x}$ with $J_{x}\geq 0$, and $J_{y}=-1+J_{x}$ with $J_{x}>1$) and the other three lines ($J_{x}=1$, $J_{y}=1$, and $J_{y}=J_{x}$) represent self-dual lines. This singular behavior may be recognized as “phase transitions” in fidelity mechanics.

Note that our discussion about fidelity mechanical-state functions in the gapless spin liquid phase may be regarded as an exterior point of view with respect to the complete lines of critical points, located on the boundary between the gapped and gapless spin liquid phases or an interior point of view with respect to the two-dimensional gapless phase (cf. Section 6).

**Figure 27:**
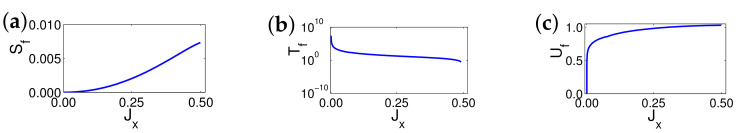
Fidelity entropy $S_{f}\left(J_{x}\right)$, fidelity temperature $T_{f}\left(J_{x}\right)$, and fidelity internal energy $U_{f}\left(J_{x}\right)$ as a function of $J_{x}$, for the spin-1/2 Kitaev model on a honeycomb lattice on the line of critical points: $J_{y}=1-J_{x}$ with $0<J_{x}<1/2$. (**a**) Fidelity entropy monotonically increases when *x* varies from $J_{x}=0$ to $J_{x}=1/2$, and it reaches its maximum at the $Z_{2}$-symmetric point: $J_{x}=J_{y}=1/2$. (**b**) Fidelity temperature $T_{f}\left(J_{x}\right)$ diverges at $J_{x}=0$, representing the QPT point (0,1), but it is zero at the $Z_{2}$-symmetric point: $J_{x}=J_{y}=1/2$. (**c**) Fidelity internal energy $U_{f}\left(J_{x}\right)$ monotonically increases when $J_{x}$ varies from $J_{x}=0$ to $J_{x}=1/2$, and it reaches its maximum at the $Z_{2}$-symmetric point: $J_{x}=J_{y}=1/2$.

The explicit expressions for fidelity entropy $S_{f}\left(J_{x},1-J_{x}\right)$, fidelity temperature $T_{f}\left(J_{x},1-J_{x}\right)$, and fidelity internal energy $U_{f}\left(J_{x},1-J_{x}\right)$ on the line of critical points ($J_{y}=1-J_{x}$ with $0\leq J_{x}\leq 1/2$) from an interior point of view are presented in Appendix P. We plot fidelity entropy $S_{f}\left(J_{x},1-J_{x}\right)$, fidelity temperature $T_{f}\left(J_{x},1-J_{x}\right)$, and fidelity internal energy $U_{f}\left(J_{x},1-J_{x}\right)$ as a function of $J_{x}$, with $0\leq J_{x}\leq 1/2$, in Figure 27a–c, respectively. (a) Fidelity entropy $S_{f}\left(J_{x},1-J_{x}\right)$ monotonically increases when *x* varies from $J_{x}=0$ to $J_{x}=1/2$ and reaches its maximum at the $Z_{2}$-symmetric point (1/2, 1/2). (b) Fidelity temperature $T_{f}\left(J_{x},1-J_{x}\right)$ diverges at $J_{x}=0$, representing the QPT point (0,1), but it is zero at the $Z_{2}$-symmetric point (1/2, 1/2). (c) Fidelity internal energy $U_{f}\left(J_{x},1-J_{x}\right)$ monotonically increases when $J_{x}$ varies from $J_{x}=0$ to $J_{x}=1/2$, and it reaches its maximum at the $Z_{2}$-symmetric point (1/2, 1/2).

Fidelity entropy $S_{f}\left(J_{x},J_{y}\right)$ is double-valued on the three lines of critical points ($J_{y}=1-J_{x}$ with $0\leq J_{x}\leq 1$, $J_{y}=1+J_{x}$ with $J_{x}\geq 0$, and $J_{y}=-1+J_{x}$ with $J_{x}\geq 1$), apart from (0,1) and its dual images (cf. scenario III-3 in Section 6). The double-valuedness of fidelity entropy implies that the TPT transitions are topological in the control parameter space, consistent with the conventional classification [165,166]. Here, we remark that more extensive numerical simulations are necessary to evaluate fidelity mechanical-state functions from an exterior point of view with respect to the complete line of critical points in scenario III-3. For this purpose, introducing an extra coupling parameter is necessary, such as an external magnetic field along the [111]-axis [100,101]. The presence of this term explicitly breaks the dihedral symmetry group $Z_{2}\times Z_{2}$ and the time-reversal symmetry group $Z_{2}$. As a consequence, a TPT transition point is turned into its variant when the dihedral symmetry group $Z_{2}\times Z_{2}$ and the symmetry group $Z_{2}$ are explicitly broken. Indeed, two successive QPTs at finite field strengths are unveiled for the AF Kitaev exchange on the $S_{3}$-symmetric line in Ref. [101].

We remark that an analogue of the Hawking radiation occurs at the TPT transition point. Indeed, fidelity temperature from an interior point of view, which diverges at the TPT transition point, matches that from an exterior point of view, when it is approached along the $Z_{2}$-symmetric line in the gapped $Z_{2}$ spin liquid phase (cf. Section 6).

## 13. Analogues of the Four Thermodynamic Laws, Fidelity Flows and Miscellanea

### 13.1. Analogues of the Four Thermodynamic Laws

Let us now address whether or not there are any formal similarities between QPTs and black holes, which has been raised as the first question in Section 1. The answer is affirmative. As shown in Table 1, there is a dictionary that translates each notion in one theory to its counterparts in other theories, among fidelity mechanics, black hole thermodynamics [167] and standard thermodynamics [88]. We remark that, in Table 1, the analogues of the four thermodynamic laws in fidelity mechanics are stated in terms of a dominant control parameter *x* and an auxiliary control parameter τ. We emphasize that although fidelity entropy, fidelity temperature, and fidelity internal energy are defined for a fixed τ, both *x* and τ should be regarded as a varying parameter in the formulation of the analogues of the four thermodynamic laws. In particular, they are *only* defined for each principal regime, respectively, in contrast to the original coupling parameters.

In fact, it is more convenient to formulate the analogues of the four thermodynamic laws in fidelity mechanics in terms of the original coupling parameters $x_{1}$ and $x_{2}$, given that there is a one-to-one correspondence between $\left(x_{1},x_{2}\right)$ and (x,τ):

(i) Zeroth law—for a given fidelity mechanical system, which is in equilibrium with its environment, fidelity temperature $T_{f}\left(x_{1},x_{2}\right)$ quantifies quantum fluctuations.
(ii) First law—fidelity internal energy may be transferred from a fidelity mechanical system, as fidelity work or fidelity heat (defined via fidelity entropy), to its environment or vice versa. Mathematically, we have $dU_{f}\left(x_{1},x_{2}\right)=T_{f}\left(x_{1},x_{2}\right)dS_{f}\left(x_{1},x_{2}\right)+\;đW_{f}\left(x_{1},x_{2}\right)$.
(iii) Second law—the total fidelity entropy of a fidelity mechanical system and its environment never decreases. Physically, this amounts to stating that the *information gain we are able to recover from the environment never exceeds information loss incurred due to information erasure in a fidelity mechanical system*. Mathematically, we have $\Delta S_{f}\left(x_{1},x_{2}\right)$
  $+\Delta S_{f}^{e}\left(x_{1},x_{2}\right)\geq 0$. Generically, $\Delta S_{f}\left(x_{1},x_{2}\right)\geq 0$ and $\Delta S_{f}^{e}\left(x_{1},x_{2}\right)\leq 0$. Therefore, $q(x_{1},x_{2})\leq 1$, with $q(x_{1},x_{2})$ being defined by $\Delta S_{f}^{e}\left(x_{1},x_{2}\right)=-q\left(x_{1},x_{2}\right)\Delta S_{f}\left(x_{1},x_{2}\right)$.
(iv) Third law—for a fidelity mechanical system, fidelity entropy $S_{f}\left(x_{1},x_{2}\right)$ approaches a (local) maximum and fidelity temperature $T_{f}\left(x_{1},x_{2}\right)$ approaches zero, as a stable fixed point is approached. However, the probability for accessing a stable fixed point is zero.

### 13.2. Fidelity Flows as an Alternative Form of RG Flows

In real space RG theories, a number of high-energy degrees of freedom are discarded during the construction of an effective Hamiltonian. This results in a reduction in the number of degrees of freedom, leading to an apparent irreversibility and causing complications around this issue. However, fidelity mechanics offers us new insights into our understanding of the irreversibility of RG flows. This is achieved by introducing an alternative form of RG flows—fidelity flows.

A fidelity mechanical system, which is in equilibrium with its environment, is *unstable* under a random perturbation. That is, it is spontaneous for such a fidelity mechanical system to flow away. Therefore, a trajectory is traversed in the parameter space, along which we formally treat $x_{1}$ and $x_{2}$ as a function of time *t*: $x_{1}=x_{1}\left(t\right),x_{2}=x_{2}\left(t\right)$. Then there is a quantum state ψ(t) attached to a point $\left(x_{1}\left(t\right),x_{2}\left(t\right)\right)$ on the trajectory, according to the (time-dependent) Schrödinger equation, with a time-dependent Hamiltonian $H(x_{1}\left(t\right),x_{2}\left(t\right))$. Apparently, there are two possibilities given that fidelity heat capacity $C_{f}\left(x_{1},x_{2}\right)=T_{f}\left(x_{1},x_{2}\right)\Delta S_{f}\left(x_{1},x_{2}\right)/\Delta T_{f}\left(x_{1},x_{2}\right)<0$ is generically negative: (i) If fidelity temperature $T_{f}\left(x_{1},x_{2}\right)$ decreases, then fidelity entropy $S_{f}\left(x_{1},x_{2}\right)$ increases due to information erasure; (ii) if fidelity temperature $T_{f}\left(x_{1},x_{2}\right)$ increases, then fidelity entropy $S_{f}\left(x_{1},x_{2}\right)$ decreases due to information gain. However, the second possibility is forbidden: If it happened, then the future would be remembered, in contradiction to the psychological/computational arrow (cf. Appendix D). Therefore, fidelity entropy $S_{f}\left(x_{1},x_{2}\right)$ monotonically increases and fidelity temperature $T_{f}\left(x_{1},x_{2}\right)$ monotonically decreases along a trajectory. Although such an evolution is time-reversal invariant in quantum mechanics, a corresponding evolution in fidelity mechanics is, generically, irreversible, due to information loss. Here, it is proper to remark that, in contrast to quantum mechanics, there are no equations of motion in fidelity mechanics, a situation exactly the same as in thermodynamics. As a consequence, irreversibility is stronger than time-reversal non-invariance in fidelity mechanics. In other words, *t*, as a microscopic time, appears in the Schrödinger equation. However, a macroscopic time emerges in a fidelity mechanical system. That is, an arrow of time emerges, resulting from information storage via recording information encoded in the past states in media—a key ingredient in a fidelity mechanical system (cf. Appendix D for a definition of both microscopic and macroscopic time). Here, we note that, for a generic trajectory traversed by a fidelity mechanical system in the control parameter space, the past states, recorded in media, differ from the past states that really occurred. Actually, the past states, recorded in media, are subject to changes as time passes. This is a consequence of the fact that an increment in fidelity internal energy is separated into an increment in fidelity heat and an increment in fidelity work. However, only the increment in fidelity heat due to an increment in fidelity entropy is attributed to changes in information storage. Physically, this is plausible, given the fact that difference between the past states recorded in media and the past states that really occurred may be attributed to a difference in the same type of irrelevant information encoded in ground-state wave functions in the same regime. This implies that such a trajectory never crosses any boundary between different regimes even in the same phase.

We define such a trajectory traversed by a fidelity mechanical system in the control parameter space as a fidelity flow. As argued, fidelity flows are irreversible. Following from the second law, fidelity entropy $S_{f}\left(x_{1},x_{2}\right)$ monotonically increases and fidelity temperature $T_{f}\left(x_{1},x_{2}\right)$ monotonically decreases along any fidelity flow: it starts from a point close to an unstable fixed point and ends at a point close to a stable fixed point in the parameter space, with fidelity temperature $T_{f}\left(x_{1},x_{2}\right)$ being divergent at an unstable fixed point and fidelity entropy being a (local) maximum and fidelity temperature being zero at a stable fixed point. Here, we emphasize that, only in this sense, does it make sense to speak of fidelity flows from an unstable fixed point to a stable fixed point. This offers us a characterization of both unstable and stable fixed points in the context of fidelity mechanics.

Fidelity flows, as defined above, may be regarded as an idealized form of RG flows in real space RG theories. Indeed, an effective Hamiltonian may be kept in the same form as the original Hamiltonian, if any irrelevant coupling constants are ignored. In addition, relevant information encoded in ground states is retained and irrelevant information encoded in ground states is discarded during the construction of an effective Hamiltonian, according to a prescribed criterion (cf. Appendix A for the notions of irrelevant and relevant information). Note that different real space RG schemes adopt different criteria, according to which high-energy degrees of freedom are distinguished from low-energy degrees of freedom. We stress that both fidelity flows and RG flows are not unique. Indeed, fidelity flows depend on the choices of a dominant control parameter, whereas RG flows depend on the choices of a criterion to distinguish high energy degrees of freedom from low-energy degrees of freedom. In this sense, there is a correspondence between fidelity flows and RG flows in real space RG theories. Hence, the irreversibility of RG flows in real space RG theories is a manifestation of the second law in fidelity mechanics. This answers the second question concerning the irreversibility of RG flows from an unstable fixed point to a stable fixed point, as raised in Section 1.

However, we emphasize that there is a subtle difference between RG flows in real space RG theories and RG flows in Zamolodchikov’s *c*-theorem: The former only concern discarding a certain type of irrelevant information in a given regime; thus, they never cross any boundary between different regimes. In contrast, the latter involve different critical points due to the existence of a monotonically decreasing *c*-function interpolating between the values of central charge *c* for the ultraviolet and infrared conformal field theories [52,53]. Therefore, it is necessary to extend the current definition of fidelity flows to accommodate this type of RG flows in fidelity mechanics. In this aspect, a brief discussion is presented in Appendix Q, for fidelity flows mimicking real-space RG flows and fidelity flows mimicking Zamolodchikov RG flows, with the quantum spin-1/2 XY model as an illustrative example. This elaborates on the necessity to make a distinction between real-space RG flows and Zamolodchikov RG flows.

**Figure 28:**
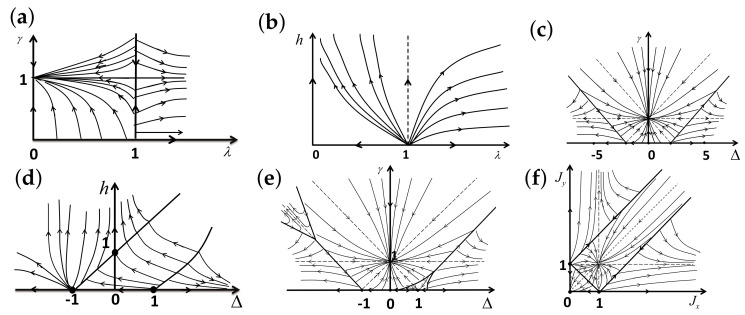
Typical fidelity flows for the six fundamental models. (**a**) For the quantum spin-1/2 XY model, two stable fixed points are identified for the Ising universality class at (0, 1) and (∞, 1), which is protected by the $Z_{2}$ symmetry, and one stable fixed point for the PT universality class at (∞, 0), which is protected by the U(1) symmetry. On the line of the Ising critical points (λ=1 with γ>0), fidelity flows start from γ=∞ and γ=0, respectively, and end at γ=1. (**b**) For the transverse-field quantum Ising model in a longitudinal field, two stable fixed points are identified at (0, 0) and (∞, 0) for the Ising universality class, which is protected by the $Z_{2}$ symmetry, and one stable fixed point is identified at (1, ∞) for the Ising universality class without any symmetry, when λ≠0 and h≠0. In addition, there is one stable fixed point (0, ∞) protected by the U(1) symmetry when λ=0. Indeed, (1, ∞) should be identified with (0, ∞). (**c**) For the quantum spin-1/2 XYZ model, three stable fixed points are identified at (0,1) and (±∞, 1) for the Gaussian universality class, and two stable fixed points are identified at (0, 1) and (∞, 0) for the KT universality class. In addition, a stable fixed point (−∞, 0) originates from the FM universality class (−1,0). Both the KT and FM transitions are protected by the U(1) symmetry as well as the dihedral symmetry group $Z_{2}\times Z_{2}$ and the time-reversal symmetry group $Z_{2}$. A fidelity flow exists on the line of the Gaussian critical points (γ=0 with −1<Δ<1), which starts from (1,0) and ends at (−1,0). This also happens on its dual lines. (**d**) For the quantum spin-1/2 XXZ model in a magnetic field, two stable fixed points (0, ∞) and (−∞, 0) are identified for the PT universality class, one stable fixed point (−∞, 0) originates from the FM transition point (−1,0), and one stable fixed point (∞, 0) is identified for the KT universality class. Both the KT and FM transitions are protected by the U(1) symmetry as well as the dihedral symmetry group $Z_{2}\times Z_{2}$ and the time-reversal symmetry group $Z_{2}$. From an interior point of view, fidelity flows exist along a horizontal line in the XY critical regime, due to the level crossings. (**e**) For the quantum spin-1 XYZ model (γ≥0), three stable fixed points (0, 1) and (±∞, 1) are identified for the Gaussian universality class, one metastable fixed point (1, 0) is identified for the KT transition point ($\Delta_{c1},0$), one stable fixed point (−∞, 0) is identified for the FM transition point (−1,0), both of which are protected by the U(1) symmetry, as well as the dihedral symmetry group $Z_{2}\times Z_{2}$ and the time-reversal symmetry group $Z_{2}$, and two stable fixed points (0,1) and (∞0) and one metastable fixed point (1,0) are identified for the Ising universality class. Note that, for a stable or metastable fixed point, its symmetric or dual images also constitute a stable or metastable fixed point. Fidelity flows exist on the line of the Gaussian critical points: γ=0 with $-1<\Delta <\Delta_{c1}$, starting from $\left(\Delta_{c1},\;0\right)$ and ending at the FM transition point (−1,0). Here, $\Delta_{c1}$ is the KT transition from the critical XY phase to the Haldane phase on the U(1)-symmetric line (γ=0). This also happens on the dual image lines. (**f**) For the spin-1/2 Kitaev model on a honeycomb lattice ($J_{x}>0$, $J_{y}>0$), in addition to three stable fixed points (0,0), (0,∞), and (∞,0), (1,∞) and (∞,1) are also identified as stable fixed points in the gapped $Z_{2}$ spin liquid phases due to the variation of the symmetry group, although (1,∞) and (∞,1) may be identified with (0,∞) and (∞,0), respectively. One stable fixed point (1,1) is identified in the gapless $Z_{2}$ spin liquid phase. From an interior point of view, fidelity flows exist on the boundaries between the gapless $Z_{2}$ spin liquid phase and the gapped $Z_{2}$ spin liquid phase: $J_{y}=1-J_{x}$ with $0\leq J_{x}\leq 1$ and its dual image lines. For $J_{y}=1-J_{x}$ with $0\leq J_{x}\leq 1$, fidelity flows start from the transition points (0,1) and (1,0) and end at the $Z_{2}$-symmetric point (1/2,1/2).

In Figure 28, we sketch typical fidelity flows for the six illustrative models.

(a) For the quantum spin-1/2 XY model (λ≥0 and γ≥0), two stable fixed points are identified for the Ising universality class at (0, 1) and (∞, 1), which is protected by the $Z_{2}$ symmetry, and one stable fixed point for the PT universality class at (∞, 0), which is protected by the U(1) symmetry. For the Ising universality class, a U(1) symmetry emerges at (0, 1) and (∞, 1), in addition to the $Z_{2}$ symmetry, whereas for the PT universality class, a $Z_{2}$ symmetry, defined by $\sigma_{2i}^{x}↔-\sigma_{2i}^{x}$, $\sigma_{2i}^{y}↔-\sigma_{2i}^{y}$, $\sigma_{2i}^{z}↔\sigma_{2i}^{z}$ and $\sigma_{2i+1}^{x}↔\sigma_{2i+1}^{x}$, $\sigma_{2i+1}^{y}↔\sigma_{2i+1}^{y}$, $\sigma_{2i+1}^{z}↔\sigma_{2i+1}^{z}$, emerges at (∞, 0), in addition to the U(1) symmetry. Generically, it is the emergence of such an extra symmetry at a stable fixed point that justifies why it is not accessible. On the other hand, given two lines of critical points belonging to two different universality classes, we interpret the disordered circle as a separation line between two different types of fidelity flows, with one type of fidelity flows starting from unstable fixed points with central charge c=1, and the other type of fidelity flows starting from unstable fixed points with central charge c=1/2. Note that both types of fidelity flows end at the same stable fixed point (0,1), at which fidelity entropy S(λ,γ) reaches a local maximum. From an interior point of view, no fidelity flow exists on the line of the Gaussian critical points (γ=0 with −1<λ<1), reflecting the fact that this line of critical points originates from the level crossings; thus, the ground-state fidelity per lattice site is zero. On the Ising line of critical points (λ=1 with γ>0), central charge *c* is 1 when γ is infinite in value, and *c* is 1/2, when γ is finite and non-zero. Therefore, fidelity flows start from (1, ∞) to (1, 1) and from (1, 0) to (1, 1).
(b) For the transverse-field quantum Ising model in a longitudinal field (h≥0), two stable fixed points (0, 0) and (∞, 0) are identified for the Ising universality class, which is protected by the $Z_{2}$ symmetry, and one stable fixed point (1, ∞) is identified for the Ising universality class without any symmetry, corresponding to the Hamiltonian with λ≠0 and h≠0. In addition, there is one stable fixed point (0, ∞) protected by the U(1) symmetry when λ=0. Indeed, (1, ∞) should be identified with (0, ∞). Note that an extra U(1) symmetry emerges at stable fixed points (0, 0), (∞, 0), and (1, ∞), and an extra $Z_{2}$ symmetry, defined by $\sigma_{2i}^{x}↔\sigma_{2i}^{x}$, $\sigma_{2i}^{y}↔-\sigma_{2i}^{y}$, $\sigma_{2i}^{z}↔-\sigma_{2i}^{z}$ and $\sigma_{2i+1}^{x}↔\sigma_{2i+1}^{x}$, $\sigma_{2i+1}^{y}↔\sigma_{2i+1}^{y}$, $\sigma_{2i+1}^{z}↔\sigma_{2i+1}^{z}$, emerges at a stable fixed point (0, ∞). This justifies why a stable fixed point is not accessible.
(c) For the quantum spin-1/2 XYZ model (γ≥0), three stable fixed points are identified for the Gaussian universality class at (0, 1) and (±∞, 1), and two stable fixed points are identified for the KT universality class at (0, 1) and (∞, 0). In addition, a stable fixed point (−∞, 0) originates from the FM transition point at (−1, 0). Both the KT and FM transitions are protected by the U(1) symmetry as well as the dihedral symmetry group $Z_{2}\times Z_{2}$ and the time-reversal symmetry group $Z_{2}$. The fact that (∞, 1) and (∞, 0) represent two different stable fixed points may be understood from both symmetry-breaking order and RG flows. In fact, a $Z_{2}^{\sigma }\times Z_{2}^{\tau }$ symmetry exists on the line (γ=0 with Δ>1), where $Z_{2}^{\sigma }$ is generated by σ: $\sigma_{i}^{x}↔\sigma_{i}^{y}$ and $\sigma_{i}^{z}↔-\sigma_{i}^{z}$, and $Z_{2}^{\tau }$ is generated by the one-site translation τ: $\sigma_{i}^{\alpha }\to \sigma_{i+1}^{\alpha }$, with α=x,y,z. However, only two-fold degeneracies exist, with each degenerate ground state invariant under the combined action στ, which generates another $Z_{2}^{\sigma \tau }$. Thus, the symmetry group, which is spontaneously broken, is $Z_{2}^{\sigma }\times Z_{2}^{\tau }/Z_{2}^{\sigma \tau }$. This is different from the cases with non-zero γ, in which the spontaneously broken symmetry group is $Z_{2}^{\tau }$. This also matches an observation that, for γ=0, there is a U(1) symmetry, which protects the KT transition. Once γ becomes nonzero, the U(1) symmetry is lost, and a continuous QPT changes from the KT universality class to the Gaussian universality class. In addition, it is the emergence of an extra symmetry at a stable fixed point, such as a U(1) symmetry at (0, 1) and (±∞, 1), and a $Z_{2}$ symmetry, defined by $\sigma_{2i}^{x}↔-\sigma_{2i}^{x}$, $\sigma_{2i}^{y}↔-\sigma_{2i}^{y}$, $\sigma_{2i}^{z}↔\sigma_{2i}^{z}$ and $\sigma_{2i+1}^{x}↔\sigma_{2i+1}^{x}$, $\sigma_{2i+1}^{y}↔\sigma_{2i+1}^{y}$, $\sigma_{2i+1}^{z}↔\sigma_{2i+1}^{z}$, at (∞, 0) that justifies why a stable fixed point is not accessible. From an interior point of view, fidelity flows exist on the line of the Gaussian critical points (γ=0 with −1<Δ<1), starting from (1, 0), and ending at (−1, 0); on the line of the Gaussian critical points (γ=−1+Δ with γ>0), starting from (∞,∞) and ending at (1, 0); on the line of the Gaussian critical points (γ=−1−Δ with γ>0), starting from (−1, 0) and ending at (∞,∞).
(d) For the quantum spin-1/2 XXZ model in a magnetic field, two stable fixed points (0, ∞) and (−∞, 0) are identified for the PT universality class; one stable fixed point (−∞, 0) originates from the FM transition point (−1,0) and one stable fixed point (∞, 0) is identified for the KT universality class, protected by the U(1) symmetry, as well as the dihedral symmetry group $Z_{2}\times Z_{2}$ and the time-reversal symmetry group $Z_{2}$. From an interior point of view, fidelity flows exist in the XY critical regime, starting from an IC transition point on the phase boundary between the XY phase and the AF phase and ending at the line of the PT transition points (h=1+Δ with Δ>−1), in contrast to the chosen dominant control parameter *x*, which is in parallel to the horizontal axis, with *h* fixed. This is because any two ground states with different values of *h* for a given Δ in the XY critical regime are orthogonal to each other due to the level crossings.
(e) For the quantum spin-1 XYZ model (γ≥0), three stable fixed points (0, 1) and (±∞, 1) are identified for the Gaussian universality class; one metastable fixed point (1, 0) is identified for the KT universality class, one stable fixed point (−∞, 0) is identified for the FM transition point (−1,0), both of which are protected by the U(1) symmetry, as well as the dihedral symmetry group $Z_{2}\times Z_{2}$ and the time-reversal symmetry group $Z_{2}$, and two stable fixed points (0,1) and (∞, 0) and one metastable fixed point (1,0) are identified for the Ising universality class. Note that, for a stable or metastable fixed point, its symmetric or dual images also constitutes a stable or metastable fixed point. From an interior point of view, fidelity flows exist on the line of the Gaussian critical points: γ=0 with $-1<\Delta <\Delta_{c1}$, starting from ($\Delta_{c1}$, 0) and ending at the FM transition point (−1, 0). Here, $\Delta_{c1}$ is the KT transition from the critical XY phase to the Haldane phase on the U(1)-symmetric line (γ=0). This also happens on the dual image lines. In addition, fidelity flows exist on the phase boundaries between the Haldane phase and the $Z_{2}$ symmetry-breaking ordered AF phases.
(f) For the spin-1/2 Kitaev model on a honeycomb lattice ($J_{x}>0$ and $J_{y}>0$), in addition to three stable fixed points (0,0), (0,∞), and (∞,0), (1,∞) and (∞,1) are also identified as stable fixed points in the gapped phases due to the variation of the symmetry group, although (1,∞) and (∞,1) may be identified with (0,∞) and (∞,0). One stable fixed point (1,1) is identified in the gapless $Z_{2}$ spin liquid phase. From an interior point of view, fidelity flows exist on the boundaries between the gapless $Z_{2}$ spin liquid phase and the gapped $Z_{2}$ spin liquid phase: $J_{y}=1-J_{x}$ with $0\leq J_{x}\leq 1$ and its dual image lines. For $J_{y}=1-J_{x}$ with $0\leq J_{x}\leq 1$, fidelity flows start from the transition points (0,1) and (1,0) and end at the $Z_{2}$-symmetric point (1/2,1/2).

### 13.3. Miscellanea

Up until this point, we have mainly focused on the first two questions raised in Section 1 regarding similarities between critical points and black holes and the intrinsic irreversibility along RG flows. Here, we briefly address remaining questions.

In our formulation of fidelity mechanics, the analogue of Landauer’s principle at zero temperature has been assumed to keep internal logical consistency (cf. Section 2), which states that in a fidelity mechanical system, to erase one bit of information at zero temperature, we need to perform the minimum fidelity work w(x): w(x)=±T(x)ln2. Here, T(x) characterizes quantum fluctuations at zero temperature, and +/− corresponds to increasing/decreasing e(x) with *x*, respectively. This answers the third question raised in Section 1.

The fourth question concerns an observation that, during the construction of an effective Hamiltonian along any RG flow, an unlimited number of irrelevant coupling constants proliferate. In practice, this prevents access to a stable fixed point. According to fidelity mechanics, this simply follows from the third law in fidelity mechanics. In fact, the third law may be rephrased as follows. *It is impossible to completely erase irrelevant information encoded in ground-state wave functions at any given regime.* Indeed, at a stable fixed point, there exists a singularity in fidelity mechanical-state functions for all models under investigation. In addition, such inaccessibility is also reflected in the conventional Landauer’s SSB theory, since an extra symmetry always emerges at a stable fixed point, as discussed in the preceding subsection.

In our opinion, the traditional definition based on a singularity in the ground-state energy density is under-descriptive, since it fails to signal QPTS in many quantum many-body systems [59]. Moreover, if one defines QPTs as a singularity in any physical quantities, then such a definition is over-descriptive. In fact, according to this definition, factorizing fields would be mistakenly treated as QPTs. In contrast, the ground-state fidelity per lattice site offers us a proper means to detect QPTs, regardless of internal order arising from symmetry-breaking order and/or topological order (cf. Appendix A). Hence, a singularity in the ground-state fidelity per lattice site is a proper criterion to define QPTs, thus offering us an answer to the fifth question raised in Section 1.

Therefore, fidelity mechanics offers a systematic framework to investigate QPTs in quantum many-body systems. It not only provides a characterization of unstable fixed points and stable fixed points but also clarifies in what sense a quantum many-body system flows from an unstable fixed point to a stable fixed point in the control parameter space by erasing irrelevant information encoded in ground-state wave functions along a fidelity flow. In Table 2, we list basic notions in fidelity mechanics, with their counterparts in the conventional theories of local-order parameters and RG flows.

Fidelity mechanics might also offer a novel perspective for understanding a long-standing mystery in physics: why should the thermodynamic, psychological/computational and cosmological arrows of time align with each other? Before proceeding, let us emphasize that the viewpoints expressed below should be regarded as speculative in nature in an attempt to present fidelity mechanics as a tentative theory to describe the psychological/computational arrow of time.

As discussed in Appendix D, only for a macroscopic time does it make sense to speak of an arrow of time. In fact, for any macroscopic time, there must exist a physical process that can, in principle, serve as a clock to track and record it. Therefore, one may single out the psychological/computational arrow of time as a master arrow of time. Then, it is necessary to develop a systematic theory to describe the psychological/computational arrow of time. In fact, the psychological/computational arrow of time is to fidelity mechanics as the thermodynamical arrow of time is to thermodynamics. The fact that both entropy and fidelity entropy are monotonically increasing underlies why the thermodynamic arrow of time aligns with the psychological/computational arrow of time. As for the cosmological arrow of time, we examine the universe from a fidelity mechanical perspective. Since the universe itself is a perfect example of naturally occurring physical systems that act as memories or records, it is a fidelity mechanical system. Here, we point out that, although fidelity mechanics is formalized for QPTs at zero temperature, it may be extended to finite temperature, as briefly discussed in Appendix F. However, a peculiar feature arises when one treats the universe as a fidelity mechanical system: There is no outside observer. That is, the universe itself is its own observer. Nevertheless, in contrast to classical and quantum mechanics, cosmology is a historical science [168]. As we have learned from cosmology, the universe may be traced back to a big bang by different thresholds, such as the formation of solar systems, the formation of galaxies, the formation of stars, the formation of atoms, and the formation of subatomic particles. One may attribute these thresholds to dynamic phase transitions at different time scales during the evolution of the universe. Then, at each scale, macroscopic time emerges, associated with a non-equilibrium physical process that can, in principle, serve as a clock. However, if one traces back further, the universe is so hot and so dense that it dissolves entirely into fluctuations at the Planck scale, with no regular oscillations left; thus, no clock is available. As such, any macroscopic time ceases to exist, but a microscopic time remains due to fluctuations. Therefore, in the universe, fidelity entropy monotonically increases, and so entropy also monotonically increases. If one interprets dark energy as a result of Landauer’s principle [169,170,171,172], then the universe has kept expanding since the big bang. A possible interpretation for dark matter is that the analogue of Landauer’s principle at zero temperature is responsible for dark matter, given the fact that galaxies are traces from quantum fluctuations in the early universe. In this sense, one may speculate that the cosmological arrow of time results from the psychological/computational arrow of time.

## 14. Outlook

A natural question concerns whether or not fidelity mechanics may provide any insight into our search for the classifications of quantum states of matter and QPTs. Given that characteristic lines, including the duality lines, the symmetric lines, and the factorizing-field lines, impose rigid constraints on fidelity flows, we expect that, for a given quantum many-body system under investigation, it is of paramount importance to elaborate on its characteristic lines in the control parameter space. Since the quantum spin-*s* XYZ model, a typical model exhibiting dualities, occupies a prominent place in conceptual developments in a diversity of research areas [35,65,66,125,173,174], it is natural to investigate a class of quantum many-body systems—an anisotropic extension of the staggered SU(3) spin-1 biquadratic model. The latter itself is a special case of the SU(2) spin-1 bilinear–biquadratic model—a model under extensive investigations, both analytically and numerically [135,136,137,138,139,140,141,142,143,144,145,146,147,148,149,150,151,152,153,154,155,156,157,158]. In our opinion, a full understanding of the underlying physics behind the SU(2) spin-1 bilinear–biquadratic model at a few selected points is still lacking [158]. The motivation to investigate an anisotropic extension of the staggered SU(3) spin-1 biquadratic model lies in the fact that it enjoys duality transformations arising from the underlying symmetric group $S_{3}$ with respect to *x*, *y*, and *z*, in exactly the same way as the quantum spin-1/2 XYZ model.

The Hamiltonian of the anisotropic extension of the spin-1 AF biquadratic model [114] takes the following form

(49) $$H_{AF}\left(J_{x},J_{y},J_{z}\right)=-\sum_{j}{\left(J_{x}S_{j}^{x}S_{j+1}^{x}+J_{y}S_{j}^{y}S_{j+1}^{y}+J_{z}S_{j}^{z}S_{j+1}^{z}\right)}^{2}.$$

Here, $S_{j}^{x}$, $S_{j}^{y}$, and $S_{j}^{z}$ are the spin-1 matrices at a lattice site *j*, and $J_{x}$, $J_{y}$, and $J_{z}$ denote coupling parameters describing anisotropic interactions. The model (49) is symmetrical under a unitary transformation: $S_{j}^{x}\to {\left(-1\right)}^{j}S_{j}^{x}$, $S_{j}^{y}\to {\left(-1\right)}^{j}S_{j}^{y}$, $S_{j}^{z}\to S_{j}^{z}$, accompanied by $J_{x}\to J_{x}$, $J_{y}\to J_{y}$ and $J_{z}\to -J_{z}$, or its counterpart under a cyclic permutation with respect to x,y and *z*. Therefore, we may restrict our discussion to the region: both $J_{x}/J_{z}$ and $J_{y}/J_{z}$ are non-negative. It enjoys distinct symmetry groups with varying coupling parameters: a symmetry group U(1)×U(1) in the entire region, generated by any two of the three generators $K_{xy}$, $K_{yz}$, and $K_{zx}$, with $K_{xy}=\sum_{j}{\left(-1\right)}^{j+1}\left[{\left(S_{j}^{x}\right)}^{2}-{\left(S_{j}^{y}\right)}^{2}\right]$, $K_{yz}=\sum_{j}{\left(-1\right)}^{j+1}\left[{\left(S_{j}^{y}\right)}^{2}-{\left(S_{j}^{z}\right)}^{2}\right]$, and $K_{zx}=\sum_{j}{\left(-1\right)}^{j+1}\left[{\left(S_{j}^{z}\right)}^{2}-{\left(S_{j}^{x}\right)}^{2}\right]$, respectively. It enlarges to a symmetry group SU(2)×U(1) on the three characteristic lines: $J_{x}=J_{y}$, $J_{y}=J_{z}$, and $J_{z}=J_{x}$. Specifically, a symmetry group $\mathrm{SU}{\left(2\right)}_{x,\mathrm{yz}}$ on the characteristic line: $J_{y}=J_{z}$ is generated from $\sum_{x}=\sum_{j}S_{j}^{x}/2$, $\sum_{y}=K_{yz}/2$ and $\sum_{z}=\sum_{j}{\left(-1\right)}^{j+1}\left(S_{j}^{y}S_{j}^{z}+S_{j}^{z}S_{j}^{y}\right)/2$, satisfying $\left[\sum_{\lambda },\sum_{\mu }\right]=i\varepsilon_{\lambda \mu \nu }\sum_{\nu }$, where $\varepsilon_{\lambda \mu \nu }$ is a completely antisymmetric tensor, with $\varepsilon_{xyz}=1$, and λ,μ,ν=x,y,z. The generators of a symmetry group $\mathrm{SU}{\left(2\right)}_{z,\mathrm{xy}}$ on the characteristic line ($J_{x}=J_{y}$) and a symmetry group $\mathrm{SU}{\left(2\right)}_{y,\mathrm{zx}}$ on the characteristic line ($J_{z}=J_{x}$) follow from a cyclic permutation with respect to *x*, *y*, and *z*. Therefore, a symmetry group SU(3) emerges at the isotropic point $J_{x}=J_{y}=J_{z}$, where the model Hamiltonian (49) becomes the SU(3) spin-1 biquadratic model.

**Figure 29:**
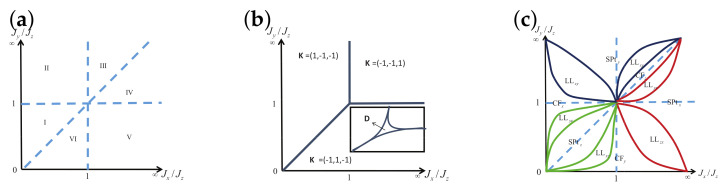
(**a**) The six sub-regions in the control parameter space for the AF spin-1 anisotropic biquadratic model, which are dual relative to each other, if we restrict to the region: $J_{x}/J_{z}\geq 0$ and $J_{y}/J_{z}\geq 0$. Here, sub-region I is chosen to be a principal sub-region. (**b**) A sketch of the ground-state phase diagram for the spin-1 AF anisotropic biquadratic model (49): three distinct SPt phases, characterized by the site-centered non-local order parameter $K=(K_{x},K_{y},K_{z})$, and a dimerized phase, characterized by a local order parameter $D=(D_{x},D_{y},D_{z})$. The inset shows a magnification of the tiny dimerized phase. Note that both the horizontal and vertical axes are shown in a scale defined by $\arctan(J_{x}/J_{z})$ and $\arctan(J_{y}/J_{z})$, respectively. Here, $K=(K_{x},K_{y},K_{z})$ is (1,−1,−1), (−1,−1,1), and (−1,1,−1) in the three distinct SPt phases, respectively. (**c**) A sketch of the ground-state phase diagram for the spin-1 FM anisotropic biquadratic model, which is adapted from [175]. We restrict our attention to the region, defined by $J_{x}/J_{z}\geq 0$ and $J_{y}/J_{z}\geq 0$, due to a symmetric consideration. Here, a solid line indicates a phase transition line. The model (50) accommodates twelve distinct phases: three CF phases labelled as ${\mathrm{CF}}_{x}$, ${\mathrm{CF}}_{y}$ and ${\mathrm{CF}}_{z}$, six LL phases labelled as ${\mathrm{LL}}_{\mathrm{xy}}$, ${\mathrm{LL}}_{\mathrm{yz}}$, ${\mathrm{LL}}_{\mathrm{zx}}$, ${\mathrm{LL}}_{\mathrm{yx}}$, ${\mathrm{LL}}_{\mathrm{xz}}$ and ${\mathrm{LL}}_{\mathrm{zy}}$, and three SPt phases labelled as ${\mathrm{SPt}}_{x}$, ${\mathrm{SPt}}_{y}$, and ${\mathrm{SPt}}_{z}$, respectively. Note that both horizontal and vertical axes are in a scale defined by $\arctan(J_{x}/J_{z})$ and $\arctan(J_{y}/J_{z})$, respectively.

In addition, the Hamiltonian (49) is subject to duality transformations, which are induced from the symmetric group $S_{3}$, consisting of the permutations with respect to *x*, *y* and *z*, which is exactly the same as that for the quantum spin-1/2 XYZ model (cf. Appendix C). Characterized by the dualities and symmetries, the entire control parameter region is partitioned into six different sub-regions, as shown in Figure 29a, which are dual relative to each other. Because of the various symmetries and dualities, we only need to focus on the phases in one of the six sub-regions—a principal sub-region—which is chosen to be sub-region I. Here, we remark that, after the ground-state phase diagram is determined, a sub-region may be further divided into more than one regime, if a phase boundary does not coincide with a dual line. This greatly reduces the resources it needs to unveil the underlying physics behind the model. As shown in Figure 29b, the ground-state phase diagram accommodates four distinct phases: three SPt phases [113] and one dimerized SSB phase. The former are characterized by means of the site-centered non-local order parameter $K=(K_{x},K_{y},K_{z})$, which is defined in Appendix J, and the latter is characterized by means of the dimerized local-order parameter D, where $D=(D_{x},D_{y},D_{z})$, with $D_{\alpha }=\langle S_{j}^{\alpha }S_{j+1}^{\alpha }-S_{j+1}^{\alpha }S_{j+2}^{\alpha }\rangle$, for α=x, *y*, and *z*. Here, $K=(K_{x},K_{y},K_{z})$ is (1,−1,−1), (−1,−1,1), and (−1,1,−1) in the three distinct SPt phases, respectively. The simulation result for the dimerized local order parameter D is presented in Ref. [114].

An interesting feature, as seen from the ground-state phase diagram in Figure 29b, is the emergence of a cycle, similarly to the quantum spin-1 XYZ model. The difference is that a symmetry-breaking ordered phase—the dimerized phase, instead of the Haldane phase—is enclosed inside the cycle. This strongly suggests that it is impossible to adiabatically connect the dimerized phase with an unentangled (factorized) state. This deserves further investigation.

For the FM anisotropic spin-1 biquadratic model, the Hamiltonian $H_{F}\left(J_{x},J_{y},J_{z}\right)$ takes the following form

(50) $$H_{F}\left(J_{x},J_{y},J_{z}\right)=\sum_{j}{\left(J_{x}S_{j}^{x}S_{j+1}^{x}+J_{y}S_{j}^{y}S_{j+1}^{y}+J_{z}S_{j}^{z}S_{j+1}^{z}\right)}^{2}.$$

Since there is only a sign difference between the FM and AF cases, it follows that the dualities and symmetries are identical. This implies that the FM anisotropic spin-1 biquadratic model share the same six sub-regions as the AF anisotropic spin-1 biquadratic model, as shown in Figure 29a. One may choose sub-region I as a principal sub-region. Therefore, we only need to focus on the principal sub-region in order to map out the ground-state phase diagram in terms of the iTEBD algorithm [46,47,48].

The ground-state phase diagram is sketched in Figure 29c, which is adapted from [175]. The model (50) accommodates twelve distinct phases: three coexisting fractal (CF) phases, labelled as ${\mathrm{CF}}_{x}$, ${\mathrm{CF}}_{y}$, and ${\mathrm{CF}}_{z}$; six Luttinger liquid (LL) phases, labelled as ${\mathrm{LL}}_{\mathrm{xy}}$, ${\mathrm{LL}}_{\mathrm{yz}}$, ${\mathrm{LL}}_{\mathrm{zx}}$, ${\mathrm{LL}}_{\mathrm{yx}}$, ${\mathrm{LL}}_{\mathrm{xz}}$, and ${\mathrm{LL}}_{\mathrm{zy}}$; and three SPt phases, labelled as ${\mathrm{SPt}}_{x}$, ${\mathrm{SPt}}_{y}$, and ${\mathrm{SPt}}_{z}$. As it turns out, a novel universality class arises from instabilities of the LL phases towards the CF phases. In addition, QPTs between the LL phases and the SPt phases are identified to be in the KT universality class.

A remarkable fact is that an exotic quantum state of matter—the CF phase, featuring highly degenerate ground states—occurs on the characteristic line ($J_{y}=J_{z}$), with the ground-state energy density being equal to $J_{x}^{2}$. Such a phase also occurs on $J_{z}=J_{x}$ and $J_{x}=J_{y}$, with the ground-state energy density being equal to $J_{y}^{2}$ and $J_{z}^{2}$, respectively. Note that the symmetry group SU(2)×U(1) emerges on the characteristic lines ($J_{y}=J_{z}$, $J_{z}=J_{x}$, and $J_{x}=J_{y}$). Therefore, a sequence of degenerate ground states appear as a result of SSB from SU(2)×U(1) to U(1)×U(1) [102,175]. As it turns out, two symmetry generators are broken, implying that there is one type-B GM [105,106,176,177,178,179,180,181,182,183,184], according to the counting rule for the GMs [105,106]. That is, the number of the type-B GMs $N_{B}$ is equal to one. Here, we emphasize that SSB with type-B GMs survives in one-dimensional quantum many-body systems, in contrast to SSB with type-A GMs. The latter is forbidden in one spatial dimension, as a result of the Mermin–Wagner–Coleman theorem [103,104,185]. In Ref. [102], it is argued that degenerate ground states in the CF phase are scale-invariant but not conformally invariant, reflected in self-similarities underlying a fractal. This is consistent with a previous field-theoretic description for a logarithmic scaling behavior of the block entanglement entropy with the block size, with the prefactor in front of the logarithmic-scaling function being half the fractal dimension $d_{f}$ [186,187] (see also [188,189]). As a result, the identification of the fractal dimension $d_{f}$ with the number of the type-B GMs $N_{B}$ is made: $d_{f}=N_{B}$ [102]. Further developments in the characterization of scale-invariant states arising from SSB with type-B GMs are currently underway, particularly for the Hamiltonian (50) when $J_{x}=J_{y}=J_{z}$.

As it turns out, the duality transformations, arising from the symmetric group $S_{3}$, plays a crucial role, together with the symmetric lines and the factorizing fields, in characterizing the underlying physics behind the model (50). A universal logarithmic scaling behavior of the block entanglement entropy is summarized in Appendix I for scale-invariant states arising from SSB with type-B GMs, which is not only relevant to our characterization of the CF phase but also to the FM phase transitions in the quantum spin-1/2 XYZ model and the quantum spin-1 XYZ model.

An interesting feature, as observed from the ground-state phase diagram in Figure 29c, is the emergence of a cycle consisting of two complete lines of critical points in contrast to cycles for the quantum spin-1 XYZ model and the spin-1/2 Kitaev model on a honeycomb lattice. Here, an SPt phase is enclosed inside the cycle.

A point to be mentioned is that the Hamiltonians (49) and (50) are an anisotropic extension of a spin-1 pure biquadratic model, which itself is a physical realization in terms of the spin-1 matrices for a representation of the Temperley-Lieb algebra [190,191]. The latter is an intriguing topic in mathematics due to its relevance to the Jones polynomial in knot theory [192,193,194,195]. On the other hand, the Temperley–Lieb algebra is closely related with the Yang–Baxter equation—the foundation for exactly solvable quantum many-body systems [125,126,127]. In fact, there are many exactly solvable models that are known to be a representation of the Temperley–Lieb algebra [196,197].

The discussion above illustrates that a characteristic line, together with a complete line of critical points, which are identified as a key ingredient in fidelity mechanics, are fundamental in characterizing quantum critical phenomena.

## 15. Conclusions

In this study, fidelity mechanics has been formalized as a systematic framework to investigate QPTs in quantum many-body systems. Fidelity temperature has been introduced to properly quantify quantum fluctuations, which, together with fidelity entropy and fidelity internal energy, constitute three basic state functions in fidelity mechanics, thus enabling us in formulating analogues of the four thermodynamic laws and Landauer’s principle at zero temperature. It is the notion of information storage that makes it possible to address a novel aspect of quantum information—information extractable by comparing the current state with the past states, both of which are stored in media. In fact, for a given fidelity mechanical system, we are able to quantify what amount of information may be recovered, due to information storage, in terms of fidelity entropy. In addition, the importance of duality in fidelity mechanics has been clarified. Indeed, it plays a defining role in the determination of a canonical form of the Hamiltonian for quantum many-body systems in fidelity mechanics. Fidelity flows have been defined, which are irreversible if information stored in the information storage media leaks into the environment, as follows from the second law in fidelity mechanics. On the other hand, fidelity flows may be interpreted as an alternative form of RG flows and allow us to characterize both stable and unstable fixed points: divergent fidelity temperature for unstable fixed points and zero-fidelity temperature and maximal fidelity entropy for stable fixed points.

Fidelity mechanics characterizes quantum critical phenomena arising not only from symmetry-breaking orders but also from topological orders. A detailed analysis of fidelity mechanical-state functions has been presented for six fundamental models: the quantum XY model, the transverse-field quantum Ising model in a longitudinal field, the quantum spin-1/2 XYZ model, the quantum spin-1/2 XXZ model in a magnetic field, the quantum spin-1 XYZ model, and the spin-1/2 Kitaev model on a honeycomb lattice. With the exception of the quantum spin-1/2 XY model and the spin-1/2 Kitaev model on a honeycomb lattice that are exactly solvable, an extensive simulation of quantum many-body systems in terms of the tensor network algorithms in the matrix-product state representation has been performed. Rich physics has been unveiled even for these well-studied models.

First, for the quantum spin-1/2 XY model, we resolved a confusing point raised in Ref. [119]; as claimed, the so-called long-range entanglement-driven order exists in the disordered regime, suggesting that a QPT occurs on disordered circle $\lambda^{2}+\gamma^{2}=1$. However, the same long-range entanglement-driven order also exists for γ≥1 at λ=0 due to the presence of duality between γ≥1 and γ≤1 at λ=0. In our opinion, no QPT occurs on the disordered circle, but a fidelity mechanical “phase transition” does occur, since fidelity mechanical-state functions exhibit singularities on the disordered circle, which has been interpreted as a separation line between two different types of fidelity flows, with one type of fidelity flows starting from an unstable fixed point with central charge c=1 and the other type of fidelity flows starting from an unstable fixed point with central charge c=1/2. Both types of fidelity flows end at the same stable fixed point (0,1), at which fidelity entropy $S_{f}\left(\lambda ,\gamma \right)$ reaches its local maximum. Another remarkable feature is that fidelity temperature $T_{f}\left(\lambda ,\gamma \right)$ is zero on the disordered circle, as it should be, since no quantum fluctuations exist in a factorized state. However, at the PT transition point (1, 0), fidelity temperature $T_{f}\left(\lambda ,\gamma \right)$ is not well-defined. In fact, it takes any value ranging from 0 to ∞, depending on how it is approached. This bears a resemblance to a previous result [120] that entanglement entropy is not well-defined at the PT transition point (1, 0); its value depends on how the PT transition point (1, 0) is approached. Second, for the transverse-field quantum Ising model in a longitudinal field, there are stable fixed points at (0,0), (0,∞), (∞,0), and (1,∞). The existence of stable fixed points (0,0) and (∞,0) is protected by the $Z_{2}$ symmetry when h=0, whereas the existence of stable fixed points (0,∞) and (1,∞) may be interpreted as a consequence of the variation of the symmetry group with λ: U(1) for λ=0 and none for λ≠0, when h≠0. In particular, the presence of a stable fixed point at (1,∞) might also be related to a well-known fact that, at λ=1 but nonzero *h*, a massive excitation spectrum involves eight massive particles, which shows a deep relation with $E_{8}$ algebraic structure [121]. Third, for the quantum spin-1/2 XYZ model, five different dualities were identified, which enable us to reproduce the ground-state phase diagram. Fourth, for the quantum spin-1/2 XXZ model in a magnetic field, at the phase boundary between the XY phase and the AF phase, fidelity temperature $T_{f}\left(\Delta ,h\right)$ is not well-defined, ranging from a finite value to ∞. That is, an IC transition interpolates between a KT transition and a PT transition, which represents a new universality class. Fifth, the Haldane phase [65,66], as a nontrivial example for the SPT phases, is discussed for the quantum spin-1 XYZ model. It is found that fidelity entropy $T_{f}\left(\Delta ,\gamma \right)$ is double-valued on the U(1)-symmetric line (γ=0) in the Haldane phase, reflecting its topological nature in the control parameter space. Sixth, the spin-1/2 Kitaev model on a honeycomb lattice is discussed as a typical example for topologically ordered states, with the topological nature reflected in the fact that fidelity entropy $T_{f}\left(J_{x},J_{y}\right)$ is multiple-valued at the $S_{3}$-symmetric point (1,1) or at the U(1)-symmetric point (0,0) and its dual image points.

As a consequence, fidelity mechanics offers us a means to characterize QPTs and quantum states of matter. Specifically, if one is not able to discard the double valuedness of fidelity entropy at a QPT point, then it is a topological QPT; if one is not able to discard the double valuedness of fidelity entropy on a symmetric line or the multiple-valuedness of fidelity entropy at a characteristic point, then it is a topological quantum state of matter. This characterization appears to be consistent with the conventional classifications, as our illustrative examples demonstrated. In addition, our characterization offers a smoking-gun signature for the PT transitions, the FM transitions, the KT transitions, the IC transitions, and the TPT transitions from a novel perspective in fidelity mechanics.

We remark that quantum many-body systems, investigated in this study as illustrative examples, are translation-invariant. However, it is possible to extend to non-translation-invariant quantum many-body systems. For this purpose, it is necessary to make some modifications in the definitions of fidelity mechanical-state functions. Specifically, the ground-state fidelity per lattice site is an average, and the ground-state energy density is a geometric average over the entire system, which are briefly discussed in Appendix F. In addition, an extension of fidelity mechanics to finite temperature is also addressed in Appendix F. Moreover, we only focus on spin systems in this study, but our formalism does work also for bosonic and fermionic systems, given that the definition of the ground-state fidelity per lattice site is independent of the types of quantum many-body systems. The only difference is that one has to develop graded tensor network algorithms to simulate a quantum many-body system consisting of fermions [42,43,44,45].

Fidelity flows always start from a point close to an unstable fixed point and end at a point close to a stable fixed point and never cross any characteristic lines, which act as a boundary between distinct regimes in the control parameter space regardless of symmetry-breaking and/or topological order. In particular, the variants of the Hamiltonian at all stable fixed points are unitarily equivalent, irrespective of a symmetry-breaking ordered phase or a topologically ordered phase involved. The only difference between a symmetry-breaking ordered phase or a topologically ordered phase lies in the fact that the unitary operator involved is local for symmetry-breaking order and non-local for topological order. This is consistent with a heuristic argument that, for quantum many-body systems, ground-state wave functions may be represented in terms of the multi-scale entanglement renormalization ansatz [198,199,200], with a top tensor being an unentangled (factorized) state for a symmetry-breaking ordered state and with a top tensor being an entangled state (characterized by a non-local unitary operator) for a topologically ordered state.

Fidelity mechanics has been formalized as an analogue of black hole thermodynamics. In addition to the formal similarity discussed in the main text, they share one more common feature: Both fidelity heat capacity in fidelity mechanics and heat capacity in black hole thermodynamics are negative. Nevertheless, the formal similarity between critical points and black holes, as unveiled, is not surprising in the sense that both QPTs and black holes share singularities as their key ingredients. For a line of critical points, we were able to bring this similarity one step further, thus leading to an analogue of the Hawking radiation in fidelity mechanics.

Moreover, a brief speculative discussion has been presented, justifying why the thermodynamic, psychological/computational, and cosmological arrows of time should align with each other in the context of fidelity mechanics, with the psychological/computational arrow of time being singled out as a master arrow of time. In this sense, fidelity mechanics may be regarded as a tentative theory for describing the psychological/computational arrow of time.

## Figures and Tables

**Table 1:** A dictionary for thermodynamics, black hole mechanics, and fidelity mechanics. Here, $S_{BH}$ is the Bekenstein–Hawking entropy, *A* is the horizon area, $\ell_{p}$ is the Plank length, *E* is the energy, κ is the surface gravity, Ω is the angular velocity, *J* is the angular momentum, Φ is the electrostatic potential, and *Q* is the electric charge. In fidelity mechanics, fidelity internal energy U(x,τ) is defined as $U\left(x,\tau \right)=\mp \ln\left(e\left(x,\tau \right)/e\left(x_{c},\tau \right)\right)V+U_{0}\left(\tau \right)$, where *x* is a dominant control parameter, e(x,τ) is the ground-state energy density, $U_{0}\left(\tau \right)$ is an additive constant, and V(x,τ) is an unknown function of *x* and τ determined from a singular first-order differential equation, Equation (14), with fidelity temperature $T\left(x,\tau \right)=-V_{x}\left(x,\tau \right)$ quantifying quantum fluctuations. Here, a dominant control parameter *x* and an auxiliary control parameter τ are introduced via a one-to-one correspondence between $\left(x_{1},x_{2}\right)$ and (x,τ), and −/+ in fidelity internal energy U(x,τ) corresponds to a monotonically increasing/decreasing e(x,τ) with *x*, respectively. For the sake of brevity, we only list a contribution to fidelity entropy from the ground-state fidelity per lattice site, although it also contains a contribution from scaling entropy if a re-parametrization operation or a duality transformation is involved for a specific model.

Thermodynamics	Black Hole Thermodynamics	Fidelity Mechanics
Temperature *T*	Surface gravity κ	Fidelity temperature T(x,τ)
dU=TdS+đW	$dE=\frac{\kappa }{8\pi }dA+\Omega dJ+\Phi dQ$	dU(x,τ)=T(x,τ)dS(x,τ)+đW(x,τ)
S=klnZ	$S_{BH}=\frac{\kappa A}{4\ell_{p}^{2}}$	$S\left(x,\tau \right)=-2\int_{x_{c}}^{x}\ln d(x,\tau ;y,\tau )dy$ + $S_{0}\left(\tau \right)$
Increasing monotonically	Increasing monotonically	Increasing monotonically
Probability for T=0 is zero	Probability for κ=0 is zero	Probability for getting access to a stable fixed point is zero
Equilibrium states	Static black holes	Ground states
Non-equilibrium states	Dynamic black holes	Low-lying states
Quasi-static	Slowly evolving	Adiabatic

**Table 2:** Fidelity mechanics offers a systematic framework to investigate quantum critical phenomena. Here, we list basic notions in fidelity mechanics, with their counterparts in conventional theories of local-order parameters and RG flows.

Orders and Fluctuations	Renormalization Group	Fidelity Mechanics
Orders	Low-energy degrees of freedom	Relevant information
Fluctuations	High-energy degrees of freedom	Irrelevant information
Local-order parameters	Effective Hamiltonians	Fidelity mechanical quantities
Transition points	Unstable fixed points	Divergent fidelity temperature
Ordered (disordered) states	Stable fixed points	Zero fidelity temperature and maximal fidelity entropy
Not available	RG flows	Fidelity flows

**Figure A1:**
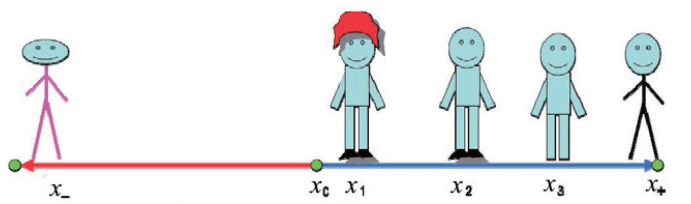
A cartoon picture to illustrate the notions of relevant and irrelevant information in fidelity mechanics. Here, information encoded in bare bodies is relevant, whereas information encoded in hats, clothes, and shoes is irrelevant. RG flows flowing from an unstable fixed point $x_{c}$ to two stable fixed points $x_{-}$ and $x_{+}$ are also depicted to justify that notions of irrelevant and relevant information are introduced as counterparts of high-energy degrees of freedom and low-energy degrees of freedom in real-space RG theories. We remark that irrelevant information originating from the same unstable fixed point is identical, but relevant information at the two stable fixed points is different.

**Figure A2:**
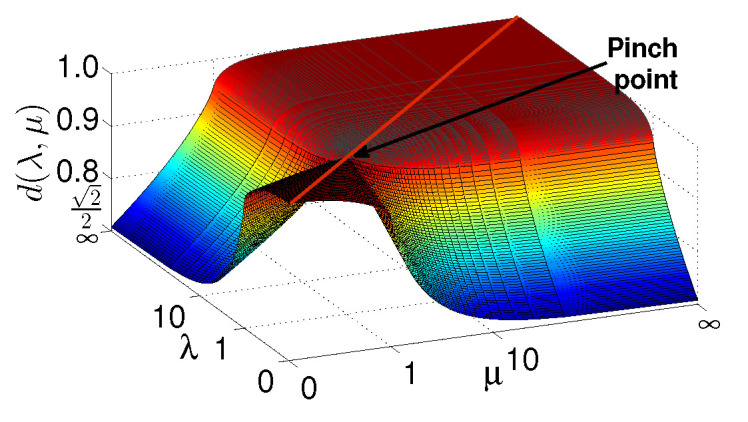
Ground-state fidelity per lattice site d(λ,μ) is shown as a function of λ and μ for the transverse-field quantum Ising model, which exhibits a pinch point at (1,1). A pinch point is defined to be an intersection point between two singular lines (λ=1 and μ=1).

**Figure A3:**
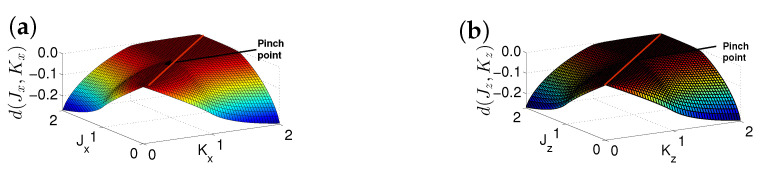
(**a**) Ground-state fidelity per lattice site $d(J_{x},K_{x})$ is shown as a function of $J_{x}$ and $K_{x}$, with $J_{y}=K_{y}=J_{z}=K_{z}=1/2$, for the spin-1/2 Kitaev model on a honeycomb lattice. It exhibits a pinch point at (1,1). (**b**) Ground-state fidelity per lattice site $d(J_{z},K_{z})$ is shown as a function of $J_{z}$ and $K_{z}$, with $J_{x}=K_{x}=J_{y}=K_{y}=1/2$, for the spin-1/2 Kitaev model on a honeycomb lattice. It exhibits a pinch point at (1,1). Here, $d(J_{x},K_{x})$ and $d(J_{z},K_{z})$ are plotted in the logarithmic scale.

**Figure A4:**
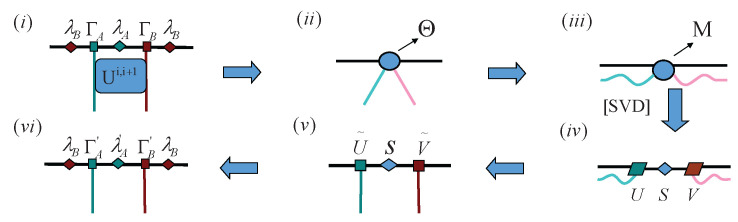
The procedure to update three-index tensors $\Gamma_{A}$ and $\Gamma_{B}$ and two singular-value diagonal matrices $\lambda_{A}$ and $\lambda_{B}$ via absorbing a two-site gate $U_{i,i+1}$. (**i**) A two-site gate $U_{i,i+1}$ is applied onto a matrix-product state, represented in terms of $\Gamma_{A}$, $\lambda_{A}$, $\Gamma_{B}$, and $\lambda_{B}$. (**ii**) A four-index tensor Θ is formed by contracting the tensors $\Gamma_{A}$, $\lambda_{A}$, $\Gamma_{B}$, and $\lambda_{B}$ with the two-site gate $U_{i,i+1}$. (**iii**) Reshape the four-index tensor into a matrix *M*. (**iv**) A singular value decomposition is performed for the matrix *M*, which yields *U*, *V*, and *S*. The latter is formed from the χ largest singular values of *M* due to truncation. That is, $\lambda_{A}$ is updated to *S*. (**v**) Reshape two matrices *U* and *V* into two three-index tensors $\tilde{U}$ and $\tilde{V}$. (**vi**) Recover the singular value diagonal matrix $\lambda_{B}$; thus, $\Gamma_{A}$ and $\Gamma_{B}$ are updated to $\Gamma_{A}^{\prime }$ and $\Gamma_{B}^{\prime }$, respectively.

**Figure A5:**
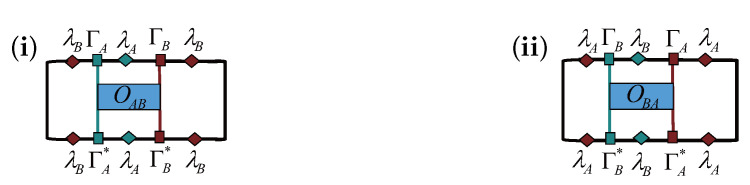
The expectation value of a two-site operator $\langle O_{i,i+1}\rangle$ for a two-site translation-invariant matrix-product state. (**i**) The expectation value of a two-site operator $\langle O_{AB}\rangle$ is computed by contracting tensors $\Gamma_{A}$, $\Gamma_{B}$, $\Gamma_{A}^{*}$, $\Gamma_{B}^{*}$, $\lambda_{A}$, $\lambda_{B}$, and a two-site operator $O_{AB}$. (**ii**) The expectation value of two-site operator $\langle O_{BA}\rangle$ is computed by contracting tensors $\Gamma_{A}$, $\Gamma_{B}$, $\Gamma_{A}^{*}$, $\Gamma_{B}^{*}$, $\lambda_{A}$, $\lambda_{B}$, and a two-site operator $O_{BA}$.

**Figure A6:**
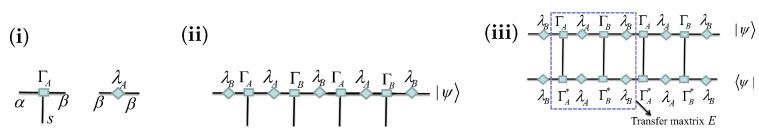
(**i**) A three-index tensor $\Gamma_{A}$ is labelled by two bond indices, denoted as α and β, and one physical index *s*, and $\lambda_{A}$ is a singular value diagonal matrix taking nonzero values only when two bond indices α and β are the same. (**ii**) A two-site translation-invariant matrix-product state representation for a ground-state wave function |ψ〉, consisting of alternating copies of the tensors $\Gamma_{A}$, $\lambda_{A}$, $\Gamma_{B}$, and $\lambda_{B}$, with each tensor connected through two bond indices. (**iii**) A matrix-product state representation for the ground-state fidelity between |ψ(x)〉 and |ψ(y)〉. The former is represented by $\Gamma_{A}$, $\lambda_{A}$, $\Gamma_{B}$, and $\lambda_{B}$, and the latter is represented by $\Lambda_{A}$, $\mu_{A}$, $\Lambda_{B}$, and $\mu_{B}$, respectively. Here, *E* is the transfer matrix.

**Figure A7:**
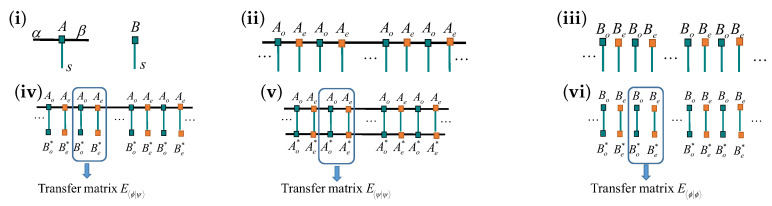
(**i**) A three-index tensor *A* and a one-index tensor *B*. Here, *s* is the physical index, α and β are the inner bond indices. (**ii**) A matrix-product state representation for a ground-state wave function |ψ〉. Here, two three-index tensors $A_{o}$ and $A_{e}$ are attached to odd and even sites, respectively. (**iii**) A matrix-product state representation for a separable state |ϕ〉. Here, two one-index tensors $B_{o}$ and $B_{e}$ are attached to odd and even sites, respectively. (**iv**) The fidelity between a ground-state wave function |ψ〉 and a separable state |ϕ〉. The transfer matrix $E_{\langle \phi |\psi \rangle }$ is constructed from the tensors $A_{o}$, $A_{e}$, $B_{o}^{*}$, and $B_{e}^{*}$. (**v**) The norm for a ground-state wave function |ψ〉, where the transfer matrix $E_{\langle \psi |\psi \rangle }$ is constructed from $A_{o}$, $A_{e}$, and their conjugates. (**vi**) The norm for a separable state |ϕ〉, where the transfer matrix $E_{\langle \phi |\phi \rangle }$ is constructed from $B_{o}$, $B_{e}$, and their conjugates.

**Figure A8:**
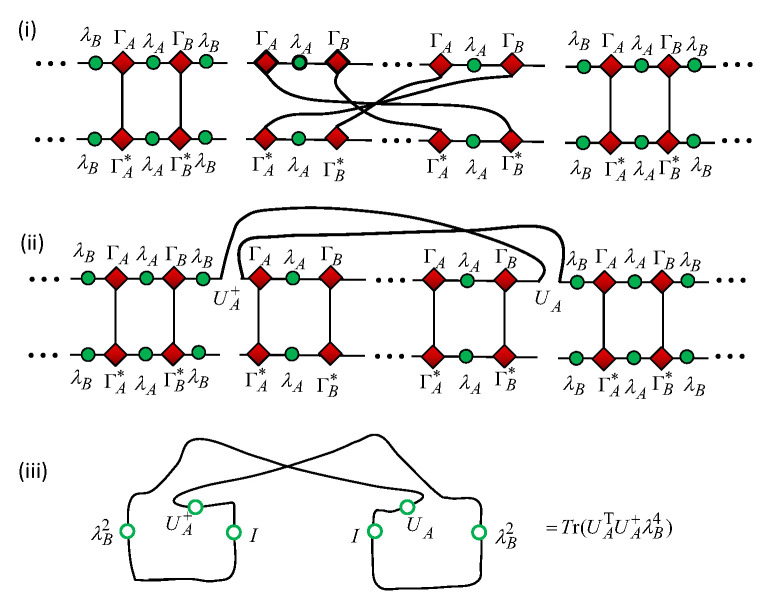
A graphical representation of the bond-centered nonlocal order parameter $O_{2n}$ (up to a proportional constant) for a ground-state wave function in a matrix-product state representation with the two-site translation invariance. (**i**) The overlap of a ground-state wave function ψ with its twisted version, which results from the action of the bond-centered inversion on a segment from 1 to 2n. (**ii**) The overlap is untwisted by reversing the segment by means of the unitary transformations $U_{I,A}$ and $U_{I,B}$. (**iii**) When n becomes large enough, we only need to keep the dominant eigenvector of the transfer matrix E. As a consequence, $O_{2n}=\mathrm{Tr}\left(U_{I,A}^{T}U_{I,A}^{†}\lambda_{B}^{4}\right)/\mathrm{Tr}\left(\lambda_{B}^{4}\right)$ for the segment that consists of n bonds of the type A−B, and $O_{2n}=\mathrm{Tr}\left(U_{I,B}^{T}U_{I,B}^{†}\lambda_{A}^{4}\right)/\mathrm{Tr}\left(\lambda_{A}^{4}\right)$ for the segment that consists of n bonds of the type B−A.

**Figure A9:**
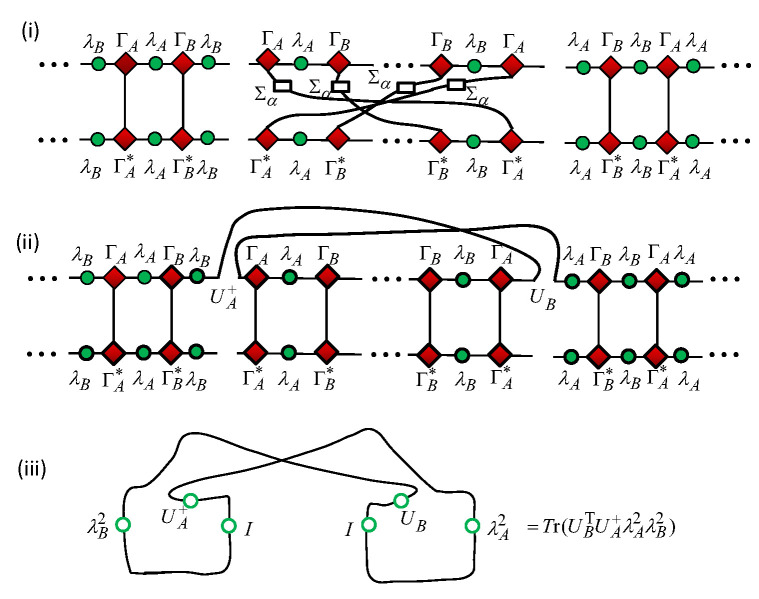
A graphical representation of the site-centered nonlocal order parameter $K_{2n+1,\alpha }$ (up to a proportional constant), with α=x, y, and z, for a ground-state wave function in a matrix-product state representation with the two-site translation invariance. (**i**) The overlap of a ground-state wave function ψ with its twisted version, which results from the action of the combined operation I∗R. (**ii**) The overlap is untwisted by reversing the segment by means of the unitary transformations $U_{I*R,A}$ and $U_{I*R,B}$. (**iii**) When n becomes large enough, we only need to keep the dominant eigenvector of the transfer matrix E. If the site-centered inversion is carried out with respect to an A-site, then it yields $K_{\alpha }=\mathrm{Tr}\left(U_{I*R,B}^{T}U_{I,A}^{†}\lambda_{A}^{2}\lambda_{B}^{2}\right)/\mathrm{Tr}\left(\lambda_{A}^{2}\lambda_{B}^{2}\right)$. If the site-centered inversion is carried out with respect to a B-site, then it yields $K_{\alpha }=\mathrm{Tr}\left(U_{I*R,A}^{T}U_{I,B}^{†}\lambda_{B}^{2}\lambda_{A}^{2}\right)/\mathrm{Tr}\left(\lambda_{B}^{2}\lambda_{A}^{2}\right)$.

**Figure A10:**
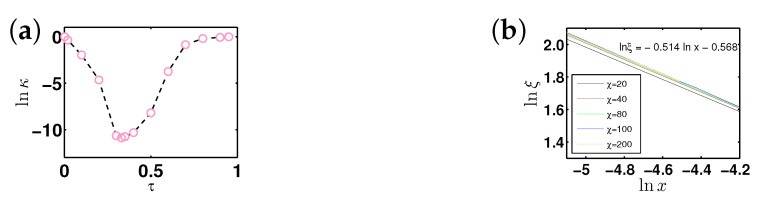
(**a**) In regime I, the parameter lnκ for the transverse-field quantum Ising model in a longitudinal field on the line of discontinuous QPT points. It approaches zero when τ→0 and τ→1 and exhibits a minimum around τ≈0.33. (**b**) In regime II, the critical exponent ν for the correlation length ξ for the transverse-field quantum Ising model in a longitudinal field. The critical point (1,0) is approached along a straight line with τ=π/4. The correlation length ξ scales as $\xi ∼x^{-\nu }$. Our numerical simulation shows that ν∼0.51. For other choices of τ, it yields similar results, with the critical exponent ν ranging from 0.51 to 0.52. Here, the model has been simulated by means of the iTEBD algorithm [39,40,41,46,47,48], in the matrix- product state representation, with the bond dimension χ ranging from 20 to 200.

**Figure A11:**
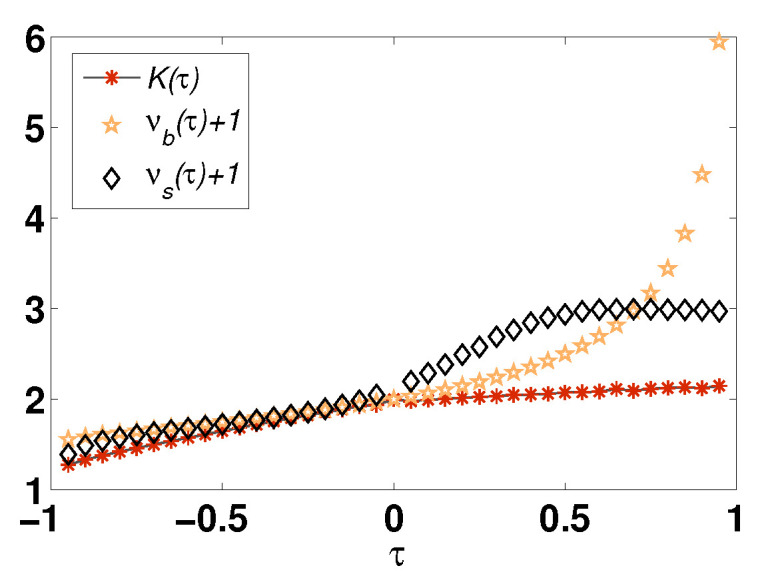
A parameter K(τ), $1+\nu_{b}\left(\tau \right)$, and 1+ν(τ) as a function of τ∈(−1,1). Here, K(τ) is defined via a scaling relation for the ground-state energy density $e\left(x,\tau \right)∼x^{K(\tau )}\;\ln x$ for a fixed τ, and $\nu_{b}\left(\tau \right)$ and ν(τ) represent, respectively, two different sets of the critical exponent ν for the correlation length: one is ν(τ), which is extracted from the scaling behavior of fidelity entropy $S^{I/\mathrm{II}}\left(x,\tau \right)$, and the other is $\nu_{b}\left(\tau \right)$, extracted from the leading singular term via the exact solution.

**Figure A12:**
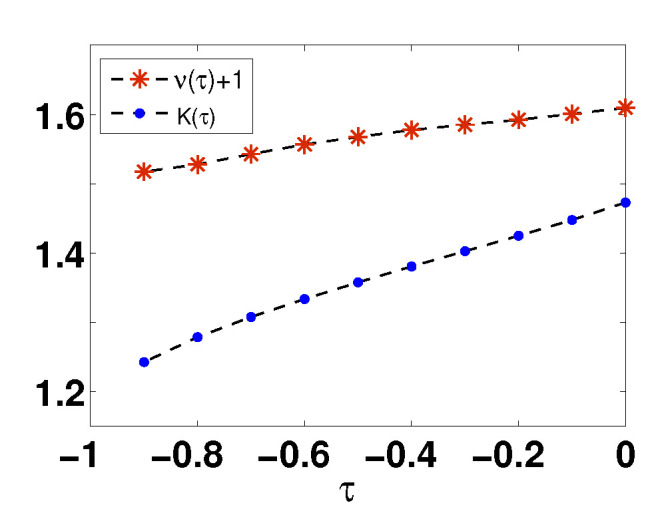
A parameter K(τ) and ν(τ)+1 as a function of τ∈(−1,0). Here, K(τ) is defined via a scaling relation for the ground-state energy density $e^{\mathrm{II}}\left(x,\tau \right)∼x^{K(\tau )}\;\ln x$ for a fixed τ, and ν(τ) represents the critical exponent for the correlation length.

**Figure A13:**
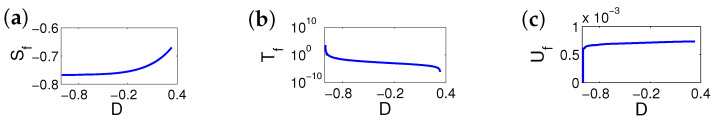
Fidelity entropy $S_{f}\left(D\right)$, fidelity temperature $T_{f}\left(D\right)$, and fidelity internal energy $U_{f}\left(D\right)$ for the spin-1 bilinear–biquadratic model. (**a**) Fidelity entropy $S_{f}\left(D\right)$ monotonically increases from D=−1 to D=1/3 and reaches its maximum at Δ=1/3. (**b**) Fidelity temperature $T_{f}\left(D\right)$ diverges at D=−1, but it is zero at a characteristic point D=1/3. (**c**) Fidelity internal energy $U_{f}\left(D\right)$ monotonically increases, starting from D=−1 to D=1/3, and it reaches its maximum at D=1/3.

**Figure A14:**
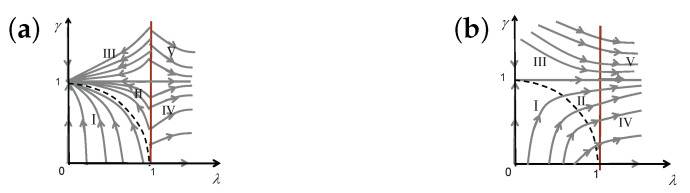
Fidelity flows for the quantum spin-1/2 XY model (1): (**a**) fidelity flows mimicking real-space RG flows and (**b**) fidelity flows mimicking Zamolodchikov RG flows.

**Figure A15:**
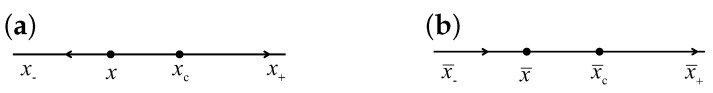
(**a**) A real-space RG flow from $x_{c}$ to $x_{+}$ and from $x_{c}$ to $x_{-}$. (**b**) A Zamolodchikov RG flow from ${\bar{x}}_{-}$ to ${\bar{x}}_{c}$ and from ${\bar{x}}_{c}$ to ${\bar{x}}_{+}$. In the part from $x_{c}$ to $x_{-}$, the direction of the Zamolodchikov RG flow is reversed. Accordingly, a dominant control parameter x is negated to be $\bar{x}$: $\bar{x}=2x_{c}-x$. Here, the presence of $2x_{c}$ is to ensure that ${\bar{x}}_{c}=x_{c}$. In contrast, in the part from $x_{c}$ to $x_{+}$, the direction of the Zamolodchikov RG flow is the same as that of the real-space RG flow. Hence, a dominant control parameter $\bar{x}$ is retained: $\bar{x}=x$.

**Figure A16:**
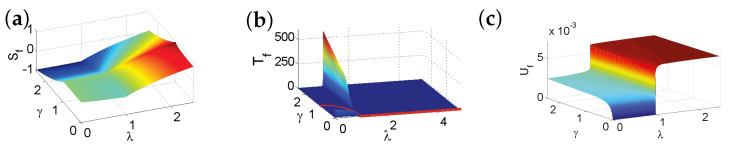
Fidelity internal energy $S_{f}\left(\lambda ,\gamma \right)$, fidelity entropy $T_{f}\left(\lambda ,\gamma \right)$, and fidelity temperature $U_{f}\left(\lambda ,\gamma \right)$ for the quantum spin-1/2 XY model tailored to Zamolodchikov RG flows. Here, we restrict ourselves to the region, defined by λ≥0 and γ≥0, due to the symmetry of the Hamiltonian (1) with respect to γ↔−γ and λ↔−λ. (**a**) Fidelity entropy $S_{f}\left(\lambda ,\gamma \right)$ exhibits singularities at the two dual lines (γ=1 and λ=0), and on the disordered circle: $\lambda^{2}+\gamma^{2}=1$, in addition to the two lines of critical points located at γ=0 with −1<λ<1 and λ=1 with γ≠0. One might view such a singularity as a “phase transition" in fidelity mechanics. Note that fidelity entropy $S_{f}\left(\lambda ,\gamma \right)$ reaches its maximum on the dual line (γ=1) when λ is infinite in value. (**b**) Fidelity temperature $T_{f}\left(\lambda ,\gamma \right)$ diverges at the two lines of critical points (γ=0 with −1<λ<1 and λ=1 with γ≠0), but it is zero on the disordered circle: $\lambda^{2}+\gamma^{2}=1$ and at a characteristic line, representing a factorizing field when λ is infinite in value. (**c**) Fidelity internal energy $U_{f}\left(\lambda ,\gamma \right)$ takes the same value on the disordered circle: $\lambda^{2}+\gamma^{2}=1$, and it reaches its maximum when λ is infinite in value.

## Data Availability

Not applicable.

## Appendix A. Relevant and Irrelevant Information via the Ground-State Fidelity per Lattice Site

Fidelity, a basic notion in quantum information science, is a measure of the similarity between two quantum states |ψ(x)〉 and |ψ(y)〉. Mathematically, it is defined as the absolute value of the overlap between two pure states F(x,y)=|〈ψ(y)|ψ(x)〉|. It should be emphasized that, as a convention, we use *x* and *y* to denote two different values of the *same* control parameter.

For quantum many-body systems, two ground states are always distinguishable (orthogonal) in the thermodynamic limit. As such, the fidelity between these two states vanishes. For a large but finite lattice size *N*, fidelity $F_{N}\left(x,y\right)$ scales as $d^{N}\left(x,y\right)$, with d(x,y) being a scaling parameter. Physically, d(x,y) may be interpreted as the ground-state fidelity per lattice site: $d\left(x,y\right)=\lim_{N\to \infty }F_{N}^{1/N}\left(x,y\right)$, which is well-defined even in the thermodynamic limit. The ground-state fidelity per lattice site d(x,y) enjoys some properties inherited from fidelity $F_{N}(x,y)$: (i) symmetry under interchange d(x,y)=d(y,x); (ii) normalization d(x,x)=1; (iii) range 0≤d(x,y)≤1.

As demonstrated in Refs. [26,27,28], QPTs may be detected through singularities exhibited in the ground-state fidelity per lattice site *d* regardless of the internal order arising from symmetry-breaking and/or topological orders. Such a singularity is reflected as a pinch point. Generically, a pinch point is defined as an intersection point between two singular lines [26]. The reason why the ground-state fidelity per lattice site d(x,y) may be used to signal QPTs is due to the fact that it distinguishes *relevant information* from *irrelevant information* encoded in ground-state wave functions for a quantum many-body system. Here, relevant information is defined to be a counterpart of orders in Landau’s SSB theory. That is, any information encoded in a ground-state wave function corresponding to an ordered (disordered) state is relevant. In contrast, irrelevant information is defined to be a counterpart of fluctuations in Landau’s SSB theory. Therefore, any information encoded in a ground-state wave function that makes it *deviate* from a ground-state wave function at an ordered (disordered) state is irrelevant. A remarkable fact is that such a deviation may be quantified by the ground-state fidelity per lattice site. In this scenario, a critical point is simply characterized as follows. *At a critical point, relevant information is covered up by irrelevant information*. In addition to Landau’s SSB theory, RG flows may also be used to justify the introduction of irrelevant and relevant information as counterparts of the high-energy degrees of freedom and low-energy degrees of freedom in the context of the fidelity approach to QPTs (see a cartoon picture in Figure A1).

Two typical examples are the quantum spin-1/2 XY model (1) and the spin-1/2 Kitaev model on a honeycomb lattice (6). The former exhibits a continuous QPT arising from SSB and the latter exhibits a topological QPT.

The Hamiltonian (1) for the quantum spin-1/2 XY model may be diagonalized by means of the Jordan–Wigner transformation [201], the Fourier transformation, and the Bogoliubov transformation [202]. In the thermodynamic limit N→∞, the logarithmic function lnd(λ,γ;μ,δ) of the ground-state fidelity per lattice site d(λ,γ;μ,δ) takes the following form:

(A1) $$\ln d\left(\lambda ,\gamma ;\mu ,\delta \right)=\frac{1}{2\pi }\int_{0}^{\pi }d\alpha \ln F\left(\lambda ,\gamma ;\mu ,\delta ;\alpha \right),$$

where F(λ,γ;μ,δ;α)=cos[ϑ(λ,γ;α)−ϑ(μ,δ;α)]/2, with $\cos\vartheta \left(\lambda ,\gamma ;\alpha \right)=\left(\cos\alpha -\lambda \right)/\sqrt{{\left(\cos\alpha -\lambda \right)}^{2}+\gamma^{2}\sin^{2}\alpha }$.

The spin-1/2 Kitaev model on a honeycomb lattice may also be diagonalized [33,203,204] by means of the Jordan–Wigner transformation [201], the Fourier transformation, and the Bogoliubov transformation [202]. In the thermodynamic limit N→∞, the logarithmic function $\ln d(\vec{J};\vec{K})$ of the ground-state fidelity per lattice site $d(\vec{J};\vec{K})$ becomes the following:

(A2) $$\ln d\left(\vec{J};\vec{K}\right)=\frac{1}{4\pi^{2}}\int \ln\left(u_{\vec{k}}^{*}\left(\vec{J}\right)u_{\vec{k}}\left(\vec{K}\right)+v_{\vec{k}}^{*}\left(\vec{J}\right)v_{\vec{k}}\left(\vec{K}\right)\right)d\left(\vec{k}\right),$$

where $\vec{J}=\left(J_{x},J_{y},J_{z}\right)$, $\vec{K}=\left(K_{x},K_{y},K_{z}\right)$, $\vec{k}=\left(k_{x},k_{y}\right)$, $|u_{\vec{k}}\left(\vec{J}\right){|}^{2}=1/2\left(1+\varepsilon_{\vec{k}}\left(\vec{J}\right)/E_{\vec{k}}\left(\vec{J}\right)\right)$ and $|v_{\vec{k}}\left(\vec{J}\right){|}^{2}=1/2\left(1-\varepsilon_{\vec{k}}\left(\vec{J}\right)/E_{\vec{k}}\left(\vec{J}\right)\right)$, with the quasiparticle excitation energy $E_{\vec{k}}\left(\vec{J}\right)$ $=\sqrt{\varepsilon_{\vec{k}}^{2}\left(\vec{J}\right)+\Delta_{\vec{k}}^{2}\left(\vec{J}\right)}$ [33] given $\varepsilon_{\vec{k}}\left(\vec{J}\right)=2\left(J_{z}-J_{x}\cos k_{x}-J_{y}\cos k_{y}\right)$ and $\Delta_{\vec{k}}\left(\vec{J}\right)$ $=2\left(J_{x}\sin k_{x}+J_{y}\sin k_{y}\right)$.

For the quantum spin-1/2 XY model, we restrict ourselves to a special case—the transverse-field quantum Ising model, with γ=1. As shown in Figure A2, the critical point $\lambda_{c}=1$ is reflected as a pinch point (1,1) in the fidelity surface [26,27].

For the spin-1/2 Kitaev model on a honeycomb lattice, we choose $J_{x}$ or $J_{z}$ as a control parameter. A critical point at $J_{xc}=1$ is reflected as a pinch point in the fidelity surface at (1,1) for fixed $J_{y}=K_{y}=J_{z}=K_{z}=1/2$ and a critical point at $J_{zc}=1$ is reflected as a pinch point in the fidelity surface at (1,1) for fixed $J_{x}=K_{x}=J_{y}=K_{y}=1/2$, as shown in Figure A3a,b, respectively [29]. We emphasize that the two plots are essentially the same due to the symmetry under permutations: (x,y,z)↔(y,z,x)↔(z,x,y).

Other developments related to the ground-state fidelity per lattice site include a bifurcation point [26,27,28,29,30,49,50,73,74,75] arising from SSB, with a symmetry group being discrete, and a catastrophe point [30,75] arising from pseudo SSB, with a continuous symmetry group, due to the finiteness of the bond dimension during a simulation of quantum many-body systems in the matrix-product state representation. The latter is attributed to a numerical artifact to retain consistency with the Mermin–Wagner–Coleman theorem [103]. However, a catastrophic point is recognized as a smoking-gun signature of the essential singularities associated with, e.g., the KT transitions [30,75].

Here, we emphasize that the definition of the ground-state fidelity per lattice site, as recalled here, is only exploited to detect pinch points, signaling QPTs. For this purpose, there is no need to introduce dominant and auxiliary control parameters. In fact, it is legitimate to keep one of the coupling parameters identical for the sake of simplicity, and so it may be dropped from the arguments. As a result, only two variables are left. In contrast, it is necessary to introduce both dominant and auxiliary control parameters to define fidelity mechanical-state functions, as was performed in Section 2.

## Appendix B. Ground-State Fidelity and Geometric Entanglement from Tensor Networks: Matrix-Product States

In this Appendix, we describe an efficient method for evaluating the ground-state fidelity per lattice site and geometric entanglement for quantum many-body systems in the context of tensor network representations. Here, we restrict ourselves to matrix-product states, which is suitable for quantum many-body systems in one spatial dimension.

### Appendix B.1. The Infinite Time-Evolving Block Decimation Algorithm

We briefly recall the iTEBD algorithm [46,47,48]. The algorithm is based on a matrix-product state representation for a ground-state wave function to simulate infinite-size translation-invariant quantum many-body systems in one spatial dimension.

Consider a quantum many-body system described by the Hamiltonian *H*:

(A3) $$H=\sum_{i}h_{i,i+1},$$

where $h_{i,i+1}$ is the Hamiltonian density describing the nearest-neighbor interactions. A two-site translation-invariant ground-state wave function takes the following form

(A4) $$|\psi \rangle =\sum_{s}\left(\ldots \Gamma_{A}^{s_{2i-1}}\lambda_{A}\Gamma_{B}^{s_{2i}}\lambda_{B}\Gamma_{A}^{s_{2i+1}}\lambda_{A}\Gamma_{B}^{s_{2i+2}}\lambda_{B}\ldots \right)|\ldots s_{2i-1}s_{2i}s_{2i+1}s_{2i+2}\ldots \rangle .$$

Here, *s* is a physical index, $\Gamma_{A}$ and $\Gamma_{B}$ are three-index tensors on odd and even sites, and $\lambda_{A}$ and $\lambda_{B}$ are χ×χ singular value diagonal matrices on odd and even bonds, respectively. If a random matrix-product state $|\psi_{0}\rangle$ in the form of (A4) is chosen as an initial state, then the imaginary time evolution yields $|\psi_{\tau }\rangle$ at imaginary time τ, which takes the following form

(A5) $$|\psi_{\tau }\rangle =\frac{\exp\left(-H\tau \right)|\psi_{0}\rangle }{\left\|\exp\left(-H\tau \right)\right|\psi_{0}\rangle \|}.$$

If τ→∞, then a matrix-product state representation of a ground-state wave function is projected as long as the initial state is not orthogonal to the genuine ground state. The algorithm is efficient, with the computational costs being proportional to $\chi^{3}$.

The imaginary time-evolution operator exp(−Hτ) for τ→∞ is implemented by the operator exp(−Hδτ) over a time slice δτ, where τ=Mδτ, with δτ→0 and M→∞. When δτ is infinitesimal, the evolution operator exp(−Hδτ) may be decomposed into a sequence of the two-site gates $U_{i,i+1}=\exp\left(-h_{i,i+1}\delta \tau \right)$, as a result of the Trotter–Suzuki decomposition. The two-site translational invariance of the Hamiltonian allows us to consider two different types of the two-site gates $U_{e}$ and $U_{o}$ corresponding to even and odd sites, where $U_{e}=\exp\left(-h_{2i,2i+1}\delta \tau \right)$ and $U_{o}=\exp\left(-h_{2i+1,2i}\delta \tau \right)$, respectively. A peculiar feature of such a decomposition is that all two-site gates in $U_{e}$ and $U_{o}$ are commutative with each other. Therefore, the problem to implement the imaginary time evolution reduces to absorb a two-site gate acting on a matrix-product state. This is achieved in terms of the singular value decomposition, as described in Figure A4. Following the procedure, $\Gamma_{A}$, $\lambda_{A}$, $\Gamma_{B}$, and $\lambda_{B}$ are updated to $\Gamma_{A}^{\prime }$, $\lambda_{A}^{\prime }$, $\Gamma_{B}^{\prime }$, and $\lambda_{B}^{\prime }$ via absorbing a two-site gate $U_{i,i+1}$ into a matrix-product state representation. In practice, the initial imaginary time slice δτ may be set as, e.g., $10^{-1}$, and then gradually decrease to a relatively small value. During the simulation, $\Gamma_{A}$, $\lambda_{A}$, $\Gamma_{B}$, and $\lambda_{B}$ are updated repeatedly until the singular value diagonal matrices $\lambda_{A}$ and $\lambda_{B}$ converge up to a preset accuracy.

Once a matrix-product state representation for a ground-state wave function is generated, one may compute the expectation value of any two-site operators $O_{AB}$ and $O_{BA}$ by contracting the tensors, as described in Figure A5i,ii, respectively. Specifically, for $O_{AB}=O_{BA}\equiv h_{i,i+1}$, it yields the ground-state energy density $e=(e_{AB}+e_{BA})/2$, where $e_{AB}$ and $e_{BA}$ represent the expectation values of the Hamiltonian density $h_{i,i+1}$ on the even and odd bonds by contracting the tensors in Figure A5i,ii, respectively.

### Appendix B.2. Ground-State Fidelity per Lattice Site

A ground-state wave function |ψ〉, generated from the iTEBD algorithm [46,47,48], is translation-invariant under a two-site shift. Then, one only needs two three-index tensors $\Gamma_{A}$ and $\Gamma_{B}$ and two singular-value diagonal matrices $\lambda_{A}$ and $\lambda_{B}$ to represent a ground-state wave function |ψ〉, as shown in Figure A6. Here, three-index tensors $\Gamma_{A}$ and $\Gamma_{B}$ are labelled by one physical index *s* and two bond indices α and β, and $\lambda_{A}$ and $\lambda_{B}$ are real and diagonal matrices. Note that the physical index *s* runs over 1,…,d, and each bond index takes 1,…,χ, with d being the physical dimension, and χ being the bond dimension.

Hence, for two ground states, |ψ(x)〉 and |ψ(y)〉, the ground-state fidelity F(x,y) between |ψ(x)〉 and |ψ(y)〉 is represented as a tensor network, with *E* being a transfer matrix, which is shown in Figure A6iii. Here, |ψ(x)〉 is represented by $\Gamma_{A}$, $\lambda_{A}$, $\Gamma_{B}$, and $\lambda_{B}$, and |ψ(y)〉 is represented by tensors $\Lambda_{A}$, $\mu_{A}$, $\Lambda_{B}$, and $\mu_{B}$, respectively. Therefore, the ground-state fidelity per lattice site d(x,y) is, by definition, nothing but the square root of the dominant eigenvalue $\lambda_{max}\left(x,y\right)$ of the transfer matrix *E*: $d\left(x,y\right)=\sqrt{\lambda_{max}\left(x,y\right)}$.

### Appendix B.3. Geometric Entanglement

Geometric entanglement has been introduced as a measure of multi-partite entanglement present in a quantum state [205]. It may be used as an indicator to identify factorized states given that it must vanish for any unentangled states [70,71]. Therefore, it provides a means to numerically locate a factorizing-field line (if any) for a quantum many-body system—one type of the characteristic lines in fidelity mechanics.

For a pure quantum state |ψ〉 with *N* parties, geometric entanglement E(|ψ〉) takes the form $E(|\psi \rangle )=-2\log_{2}\Lambda_{\max}$, where $\Lambda_{\max}$ is the maximum fidelity between |ψ〉 and all possible separable (unentangled) and normalized states |ϕ〉

(A6) $$\Lambda_{\max}=\max_{|\phi \rangle }\;\left|\langle \psi |\phi \rangle \right|.$$

Physically, this amounts to identifying the closest separable (unentangled) state to |ψ〉. Then, geometric entanglement per party $E_{N}(|\psi \rangle )$ is defined as $E_{N}\left(|\psi \rangle )=N^{-1}E(|\psi \rangle \right)$. Equivalently, we have $E_{N}(|\psi \rangle )=-2\log_{2}\lambda_{N}^{\max}$, where $\lambda_{N}^{\max}$ is the maximum fidelity per party, which is defined as $\lambda_{N}^{\max}=\Lambda_{\max}^{\frac{1}{N}}$.

For a quantum many-body system, one may introduce the ground-state geometric entanglement per unit cell $E_{N}(|\psi \rangle )$, which is well-defined even in the thermodynamic limit. Our aim is to find an efficient way to compute the maximum fidelity between a ground-state wave function |ψ〉 and all possible separable (unentangled) and normalized states |ϕ〉 in the context of tensor networks.

A crucial step in evaluating the geometric entanglement per unit cell is how to maximize |〈ϕ|ψ〉| over all the possible separable states |ϕ〉. In this regard, a gradient-directed method turns out to be efficient. Specifically, consider a two-site translation-invariant matrix-product state, represented in terms of three-index tensors $A_{o}$ and $A_{e}$, as shown in Figure A7i,ii. Then, the closest separable state may be represented in terms of one-index tensors $B_{o}$ and $B_{e}$, as shown in Figure A7i,iii. Here, subscripts *o* and *e* represent odd and even sites, respectively. In Figure A7iv, we introduce the transfer matrix $E_{\langle \phi |\psi \rangle }$ for the fidelity between a ground-state wave function |ψ〉 and a separable state |ϕ〉, which is constructed from two three-index tensors, $A_{o}$ and $A_{e}$, and two one-index tensors, $B_{o}^{*}$ and $B_{e}^{*}$. In Figure A7v, we introduce the transfer matrix $E_{\langle \psi |\psi \rangle }$ for the norm of a ground-state wave function |ψ〉, which is constructed from two three-index tensors, $A_{o}$ and $A_{e}$, together with their conjugates. In Figure A7vi, we introduce the transfer matrix $E_{\langle \phi |\phi \rangle }$ for the norm of a separable state |ϕ〉, which is constructed from two one-index tensors, $B_{o}$ and $B_{e}$, together with their conjugates. Then, the fidelity per unit cell λ between a ground-state wave function |ψ〉 and a separable state |ϕ〉 takes the following form:

(A7) $$\lambda =\frac{|\eta_{\langle \phi |\psi \rangle }|}{\sqrt{\eta_{\langle \psi |\psi \rangle }\eta_{\langle \phi |\phi \rangle }}},$$

where $\eta_{\langle \phi |\psi \rangle }$, $\eta_{\langle \psi |\psi \rangle }$, and $\eta_{\langle \phi |\phi \rangle }$ are the dominant eigenvalues of the transfer matrices $E_{\langle \phi |\psi \rangle }$, $E_{\langle \psi |\psi \rangle }$, and $E_{\langle \phi |\phi \rangle }$ for the matrix-product state representations of 〈ϕ|ψ〉, 〈ψ|ψ〉, and 〈ϕ|ϕ〉, respectively. For a normalized |ψ〉, we have $\eta_{\langle \psi |\psi \rangle }=1$.

We then proceed to compute the geometric entanglement per unit cell, which involves the optimization over all separable states. For brevity, we define $F=\lambda^{2}$. The optimization amounts to computing the logarithmic derivative of *F* with respect to $B^{*}$, which is expressed as follows

(A8) $$G\equiv \frac{\partial \ln F}{\partial B^{*}}=\frac{1}{\eta_{\langle \phi |\psi \rangle }}\frac{\partial \eta_{\langle \phi |\psi \rangle }}{\partial B^{*}}-\frac{1}{\eta_{\langle \phi |\phi \rangle }}\frac{\partial \eta_{\langle \phi |\phi \rangle }}{\partial B^{*}}.$$

Here, $B^{*}$ is either $B_{o}^{*}$ or $B_{e}^{*}$. The problem, therefore, reduces to the computation of *G* in the context of tensor network representations. Once *G* is determined, we update the real and imaginary parts of $B^{s}$ separately

$$\begin{array}{ccc} R(B^{s}) & = & R\left(B^{s}\right)+\delta R\left(G^{s}\right),\\ I(B^{s}) & = & I\left(B^{s}\right)+\delta I\left(G^{s}\right). \end{array}$$

Here, δ∈[0,1) is the step size, which is tuned to be decreasing during the optimization process. In addition, we need to normalize the real and imaginary parts of the gradient *G* such that their respective largest entry remains to be unity. If λ converges, then the closest separable state |ϕ〉 is achieved. Therefore, the ground-state geometric entanglement per unit cell for a ground-state wave function |ψ〉 follows.

The argument, presented for the matrix-product states in this Appendix, may be extended to the projected-entangled pair states [39,40,41] and the graded projected-entangled pair states [42,43] suitable for representing ground-state wave functions for quantum many-body systems in two and higher spatial dimensions [28].

## Appendix C. Dualities for the Quantum Spin-s XYZ Model and the Spin-1/2 Kitaev Model on a Honeycomb Lattice

In this Appendix, we present duality transformations for both the quantum spin-s XYZ model and the spin-1/2 Kitaev model on a honeycomb lattice, which are induced from the symmetric group $S_{3}$ generated from the permutations with respect to x, y, and z.

### Appendix C.1. The Quantum Spin-s XYZ Model

In order to treat the quantum spin-1/2 and spin-1 XYZ models on the same footing, as a special case of the quantum spin-s XYZ model, we introduce the spin-1/2 operators $S_{i}^{\alpha }=1/2\;\sigma_{i}^{\alpha }$ (α=x, y, and z). For the quantum spin-s XYZ model, the Hamiltonian takes the same form as Equation (5). There are five different dualities [206]:

(1) The Hamiltonian H(Δ,γ) for γ≥1 is dual relative to the Hamiltonian $H(\Delta^{\prime },\gamma^{\prime })$ for $0<\gamma^{\prime }\leq 1$ under a local unitary transformation $U_{0}$: $S_{2i}^{x}\to S_{2i}^{x}$, $S_{2i}^{y}\to S_{2i}^{y}$, $S_{2i}^{z}\to S_{2i}^{z}$, $S_{2i+1}^{x}\to S_{2i+1}^{x}$, $S_{2i+1}^{y}\to -S_{2i+1}^{y}$ and $S_{2i+1}^{z}\to -S_{2i+1}^{z}$: $H\left(\Delta ,\gamma \right)=k\left(\Delta ,\gamma \right)U_{0}H\left(\Delta^{\prime },\gamma^{\prime }\right)U_{0}^{†}$, with $\Delta^{\prime }=-\Delta /\gamma$, $\gamma^{\prime }=1/\gamma$, and k(Δ,γ)=γ. The Hamiltonian is self-dual when Δ=0 and γ=1.
(2) Under a local unitary transformation $U_{1}$: $S_{i}^{x}\to -S_{i}^{x}$, $S_{i}^{y}\to S_{i}^{z}$, $S_{i}^{z}\to S_{i}^{y}$, we have $H\left(\Delta ,\gamma \right)=k\left(\Delta ,\gamma \right)U_{1}H\left(\Delta^{\prime },\gamma^{\prime }\right)U_{1}^{†}$, with $\Delta^{\prime }=\left(2-2\gamma \right)/\left(1+\gamma +\Delta \right)$, $\gamma^{\prime }=\left(1+\gamma -\Delta \right)/$
  (1+γ+Δ), and k(Δ,γ)=(1+γ+Δ)/2. The Hamiltonian on the line (γ=1−Δ) is self-dual.
(3) Under a local unitary transformation $U_{2}$: $S_{2i}^{x}\to -S_{2i}^{x}$, $S_{2i}^{y}\to -S_{2i}^{z}$, $S_{2i}^{z}\to -S_{2i}^{y}$, $S_{2i+1}^{x}$
  $\to -S_{2i+1}^{x}$, $S_{2i+1}^{y}\to S_{2i+1}^{z}$ and $S_{2i+1}^{z}\to S_{2i+1}^{y}$, we have $H\left(\Delta ,\gamma \right)=k\left(\Delta ,\gamma \right)U_{2}H\left(\Delta^{\prime },\gamma^{\prime }\right)$
  $U_{2}^{†}$, with $\Delta^{\prime }=\left(2\gamma -2\right)/\left(1+\gamma -\Delta \right)$, $\gamma^{\prime }=\left(\gamma +\Delta +1\right)/\left(1+\gamma -\Delta \right)$, and k(Δ,γ)
  =(1+γ−Δ)/2. The Hamiltonian on the line (γ=1+Δ) is self-dual.
(4) Under a local unitary transformation $U_{3}$: $S_{i}^{x}\to S_{i}^{z}$, $S_{i}^{y}\to -S_{i}^{y}$, $S_{i}^{z}\to S_{i}^{x}$, we have $H\left(\Delta ,\gamma \right)=k\left(\Delta ,\gamma \right)U_{3}H\left(\Delta^{\prime },\gamma^{\prime }\right)U_{3}^{†}$, with $\Delta^{\prime }=\left(2+2\gamma \right)/\left(1-\gamma +\Delta \right)$, $\gamma^{\prime }=\left(\gamma +\Delta -1\right)/$
  (1−γ+Δ), and k(Δ,γ)=(1−γ+Δ)/2. The Hamiltonian on the line (γ=−1+Δ) is self-dual.
(5) Under a local unitary transformation $U_{4}$: $S_{2i}^{x}\to -S_{2i}^{z}$, $S_{2i}^{y}\to -S_{2i}^{y}$, $S_{2i}^{z}\to -S_{2i}^{x}$, $S_{2i+1}^{x}$
  $\to S_{2i+1}^{z}$, $S_{2i+1}^{y}\to -S_{2i+1}^{y}$ and $S_{2i+1}^{z}\to S_{2i+1}^{x}$, we have $H\left(\Delta ,\gamma \right)=k\left(\Delta ,\gamma \right)U_{4}H\left(\Delta^{\prime },\gamma^{\prime }\right)$
  $U_{4}^{†}$, with $\Delta^{\prime }=\left(2\gamma +2\right)/\left(\gamma +\Delta -1\right)$, $\gamma^{\prime }=\left(-1+\gamma -\Delta \right)/\left(1-\gamma -\Delta \right)$, and k(Δ,γ)
  =(1−γ−Δ)/2. The Hamiltonian on the line (γ=−1−Δ) is self-dual.

Here, we point out that the symmetries of the Hamiltonian (3) under the permutations with respect to x, y, and z have been discussed in Ref. [125], although they are not treated as dualities.

In addition, dualities discussed for the quantum spin-s XYZ model in one spatial dimension may be extended to the quantum XYZ model with arbitrary spin s on a bipartite lattice in any spatial dimensions. Moreover, it is also possible to extend to the quantum XYZ model with arbitrary spin s on a non-bipartite lattice in any spatial dimensions, with the quantum spin-1/2 XYZ model on a triangular lattice as an illustrative example.

### Appendix C.2. The Spin-1/2 Kitaev Model on a Honeycomb Lattice

The spin-1/2 Kitaev model on a honeycomb lattice is described by the Hamiltonian (6). Here, we set $J_{z}=1$ for brevity. Then, the Hamiltonian (6) is denoted as $H(J_{x},J_{y})$.

The Hamiltonian $H(J_{x},J_{y})$ (6) is symmetrical with respect to two symmetric transformations:

(a) A local unitary transformation $U_{0}$: $J_{x}\to J_{y}$, $J_{y}\to J_{x}$, $\sigma_{i}^{x}\to \sigma_{i}^{y}$, $\sigma_{i}^{y}\to \sigma_{i}^{x}$, and $\sigma_{i}^{z}\to -\sigma_{i}^{z}$, accompanied by the lattice symmetry between the x-bonds and the y-bonds.
(b) A local unitary transformation $V_{0}$: $J_{x}\to -J_{y}$, $J_{y}\to -J_{x}$, $\sigma_{2i}^{x}\to -\sigma_{2i}^{y}$, $\sigma_{2i}^{y}\to -\sigma_{2i}^{x}$, $\sigma_{2i}^{z}\to -\sigma_{2i}^{z}$, $\sigma_{2i+1}^{x}\to \sigma_{2i+1}^{y}$, $\sigma_{2i+1}^{y}\to \sigma_{2i+1}^{x}$, and $\sigma_{2i+1}^{z}\to -\sigma_{2i+1}^{z}$, accompanied by the lattice symmetry between the x-bonds and the y-bonds.

The Hamiltonian $H(J_{x},J_{y})$ exhibits four duality transformations:

(1) The Hamiltonian $H(J_{x},J_{y})$ is dual to the Hamiltonian $H(J_{x}^{\prime },J_{y}^{\prime })$ under a local unitary transformation $U_{1}$: $\sigma_{i}^{x}\to -\sigma_{i}^{x}$, $\sigma_{i}^{y}\to \sigma_{i}^{z}$, $\sigma_{i}^{z}\to \sigma_{i}^{y}$, accompanied by the lattice symmetry between the y-bonds and the z-bonds, $H\left(J_{x},J_{y}\right)=k^{\prime }\left(J_{x}^{\prime },J_{y}^{\prime }\right)U_{1}H^{\prime }\left(J_{x}^{\prime },J_{y}^{\prime }\right)U_{1}^{†}$, with $J_{x}=J_{x}^{\prime }/J_{y}^{\prime }$, $J_{y}=1/J_{y}^{\prime }$, and $k^{\prime }\left(J_{x}^{\prime },J_{y}^{\prime }\right)=1/J_{y}^{\prime }$. The Hamiltonian is self-dual when $J_{y}=1$.
(2) The Hamiltonian $H(J_{x},J_{y})$ is dual to the Hamiltonian $H^{\prime }\left(J_{x}^{\prime },J_{y}^{\prime }\right)$ under a local transformation $U_{2}$: $\sigma_{i}^{x}\to \sigma_{i}^{z}$, $\sigma_{i}^{y}\to -\sigma_{i}^{y}$, $\sigma_{i}^{z}\to \sigma_{i}^{x}$, accompanied by the lattice symmetry between the x-bonds and the z-bonds, $H\left(J_{x},J_{y}\right)=k^{\prime }\left(J_{x}^{\prime },J_{y}^{\prime }\right)U_{2}H^{\prime }\left(J_{x}^{\prime },J_{y}^{\prime }\right)U_{2}^{†}$, with $J_{x}=1/J_{x}^{\prime }$, $J_{y}=J_{y}^{\prime }/J_{x}^{\prime }$, and $k^{\prime }\left(J_{x}^{\prime },J_{y}^{\prime }\right)=1/J_{x}^{\prime }$. The Hamiltonian is self-dual when $J_{x}=1$.
(3) For $J_{y}<0$, the Hamiltonian $H(J_{x},J_{y})$ is dual to the Hamiltonian $H^{\prime }\left(J_{x}^{\prime },J_{y}^{\prime }\right)$ under a local transformation $U_{3}$: $\sigma_{2i}^{x}\to -\sigma_{2i}^{x}$, $\sigma_{2i}^{y}\to -\sigma_{2i}^{z}$, $\sigma_{2i}^{z}\to -\sigma_{2i}^{y}$, $\sigma_{2i+1}^{x}\to -\sigma_{2i+1}^{x}$, $\sigma_{2i+1}^{y}\to \sigma_{2i+1}^{z}$ and $\sigma_{2i+1}^{z}\to \sigma_{2i+1}^{y}$, accompanied by the lattice symmetry between the y-bonds and the z-bonds, $H\left(J_{x},J_{y}\right)=k^{\prime }\left(J_{x}^{\prime },J_{y}^{\prime }\right)U_{3}H^{\prime }\left(J_{x}^{\prime },J_{y}^{\prime }\right)U_{3}^{†}$, with $J_{x}=-J_{x}^{\prime }/J_{y}^{\prime }$, $J_{y}=1/J_{y}^{\prime }$, and $k^{\prime }\left(J_{x}^{\prime },J_{y}^{\prime }\right)=-1/J_{y}^{\prime }$. The Hamiltonian is self-dual when $J_{y}=-1$.
(4) For $J_{x}<0$, the Hamiltonian $H(J_{x},J_{y})$ is dual to the Hamiltonian $H^{\prime }\left(J_{x}^{\prime },J_{y}^{\prime }\right)$ under a local transformation $U_{4}$: $\sigma_{2i}^{x}\to -\sigma_{2i}^{z}$, $\sigma_{2i}^{y}\to -\sigma_{2i}^{y}$, $\sigma_{2i}^{z}\to -\sigma_{2i}^{x}$, $\sigma_{2i+1}^{x}\to \sigma_{2i+1}^{z}$, $\sigma_{2i+1}^{y}\to -\sigma_{2i+1}^{y}$ and $\sigma_{2i+1}^{z}\to \sigma_{2i+1}^{x}$, accompanied by the lattice symmetry between the x-bonds and the z-bonds, $H\left(J_{x},J_{y}\right)=k^{\prime }\left(J_{x}^{\prime },J_{y}^{\prime }\right)U_{4}H^{\prime }\left(J_{x}^{\prime },J_{y}^{\prime }\right)U_{4}^{†}$, with $J_{x}=1/J_{x}^{\prime }$, $J_{y}=-J_{y}^{\prime }/J_{x}^{\prime }$, and $k^{\prime }\left(J_{x}^{\prime },J_{y}^{\prime }\right)=-1/J_{x}^{\prime }$. The Hamiltonian is self-dual when $J_{x}=-1$.

## Appendix D. Thermodynamic Arrow of Time, Psychological/Computational Arrow of Time, and Cosmological Arrow of Time

There are at least three arrows of time: the thermodynamic arrow of time, the psychological arrow of time, and the cosmological arrow of time. Actually, ten arrows of time have been listed in Ref. [86]. It remains to be controversial whether or not there is a single arrow of time, which governs all physical processes.

As our everyday experience shows, we remember the past but not the future. This defines the psychological arrow of time. The psychological arrow of time may be rephrased as the computational arrow of time, if cognitive processes are regarded as computational. However, how do we distinguish the future from the past given the interchangeability of past and future with respect to the laws of microscopic physics? One possible answer is that the observed asymmetry of past and future arises from the second law of thermodynamics, which states that the entropy of an isolated thermodynamic system increases monotonically. This defines the thermodynamic arrow of time. The cosmological arrow of time arises from the observation that the universe has been expanding since the big bang. In some sense, the present is an idealized point between the past and the future. Many efforts have been made in an attempt to understand why the thermodynamic arrow of time, the psychological/computational arrow of time, and the cosmological arrow of time should align with each other (see, e.g., Hawking [207], Wolpert [208], Hartle [209] and Mlodinow and Brun [210]).

However, time-reversal invariance and reversibility are not the same but independent from each other [211]. Indeed, time-reversal invariance is a property of a dynamical equation, such as the Schrödinger equation, thus involving a set of its solutions, whereas reversibility is a property of one single solution of the dynamical equation. Therefore, a plausible resolution to the apparent contradiction between the interchangeability of past and future with respect to the laws of microscopic physics and the irreversibility of physical processes observed in macroscopic phenomena is based on a conceptual distinction between microscopic time and macroscopic time [212]: Under the time-reversal symmetry operation, the former is symmetrical, but the latter is asymmetrical. In this sense, the mystery regarding arrows of time solely concerns macroscopic time. In addition, microscopic time always exists due to quantum fluctuations arising from the uncertainty principle, whereas macroscopic time may be absent in certain circumstances.

According to Mlodinov and Brun [210], the key to unlock this mystery lies in the presence of a physical system that can function as a memory or a record in the sense of preserving a record of the state of some other system. In our opinion, it is information storage involved in a memory or record that is a key ingredient underlying the arrows of time, including the thermodynamic arrow of time, the psychological/computational arrow of time, and the cosmological arrow of time. This is due to the fact that, for any macroscopic time, there must exist a physical process that can, in principle, serve as a clock to track and record it. With this observation in mind, we may single out the psychological/computational arrow of time as the master arrow of time.

However, no systematic theoretical description is available for the psychological/computational arrow of time, in contrast to the thermodynamic arrow of time and the cosmological arrow of time. Actually, not only is the notion of entropy available to measure degree of disorder in a thermodynamic system but also the entire machinery based on thermodynamics offers a full description of physical properties of the system. There is also a plethora of theories on the big bang to describe different scenarios for the cosmological arrow of time. In this aspect, fidelity mechanics may be regarded as an attempt to understand the psychological/computational arrow of time in the context of quantum many-body systems and in a sense a specific physical meaning has been attached to the present, the past, and the future via information storage.

## Appendix E. Three Theorems in Quantum Information Science

We recall three theorems in quantum information science: the no-cloning theorem, the no-deleting theorem, and the no-hiding theorem.

(a) *No-cloning theorem*: It is impossible to create an identical copy of an arbitrary unknown quantum state. The theorem was first articulated in Refs. [213,214]. It has profound implications in quantum information processing. Mathematically, the no-cloning theorem states that for an arbitrary normalized state ${|\psi \rangle }_{A}$ on a system A and an arbitrary normalized state ${|e\rangle }_{B}$ on a system B, there is no unitary operator U satisfying ${U|\psi \rangle }_{A}{|e\rangle }_{B}={\exp i\alpha |\psi \rangle }_{A}{|\psi \rangle }_{B}$, with α depending on |ψ〉 and |e〉.
(b) *No-deleting theorem*: It appears as time-reversed and so is dual to the no-cloning theorem. Given two copies of some arbitrary quantum state, it is impossible to delete one of the copies [215]. Mathematically, suppose |ψ〉 is an unknown quantum state in a Hilbert space. Then, there is no linear isometric transformation U such that ${U|\psi \rangle }_{A}{|\psi \rangle }_{B}{|A\rangle }_{C}={|\psi \rangle }_{A}{|0\rangle }_{B}{|A^{\prime }\rangle }_{C}$, with the final state of the ancilla being independent of |ψ〉.
(c) *No-hiding theorem*: If information is missing from a given system due to interaction with the environment, then it is simply residing somewhere else. In other words, *the missing information cannot be hidden in the correlations between a system and its environment*. It was formalized in Ref. [216] and experimentally confirmed in Ref. [217].

The theorems follow from the linearity of quantum mechanics. In fact, the principle of superposition states that when two evolving states solve the Schrödinger equation, any linear combination of the two is also a solution. As a corollary, perfect copying can be achieved only when states involved are mutually orthogonal to each other [218]. That is, for a collection of mutually orthogonal states, it is possible to set up a quantum copier exclusively tailored to this set of mutually orthogonal states.

## Appendix F. Three Extensions

### Appendix F.1. Fidelity Internal Energy U(x), Fidelity Entropy S(x), and Fidelity Temperature T(x) When the Ground-State Energy Density e(x) Is Always Positive

In Section 2, when fidelity internal energy U(x) was introduced, we required that the ground-state energy density e(x) always be negative for any x. This is not necessary. In fact, it is legitimate to define fidelity internal energy U(x), if e(x)>0 for any x. Specifically, we have the following:

(A9) $$U\left(x\right)=\pm \ln\left(\frac{e(x)}{e(x_{c})}\right)V\left(x\right)+U_{0},$$

with $U_{0}$ being an additive constants. Here, +/− corresponds to a monotonically increasing/decreasing e(x) with x, respectively, and V(x) is a positive-valued function V(x) satisfying a singular first-order differential equation:

(A10) $$V_{x}\left(x\right)=\alpha \left(x\right)\;V\left(x\right),$$

where α(x) is defined to be the following

(A11) $$\alpha \left(x\right)=\mp \frac{{\left(\ln\left(e\left(x\right)/e\left(x_{c}\right)\right)\right)}_{x}}{S_{x}\left(x\right)\pm \ln\left(e\left(x\right)/e\left(x_{c}\right)\right)}.$$

We emphasize that α(x) is always negative, thus guaranteeing that V(x) monotonically decreases with x. Meanwhile, fidelity entropy S(x) and fidelity temperature T(x) are left intact: $S\left(x\right)=-2\int_{x_{c}}^{x}\ln d(x,y)dy+S_{0}$, with $S_{0}$ being an additive constant, and $T\left(x\right)=-V_{x}\left(x\right)$.

### Appendix F.2. Fidelity Entropy, Fidelity Temperature, and Fidelity Internal Energy for Non-Translation-Invariant Quantum Many-Body Systems

Recall that the ground-state fidelity between the two non-translation-invariant ground states |ψ(x)〉 and |ψ(y)〉 takes the form F(x,y)=|〈ψ(x)|ψ(y)〉| and scales exponentially with the system size N: $F\left(x,y\right)={\left[d_{a}\left(x,y\right)\right]}^{N}$, with $d_{a}\left(x,y\right)$ being the ground-state fidelity per lattice site (see Appendix A for the definition, which is valid for both translation-invariant and non-translation-invariant systems). Here, the ground-state fidelity per lattice site $d_{a}\left(x,y\right)$ is understood as an average over the entire system for the non-translation-invariant quantum many-body systems, in contrast to that for translation-invariant quantum many-body systems. In addition, if we assume that $e_{j}\left(x\right)$ is negative for all j, then the average ground-state energy density $e_{a}\left(x\right)$ is, up to a possible sign, defined as a geometric average

(A12) $$e_{a}\left(x\right)=\pm \sqrt[{N}]{e_{1}\left(x\right)e_{2}\left(x\right)\ldots e_{N}\left(x\right)}.$$

Here, it takes a plus sign “+” if N is odd and a minus sign “-” if N is even, and $e_{j}\left(x\right)$ is the ground-state energy density at the j-th lattice site. Physically, this is due to the fact that, for a translation-invariant quantum many-body system, as argued in Section 2, a logarithmic function lne(x) in the definition of fidelity internal energy (10) appears as an integral of a relative uncertainty Δe(x)/e(x), whereas for a non-translation-invariant system, one may anticipate that the relative uncertainty Δe(x)/e(x) is replaced by the average relative uncertainty, i.e., $\sum_{j}\Delta e_{j}\left(x\right)/e_{j}\left(x\right)$ divided by the system size N. As a result, the geometric average $e_{a}\left(x\right)$ appears in the definition of fidelity internal energy.

With the above remarks in mind, fidelity internal energy $U_{f}\left(x\right)$, fidelity temperature $T_{f}\left(x\right)$, and fidelity entropy $S_{f}\left(x\right)$ are determined as follows:

(A13) $$U_{f}\left(x\right)=\pm \ln\frac{e_{a}\left(x\right)}{e_{a}\left(x_{c}\right)}V_{a}\left(x\right)+U_{0},$$

(A14) $$T_{f}\left(x\right)=-\frac{\partial V_{a}\left(x\right)}{\partial x},$$

and

(A15) $$S_{f}\left(x\right)=-2\int_{x_{c}}^{x}\ln d_{a}\left(x,y\right)\mathrm{dy}+S_{0},$$

respectively. Here, +/− corresponds to a monotonically decreasing/increasing $e_{a}\left(x\right)$ with x, when $e_{a}\left(x\right)$ is assumed to be negative for any x, and $S_{0}$ and $U_{0}$ are additive constants. Note that $V_{a}\left(x\right)$, as a positive-valued function of x, satisfies the following first-order differential equation:

(A16) $$\frac{\partial V_{a}\left(x\right)}{\partial x}=\alpha_{a}\left(x\right)\;V_{a}\left(x\right),$$

where $\alpha_{a}\left(x\right)$ is defined to be the following

(A17) $$\alpha_{a}\left(x\right)=\mp \frac{{\left(\ln\left(e_{a}\left(x\right)/e_{a}\left(x_{c}\right)\right)\right)}_{x}}{S_{x}\left(x\right)\pm \ln\left(e_{a}\left(x\right)/e_{a}\left(x_{c}\right)\right)}.$$

Here, $\alpha_{a}\left(x\right)$ is always negative, thus guaranteeing that $V_{a}\left(x\right)$ monotonically decreases with x.

That is, they take exactly the same form as their counterparts for translation-invariant quantum many-body systems when $e_{j}\left(x\right)$ are negative for all j. The same argument works if $e_{j}\left(x\right)$ are positive for all j, similarly to the translation-invariant systems, as discussed in the preceding subsection. The situation becomes more complicated if not all $e_{j}\left(x\right)$ are negative. Generically, there are N roots, as solutions to the algebraic equations $e_{j}\left(x\right)+b=0$, j=1,2,…,N, for a non-translation-invariant system. Hence, it is necessary to extend the approach developed in Section 5 for translation-invariant systems. Physically, the total amount of fidelity work needed to be performed in order to access N roots scales with N linearly in contrast to the case for a translation-invariant system, in which fidelity work needed to be performed to access one root, as a solution to the algebraic equation e(x)+b=0, does not scale with N (cf. Section 5).

To justify our choice for the geometric average $e_{a}\left(x\right)$, we remark that if the arithmetic average $\bar{e}\left(x\right)$ is chosen in the definition of fidelity internal energy, then there is only one root of the algebraic equation $\bar{e}\left(x\right)+b=0$. Fidelity work is needed to be performed to access the root, and it does not scale with N, similarly to the case for a translation-invariant system. Here, the arithmetic average $\bar{e}\left(x\right)$ is defined as $\bar{e}\left(x\right)=(e_{1}\left(x\right)+e_{2}\left(x\right)+\ldots +e_{N}\left(x\right)/N$. However, the root of the algebraic equation $\bar{e}\left(x\right)+b=0$ is shifted away from any roots of the set of the algebraic equations $e_{j}\left(x\right)+b=0$, j=1,2,…,N.

Physically, the adoption of arithmetic average simply means that an information processor is only concerned about the behaviors of the (arithmetically) averaged ground-state energy density $\bar{e}\left(x\right)$, with the uncertainty being defined by $\Delta \bar{e}\left(x\right)/\bar{e}\left(x\right)=(\Delta e_{1}\left(x\right)+\Delta e_{2}\left(x\right)$ $+\ldots +\Delta e_{N}\left(x\right))/\left(N\bar{e}\left(x\right)\right)$, whereas the adoption of the geometric average means that an information processor is concerned with uncertainty $\Delta e_{j}\left(x\right)/e_{j}\left(x\right)$ on each local bond at the lattice site j; thus, the uncertainty involved is $[\Delta e_{1}\left(x\right)/e_{1}\left(x\right)+\Delta e_{2}\left(x\right)/e_{2}\left(x\right)$ $+\ldots +\Delta e_{N}\left(x\right)/e_{N}\left(x\right)]/N$. In our opinion, the geometric average offers much more information, and it is consistent with everyday experience gained from a simulation of quantum many-body system on computers. This quantifies an observation that the computational costs to solve a non-translation-invariant system are, generally speaking, higher.

In principle, it is possible to develop tensor network algorithms to perform efficient numerical simulations for non-translation-invariant quantum many-body systems. In this regard, a few powerful tensor network algorithms are available in the literature [39,40,41,219,220,221,222].

### Appendix F.3. Fidelity Entropy, Fidelity Temperature, and Fidelity Internal Energy at Finite Temperature

Here, we briefly describe an extension of fidelity mechanics to finite temperature when a quantum many-body system is at an equilibrium state, described in terms of a quantum mixed state $\rho_{N}(x)$, with x being a dominant control parameter. Here, the dependence of $\rho_{N}(x)$ on an auxiliary control parameter τ is neglected.

At finite temperature, the thermal state fidelity $F_{N}(x,y)$, between two quantum mixed states $\rho_{N}(x)$ and $\rho_{N}(y)$, takes the following form

(A18) $$F_{N}\left(x,y\right)=\mathrm{Tr}\sqrt{\sqrt{\rho_{N}(x)}\rho_{N}\left(y\right)\sqrt{\rho_{N}(x)}}.$$

The thermal state fidelity per lattice site d(x,y) is defined as $d\left(x,y\right)=\lim_{N\to \infty }$ $F_{N}{\left(x,y\right)}^{1/N}$, with N being the system size. As it turns out, d(x,y) is well-defined in the thermodynamic limit. In this case, the free energy density f(x) plays the same role as the ground-state energy density e(x) at zero temperature.

Suppose a quantum many-body system undergoes a thermal phase transition at critical temperature $T_{c}$. Then, temperature T is a control parameter in addition to the other control parameters, which appear to be the coupling parameters in the Hamiltonian. Since the dependence of fidelity mechanical-state functions on the coupling parameters has been extensively discussed in Section 2, we focus on the situation that a dominant control parameter x is chosen to be relevant to T. If $0<T<T_{c}$, one may choose x=T. If $T>T_{c}$, one may choose x=T/(1+T).

With this fact in mind, fidelity internal energy $U_{f}\left(x\right)$, fidelity temperature $T_{f}\left(x\right)$, and fidelity entropy $S_{f}\left(x\right)$ are defined as follows:

(A19) $$U_{f}\left(x\right)=\pm \ln\left(f\left(x\right)/f\left(x_{c}\right)\right)V\left(x\right)+U_{0},$$

(A20) $$T_{f}\left(x\right)=-V_{x}\left(x\right),$$

and

(A21) $$S_{f}\left(x\right)=-2\int_{x_{c}}^{x}\ln d\left(x,y\right)dy+S_{0},$$

where V(x) is determined from a singular first-order differential equation

(A22) $$V_{x}\left(x\right)=\alpha \left(x\right)V\left(x\right),$$

with

(A23) $$\alpha \left(x\right)=\mp \frac{{\left(\ln\left(f\left(x\right)/f\left(x_{c}\right)\right)\right)}_{x}}{S_{x}\left(x\right)\pm \ln\left(f\left(x\right)/f\left(x_{c}\right)\right)}.$$

Here, +/− corresponds a monotonically decreasing/increasing f(x) with x, assuming that f(x) is negative for any x.

In order to efficiently evaluate fidelity mechanical-state functions at finite temperature, it is necessary to develop finite-temperature tensor network algorithms [223,224,225]. In fact, the thermal-state fidelity per lattice site has been introduced to signal thermal phase transitions for quantum many-body systems at finite temperature [225].

## Appendix G. Scaling Entropy

In this Appendix, we focus on scaling entropy in non-principal regimes, which are dual relative to a given principal regime for the quantum spin-1/2 XYZ model, the quantum spin-1 XYZ model, and the spin-1/2 Kitaev model on a honeycomb lattice, according to a prescription described in Section 2 about scaling entropy. Since the contribution to fidelity entropy from the ground-state fidelity per lattice site is the same for all dual regimes, the continuity requirement for fidelity entropy reduces to that for scaling entropy. Here, we remark that a principal part and its dual image parts share the same dominant control parameter x, although its mathematical expression depends on a specific part.

### Appendix G.1. The Quantum Spin-1/2 XYZ Model

For the quantum spin-1/2 XYZ model, with γ≥0, there are twelve regimes that are separated into two groups, with six regimes in each group that are dual to each other: Regime I, regime III, regime V, regime VII, regime IX, and regime XI are dual to each other, whereas regime II, regime IV, regime VI, regime VIII, regime X, and regime XII are dual to each other. Here, we chose regime I and regime II as the two principal regimes. As it turns out, the duality transformations are induced from the symmetric group $S_{3}$ with respect to x, y, and z and from the cyclic group $Z_{2}$ generated from a local unitary operation: $\sigma_{2i}^{x}\to \sigma_{2i}^{x}$, $\sigma_{2i}^{y}\to \sigma_{2i}^{y}$, $\sigma_{2i}^{z}\to \sigma_{2i}^{z}$, $\sigma_{2i+1}^{x}\to \sigma_{2i+1}^{x}$, $\sigma_{2i+1}^{y}\to -\sigma_{2i+1}^{y}$, and $\sigma_{2i+1}^{z}\to -\sigma_{2i+1}^{z}$. In the (Δ,γ) plane, subgroup $Z_{2}^{+}$, generated from an exchange between x and y, induces a symmetric transformation with γ↔−γ. Therefore, we need to consider a left or right coset that induces two primary duality transformations induced from $S_{3}$, with regime I, regime III, and regime V in one group and regime II, regime IV, and regime VI in the other group. First, regime I and regime III share the self-dual line (γ=1−Δ with 0<Δ<1), on which the ground-state energy density is not monotonic, although it is monotonic with x on the semi-self-dual line (Δ=0), with x=γ in regime I and on the semi-self-dual line (γ=1 with 0<Δ<2), with x=(2−Δ)/(2+Δ) in regime III. Thus, it is necessary to perform a re-parametrization operation on the self-dual line (γ=1−Δ with 0<Δ<1). Note that the extent of the control parameters (Δ,γ) is finite in regime III, but it is not finite in regime V. Therefore, according to our discussions in Section 2, regime I, regime III, and regime V constitute an example for the first situation. Then, we consider the group that consists of regime II, regime IV, and regime VI. Here, regime II and regime IV share the factorizing-field line (γ=1+Δ with −1<Δ<0), on which the ground-state energy density is a constant after a re-parametrization operation, although it is monotonic with x on the semi-self-dual line (Δ=0 with 0<γ<1), with x=γ in regime II and on the semi-self-dual line (γ=1 with −2<Δ<0), with x=(2+Δ)/(2−Δ) in regime IV, respectively. Note that the extent of the control parameters (Δ,γ) is finite in regime IV, but it is not finite in regime VI. Therefore, according to our discussions in Section 2, regime II, regime IV, and regime VI constitute an example for the first situation.

Specifically, one primary duality transformation connects regime I with regime III, and the other connects regime III with regime V. First, let us determine scaling entropy $S_{\sigma }^{I}\left(x,\tau \right)$ with the chosen dominant control parameter x and auxiliary control parameter τ in regime I (cf. Section 9). Since the ground state energy density e(Δ,1−Δ) is not monotonic on the U(1)-symmetric line (γ=1−Δ with 0≤Δ≤1), labelled as ii, it is necessary to perform a re-parametrization operation in the ground state energy density e(Δ,1−Δ) (cf. Appendix M): e(Δ,1−Δ)=m(x)e(x), with x=1−Δ/(2−Δ) and m(x)=2/(1+x). As such, the rescaled ground-state energy density e(x) after the re-parametrization operation monotonically decreases with x on the self-dual line (γ=1−Δ with 0≤Δ≤1). Hence, a contribution to scaling entropy $S_{\sigma }^{\mathrm{ii}}\left(x\right)$ on the self-dual line (γ=1−Δ with 0≤Δ≤1), arising from a multiplying factor m(x), takes a minus sign to retain consistency with the analogue of the Landauer’s principle at zero temperature. Given a one-to-one correspondence between the auxiliary control parameter τ in regime I and the dominant control parameter x on the self-dual line (γ=1−Δ with 0≤Δ≤1), this re-parametrization operation induces a contribution to scaling entropy $S_{\sigma }^{I}\left(x,\tau \right)$ in regime I (cf. Appendix M): $S_{\sigma }^{I}\left(x,\tau \right)=-\ln m^{I}\left(x,\tau \right)$, with $m^{I}\left(x,\tau \right)=1+\tau /\left(4-3\tau \right)$, as a result of the continuity requirement for scaling entropy. Second, since the ground-state energy density e(x) on the semi-self-dual line (Δ=0 with 0≤γ≤1), labelled as iii, in regime I monotonically decreases with the chosen dominant control parameter x, with x=γ, a contribution to scaling entropy $S_{\sigma }^{\mathrm{III}}\left(x,\tau \right)$ takes a minus sign to retain consistency with the analogue of the Landauer’s principle at zero temperature. That is, $S_{\sigma }^{\mathrm{III}}\left(x,\tau \right)=S_{\sigma }^{I}\left(x,\tau \right)+S_{\sigma }^{\mathrm{III}\;I}\left(x,\tau \right)$, where $S_{\sigma }^{\mathrm{III}\;I}\left(x,\tau \right)=-\ln k_{\mathrm{III}\;I}\left(x,\tau \right)$, with $k_{\mathrm{III}\;I}\left(x,\tau \right)$ being a multiplying factor $k_{\mathrm{III}\;I}\left(x,\tau \right)=2/\left(1+x+\tau \right)$. Third, the ground-state energy density e(x) on the semi-self-dual line (γ=1 with 0≤Δ≤2) in regime III, with x=(2−Δ)/(2+Δ), which is the dual image line of the semi-self-dual line (Δ=0 with 0≤γ≤1), labelled as iii, monotonically increases with x. However, as mentioned above, the ground state energy density e(Δ,1−Δ) is not monotonic on the U(1)-symmetric line (γ=1−Δ with 0≤Δ≤1), labelled as ii. According to our discussions in Section 2, it requires to perform an alternative re-parametrization operation in the ground state energy density e(Δ,1−Δ): $e\left(\Delta ,1-\Delta \right)=m_{A}\left(x\right)e_{A}\left(x\right)$, with $m_{A}\left(x\right)=1/\left(2-x\right)$, to ensure that the rescaled ground-state energy density $e_{A}\left(x\right)$ monotonically increases with x on the self-dual line (γ=1−Δ with 0≤Δ≤1), labelled as ii. The existence of such an alternative re-parametrization operation ensures that regime III, as the first dual image regime, may be treated as if it was a principal regime, as discussed in Section 2. Hence, a contribution to scaling entropy $S_{\sigma }^{V}\left(x,\tau \right)$ in regime V takes a plus sign to retain consistency with the analogue of the Landauer’s principle at zero temperature. That is, $S_{\sigma }^{V}\left(x,\tau \right)=S_{\sigma }^{\mathrm{III}}\left(x,\tau \right)+S_{\sigma }^{V\;\mathrm{III}}\left(x,\tau \right)$, where $S_{\sigma }^{V\;\mathrm{III}}\left(x,\tau \right)=\ln k_{V\;\mathrm{III}}\left(x,\tau \right)$, with $k_{V\;\mathrm{III}}\left(x,\tau \right)$ being a multiplying factor $k_{V\;\mathrm{III}}\left(x,\tau \right)=\left(1+x+\tau \right)/\left(1-x+\tau \right)$.

Similarly, one primary duality transformation connects regime II with regime IV and the other connects regime IV with regime VI. First, let us determine scaling entropy $S_{\sigma }^{\mathrm{II}}\left(x,\tau \right)$, with the chosen dominant control parameter x and auxiliary control parameter τ in regime II (cf. Section 9). On the factorizing-field line (γ=1+Δ with −1≤Δ≤0), labelled as i, the same factorized state occurs as the ground state function, with the ground-state energy density e(Δ,1+Δ)=−(2+Δ)/2. Here, a re-parametrization operation was performed(cf. Appendix M): e(Δ,1+Δ)=m(x)e(x), with x=1+Δ and m(x)=(1+x)/2, resulting in the rescaled ground-state energy density e(x)=−1. As such, a contribution to scaling entropy $S_{\sigma }^{i}\left(x\right)$ on the factorizing-field line (γ=1+Δ with −1≤Δ≤0), arising from a multiplying factor m(x), takes a plus sign, as follows from the supplementary rule, given that the rescaled ground-state energy density e(x) is a constant. Given a one-to-one correspondence between the auxiliary control parameter τ in regime II and the dominant control parameter x on the factorizing-field line (γ=1+Δ with −1≤Δ≤0), this re-parametrization operation induces a contribution to scaling entropy $S_{\sigma }^{\mathrm{II}}\left(x,\tau \right)$ in regime II (cf. Appendix M): $S_{\sigma }^{\mathrm{II}}\left(x,\tau \right)=\ln m^{\mathrm{II}}\left(x,\tau \right)$, with $m^{\mathrm{II}}\left(x,\tau \right)=\left(2+\tau \right)/2$, as a result of the continuity requirement for scaling entropy. Second, since the ground-state energy density e(x) monotonically decreases with x on the semi-self-dual line (Δ=0 with 0≤γ≤1), labelled as iii, with x=γ, a contribution to scaling entropy $S_{\sigma }^{\mathrm{IV}}\left(x,\tau \right)$ in regime II takes a minus sign to retain consistency with the analogue of the Landauer’s principle at zero temperature. That is, $S_{\sigma }^{\mathrm{IV}}\left(x,\tau \right)=S_{\sigma }^{\mathrm{II}}\left(x,\tau \right)S_{\sigma }^{\mathrm{IV}\;\mathrm{II}}\left(x,\tau \right)$, where $S_{\sigma }^{\mathrm{IV}\;\mathrm{II}}\left(x,\tau \right)=-\ln k_{\mathrm{IV}\;\mathrm{II}}\left(x,\tau \right)$, with $k_{\mathrm{IV}\;\mathrm{II}}\left(x,\tau \right)$ being a multiplying factor $k_{\mathrm{IV}\;\mathrm{II}}\left(x,\tau \right)=2/\left(1+x-\tau \right)$. Third, the ground-state energy density e(x) monotonically increases with x on the semi-self-dual line (γ=1 with −2≤Δ≤0) in regime IV, with x=(2+Δ)/(2−Δ), which is the dual image line of the semi-self-dual line (Δ=0 with 0≤γ≤1), labelled as iii. Hence, a contribution to scaling entropy $S_{\sigma }^{\mathrm{IV}}\left(x,\tau \right)$ in regime IV takes a plus sign to retain consistency with the analogue of the Landauer’s principle at zero temperature. That is, $S_{\sigma }^{\mathrm{VI}}\left(x,\tau \right)=S_{\sigma }^{\mathrm{IV}}\left(x,\tau \right)+S_{\sigma }^{\mathrm{VI}\;\mathrm{IV}}\left(x,\tau \right)$, where $S_{\sigma }^{\mathrm{VI}\;\mathrm{IV}}\left(x,\tau \right)=\ln k_{\mathrm{VI}\;\mathrm{IV}}\left(x,\tau \right)$, with $k_{\mathrm{VI}\;\mathrm{IV}}\left(x,\tau \right)$ being a multiplying factor $k_{\mathrm{VI}\;\mathrm{IV}}\left(x,\tau \right)=\left(1+x-\tau \right)/\left(1-x-\tau \right)$.

Once $S_{\sigma }^{\beta }\left(x,\tau \right)$ is determined in each of the six regimes for γ<1, scaling entropy $S_{\sigma }^{\beta }\left(\Delta ,\gamma \right)$ follows, with β= I, II, III, IV, V, and VI. That is, $S_{\sigma }^{\beta }\left(\Delta ,\gamma \right)\equiv S_{\sigma }^{\beta }\left(x,\tau \right)$, when we move from x and τ to Δ and γ, meaning that x and τ are regarded as functions of Δ and γ. Hence, a contribution to scaling entropy $S_{\sigma }^{\beta }\left(\Delta ,\gamma \right)$ (β= VII, VIII, IX, X, XI, and XII) takes the form $S_{\sigma }\left(\gamma \right)=-\ln\gamma$ for γ>1, as follows from the primary duality transformation induced by the cyclic group $Z_{2}$, with a multiplying factor of k(Δ,γ)=γ. Here, a minus sign is chosen to retain consistency with the analogue of the Landauer’s principle at zero temperature, since the ground-state energy density monotonically decreases with x on the semi-self dual line (Δ=0), where x=γ, as γ varies from 0 to 1. We remark that the same sign also follows from the rule of thumb. Physically, this is consistent with the fact that all transitions involved belong to the same Gaussian universality class. Hence, we have $S_{\sigma }^{\mathrm{VII}}\left(\Delta ,\gamma \right)=S_{\sigma }^{I}\left(-\Delta /\gamma ,1/\gamma \right)-\ln\gamma$; $S_{\sigma }^{\mathrm{VIII}}\left(\Delta ,\gamma \right)=S_{\sigma }^{\mathrm{II}}\left(-\Delta /\gamma ,1/\gamma \right)-\ln\gamma$; $S_{\sigma }^{\mathrm{IX}}\left(\Delta ,\gamma \right)=S_{\sigma }^{\mathrm{III}}\left(-\Delta /\gamma ,1/\gamma \right)-\ln\gamma$; $S_{\sigma }^{X}\left(\Delta ,\gamma \right)=S_{\sigma }^{\mathrm{IV}}\left(-\Delta /\gamma ,1/\gamma \right)-\ln\gamma$; $S_{\sigma }^{\mathrm{XI}}\left(\Delta ,\gamma \right)=S_{\sigma }^{V}\left(-\Delta /\gamma ,1/\gamma \right)-\ln\gamma$; $S_{\sigma }^{\mathrm{XII}}\left(\Delta ,\gamma \right)=S_{\sigma }^{\mathrm{VI}}\left(-\Delta /\gamma ,1/\gamma \right)-\ln\gamma$.

### Appendix G.2. The Quantum Spin-1 XYZ Model

For the quantum spin-1 XYZ model, with γ≥0, there are twenty four regimes that are separated into four groups, with six regimes in each group that are dual to each other: Regime I, regime V, regime IX, regime XIII, regime XVII, and regime XXI are dual to each other, regime II, regime VI, regime X, regime XIV, regime XVIII, and regime XXII are dual to each other, regime III, regime VII, regime XI, regime XV, regime XIX, and regime XXIII are dual to each other, whereas regime IV, regime VIII, regime XII, regime XVI, regime XX, and regime XXIV are dual to each other. Here, we chose regime I, regime II, regime III, and regime IV as the four principal regimes. As it turns out, the duality transformations are induced from the symmetric group $S_{3}$ with respect to x, y, and z and from the cyclic group $Z_{2}$ generated from a local unitary operation: $S_{2i}^{x}\to S_{2i}^{x}$, $S_{2i}^{y}\to S_{2i}^{y}$, $S_{2i}^{z}\to S_{2i}^{z}$, $S_{2i+1}^{x}\to S_{2i+1}^{x}$, $S_{2i+1}^{y}\to -S_{2i+1}^{y}$, and $S_{2i+1}^{z}\to -S_{2i+1}^{z}$. In the (Δ,γ) plane, the subgroup $Z_{2}^{+}$, generated from an exchange between x and y, induces a symmetric transformation with γ↔−γ. Therefore, we need to consider a left or right coset that induces two primary duality transformations, with regime I, regime V, and regime IX in one group; regime II, regime VI and regime X in one group; regime III, regime VII, and regime XI in one group; and regime IV, regime VIII, and regime XII in one group.

For the first group, regime I and regime V share the self-dual line (γ=1−Δ with $0<\Delta <\Delta_{c0}$), on which the ground-state energy density is not monotonic, although it is monotonic with x on the semi-self-dual line (Δ=0 with $\Delta_{c1}<\Delta <1$), with x=γ in regime I and on the semi-self-dual line (γ=1 with $0<\Delta <-2+2\Delta_{c2}$), with x=(2−Δ)/(2+Δ) in regime V. Here, $\left(\Delta_{c0},1-\Delta_{c0}\right)$ denotes the Ising critical point between the Haldane phase and the ${\mathrm{AF}}_{x}$ phase on the self-dual line (γ=1−Δ with 0<Δ<1); $\left(\Delta_{c1},0\right)$ denotes the KT transition point between the Haldane phase and the critical XY phase on the U(1)-symmetric line (γ=0 with 0<Δ<1); and $\left(\Delta_{c2},-1+\Delta_{c2}\right)$ denotes the KT transition point between the Haldane phase and the critical XY phase on the self-dual line (γ=−1+Δ with 1<Δ<2). Thus, it is necessary to perform a re-parametrization operation on the self-dual line (γ=1−Δ with $0<\Delta <\Delta_{c0}$). Note that the extent of the control parameters (Δ,γ) is finite in regime V, but it is not finite in regime IX. Therefore, according to our discussions in Section 2, regime I, regime V, and regime XI constitute an example for the first situation. For the second group, regime II and regime VI share the factorizing-field line (γ=1+Δ with −1<Δ<0), on which the rescaled ground-state energy density is a constant after a re-parametrization operation, although it is monotonic with x on the semi-self-dual line (Δ=0 with 0<γ<1), with x=γ in regime II and on the semi-self-dual line (γ=1 with −2<Δ<0), with x=(2+Δ)/(2−Δ) in regime VI. Note that the extent of the control parameters (Δ,γ) is finite in regime VI, but it is not finite in regime X. Therefore, according to our discussions in Section 2, regime II, regime VI, and regime X constitute an example for the first situation. For the third group, regime III and regime VII share the self-dual line (γ=1−Δ with $\Delta_{c0}<\Delta <1$), on which the ground-state energy density e(x) is monotonically decreasing with $x=\Delta /\left(2-\Delta \right)-\Delta_{c0}/\left(2-\Delta_{c0}\right)$, and regime VII and regime XI share the self-dual line (γ=−1+Δ with $1<\Delta <\Delta_{c2}$), on which the ground-state energy density e(x) is monotonically increasing with x=−1+2/(1+γ). In addition, the ground-state energy density e(x) is monotonically decreasing on the U(1)-symmetric line (γ=0 with $\Delta_{c1}<\Delta <1$), with $x=\Delta -\Delta_{c1}$ in regime III. That is, the monotonicity of the ground-state energy density with a dominant control parameter is not consistent on the two characteristic lines in the first dual image regime, i.e., regime VII. Therefore, according to our discussions in Section 2, regime III, regime VII, and regime XI constitute an example for the second situation. For the fourth group, the ground-state energy density on the characteristic (soft) line ($\gamma =\Delta_{c1}-\Delta$ with $0<\Delta <\Delta_{c1}$) in regime IV and the ground-state energy density on the characteristic (soft) line ($\gamma =-1+2\Delta_{c2}-\Delta$ with $-2+2\Delta_{c2}<\Delta <\Delta_{c2}$) in regime VIII are not monotonic, although the ground-state energy density is monotonic with x on the U(1)-symmetric line (γ=0 with $0<\Delta <\Delta_{c1}$), with $x=\Delta -\Delta_{c1}$ in regime IV and on the semi-self dual line (γ=1 with $-2+2\Delta_{c2}<\Delta <1$), with x=(2−Δ)/(2+Δ) in regime VIII. Thus, it is necessary to perform a re-parametrization operation on the characteristic (soft) line ($\gamma =\Delta_{c1}-\Delta$ with $0<\Delta <\Delta_{c1}$) in regime IV and on the characteristic (soft) line ($\gamma =-1+2\Delta_{c2}-\Delta$ with $-2+2\Delta_{c2}<\Delta <\Delta_{c2}$) in regime VIII. Note that the extent of the control parameters (Δ,γ) is finite in regime VIII, but it is not finite in regime XII. Therefore, according to our discussions in Section 2, regime IV, regime VIII, and regime XII constitute an example for the first situation.

Specifically, for the first group, one primary duality transformation connects regime I with regime V and the other connects regime V with regime IX. First, let us determine scaling entropy $S_{\sigma }^{I}\left(x,\tau \right)$, with the chosen dominant control parameter x and auxiliary control parameter τ in regime I (cf. Section 11). Since the ground state energy density e(Δ,1−Δ) is not monotonic on the U(1)-symmetric line (γ=1−Δ with $0\leq \Delta \leq \Delta_{c0}$), labelled as ii, it is necessary to perform a re-parametrization operation in the ground state energy density e(Δ,1−Δ) (cf. Appendix O): e(Δ,1−Δ)=m(x)e(x), with $x=\Delta_{c0}/\left(2-\Delta_{c0}\right)-\Delta /\left(2-\Delta \right)$ and $m\left(x\right)=\left(\Delta_{c0}/\left(2-\Delta_{c0}\right)+1\right)/\left(x+1\right)$. As such, the rescaled ground-state energy density e(x) after the re-parametrization operation monotonically decreases with x on the self-dual line (γ=1−Δ with $0\leq \Delta \leq \Delta_{c0}$). A re-parametrization operation was performed in the ground state energy density $e^{I}\left(\Delta ,\gamma \right)$ (cf. Appendix O) in regime I: $e^{I}\left(\Delta ,\gamma \right)=m^{I}\left(x,\tau \right)e^{I}\left(x,\tau \right)$, with $m^{I}\left(x,\tau \right)=\left(\Delta_{c}/\left(2-\Delta_{c}\right)+1\right)/\left(x+1\right)$, to ensure that the rescaled ground state energy density $e^{I}\left(x,\tau \right)$ monotonically decreases with x for a fixed τ. Hence, a contribution to scaling entropy $S_{\sigma }^{I}\left(x,\tau \right)$ in regime I, arising from a multiplying factor $m^{I}\left(x,\tau \right)$, takes a minus sign to retain consistency with the analogue of the Landauer’s principle at zero temperature. That is, $S_{\sigma }^{I}\left(x,\tau \right)=-\ln m^{I}\left(x,\tau \right)$. Second, since the ground-state energy density e(x) monotonically decreases with x on the semi-self-dual line (Δ=0 with $\Delta_{c1}\leq \gamma \leq 1$), labelled as vi, with x=γ in regime I, a contribution to scaling entropy $S_{\sigma }^{V}\left(x,\tau \right)$ takes a minus sign to retain consistency with the analogue of the Landauer’s principle at zero temperature. That is, $S_{\sigma }^{V}\left(x,\tau \right)=S_{\sigma }^{I}\left(x,\tau \right)+S_{\sigma }^{V\;I}\left(x,\tau \right)$, where $S_{\sigma }^{V\;I}\left(x,\tau \right)=-\ln k_{V\;I}\left(x,\tau \right)$, with $k_{V\;I}\left(x,\tau \right)$ being a multiplying factor $k_{V\;I}\left(x,\tau \right)=2/\left(1+\tau \right)$. Third, the ground-state energy density e(x) monotonically increases with x on the semi-self-dual line (γ=1 with $0\leq \Delta \leq -2+2\Delta_{c2}$), with x=(2−Δ)/(2+Δ) in regime V, which is the dual image line of the semi-self-dual line (Δ=0 with $\Delta_{c1}\leq \gamma \leq 1$), labelled as vi. However, as mentioned above, the ground state energy density e(Δ,1−Δ) is not monotonic on the U(1)-symmetric line (γ=1−Δ with $0\leq \Delta \leq \Delta_{c0}$), labelled as ii. According to our discussions in Section 2, it requires to perform an alternative re-parametrization operation in the ground state energy density e(Δ,1−Δ): $e\left(\Delta ,1-\Delta \right)=m_{A}\left(x\right)e_{A}\left(x\right)$, with $m_{A}\left(x\right)=\left(2-\Delta_{c0}\right)/\left(2-2x+\Delta_{c0}x\right)$, to ensure that the rescaled ground-state energy density $e_{A}\left(x\right)$ monotonically increases with x on the self-dual line (γ=1−Δ with $0\leq \Delta \leq \Delta_{c0}$), labelled as ii. The existence of such an alternative re-parametrization operation ensures that regime V, as the first dual image regime, may be treated as if it was a principal regime, as discussed in Section 2. Hence, a contribution to scaling entropy $S_{\sigma }^{\mathrm{IX}}\left(x,\tau \right)$ takes a plus sign to retain consistency with the analogue of the Landauer’s principle at zero temperature. That is, $S_{\sigma }^{\mathrm{IX}}\left(x,\tau \right)=S_{\sigma }^{V}\left(x,\tau \right)+S_{\sigma }^{\mathrm{IX}\;V}\left(x,\tau \right)$, where $S_{\sigma }^{\mathrm{IX}\;V}\left(x,\tau \right)=\ln k_{\mathrm{IX}\;V}\left(x,\tau \right)$, with $k_{\mathrm{IX}\;V}\left(x,\tau \right)$ being a multiplying factor $k_{\mathrm{IX}\;V}\left(x,\tau \right)=\left(1+\tau \right)/\left(5-4/\left(x_{0}-x+1\right)-\tau \right)$. Here, $x_{0}=\Delta_{c}/\left(2-\Delta_{c}\right)$, with $\left(\Delta_{c},\tau -\Delta_{c}\right)$ denoting the Ising critical point between the Haldane phase and the ${\mathrm{AF}}_{x}$ phase.

Similarly, for the second group, one primary duality transformation connects regime II with regime IV and the other connects regime IV with regime X. First, let us determine scaling entropy $S_{\sigma }^{\mathrm{II}}\left(x,\tau \right)$, with the chosen dominant control parameter x and auxiliary control parameter τ in regime II (cf. Section 11). On the factorizing-field line (γ=1+Δ with −1≤Δ≤0), labelled as iv, the same factorized state occurs as the ground state function, with the ground-state energy density e(Δ,1+Δ)=−(2+Δ)/2. Here, a re-parametrization operation was performed (cf. Appendix O): e(Δ,1+Δ)=m(x)e(x), with x=1+Δ and m(x)=(1+x)/2, resulting in the rescaled ground-state energy density e(x)=−1. As such, a contribution to scaling entropy $S_{\sigma }^{\mathrm{iv}}\left(x\right)$ on the factorizing-field line (γ=1+Δ with −1≤Δ≤0), arising from a multiplying factor m(x), takes a plus sign, as follows from the supplementary rule, given that the rescaled ground-state energy density e(x) is a constant. Given a one-to-one correspondence between the auxiliary control parameter τ in regime II and the dominant control parameter x on the factorizing-field line (γ=1+Δ with −1≤Δ≤0), this re-parametrization operation induces a contribution to scaling entropy $S_{\sigma }^{\mathrm{II}}\left(x,\tau \right)$ in regime II (cf. Appendix O): $S_{\sigma }^{\mathrm{II}}\left(x,\tau \right)=\ln m^{\mathrm{II}}\left(x,\tau \right)$, with $m^{\mathrm{II}}\left(x,\tau \right)=\left(2+\tau \right)/2$, as a result of the continuity requirement for scaling entropy. Second, since the ground-state energy density e(x) monotonically decreases with x on the semi-self-dual line (Δ=0 with 0≤γ≤1), with x=γ in regime II, labelled as v, a contribution to scaling entropy $S_{\sigma }^{\mathrm{VI}}\left(x,\tau \right)$ takes a minus sign to retain consistency with the analogue of the Landauer’s principle at zero temperature. That is, $S_{\sigma }^{\mathrm{VI}}\left(x,\tau \right)=S_{\sigma }^{\mathrm{II}}\left(x,\tau \right)+S_{\sigma }^{\mathrm{VI}\;\mathrm{II}}\left(x,\tau \right)$, where $S_{\sigma }^{\mathrm{VI}\;\mathrm{II}}\left(x,\tau \right)-\ln k_{\mathrm{VI}\;\mathrm{II}}\left(x,\tau \right)$, with $k_{\mathrm{VI}\;\mathrm{II}}\left(x,\tau \right)$ being a multiplying factor $k_{\mathrm{VI}\;\mathrm{II}}\left(x,\tau \right)=2/\left(1+x-\tau \right)$. Third, the ground-state energy density e(x) monotonically increases with x on the semi-self-dual line (γ=1 with −2≤Δ≤0), with x=(2+Δ)/(2−Δ) in regime VI, which is the dual image line of the semi-self-dual line (Δ=0 with 0≤γ≤1), labelled as v. Hence, a contribution to scaling entropy $S_{\sigma }^{X}\left(x,\tau \right)$ in regime X takes a plus sign to retain consistency with the analogue of the Landauer’s principle at zero temperature. That is, $S_{\sigma }^{X}\left(x,\tau \right)=S_{\sigma }^{\mathrm{VI}}\left(x,\tau \right)+S_{\sigma }^{X\;\mathrm{VI}}\left(x,\tau \right)$, where $S_{\sigma }^{X\;\mathrm{VI}}\left(x,\tau \right)=\ln k_{X\;\mathrm{VI}}\left(x,\tau \right)$, with $k_{X\;\mathrm{VI}}\left(x,\tau \right)$ being a multiplying factor $k_{X\;\mathrm{VI}}\left(x,\tau \right)=\left(1+x-\tau \right)/\left(1-x-\tau \right)$.

Similarly, for the third group, one primary duality transformation connects regime III with regime VII and the other connects regime III with regime XI. First, scaling entropy $S_{\sigma }^{\mathrm{III}}\left(x,\tau \right)$ is equal to 0, with the chosen dominant control parameter x and auxiliary control parameter τ in regime III (cf. Section 11). Second, since the ground-state energy density e(x) monotonically decreases with x on the U(1)-symmetric line (γ=0 with $\Delta_{c1}\leq \Delta \leq 1$), labelled as i, with $x=\Delta -\Delta_{c1}$ and on the U(1)-symmetric line (γ=1−Δ with $\Delta_{c0}\leq \Delta \leq 1$), labelled as iii, with $x=\Delta /\left(2-\Delta \right)-\Delta_{c0}/\left(2-\Delta_{c0}\right)$ in regime III, a contribution to scaling entropy $S_{\sigma }^{\mathrm{VII}}\left(x,\tau \right)$ takes a minus sign to retain consistency with the analogue of the Landauer’s principle at zero temperature. That is, $S_{\sigma }^{\mathrm{VII}}\left(x,\tau \right)=S_{\sigma }^{\mathrm{III}}\left(x,\tau \right)+S_{\sigma }^{\mathrm{VII}\;\mathrm{III}}\left(x,\tau \right)$, where $S_{\sigma }^{\mathrm{VII}\;\mathrm{III}}\left(x,\tau \right)=-\ln k_{\mathrm{VII}\;\mathrm{III}}\left(x,\tau \right)$, with $k_{\mathrm{VII}\;\mathrm{III}}\left(x,\tau \right)$ being a multiplying factor $k_{\mathrm{VII}\;\mathrm{III}}\left(x,\tau \right)=2/\left(1+\tau \right)$. Third, from the same reasoning as in regime VII, a contribution to scaling entropy $S_{\sigma }^{\mathrm{XI}}\left(x,\tau \right)$ in regime XI takes a minus sign to retain consistency with the analogue of the Landauer’s principle at zero temperature. That is, $S_{\sigma }^{\mathrm{XI}}\left(x,\tau \right)=S_{\sigma }^{\mathrm{III}}\left(x,\tau \right)+S_{\sigma }^{\mathrm{XI}\;\mathrm{III}}\left(x,\tau \right)$, where $S_{\sigma }^{\mathrm{XI}\;\mathrm{III}}\left(x,\tau \right)=-\ln k_{\mathrm{XI}\;\mathrm{III}}\left(x,\tau \right)$ and $k_{\mathrm{XI}\;\mathrm{III}}\left(x,\tau \right)$ are multiplying factors $k_{\mathrm{XI}\;\mathrm{III}}\left(x,\tau \right)=2/\left(5-4/\left(x+x_{0}+1\right)-\tau \right)$. Here, we remark that it is also legitimate to choose regime XI as a principal regime, with regime VII and regime III as the first and second dual image regimes, respectively. As a result, contributions to scaling entropies $S_{\sigma }^{\mathrm{VII}}\left(x,\tau \right)$ in regime VII and regime III, arising from multiplying factors $k_{\mathrm{VII}\;\mathrm{XI}}\left(x,\tau \right)$ and $k_{\mathrm{III}\;\mathrm{XI}}\left(x,\tau \right)$, respectively, take a plus sign to retain consistency with the analogue of the Landauer’s principle at zero temperature.

Similarly, for the fourth group, one primary duality transformation connects regime IV with regime VIII and the other connects regime VIII with regime XII. First, let us determine scaling entropy $S_{\sigma }^{\mathrm{IV}}\left(x,\tau \right)$ with the chosen dominant control parameter x and auxiliary control parameter τ in regime IV (cf. Section 11). On the characteristic (soft) line ($\gamma =\Delta_{c1}-\Delta$ with $0\leq \Delta \leq \Delta_{c1})$, labelled as vii, a boundary between regime I and regime IV, a re-parametrization operation was performed in the ground state energy density $e(\Delta ,\Delta_{c1}-\Delta )$ (cf. Appendix O): $e\left(\Delta ,\Delta_{c1}-\Delta \right)=m\left(x\right)e\left(x\right)$, with $x=\Delta_{c1}/\left(2-\Delta_{c1}\right)-\Delta /\left(2-\Delta \right)$ and $m\left(x\right)=\left(\Delta_{c1}/\left(2-\Delta_{c1}\right)+1\right)/\left(x+1\right)$. This re-parametrization operation is induced from the re-parametrization operation with $m\left(x\right)=\left(\Delta_{c0}/\left(2-\Delta_{c0}\right)+1\right)/\left(x+1\right)$ on the self-dual line (γ=1−Δ with $0\leq \Delta \leq \Delta_{c0}$), on which the ground state energy density e(Δ,1−Δ) is not monotonic. As such, the rescaled ground-state energy density e(x) after the re-parametrization operation monotonically decreases with x on the characteristic (soft) line ($\gamma =\Delta_{c1}-\Delta$ with $0\leq \Delta \leq \Delta_{c1}$). The continuity requirement for scaling entropy demands to perform a re-parametrization operation in the ground state energy density $e^{\mathrm{IV}}\left(\Delta ,\gamma \right)$ (cf. Appendix O) in regime IV: $e^{\mathrm{IV}}\left(\Delta ,\gamma \right)=m^{\mathrm{IV}}\left(x,\tau \right)e^{\mathrm{IV}}\left(x,\tau \right)$, with $m^{\mathrm{IV}}\left(x,\tau \right)=\left(\tau /\left(2-\tau \right)+1\right)/\left(x+1\right)$, which ensures that the ground state energy density $e^{\mathrm{III}}\left(x,\tau \right)$ monotonically decreases with x for a fixed τ. Hence, a contribution to scaling entropy $S_{\sigma }^{\mathrm{IV}}\left(x,\tau \right)$ in regime IV, arising from a multiplying factor $m^{\mathrm{IV}}\left(x,\tau \right)$, takes a minus sign to retain consistency with the analogue of the Landauer’s principle at zero temperature. That is, $S_{\sigma }^{\mathrm{IV}}\left(x,\tau \right)=-\ln m^{\mathrm{IV}}\left(x,\tau \right)$. Second, since the ground-state energy density e(x) monotonically decreases with x on the semi-self-dual line (Δ=0 with $0\leq \gamma \leq \Delta_{c1}$), with x=γ in regime IV, labelled as viii, a contribution to scaling entropy $S_{\sigma }^{\mathrm{VIII}}\left(x,\tau \right)$ takes a minus sign to retain consistency with the analogue of the Landauer’s principle at zero temperature. That is, $S_{\sigma }^{\mathrm{VIII}}\left(x,\tau \right)=S_{\sigma }^{\mathrm{IV}}\left(x,\tau \right)+S_{\sigma }^{\mathrm{VIII}\;\mathrm{IV}}\left(x,\tau \right)$, where $S_{\sigma }^{\mathrm{VIII}\;\mathrm{IV}}\left(x,\tau \right)=-\ln k_{\mathrm{VIII}\;\mathrm{IV}}\left(x,\tau \right)$, with $k_{\mathrm{VIII}\;\mathrm{IV}}\left(x,\tau \right)$ being a multiplying factor $k_{\mathrm{VIII}\;\mathrm{IV}}\left(x,\tau \right)=2/\left(1+\tau \right)$. Third, the ground-state energy density e(x) on the self-dual line (γ=1 with $2\Delta_{c2}-2\leq \Delta \leq 2$), with x=(2−Δ)/(2+Δ) in regime VIII, which is the dual image line of the semi-self-dual line (Δ=0 with $0\leq \gamma \leq \Delta_{c1}$), labelled as viii, monotonically increases with x. On the characteristic (soft) line ($\gamma =-1+2\Delta_{c2}-\Delta$ with $-2+2\Delta_{c2}\leq \Delta \leq \Delta_{c2}$), labelled as ix, a re-parametrization operation is performed in the ground state energy density $e(\Delta ,\Delta_{c1}-\Delta )$: $e\left(\Delta ,-1+2\Delta_{c2}-\Delta \right)=m_{A}\left(x\right)e_{A}\left(x\right)$, with $x=\Delta_{c1}/\left(2\;-\;\Delta_{c1}\right)\;-\;\left(2\;-\;2\Delta_{c2}+\Delta \right)/\left(-2\;+\;4\Delta_{c2}\;-\;\Delta \right)$ and $m_{A}\left(x\right)=\left(2\;-\;\Delta_{c1}\right)/\left(2\;-\;2x\;+\;\Delta_{c1}x\right)$, to ensure that the rescaled ground-state energy density $e_{A}\left(x\right)$ monotonically increases with x. This re-parametrization operation is induced from the alternative re-parametrization operation, with $m_{A}\left(x\right)=\left(2-\Delta_{c0}\right)/\left(2-2x+\Delta_{c0}x\right)$, on the self-dual line (γ=1−Δ with $0\leq \Delta \leq \Delta_{c0}$), on which the ground state energy density e(Δ,1−Δ) is not monotonic. The existence of such an alternative re-parametrization operation ensures that regime VIII, as the first dual image regime, may be treated as if it was a principal regime, as discussed in Section 2. Hence, a contribution to scaling entropy $S_{\sigma }^{\mathrm{XII}}\left(x,\tau \right)$ in regime XII takes a plus sign to retain consistency with the analogue of the Landauer’s principle at zero temperature. That is, $S_{\sigma }^{\mathrm{XII}}\left(x,\tau \right)=S_{\sigma }^{\mathrm{VIII}}\left(x,\tau \right)+S_{\sigma }^{\mathrm{XII}\;\mathrm{VIII}}\left(x,\tau \right)$, where $S_{\sigma }^{\mathrm{XII}\;\mathrm{VIII}}\left(x,\tau \right)=\ln k_{\mathrm{XII}\;\mathrm{VIII}}\left(x,\tau \right)$, with $k_{\mathrm{XII}\;\mathrm{VIII}}\left(x,\tau \right)$ being a multiplying factor $k_{\mathrm{XII}\;\mathrm{VIII}}\left(x,\tau \right)=\left(1+\tau \right)/\left(5-4/\left(x_{1}-x+1\right)-\tau \right)$. Here, $x_{1}=\tau /\left(2-\tau \right)$.

Alternatively, one may treat regime I and regime IV as a composite regime I+IV, since they are separated by the soft line ($\gamma =\Delta_{c1}-\Delta$ with $0\leq \Delta \leq \Delta_{c1}$), labelled as vi. As a result of dualities induced from the symmetric group $S_{3}$, two other composite regimes emerge as its dual image composite regimes, labelled as V+VIII and IX+XII, respectively. As such, the principal composite regime I+IV and the first dual image composite regime V+VIII share the self-dual line (γ=1−Δ with $0\leq \Delta \leq \Delta_{c0}$), and the first dual image composite regime V+VIII and the second dual image composite regime IX+XII share the self-dual line (γ=−1+Δ with $1\leq \Delta \leq \Delta_{c2}$). Therefore, according to our discussions in Section 2, the three composite regimes I+IV, V+VIII and IX+XII constitute an example for the first situation. As a consequence, regime V and regime VIII share the same minus sign, whereas regime IX and regime XII share the same plus sign, consistent with the above discussions.

To ensure the continuity requirement for scaling entropy on the line of the Ising critical points between the Haldane phase and the ${\mathrm{AF}}_{z}$ phase, $S_{\sigma }^{\mathrm{III}}\left(x,\tau \right)$ is shifted to $S_{\sigma }^{\mathrm{III}}\left(x,\tau \right)-S_{s}^{\mathrm{III}}\left(\tau \right)$, with $S_{s}^{\mathrm{III}}\left(\tau \right)=S_{\sigma }^{\mathrm{III}}\left(0,\tau \right)-S_{\sigma }^{I}\left(0,\tau \right)$; $S_{\sigma }^{\mathrm{VII}}\left(x,\tau \right)$ is shifted to $S_{\sigma }^{\mathrm{VII}}\left(x,\tau \right)-S_{s}^{\mathrm{VII}}\left(\tau \right)$, with $S_{s}^{\mathrm{VII}}\left(\tau \right)=S_{\sigma }^{\mathrm{VII}}\left(0,\tau \right)-S_{\sigma }^{V}\left(0,\tau \right)$; $S_{\sigma }^{\mathrm{XI}}\left(x,\tau \right)$ is shifted to $S_{\sigma }^{\mathrm{XI}}\left(x,\tau \right)-S_{s}^{\mathrm{XI}}\left(\tau \right)$, with $S_{s}^{\mathrm{XI}}\left(\tau \right)=S_{\sigma }^{\mathrm{XI}}\left(0,\tau \right)-S_{\sigma }^{\mathrm{IX}}\left(0,\tau \right)$.

Once scaling entropy $S_{\sigma }^{\beta }\left(x,\tau \right)$ is determined in each of the twelve regimes for γ<1, scaling entropy $S_{\sigma }^{\beta }\left(\Delta ,\gamma \right)$ (β= I, II, III, IV, V, VI, VII, VIII, IX, X, XI, and XII) follows. That is, $S_{\sigma }^{\beta }\left(\Delta ,\gamma \right)\equiv S_{f}^{\beta }\left(x,\tau \right)$, when we move from x and τ to Δ and γ, meaning that x and τ are regarded as functions of Δ and γ. Then, scaling entropy $S_{\sigma }^{\beta }\left(\Delta ,\gamma \right)$ (β= XIII, XIV, XV, XVI, XVII, XVIII, XIX, XX, XXI, XXII, XXIII and XXIV) follows from the primary duality transformation induced by the cyclic group $Z_{2}$, with a multiplying factor k(Δ,γ)=γ. As follows from the rule of thumb, at the exterior of the Haldane phase, scaling entropy takes the same sign as that on the semi-self dual line (Δ=0 with γ>1), which is dual to (Δ=0 with 0<γ<1), whereas in the interior of the Haldane phase, scaling entropy takes the same sign as that on the U(1)-symmetric line (γ=−1−Δ with $\Delta <-\Delta_{c2}/\left(-1+\Delta_{c2}\right)$), which is dual to the U(1)-symmetric line (γ=−1+Δ with $1<\Delta <\Delta_{c2}$). That is, a contribution to scaling entropy $S_{f}^{\beta }\left(\Delta ,\gamma \right)$, with β= XIII, XIV, XVI, XVII, XVIII, XX, XXI, XXII, and XXIV, takes the form $S_{\sigma }\left(\Delta ,\gamma \right)=-\ln\gamma$ for γ>1. Here, the minus sign is chosen to retain consistency with the analogue of Landauer’s principle at zero temperature, since the ground-state energy density monotonically decreases with x on the semi-self dual line (Δ=0), where x was chosen to be γ, as γ varies from 0 to 1. In contrast, a contribution to scaling entropy $S_{f}^{\beta }\left(\Delta ,\gamma \right)$, with β= XV, XIX, and XXIII, takes the form $S_{\sigma }\left(\Delta ,\gamma \right)=\ln\gamma$ for γ>1. Here, the plus sign is chosen to retain consistency with the analogue of the Landauer’s principle at zero temperature, since the ground-state energy density e(x) on the U(1)-symmetric line (γ=−1+Δ with $1<\Delta <\Delta_{c2}$) in regime VII or regime XI monotonically increases with x, where x was chosen to be −1+2/(1+γ), as γ varies from $-1+\Delta_{c2}$ to 0. We remark that regime XI is a proper choice as a principal regime, as far as the determination of the sign in scaling entropy for the primary duality transformation induced from the cyclic group $Z_{2}$ is concerned, as discussed in Section 2. This is due to the fact that the one-to-one correspondence between the auxiliary control parameter τ in regime III and the dominant control parameter x, with $x=\Delta -\Delta_{c1}$, on the U(1)-symmetric line (γ=0 with $\Delta_{c1}\leq \Delta \leq 1$), labelled as i, is not retained after the primary duality transformation induced from the cyclic group $Z_{2}$ is performed, in contrast to the U(1)-symmetric line (γ=−1+Δ with $1<\Delta <\Delta_{c2}$). Physically, this is consistent with the fact that two distinct types of the universality classes, the Gaussian transitions and the KT transitions, are separated by the lines of the Ising critical points. Hence, we have $S_{\sigma }^{\mathrm{XIII}}\left(\Delta ,\gamma \right)=S_{\sigma }^{I}\left(-\Delta /\gamma ,1/\gamma \right)-\ln\gamma$; $S_{\sigma }^{\mathrm{XIV}}\left(\Delta ,\gamma \right)=S_{\sigma }^{\mathrm{II}}\left(-\Delta /\gamma ,1/\gamma \right)-\ln\gamma$; $S_{\sigma }^{\mathrm{XV}}\left(\Delta ,\gamma \right)=S_{\sigma }^{\mathrm{III}}\left(-\Delta /\gamma ,1/\gamma \right)+\ln\gamma$; $S_{\sigma }^{\mathrm{XVI}}\left(\Delta ,\gamma \right)=S_{\sigma }^{\mathrm{IV}}\left(-\Delta /\gamma ,1/\gamma \right)-\ln\gamma$; $S_{\sigma }^{\mathrm{XVII}}\left(\Delta ,\gamma \right)=S_{\sigma }^{V}\left(-\Delta /\gamma ,1/\gamma \right)-\ln\gamma$; $S_{\sigma }^{\mathrm{XVIII}}\left(\Delta ,\gamma \right)=S_{\sigma }^{\mathrm{VI}}\left(-\Delta /\gamma ,1/\gamma \right)-\ln\gamma$; $S_{\sigma }^{\mathrm{XIX}}\left(\Delta ,\gamma \right)$ $=S_{\sigma }^{\mathrm{VII}}\left(-\Delta /\gamma ,1/\gamma \right)+\ln\gamma$; $S_{\sigma }^{\mathrm{XX}}\left(\Delta ,\gamma \right)=S_{\sigma }^{\mathrm{VIII}}\left(-\Delta /\gamma ,1/\gamma \right)-\ln\gamma$; $S_{\sigma }^{\mathrm{XXI}}\left(\Delta ,\gamma \right)$ $=S_{\sigma }^{\mathrm{IX}}\left(-\Delta /\gamma ,1/\gamma \right)-\ln\gamma$; $S_{\sigma }^{\mathrm{XXII}}\left(\Delta ,\gamma \right)=S_{\sigma }^{X}\left(-\Delta /\gamma ,1/\gamma \right)-\ln\gamma$; $S_{\sigma }^{\mathrm{XXIII}}\left(\Delta ,\gamma \right)$ $=S_{\sigma }^{\mathrm{XI}}\left(-\Delta /\gamma ,1/\gamma \right)+\ln\gamma$; $S_{\sigma }^{\mathrm{XXIV}}\left(\Delta ,\gamma \right)=S_{\sigma }^{\mathrm{XII}}\left(-\Delta /\gamma ,1/\gamma \right)-\ln\gamma$.

To ensure the continuity requirement for scaling entropy, $S_{\sigma }^{\mathrm{XV}}\left(x,\tau \right)$ is shifted to $S_{\sigma }^{\mathrm{XV}}\left(x,\tau \right)-S_{s}^{\mathrm{XV}}\left(\tau \right)$, with $S_{s}^{\mathrm{XV}}\left(\tau \right)=S_{\sigma }^{\mathrm{XV}}\left(0,\tau \right)-S_{\sigma }^{\mathrm{XIII}}\left(0,\tau \right)$; $S_{\sigma }^{\mathrm{XIX}}\left(x,\tau \right)$ is shifted to $S_{\sigma }^{\mathrm{XIX}}\left(x,\tau \right)$ $-S_{s}^{\mathrm{XIX}}\left(\tau \right)$, with $S_{s}^{\mathrm{XIX}}\left(\tau \right)=S_{\sigma }^{\mathrm{XIX}}\left(0,\tau \right)-S_{\sigma }^{\mathrm{XVII}}\left(0,\tau \right)$; $S_{\sigma }^{\mathrm{XXIII}}\left(x,\tau \right)$ is shifted to $S_{\sigma }^{\mathrm{XXIII}}\left(x,\tau \right)$ $-S_{s}^{\mathrm{XXIII}}\left(\tau \right)$, with $S_{s}^{\mathrm{XXIII}}\left(\tau \right)=S_{\sigma }^{\mathrm{XXIII}}\left(0,\tau \right)-S_{\sigma }^{\mathrm{XXI}}\left(0,\tau \right)$, on the line of the Ising critical points between the Haldane phase and the ${\mathrm{AF}}_{z}$ phase.

### Appendix G.3. The Spin-1/2 Kitaev Model on a Honeycomb Lattice

For the spin-1/2 Kitaev model on a honeycomb lattice, there are twelve regimes that are separated into two groups, with six regimes in each group being dual to each other: regime I, regime III, regime V, regime VII, regime IX, and regime XI are dual to each other, whereas regime II, regime IV, regime VI, regime VIII, regime X, and regime XII are dual to each other. Here, we chose regime I and regime II as the two principal regimes. As it turns out, duality transformations are induced from the symmetric group $S_{3}$ with respect to x, y, and z. In the $\left(J_{x},J_{y}\right)$ plane, the subgroup $Z_{2}^{+}$, generated from an exchange between x and y, induces a symmetric transformation with $J_{x}↔J_{y}$. Therefore, only six regimes need to be addressed, with regime I, regime III, and regime V being in one group, and with regime II, regime IV and regime VI being in the other group. Given that the extent of the control parameters $\left(J_{x},J_{y}\right)$ in regime III is not finite, regime I, regime III, and regime V constitute an example for the second situation, as follows from our discussions in Section 2. In addition, since the monotonicity of the ground-state energy density on the two characteristic lines in regime IV is not consistent, regime II, regime IV, and regime VI constitute an example for the second situation, as follows from our discussions in Section 2.

Specifically, one primary duality transformation connects regime I with regime III and the other connects regime I with regime V. First, scaling entropy $S_{\sigma }^{I}\left(x,\tau \right)$ is equal to 0, with the chosen dominant control parameter x and auxiliary control parameter τ in regime I (cf. Section 12). Second, since the ground-state energy density $e^{i}\left(x\right)$ on the $Z_{2}$-symmetric line ($J_{x}=0$ with $0<J_{y}<1$), labelled as i, monotonically increases with $x=1-J_{y}$, and the ground-state energy density $e^{\mathrm{ii}}\left(x\right)$ on the $Z_{2}$-symmetric line ($J_{y}=J_{x}$ with $0<J_{x}<1/2$), labelled as ii, monotonically increases with $x=-J_{x}$, a contribution to scaling entropy $S_{\sigma }^{\mathrm{III}}\left(x,\tau \right)$ takes a plus sign to retain consistency with the analogue of the Landauer’s principle at zero temperature. That is, $S_{\sigma }^{\mathrm{III}}\left(x,\tau \right)=S_{\sigma }^{I}\left(x,\tau \right)+S_{\sigma }^{\mathrm{III}\;I}\left(x,\tau \right)$, where $S_{\sigma }^{\mathrm{III}\;I}\left(x,\tau \right)=\ln k_{\mathrm{III}\;I}\left(x,\tau \right)$, with $k_{\mathrm{III}\;I}\left(x,\tau \right)$ being a multiplying factor $k_{\mathrm{III}\;I}\left(x,\tau \right)=1/\left[\tau /\left(\tau +1\right)-x\sin\arctan\tau \right]$. Third, from the same reasoning as in regime III, a contribution to scaling entropy $S_{\sigma }^{\mathrm{IV}}\left(x,\tau \right)$ in regime V takes a plus sign to retain consistency with the analogue of the Landauer’s principle at zero temperature. That is, $S_{\sigma }^{V}\left(x,\tau \right)=S_{\sigma }^{I}\left(x,\tau \right)+S_{\sigma }^{V\;I}\left(x,\tau \right)$, where $S_{\sigma }^{V\;I}\left(x,\tau \right)=\ln k_{V\;I}\left(x,\tau \right)$, with $k_{V\;I}\left(x,\tau \right)$ being a multiplying factor $k_{V\;I}\left(x,\tau \right)=1/\left[1/\left(\tau +1\right)-x\cos\arctan\tau \right]$, in regime V.

Similarly, one primary duality transformation connects regime II with regime IV and the other connects regime II with regime VI. First, scaling entropy $S_{\sigma }^{\mathrm{II}}\left(x,\tau \right)$ is equal to 0, with the chosen dominant control parameter x and auxiliary control parameter τ in regime II (cf. Section 12). Second, since the ground-state energy density $e^{\mathrm{iii}}\left(x\right)$ on the $Z_{2}$-symmetric line ($J_{y}=1$ with $0<J_{x}<1$), labelled as iii, monotonically decreases with $x=J_{x}$ and the ground-state energy density $e^{\mathrm{ii}}\left(x\right)$ on the $Z_{2}$-symmetric line ($J_{y}=J_{x}$ with $1/2<J_{x}<1$), labelled as ii, monotonically decreases with $x=J_{x}$, a contribution to scaling entropy $S_{\sigma }^{\mathrm{IV}}\left(x,\tau \right)$ takes a minus sign to retain consistency with the analogue of the Landauer’s principle at zero temperature. That is, $S_{\sigma }^{\mathrm{IV}}\left(x,\tau \right)=S_{\sigma }^{\mathrm{II}}\left(x,\tau \right)+S_{\sigma }^{\mathrm{IV}\;\mathrm{II}}\left(x,\tau \right)$, where $S_{\sigma }^{\mathrm{IV}\;\mathrm{II}}\left(x,\tau \right)=-\ln k_{\mathrm{IV}\;\mathrm{II}}\left(x,\tau \right)$, with $k_{\mathrm{IV}\;\mathrm{II}}\left(x,\tau \right)$ being a multiplying factor $k_{\mathrm{IV}\;\mathrm{II}}\left(x,\tau \right)=1/\left(\tau /\left(\tau +1\right)+x\sin\arctan\tau \right)$. Third, from the same reasoning as in regime IV, a contribution to scaling entropy $S_{\sigma }^{\mathrm{VI}}\left(x,\tau \right)$ in regime VI takes a minus sign to retain consistency with the analogue of the Landauer’s principle at zero temperature. That is, $S_{\sigma }^{\mathrm{VI}}\left(x,\tau \right)=S_{\sigma }^{\mathrm{II}}\left(x,\tau \right)+S_{\sigma }^{\mathrm{VI}\;\mathrm{II}}\left(x,\tau \right)$, where $S_{\sigma }^{\mathrm{VI}\;\mathrm{II}}\left(x,\tau \right)=-\ln k_{\mathrm{VI}\;\mathrm{II}}\left(x,\tau \right)$, with $k_{\mathrm{VI}\;\mathrm{II}}\left(x,\tau \right)$ being a multiplying factor $k_{\mathrm{VI}\;\mathrm{II}}\left(x,\tau \right)=1/\left(1/\left(\tau +1\right)+x\cos\arctan\tau \right)$.

To ensure the continuity requirement for scaling entropy, $S_{\sigma }^{\mathrm{III}}\left(x,\tau \right)$ is shifted to $S_{\sigma }^{\mathrm{III}}\left(x,\tau \right)-S_{s}^{\mathrm{III}}\left(\tau \right)$, with $S_{s}^{\mathrm{III}}\left(\tau \right)=S_{\sigma }^{\mathrm{III}}\left(0,\tau \right)-S_{\sigma }^{\mathrm{IV}}\left(0,\tau \right)$, and $S_{\sigma }^{V}\left(x,\tau \right)$ is shifted to $S_{\sigma }^{V}\left(x,\tau \right)-S_{s}^{V}\left(\tau \right)$, with $S_{s}^{V}\left(\tau \right)=S_{\sigma }^{V}\left(0,\tau \right)-S_{\sigma }^{\mathrm{VI}}\left(0,\tau \right)$, on the line of critical points ($J_{y}=1+J_{x}$ with $J_{x}>0$).

## Appendix H. A Scaling Analysis of Fidelity Entropy S(x) in the Vicinity of a Critical/Transition Point $x_{c}$ and Beyond

### Appendix H.1. A Scaling Analysis of Fidelity Entropy S(x) in the Vicinity of a Critical/Transition Point $x_{c}$

Here, we perform a scaling analysis of fidelity entropy S(x) in the vicinity of a critical/transition point $x=x_{c}$.

For the sake of simplicity, we denote f(x,y)=lnd(x,y), with d(x,y) being the ground-state fidelity per lattice site. Then, fidelity entropy S(x) takes the following form: $S\left(x\right)=-2\int_{x_{c}}^{x}f(x,y)dy+S_{0}$. We expand f(x,y) into a Taylor series at y=x. Keeping the second-order term, we have the following

(A24) $$f\left(x,y\right)=f\left(x,x\right)+\frac{\partial f(x,y)}{\partial y}{|}_{y=x}\left(y-x\right)+\frac{1}{2}\frac{\partial^{2}f(x,y)}{\partial y^{2}}{|}_{y=x}{\left(y-x\right)}^{2}+O\left({\left(y-x\right)}^{3}\right).$$

Since f(x,x)=0 and $\frac{\partial f(x,y)}{\partial y}{|}_{y=x}=0$, we have the following

(A25) $$f\left(x,y\right)=\frac{1}{2}\frac{\partial^{2}f(x,y)}{\partial y^{2}}{|}_{y=x}{\left(y-x\right)}^{2}+O\left({\left(y-x\right)}^{3}\right).$$

Therefore, the leading contribution to fidelity entropy S(x) is from the second-order (partial) derivative of f(x,y) with respect to y in the vicinity of a critical point $x=x_{c}$

(A26) $$S\left(x\right)=-\frac{1}{3}\frac{\partial^{2}f(x,y)}{\partial y^{2}}{|}_{y=x}{\left(x-x_{c}\right)}^{3}.$$

For a quantum many-body system on a d-dimensional lattice with the rotational invariance, the second-order derivative of f(x,y) with respect to y at y=x is related with the critical exponent for the correlation length ν [226]

(A27) $$\frac{\partial^{2}f(x,y)}{\partial y^{2}}{|}_{y=x}∼{\left(x-x_{c}\right)}^{d\nu -2},$$

Therefore, when x is close to a critical point $x_{c}$, fidelity entropy S(x) scales as follows

(A28) $$S\left(x\right)∼{\left(x-x_{c}\right)}^{d\nu +1}.$$

Generically, for a quantum many-body system on a d-dimensional lattice without the rotational invariance, we need to introduce $\nu_{\|}$ and $\nu_{⊥}$ to denote critical exponents for the correlation length in the two perpendicular directions, with m and d−m being the effective dimensions, respectively. Then, we have the following

(A29) $$S\left(x\right)∼{\left(x-x_{c}\right)}^{m\nu_{\|}+\left(d-m\right)\nu_{⊥}+1}.$$

As an illustrative example, we consider the transverse-field quantum Ising model and the spin-1/2 Kitaev model on a honeycomb lattice. For the transverse-field quantum Ising model, we have d=1 and ν=1. Hence, $S\left(x\right)∼{\left(x-x_{c}\right)}^{2}$. For the spin-1/2 Kitaev model on a honeycomb lattice, we have d=2, m=1, $\nu_{\|}=1$, and ⊥=1/2 [227]. Hence, $S\left(x\right)∼{\left(x-x_{c}\right)}^{5/2}$. Our numerical simulations confirm the predictions from this scaling analysis.

### Appendix H.2. A Scaling Analysis of the Ground-State Energy Density e(λ,γ) Close to a Gaussian Critical Point for the Quantum Spin-1/2 XY Model

Here, we perform a scaling analysis of the ground-state energy density e(λ,γ) close to a Gaussian critical point for the quantum spin-1/2 XY model. For this model, Gaussian phase transitions occur at γ=0 and −1<λ<1. As an exactly solvable model, the ground-state energy density e(λ,γ) is known to be [116,118]

(A30) $$e\left(\lambda ,\gamma \right)=-1/\pi \int_{0}^{\pi }\sqrt{{\left(\cos\alpha -\lambda \right)}^{2}+{\left(\gamma \sin\alpha \right)}^{2}}d\alpha .$$

For the sake of brevity, we denote x≡cosα. Then, the second-order derivative of e(λ,γ) with respect to γ takes the following form

(A31) $$\frac{\partial^{2}e\left(\lambda ,\gamma \right)}{\partial \gamma^{2}}=1/\pi \int_{1}^{-1}\frac{\sqrt{1-x^{2}}}{\sqrt{{\left(x-\lambda \right)}^{2}+{\left(\gamma \sqrt{1-x^{2}}\right)}^{2}}}dx.$$

As it turns out, the integral diverges when x→λ and γ→0. To determine its leading divergent behavior, we divide this integral into four parts, i.e., $\int_{1}^{-1}\;=\;\int_{1}^{\lambda \;+\;\delta }\;+\;\int_{\lambda \;+\;\delta }^{\lambda }\;+\;\int_{\lambda }^{\lambda \;-\;\delta }\;+\;\int_{\lambda \;-\;\delta }^{-1}$. Given the first and last parts are regular, we only need to consider the second and third parts. Since δ→0, for these two parts, the integrand in the integral (A31) is simplified into the following

(A32) $$\frac{\sqrt{1-x^{2}}}{\sqrt{{\left(x-\lambda \right)}^{2}+{\left(\gamma \sqrt{1-x^{2}}\right)}^{2}}}\to \frac{\sqrt{1-\lambda^{2}}}{\sqrt{{\left(x-\lambda \right)}^{2}+{\left(\gamma \sqrt{1-\lambda^{2}}\right)}^{2}}}.$$

Hence, the leading contribution from the second and third parts is as follows

(A33) $$\frac{\partial^{2}e\left(\lambda ,\gamma \right)}{\partial \gamma^{2}}∼\frac{2\sqrt{1-\lambda^{2}}}{\pi }\ln\gamma +\cdots .$$

We have numerically confirmed this scaling analysis.

## Appendix I. A Universal Logarithmic Scaling of the Entanglement Entropy S(n) with a Block Size n for Scale-Invariant States Arising from Spontaneous Symmetry-Breaking with Type-B Goldstone Modes

In this Appendix, we restrict ourselves in considering quantum many-body systems in one spatial dimension. As argued in Ref. [102], for a scale-invariant state, the entanglement entropy S(n) scales logarithmically with a block size n:

(A34) $$S\left(n\right)=\frac{N_{B}}{2}\ln n+S_{0},$$

where $N_{B}$ is the number of the type-B GMs, and $S_{0}$ is an additive contribution to the entanglement entropy, which is non-universal. In addition, a field-theoretic approach [186] predicts the following:

(A35) $$S\left(n\right)=\frac{d_{f}}{2}\ln n+S_{0},$$

with $d_{f}$ being the fractal dimension. Therefore, we are able to identify the fractal dimension $d_{f}$ with the number of the type-B GMs: $d_{f}=N_{B}$, for highly degenerate ground states arising from SSB with a type-B GM.

Historically, the pursuit for a proper classification of the GMs [105,106,176,177,178,180,181,182,183,184] culminated in the introduction of the type-A and type-B GMs [105,181], based on a previous observation made by Nambu [179]. In this classification, the so-called Watanabe–Brauner matrix [180] plays a crucial role. As a result, when the symmetry group G is spontaneously broken into H, the counting rule for the GMs may be formulated as follows:

(A36) $$N_{A}+2N_{B}=N_{\mathrm{BG}},$$

where $N_{A}$ is the number of the type-A GMs, and $N_{\mathrm{BG}}$ is equal to the dimension of the coset space G/H. Note that SSB with a type-A GM is forbidden in one spatial dimension, as a result of the Mermin–Wagner-Coleman theorem [103], whereas SSB with a type-B GM survives quantum fluctuations even in one spatial dimension.

It seems appropriate to mention that this formalism for scale-invariant states is suitable even for PT transitions in a restricted sense, if one only is concerned about the minimum entanglement entropy, although they are usually characterized as a commensurate–incommensurate transition in the literature [63,64]. In fact, PT transitions occur as a result of the level crossings due to the presence of a conserved U(1) generator in the Hamiltonian (cf. Section 7 and Section 10 for an illustrative example). In this sense, PT transitions are protected by the U(1) symmetry. We remark that the entanglement entropy is not well-defined at a PT transition point, with its value depending on how the transition point is approached, as shown in Ref. [120] for the quantum spin-1/2 XY model. Note that the minimum value is achieved when the PT transition point is approached along the U(1)-symmetric line. According to the counting rule (A36), both $N_{A}=0$ and $N_{B}=0$, given $N_{\mathrm{BG}}=0$. As a consequence, the entanglement entropy S(n) is simply zero, consistent with the fact that the non-degenerate ground state is unentangled at a PT transition point. This is in sharp contrast to the SU(N+1) FM model and the SU(2) CF phase [102], which feature highly degenerate and highly entangled ground states arising from SSB with type-B GMs. In other words, a PT transition point offers a trivial example for scale-invariant states.

A similar argument also applies to the IC transitions, which interpolate between the KT transitions and PT transitions for the quantum XXZ model in a magnetic field, if one only concerns about the minimum entanglement entropy. A notable difference between the IC transitions and the PT transitions is that the ground states at the transition points are entangled but saturated. Therefore, the scaling relations (A34) and (A35) are still valid, with $d_{f}=N_{B}=0$. In addition, the counting rule (A36) is satisfied, with both $N_{A}=0$ and $N_{B}=0$, and $N_{\mathrm{BG}}=0$. Here, we anticipate that the entanglement entropy is also not well-defined at an IC transition point, with its value depending on how the transition point is approached. However, such a ground state is not scale-invariant due to the fact that the minimum entanglement entropy is nonzero.

We remark that highly degenerate and highly entangled ground states arising from SSB with a type-B GM provide a specific example for scale-invariant quantum states (cf. a speculation that scale invariance implies conformal invariance [228]).

## Appendix J. The Bond-Centered Nonlocal Order Parameter for the Symmetry-Protected Topological Phases and the Site-Centered Nonlocal Order Parameter for the Symmetry-Protected Trivial Phases

In this Appendix, we recall the definition of the bond-centered nonlocal order parameter for the SPT phases and the definition of the site-centered nonlocal order parameter for the SPt phases and describe an efficient method for computing them in the context of tensor network algorithms [39,40,41,46,47,48,67,68,69].

A ground-state wave function |ψ〉, generated from the iTEBD algorithm [46,47,48] and the iDMRG algorithm [67,68,69], is expressed in terms of a matrix-product state representation, which is invariant under the operation of the two-site translation. The bond-centered non-local order parameter $O_{2n}$ is defined in terms of the bond-centered inversion $I_{\left(1,2n\right)}$, which is the inversion on a segment from 1 to 2n

(A37) $$\begin{array}{c} O_{2n}\propto \langle \psi |I_{\left(1,2n\right)}|\psi \rangle . \end{array}$$

A graphical representation of the bond-centered non-local order parameter $O_{2n}$ is shown in Figure A8i, which is the overlap of a ground-state wave function ψ with its twisted version, which results from the action of the bond-centered inversion on a segment from 1 to 2n. Then, the overlap is untwisted by reversing the segment by means of the unitary transformations $U_{I,A}$ and $U_{I,B}$, as shown in Figure A8ii. Here, $U_{I,A}$ and $U_{I,B}$ are unitary transformations, generated from the action of the bond-centered inversion $I_{\left(1,2n\right)}$ on |ψ〉 in a matrix-product state representation: $\Gamma_{I,A}^{T}=\exp i\theta_{I,A}U_{I,A}^{†}\Gamma_{I,A}U_{I,B}$ and $\Gamma_{I,B}^{T}$ $=\exp i\theta_{I,B}U_{I,B}^{†}\Gamma_{I,B}U_{I,A}$, with $\theta_{I,A}$ and $\theta_{I,B}$ being the phase factors. When n becomes large enough, we only need to keep the dominant eigenvector of the transfer matrix E, which has been defined in Appendix D. For the segment consisting of n bonds of the type A−B, we have the following

(A38) $$\begin{array}{c} O=\frac{\mathrm{Tr}(U_{I,A}^{T}U_{I,A}^{†}\lambda_{B}^{4})}{\mathrm{Tr}(\lambda_{B}^{4})}. \end{array}$$

For the segment consisting of n bonds of the type B−A, we have the following

(A39) $$\begin{array}{c} O=\frac{\mathrm{Tr}(U_{I,B}^{T}U_{I,B}^{†}\lambda_{A}^{4})}{\mathrm{Tr}(\lambda_{A}^{4})}. \end{array}$$

The site-centered non-local order parameter for the SPt phases is defined through a combined operation I∗R of the site-centered inversion symmetry $I_{\left(1,2n+1\right)}$ with a π-rotation around the α-axis in the spin space: $R_{\alpha }\left(\pi \right)=\exp\left(i\pi \sum_{j=1}^{2n+1}S_{j}^{\alpha }\right)$, where $S^{\alpha }$ denote the spin-s matrices, with α=x,y, and z [113,114]

(A40) $$\begin{array}{c} K_{2n+1,\alpha }\propto \langle \psi |I_{\left(1,2n+1\right)}*R_{\alpha }\left(\pi \right)|\psi \rangle . \end{array}$$

A graphical representation of the site-centered non-local order parameter $K_{2n+1,\alpha }$ is shown in Figure A9i, which is the overlap of a ground-state wave function ψ with its twisted version, which results from the action of the combined operation I∗R. Then, the overlap is untwisted by reversing the segment by means of unitary transformations $U_{I*R,A}$ and $U_{I*R,B}$, as shown in Figure A9ii. Here, $U_{I*R,A}$ and $U_{I*R,B}$ are unitary transformations, generated from the action of the combined operation I∗R on |ψ〉 in a matrix- product state representation: $\Gamma_{I*R,A}^{T}=\exp i\theta_{I*R,A}U_{I*R,A}^{†}\Gamma_{I*R,A}U_{I*R,B}$ and $\Gamma_{I*R,B}^{T}=\exp i\theta_{I*R,B}U_{I*R,B}^{†}\Gamma_{I*R,B}U_{I*R,A}$, with $\theta_{I*R,A}$ and $\theta_{I*R,B}$ being the phase factors. When n becomes large enough, we only need to keep the dominant eigenvector of the transfer matrix E. If the site-centered inversion is carried out with respect to an A-site, then it yields the following

(A41) $$\begin{array}{c} K_{\alpha }=\frac{\mathrm{Tr}(U_{I*R,B}^{T}U_{I,A}^{†}\lambda_{A}^{2}\lambda_{B}^{2})}{\mathrm{Tr}(\lambda_{A}^{2}\lambda_{B}^{2})}. \end{array}$$

If the site-centered inversion is carried out with respect to a B-site, then it yields the following

(A42) $$\begin{array}{c} K_{\alpha }=\frac{\mathrm{Tr}(U_{I*R,A}^{T}U_{I,B}^{†}\lambda_{B}^{2}\lambda_{A}^{2})}{\mathrm{Tr}(\lambda_{B}^{2}\lambda_{A}^{2})}. \end{array}$$

## Appendix K. Fidelity Entropy $S_{f}\left(\lambda ,\gamma \right)$, Fidelity Temperature $T_{f}\left(\lambda ,\gamma \right)$, and Fidelity Internal Energy $U_{f}\left(\lambda ,\gamma \right)$ for the Quantum Spin-1/2 XY Model

In this Appendix, we present mathematical details about fidelity entropy $S_{f}\left(\lambda ,\gamma \right)$, fidelity temperature $T_{f}\left(\lambda ,\gamma \right)$, and fidelity internal energy $U_{f}\left(\lambda ,\gamma \right)$ for the quantum spin-1/2 XY model. For this model, the Hamiltonian (1) is symmetrical under γ↔−γ and λ↔−λ. Therefore, we may restrict ourselves to the region defined by λ≥0 and γ≥0.

There are two dualities that occur on the two characteristic lines (γ=1 and λ=0):

(i) If γ=1, then the Hamiltonian (1) is reduced to the transverse-field quantum Ising model $H\left(\lambda ,1\right)=-\sum_{i}\left(\sigma_{i}^{x}\sigma_{i+1}^{x}+\lambda \sigma_{i}^{z}\right)$. Hence, under the Kramers–Wannier unitary transformation $U_{1}$: $\Pi_{j<i}\sigma_{j}^{z}\to \tau_{i}^{x}\tau_{i+1}^{x}$, and $\sigma_{i}^{x}\sigma_{i+1}^{x}\to \tau_{i}^{z}$, we have H(λ,1) $=k^{\prime }\left(\lambda^{\prime }\right)U_{1}H\left(\lambda^{\prime },1\right)U_{1}^{†}$, with $\lambda^{\prime }=1/\lambda$ and $k^{\prime }\left(\lambda^{\prime }\right)=1/\lambda^{\prime }$. The self-dual point is located at λ=1.
(ii) If λ=0, then the Hamiltonian (1) is simplified to $H\left(0,\gamma \right)=-1/2\sum_{i}[\left(1+\gamma \right)\sigma_{i}^{x}\sigma_{i+1}^{x}$ $+\left(1-\gamma \right)\sigma_{i}^{y}\sigma_{i+1}^{y}]$. Under a unitary transformation $U_{2}$: $\sigma_{2i}^{x}\to \sigma_{2i}^{x}$, $\sigma_{2i+1}^{x}\to \sigma_{2i+1}^{x}$, $\sigma_{2i}^{y}\to \sigma_{2i}^{y}$, $\sigma_{2i+1}^{y}\to -\sigma_{2i+1}^{y}$, $\sigma_{2i}^{z}\to \sigma_{2i}^{z}$, and $\sigma_{2i+1}^{z}\to -\sigma_{2i+1}^{z}$, we have H(0,γ) $=k^{\prime }\left(\gamma^{\prime }\right)U_{2}H\left(0,\gamma^{\prime }\right)U_{2}^{†}$, with $\gamma^{\prime }=1/\gamma$ and $k^{\prime }\left(\gamma^{\prime }\right)=1/\gamma^{\prime }$. The self-dual point is located at γ=1.

The presence of the self-dual points makes it possible to divide two dual lines into four parts: (i) γ=1 with $0\leq \lambda <\lambda_{c}=1$; (ii) γ=1 with $\lambda >\lambda_{c}=1$; (iii) λ=0 with 0<γ≤1; (iv) λ=0 with γ≥1. Here, part i and part iii are two principal parts, whereas part ii and part iv are two non-principal parts that are dual to part i and part iii, respectively. In addition, the disordered circle $\gamma^{2}+\lambda^{2}=1$, featuring unentangled (factorized) ground states [77,78,79,80,81,82], constitutes an additional characteristic line. That is, it is a factorizing-field line. This is the third principal part.

The consideration of the dualities and factorizing fields allows us to separate the entire region, defined by γ>0 and λ>0, into five different principal regimes: (I) the regime inside the disordered circle, with 0<λ<1 and $0<\gamma <\sqrt{1-\lambda^{2}}$; (II) the regime outside the disordered circle, with 0<λ<1 and $\sqrt{1-\lambda^{2}}<\gamma <1$; (III) the regime with λ<1 and γ>1; (IV) the regime with λ>1 and 0<γ<1; (V) the regime with λ>1 and γ>1.

Now we turn to the explicit mathematical expressions for fidelity entropy $S_{f}\left(\lambda ,\gamma \right)$, fidelity temperature $T_{f}\left(\lambda ,\gamma \right)$, and fidelity internal energy $U_{f}\left(\lambda ,\gamma \right)$.

### Appendix K.1. Fidelity Entropy $S_{f}\left(\lambda ,\gamma \right)$, Fidelity Temperature $T_{f}\left(\lambda ,\gamma \right)$, and Fidelity Internal Energy $U_{f}\left(\lambda ,\gamma \right)$: An Exterior Point of View

Let us start from fidelity entropy S(λ,γ) for the three principal parts, labelled as i, iii, and v, on the two dual lines and the disordered circle, respectively.

(1) In part i (γ=1 with $0\leq \lambda <\lambda_{c}=1$), we recall that a dominant control parameter x was chosen to be x=1−λ. From Equation (9), fidelity entropy $S^{i}\left(\lambda \right)$ takes the following form:
  (A43) $$S^{i}\left(x\right)=-2\int_{x_{c}}^{x}\ln d^{i}\left(x;y\right)\;dy+S_{0}^{i}.$$
  Here, $d^{i}\left(x;y\right)$ denotes the ground-state fidelity per lattice site in part i, and $S_{0}^{i}$ is the residual fidelity entropy at the critical point $x_{c}=0$. According to our convention (cf. Section 2), we have $S_{f}^{i}\left(x\right)=S^{i}\left(x\right)$.
(2) In part iii (λ=0 with 0<γ≤1), we recall that a dominant control parameter x was chosen to be x=γ. From Equation (9), fidelity entropy $S^{\mathrm{iii}}\left(x\right)$ takes the same form as Equation (A43) for part i, with the label being changed from i to iii. According to our convention (cf. Section 2), we have $S_{f}^{\mathrm{iii}}\left(x\right)=S^{\mathrm{iii}}\left(x\right)$.
(3) In part v, we recall that a dominant control parameter x was chosen to be x=arctan(γ/λ), starting from the PT transition point (1,0) up to the U(1)-symmetric point (0,1) on the disordered circle: $\lambda^{2}+\gamma^{2}=1$. Given an exotic QPT that exists at the PT transition point on the disordered circle [59], we need to treat it separately. From Equation (9), fidelity entropy $S^{v}\left(x\right)$ takes the same form as Equation (A43) for part i, with the label being changed from i to v. According to our convention (cf. Section 2), we have $S_{f}^{v}\left(x\right)=S^{v}\left(x\right)$.

Now, we move to the five principal regimes:

(a) In regime I (0<λ<1 and $0<\gamma <\sqrt{1-\lambda^{2}}$), we recall that a dominant control parameter x was chosen to be x=γ and an auxiliary control parameter τ was chosen to be τ=λ. From Equation (28), fidelity entropy $S^{I}\left(x,\tau \right)$ takes the following form
  (A44) $$S^{I}\left(x,\tau \right)=-2\int_{x_{c}}^{x}\ln d^{I}\left(x,\tau ;y,\tau \right)\;dy+S_{0}^{I}\left(\tau \right).$$
  Here, $d^{I}\left(x,\tau ;y,\tau \right)$ denotes the ground-state fidelity per lattice site in regime I, and $S_{0}^{I}\left(\tau \right)$ is the residual fidelity entropy at a critical point $x_{c}$ for a fixed τ, with $x_{c}=0$. According to our convention (cf. Section 2), we have $S_{f}^{I}\left(x,\tau \right)=S^{I}\left(x,\tau \right)$.
(b) In regime II (0<λ<1 and $\sqrt{1-\lambda^{2}}<\gamma <1$), we recall that a dominant control parameter x was chosen to be x=1−λ and an auxiliary control parameter τ was chosen to be τ=γ. From Equation (28), fidelity entropy $S^{\mathrm{II}}\left(x,\tau \right)$ takes the same form as Equation (A44) for regime I, with the label being changed from I to II. According to our convention (cf. Section 2), we have $S_{f}^{\mathrm{II}}\left(x,\tau \right)=S^{\mathrm{II}}\left(x,\tau \right)$.
(c) In regime III ( 0<λ<1 and γ>1), we recall that a dominant control parameter x was chosen to be x=1−λ and an auxiliary control parameter τ was chosen to be τ=γ. From Equation (28), fidelity entropy $S^{\mathrm{III}}\left(x,\tau \right)$ takes the same form as Equation (A44) for regime I, with the label being changed from I to III. In this regime, the continuity requirement for $S_{f}\left(x,\tau \right)$ on the dual line, labelled as iv, implies that $S_{f}^{\mathrm{III}}\left(x,\tau \right)$ includes a contribution from scaling entropy $S_{\sigma }^{\mathrm{III}}\left(\tau \right)=S_{\sigma }^{\mathrm{iv}}\left(1/\tau \right)=-\ln\tau$, due to a multiplying factor k(1/τ)=1/τ in part iv. Hence, we have $S_{f}^{\mathrm{III}}\left(x,\tau \right)=S^{\mathrm{III}}\left(x,\tau \right)+S_{\sigma }^{\mathrm{III}}\left(\tau \right)$.
(d) In regime IV (λ>1 and 0<γ<1), we recall that a dominant control parameter x was chosen to be x=1−1/λ and an auxiliary control parameter τ was chosen to be τ=γ. Here, a re-parametrization operation in the ground-state energy density e(λ,γ): $e\left(\lambda ,\gamma \right)=m^{\mathrm{IV}}\left(x,\tau \right)e^{\mathrm{IV}}\left(x,\tau \right)$, with $m^{\mathrm{IV}}\left(x,\tau \right)=1/\left(1-x\right)$, is performed to ensure that the extent of a dominant control parameter x is finite. As discussed in Section 2, $S_{f}^{\mathrm{IV}}\left(x,\tau \right)$ includes a contribution from scaling entropy $S_{\sigma }^{\mathrm{IV}}\left(x,\tau \right)$. Here, $S_{\sigma }^{\mathrm{IV}}\left(x,\tau \right)=\ln m^{\mathrm{IV}}\left(x,\tau \right)=-\ln\left(1-x\right)$. Thus, we have $S_{f}^{\mathrm{IV}}\left(x,\tau \right)=S^{\mathrm{IV}}\left(x,\tau \right)+S_{\sigma }^{\mathrm{IV}}\left(x,\tau \right)$, where fidelity entropy $S^{\mathrm{IV}}\left(x,\tau \right)$, as follows from Equation (28), takes the same form as Equation (A44) for regime I, with the label being changed from I to IV.
(e) In regime IV (λ>1 and γ>1), we recall that a dominant control parameter x was chosen to be x=1−1/λ and an auxiliary control parameter τ was chosen to be τ=γ. Here, a re-parametrization operation in the ground-state energy density e(λ,γ): $e\left(\lambda ,\gamma \right)=m^{V}\left(x,\tau \right)e^{V}\left(x,\tau \right)$, with $m^{V}\left(x,\tau \right)=1/\left(1-x\right)$, is performed to ensure that the extent of a dominant control parameter x is finite. As discussed in Section 2, $S_{f}^{V}\left(x,\tau \right)$ includes a contribution from scaling entropy $S_{\sigma }^{V}\left(x,\tau \right)$. Here, $S_{\sigma }^{V}\left(x,\tau \right)=\ln m^{V}\left(x,\tau \right)=-\ln\left(1-x\right)$. Thus, we have $S_{f}^{V}\left(x,\tau \right)=S^{V}\left(x,\tau \right)+S_{\sigma }^{V}\left(x,\tau \right)$, where fidelity entropy $S^{V}\left(x,\tau \right)$, as follows from Equation (28), takes the same form as Equation (A44) for regime I, with the label being changed from I to V.

We start our discussion from the three principal parts, labelled as i, iii, and v:

(1) In part i (γ=1 with $0\leq \lambda <\lambda_{c}=1$), for the chosen dominant control parameter x: x=1−λ, the ground-state energy density $e^{i}\left(x\right)$ increases with x. Then, from Equation (10), fidelity internal energy $U^{i}\left(x\right)$ takes the following form
  (A45) $$U^{i}\left(x\right)=-\ln\frac{e^{i}\left(x\right)}{e^{i}\left(0\right)}V^{i}\left(x\right)+U_{0}^{i}.$$
  Here, $U_{0}^{i}$ is an additive constant, and $V^{i}\left(x\right)>0$ satisfies the singular first-order differential equation
  (A46) $$\partial V^{i}\left(x\right)/\partial x=\alpha^{i}\left(x\right)\;V^{i}\left(x\right),$$
  with
  (A47) $$\alpha^{i}\left(x\right)=\frac{\partial \ln\left(e^{i}\left(x\right)/e^{i}\left(0\right)\right)/\partial x}{\partial S^{i}\left(x\right)/\partial x-\ln\left(e^{i}\left(x\right)/e^{i}\left(0\right)\right)}.$$
  Accordingly, fidelity temperature $T^{i}\left(x\right)$ follows from
  (A48) $$T^{i}\left(x\right)=-\frac{\partial V^{i}\left(x\right)}{\partial x}.$$
(2) In part iii (λ=0 with 0<γ≤1), for the chosen dominant control parameter x: x=γ, the ground-state energy density $e^{\mathrm{iii}}\left(x\right)$ decreases with x. Then, fidelity internal energy $U^{\mathrm{iii}}\left(x\right)$ takes the following form
  (A49) $$U^{\mathrm{iii}}\left(x\right)=\ln\frac{e^{\mathrm{iii}}\left(x\right)}{e^{\mathrm{iii}}\left(0\right)}V^{\mathrm{iii}}\left(x\right)+U_{0}^{\mathrm{iii}}.$$
  Here, $U_{0}^{\mathrm{iii}}$ is an additive constant, and $V^{\mathrm{iii}}\left(x\right)>0$ satisfies the singular first-order differential equation
  (A50) $$\partial V^{\mathrm{iii}}\left(x\right)/\partial x=\alpha^{\mathrm{iii}}\left(x\right)\;V^{\mathrm{iii}}\left(x\right),$$
  with
  (A51) $$\alpha^{\mathrm{iii}}\left(x\right)=-\frac{\partial \ln\left(e^{\mathrm{iii}}\left(x\right)/e^{\mathrm{iii}}\left(0\right)\right)/\partial x}{\partial S^{\mathrm{iii}}\left(x\right)/\partial x+\ln\left(e^{\mathrm{iii}}\left(x\right)/e^{\mathrm{iii}}\left(0\right)\right)}.$$
  Accordingly, fidelity temperature $T^{\mathrm{iii}}\left(x\right)$ follows from
  (A52) $$T^{\mathrm{iii}}\left(x\right)=-\frac{\partial V^{\mathrm{iii}}\left(x\right)}{\partial x}.$$
(3) In part v, on the disordered circle, the ground-state energy density $e^{v}\left(x\right)$ is a constant for the chosen dominant control parameter x: x=θ=arctan(γ/λ). Then, fidelity internal energy $U^{v}\left(x\right)$ is a constant, and fidelity temperature $T^{v}\left(x\right)$ is zero:
  (A53) $$U^{v}\left(x\right)=U^{\mathrm{iii}}\left(1\right),\;T^{v}\left(x\right)=0.$$
  As discussed in Section 2, we have $U_{f}^{v}\left(x\right)=U^{v}\left(x\right)$ and $T_{f}^{v}\left(x\right)=T^{v}\left(x\right)$.

Now we move to the five principal regimes, labelled as I, II, III, IV, and V:

(a) In regime I (0<λ<1 and $0<\gamma <\sqrt{1-\lambda^{2}}$), for the chosen dominant control parameter x: x=γ, the ground-state energy density $e^{I}\left(x,\tau \right)$ monotonically decreases with x. Then, from Equation (30), fidelity internal energy $U^{I}\left(x,\tau \right)$ takes the following form
  (A54) $$U^{I}\left(x,\tau \right)=\ln\frac{e^{I}\left(x,\tau \right)}{e^{I}\left(0,\tau \right)}V^{I}\left(x,\tau \right)+U_{0}^{I}\left(\tau \right).$$
  Here, $U_{0}^{I}\left(\tau \right)$ is a function of τ, and $V^{I}\left(x,\tau \right)>0$ satisfies the singular first-order differential equation
  (A55) $$\frac{\partial V^{I}\left(x,\tau \right)}{\partial x}=\alpha^{I}\left(x,\tau \right)\;V^{I}\left(x,\tau \right),$$
  with
  (A56) $$\alpha^{I}\left(x,\tau \right)=-\frac{\partial \ln\left(e^{I}\left(x,\tau \right)/e^{I}\left(0,\tau \right)\right)/\partial x}{\partial S^{I}\left(x,\tau \right)/\partial x+\ln\left(e^{I}\left(x,\tau \right)/e^{I}\left(0,\tau \right)\right)}.$$
  Accordingly, fidelity temperature $T^{I}\left(x,\tau \right)$ in this regime is given by the following
  (A57) $$T^{I}\left(x,\tau \right)=-\frac{\partial V^{I}\left(x,\tau \right)}{\partial x}.$$
(b) In regime II (0<λ<1 and $\sqrt{1-\lambda^{2}}<\gamma <1$ ), for the chosen dominant control parameter x: x=1−λ, the ground-state energy density $e^{\mathrm{II}}\left(x,\tau \right)$ monotonically increases with x. Then, from Equation (30), fidelity internal energy $U^{\mathrm{II}}\left(x,\tau \right)$ takes the following form
  (A58) $$U^{\mathrm{II}}\left(x,\tau \right)=-\ln\frac{e^{\mathrm{II}}\left(x,\tau \right)}{e^{\mathrm{II}}\left(0,\tau \right)}V^{\mathrm{II}}\left(x,\tau \right)+U_{0}^{\mathrm{II}}\left(\tau \right).$$
  Here, $U_{0}^{\mathrm{II}}\left(\tau \right)$ is a function of τ, and $V^{\mathrm{II}}\left(x,\tau \right)>0$ satisfies the singular first-order differential equation
  (A59) $$\frac{\partial V^{\mathrm{II}}\left(x,\tau \right)}{\partial x}=\alpha^{\mathrm{II}}\left(x,\tau \right)\;V^{\mathrm{II}}\left(x,\tau \right),$$
  with
  (A60) $$\alpha^{\mathrm{II}}\left(x,\tau \right)=\frac{\partial \ln\left(e^{\mathrm{II}}\left(x,\tau \right)/e^{\mathrm{II}}\left(0,\tau \right)\right)/\partial x}{\partial S^{\mathrm{II}}\left(x,\tau \right)/\partial x-\ln e^{\mathrm{II}}\left(x,\tau \right)/e\left(0,\tau \right)}.$$
  Accordingly, fidelity temperature $T^{\mathrm{II}}\left(x,\tau \right)$ in this regime is given by the following
  (A61) $$T^{\mathrm{II}}\left(x,\tau \right)=-\frac{\partial V^{\mathrm{II}}\left(x,\tau \right)}{\partial x}.$$
(c) In regimes ω= III, IV, and V, fidelity internal energy $U^{\omega }\left(x,\tau \right)$, fidelity temperature $T^{\omega }\left(x,\tau \right)$, and $V^{\omega }\left(x,\tau \right)$, together with the singular first-order differential equations with $\alpha^{\omega }\left(x,\tau \right)$ take the same form as Equations (A58)–(A61) for regime II, with the label being changed from II to III, IV, and V, respectively.

In order to solve a singular first-order differential equation in principal part i and principal part ii and in each principal regime, we perform a scaling analysis of $\alpha^{q}\left(x\right)$ (q= i and ii) and $\alpha^{\omega }\left(x,\tau \right)$ (ω = I, II, III, IV, and IV) in the vicinity of a critical point $x_{c}=0$, which falls into two universality classes: (A) the Gaussian universality class for part iii on the dual line and regime I, and (B) the Ising universality class for part i on the dual line and regime II, regime III, regime IV, and regime V:

(A) When a Gaussian critical point $x_{c}=0$ is approached, fidelity entropy $S^{\mathrm{iii}}\left(x\right)$ in part iii and $S^{I}\left(x,\tau \right)$ in regime I scale as $S^{\mathrm{iii}}\left(x\right)∼x^{2}$ and $S^{I}\left(x,\tau \right)∼x^{2}$, respectively. This indicates that the critical exponent is ν=1 (cf. Appendix H for details). Combined with a scaling analysis of the ground-state energy density $e^{\mathrm{iii}}\left(x\right)$ and $e^{I}\left(x,\tau \right)$ near a Gaussian critical point $x_{c}=0$: $\ln\left(e^{\mathrm{iii}}\left(x\right)/e^{\mathrm{iii}}\left(0\right)\right)∼\ln x\;x^{2}$ and $\ln\left(e^{I}\left(x,\tau \right)/e^{I}\left(0,\tau \right)\right)∼\ln x\;x^{2}$ (cf. Appendix H for details), we have the following:
  (A62) $$\alpha^{\mathrm{iii}}\left(x\right)∼\ln\;x,$$
  and
  (A63) $$\alpha^{I}\left(x,\tau \right)∼\ln\;x.$$
  The scaling behaviors for part iii and regime I are the same, as anticipated from the fact that they both belong to the Gaussian universality class. Our numerical simulations confirm this scaling analysis.
(B) When an Ising critical point is approached, fidelity entropy $S^{i}\left(x\right)$ in part i, and fidelity entropy $S^{\mathrm{II}}\left(x,\tau \right)$, $S^{\mathrm{III}}\left(x,\tau \right)$, $S^{\mathrm{IV}}\left(x,\tau \right)$, and $S^{V}\left(x,\tau \right)$ in regime II, regime III, regime IV, and regime V scale as $S^{i}\left(x\right)∼x^{2}$ and $S^{\omega }\left(x,\tau \right)∼x^{2}$ (ω=II,III,IV, and V). This indicates that the critical exponent is ν=1 (cf. Appendix H for details). Taking into account the fact that the first-order derivative of $\ln\left(e^{i}\left(x\right)/e^{i}\left(0\right)\right)$ and $\ln\left(e^{\omega }\left(x,\tau \right)/e^{\omega }\left(0,\tau \right)\right)$ with respect to x at a critical point $x_{c}=0$ is nonzero, we have the following:
  (A64) $$\alpha^{i}\left(x\right)∼\frac{1}{x},$$
  and
  (A65) $$\alpha^{\omega }\left(x,\tau \right)∼\frac{1}{x}.$$
  The scaling behaviors for part i and regimes ω, with ω=II,III,IV, and V, are the same, as anticipated from the fact that they belong to the Ising universality class. Our numerical simulations confirm this scaling analysis.

Our analysis enables us to solve a singular first-order differential equation, as shown below.

(1) In part i (γ=1 with $0\leq \lambda <\lambda_{c}=1$), since the integration of $\alpha^{i}\left(x\right)$ with respect to x is finite, the singular first-order differential equation, Equation (A46), may be solved as follows:
  (A66) $$V^{i}\left(x\right)=V_{0}^{i}V_{1}^{i}\left(x\right),$$
  where $V_{0}^{i}$ is a constant to be determined, and $V_{1}^{i}\left(x\right)$ takes the following form
  (A67) $$V_{1}^{i}\left(x\right)=\exp\left(\int_{0}^{x}\alpha^{i}\left(y\right)dy\right).$$
(2) In part iii (λ=0 with 0<γ≤1), the solution $V^{\mathrm{iii}}\left(x\right)$ to the singular first-order differential equation equation, Equation (A50), takes the same form as Equations (A66) and (A67) in part i, with the label being changed from i to iii.
(3) In part v, $V^{v}\left(x\right)$ simply vanishes, given that $U_{f}^{v}\left(x\right)$ is a constant on the disordered circle: $\lambda^{2}+\gamma^{2}=1$.

Similarly, we may determine $V^{\omega }\left(x,\tau \right)$ in the five principal regimes, with ω=I,II,III,IV and V:

(a) In regime I (0<λ<1 and $0<\gamma <\sqrt{1-\lambda^{2}}$), since the integration of $\alpha^{I}\left(x,\tau \right)$ with respect to x for a fixed τ is finite, the singular first-order differential equation, Equation (A55), may be solved as follows:
  (A68) $$V^{I}\left(x,\tau \right)=V_{0}^{I}\left(\tau \right)V_{1}^{I}\left(x,\tau \right),$$
  where $V_{0}^{I}\left(\tau \right)$ is a function of τ, and $V_{1}^{I}\left(x,\tau \right)$ is defined as follows
  (A69) $$V_{1}^{I}\left(x,\tau \right)=\exp\left(\int_{0}^{x}\alpha^{I}\left(y,\tau \right)\mathrm{dy}\right).$$
(b) In regime II, regime III, regime IV, and regime V, the solutions $V^{\mathrm{II}}\left(x,\tau \right)$, $V^{\mathrm{III}}\left(x,\tau \right)$, $V^{\mathrm{IV}}\left(x,\tau \right)$, and $V^{V}\left(x,\tau \right)$ to the singular first-order differential equations take the same form as Equations (A68) and (A69) for regime I, with the label being changed from I to II, III, IV, and V, respectively.

Now we turn to the residual fidelity entropy on the two lines of critical points. We remark that fidelity entropy, generically, is *relative*, in the sense that it is *only* determined up to a constant. Here, as a convention, we choose fidelity entropy to be zero at the critical point (1, 0). That is, we choose $S_{0}^{\mathrm{iii}}=0$. Then, $S_{f}^{\mathrm{iii}}\left(x\right)$ follows from its definition, which takes the same form as (A43), with the label being changed from i to iii. Furthermore, the residual fidelity entropy $S_{0}^{i}$ for part i and the residual fidelity entropy $S_{0}^{v}$ for part v are determined from the continuity requirement for fidelity entropy at the characteristic point (λ=0 and γ=1): $S_{f}^{i}\left(1\right)=S_{f}^{\mathrm{iii}}\left(1\right)$ and $S_{f}^{v}\left(\pi /2\right)=S_{f}^{\mathrm{iii}}\left(1\right)$. Hence, fidelity entropy $S^{i}\left(x\right)$ on the dual line, labelled as i, and fidelity entropy $S^{v}\left(x\right)$ on the disordered circle, labelled as v, follow from (A43) and its counterpart in part v, with the label being changed from i to v, respectively.

Therefore, fidelity entropy in part ii and part iii follow from their duality transformation to part i and to part iv, respectively.

(1) In part ii (γ=1 with $\lambda >\lambda_{c}=1$), which is dual to principal part i, we have the following: $H^{\mathrm{ii}}\left(\lambda \right)=k_{\mathrm{ii}\;i}\left(x\right)U_{1}H^{i}\left(x\right)U_{1}^{†}$, with x=1/λ and $k_{\mathrm{ii}\;i}\left(x\right)=1/x$. This results in rescaling in the ground-state energy density $e^{\mathrm{ii}}\left(\lambda \right)$: $e^{\mathrm{ii}}\left(\lambda \right)=k_{\mathrm{ii}\;i}\left(x\right)e^{i}\left(x\right)$. As discussed in Section 2, an additional contribution to fidelity entropy $S_{f}^{\mathrm{ii}}\left(\lambda \right)$ is scaling entropy $S_{\sigma }^{\mathrm{ii}}\left(\lambda \right)\equiv S_{\sigma }^{\mathrm{ii}\;i}\left(\lambda \right)$, with $S_{\sigma }^{\mathrm{ii}\;i}\left(\lambda \right)=\ln k_{\mathrm{ii}\;i}\left(\lambda \right)$, arising from a multiplying factor $k_{\mathrm{ii}\;i}\left(\lambda \right)$, in addition to the contribution from the ground-state fidelity per lattice site $S^{i}\left(\lambda \right)$. Here, $k_{\mathrm{ii}\;i}\left(\lambda \right)\equiv k_{\mathrm{ii}\;i}\left(x\right)$, so $k_{\mathrm{ii}\;i}\left(\lambda \right)=\lambda$. That is, we have $S_{f}^{\mathrm{ii}}\left(\lambda \right)=S_{f}^{i}\left(\lambda \right)+S_{\sigma }^{\mathrm{ii}}\left(\lambda \right)$.

(2) In part iv (λ=0 with γ≥1), which is dual to principal part iii, we have the following: $H^{\mathrm{iv}}\left(\gamma \right)=k_{\mathrm{iv}\;\mathrm{iii}}\left(x\right)U_{2}H^{\mathrm{iii}}\left(x\right)U_{2}^{†}$, with x=1/γ and $k_{\mathrm{iv}\;\mathrm{iii}}\left(x\right)=1/x$. This results in rescaling in the ground-state energy density $e^{\mathrm{iv}}\left(\gamma \right)$: $e^{\mathrm{iv}}\left(\gamma \right)=k_{\mathrm{iv}\;\mathrm{iii}}\left(x\right)e^{\mathrm{iii}}\left(x\right)$. As discussed in Section 2, an additional contribution to fidelity entropy $S_{f}^{\mathrm{iv}}\left(\gamma \right)$ is scaling entropy $S_{\sigma }^{\mathrm{iv}}\left(\gamma \right)\equiv S_{\sigma }^{\mathrm{iv}\;\mathrm{iii}}\left(\gamma \right)$, with $S_{\sigma }^{\mathrm{iv}\;\mathrm{iii}}\left(\gamma \right)=-\ln k_{\mathrm{iv}\;\mathrm{iii}}\left(\gamma \right)$, arising from a multiplying factor $k_{\mathrm{iv}\;\mathrm{iii}}\left(\gamma \right)$, in addition to the contribution from the ground-state fidelity per lattice site $S^{\mathrm{iii}}\left(\gamma \right)$. Here, $k_{\mathrm{iv}\;\mathrm{iii}}\left(\gamma \right)\equiv k_{\mathrm{iv}\;\mathrm{iii}}\left(x\right)$; thus, $k_{\mathrm{iv}\;\mathrm{iii}}\left(\gamma \right)=\gamma$. That is, we have $S_{f}^{\mathrm{iv}}\left(\gamma \right)=S_{f}^{\mathrm{iv}}\left(\gamma \right)+S_{\sigma }^{\mathrm{iv}}\left(\gamma \right)$.

We are free to choose $V_{0}$ on one of the characteristic lines, since fidelity internal energy is only determined up to a constant factor. Here, we set $V_{0}^{\mathrm{iii}}=1$ for the dual line (λ=0 with 0<γ≤1), labelled as iii. Then, fidelity internal energy $U^{\mathrm{iii}}\left(x\right)$ on the $Z_{2}$-symmetric and dual line (λ=0 with 0<γ≤1) is determined from (A49). A shift from $T^{\mathrm{iii}}\left(x\right)$ to $T^{\mathrm{iii}}\left(x\right)-T_{0}^{\mathrm{iii}}$ induces a shift in fidelity internal energy in part iii: $U^{\mathrm{iii}}\left(x\right)-T_{0}^{\mathrm{iii}}S^{\mathrm{iii}}\left(x\right)$, with $T_{0}^{\mathrm{iii}}=T^{\mathrm{iii}}\left(1\right)$. We refer to $T^{\mathrm{iii}}\left(x\right)-T_{0}^{\mathrm{iii}}$ and $U^{\mathrm{iii}}\left(x\right)-T_{0}^{\mathrm{iii}}S^{\mathrm{iii}}\left(x\right)$ as $T_{f}^{\mathrm{iii}}\left(x\right)$ and $U_{f}^{\mathrm{iii}}\left(x\right)$, respectively. That is, $T_{f}^{\mathrm{iii}}\left(x\right)\equiv T^{\mathrm{iii}}\left(x\right)-T_{0}^{\mathrm{iii}}$ and $U_{f}^{\mathrm{iii}}\left(x\right)\equiv U^{\mathrm{iii}}\left(x\right)-T_{0}^{\mathrm{iii}}S^{\mathrm{iii}}\left(x\right)$. Then, as discussed in Section 2, fidelity internal energy $U_{f}^{\mathrm{iv}}\left(\gamma \right)$ and $T_{f}^{\mathrm{iv}}\left(\gamma \right)$ in part iv (λ=0 with γ≥1) follow from duality. That is, fidelity internal energy $U^{\mathrm{iv}}\left(\gamma \right)$ and fidelity temperature $T^{\mathrm{iv}}\left(\gamma \right)$ take the following form: $U^{\mathrm{iv}}\left(\gamma \right)=U^{\mathrm{iii}}\left(1/\gamma \right)$ and $T^{\mathrm{iv}}\left(\gamma \right)=T^{\mathrm{iii}}\left(1/\gamma \right)$. In addition, as shown in (A53), fidelity internal energy $U^{v}\left(x\right)$ on the disordered circle, labelled as v, is a constant, which is determined to be $U_{m}$, with $U_{m}=U_{f}^{\mathrm{iii}}\left(1\right)$, from the continuity requirement for fidelity internal energy.

On the dual line (γ=1 with 0<λ<1), labelled as i, $T^{i}\left(x\right)$ is shifted to $T^{i}\left(x\right)-T_{0}^{i}$, with $T_{0}^{I}=T^{i}\left(1\right)$. Then, $U^{i}\left(x\right)$ is shifted to $U^{i}\left(x\right)-T_{0}^{i}S^{i}\left(x\right)$. In addition, fidelity internal energy at a critical point with $x=x_{c}=0$ or, equivalently, λ=1 and γ=1 must be zero, and fidelity internal energy satisfies the continuity requirement at the stable fixed point with x=1 or, equivalently, λ=0 and γ=1: $U_{0}^{i}=T_{0}^{i}S_{0}^{i}$ and $U^{i}\left(1\right)-T_{0}^{i}S^{i}\left(1\right)=U_{f}^{\mathrm{iii}}\left(1\right)$. Therefore, $V_{0}^{i}$ is determined as follows

(A70) $$V_{0}^{i}=\frac{U_{f}^{\mathrm{iii}}\left(1\right)}{-\ln\left(e^{i}\left(1\right)/e^{i}\left(0\right)\right)V_{1}^{i}\left(1\right)+\alpha^{i}\left(1\right)V_{1}^{i}\left(1\right)\left(S^{i}\left(1\right)-S_{0}^{i}\right)}.$$

Once $V_{0}^{i}$ and $U_{0}^{i}$ are determined, fidelity temperature $T^{i}\left(x\right)$ and fidelity internal energy $U^{i}\left(x\right)$ follow from (A45) and (A48). We refer to $T^{i}\left(x\right)-T_{0}^{i}$ and $U^{i}\left(x\right)-T_{0}^{i}S^{i}\left(x\right)$ as $T_{f}^{i}\left(x\right)$ and $U_{f}^{i}\left(x\right)$, respectively. That is, $T_{f}^{i}\left(x\right)\equiv T^{i}\left(x\right)-T_{0}^{i}$ and $U_{f}^{i}\left(x\right)\equiv U^{i}\left(x\right)-T_{0}^{i}S^{i}\left(x\right)$. Then, as discussed in Section 2, fidelity temperature $T_{f}^{\mathrm{ii}}\left(x\right)$ and fidelity internal energy $U_{f}^{\mathrm{ii}}\left(x\right)$ in part ii ($\lambda >\lambda_{c}=1$ and γ=1) follow from duality. That is, fidelity temperature $T^{\mathrm{ii}}\left(\lambda \right)$ and fidelity internal energy $U^{\mathrm{ii}}\left(\lambda \right)$ take the following form: $T^{\mathrm{ii}}\left(\lambda \right)=T^{i}\left(1/\lambda \right)$ and $U^{\mathrm{ii}}\left(\lambda \right)$ $=U^{i}\left(1/\lambda \right)$, respectively.

Now we have to ensure that fidelity mechanical-state functions are *continuous* at the boundaries between different regimes for each phase. With this in mind, we are able to determine the residual fidelity entropy $S_{0}^{I}\left(\tau \right)$ on the line of the Gaussian critical points (γ=0 with 0<λ<1) and $S_{0}^{\mathrm{II}}\left(\tau \right)$ on the line of the Ising critical points (λ=1 with 0<γ<1) from the continuity requirements on the disordered circle $\lambda^{2}+\gamma^{2}=1$, labelled as v: $S_{f}^{I}\left(\sqrt{1-\tau^{2}},\tau \right)=S_{f}^{v}\left(\arctan\left(\sqrt{1-\tau^{2}}/\tau \right)\right)$ and $S_{f}^{\mathrm{II}}\left(\sqrt{1-\tau^{2}},\tau \right)=S_{f}^{v}\left(\arctan\left(\tau /\sqrt{1-\tau^{2}}\right)\right)$. Similarly, the residual fidelity entropy $S_{0}^{\mathrm{III}}\left(\tau \right)$ on the line of the Ising critical points (λ=1 with γ>1) is determined from the continuity requirement on the dual line (λ=0 with γ>1), labelled as iv: $S_{f}^{\mathrm{III}}\left(1,\tau \right)=S_{f}^{\mathrm{iv}}\left(1/\tau \right)$. In addition, the continuity requirement for fidelity entropy on the line of the Ising critical points (λ=1 with γ>0), implies that $S_{0}^{\mathrm{IV}}\left(\tau \right)=S_{0}^{\mathrm{II}}\left(\tau \right)$ and $S_{0}^{V}\left(\tau \right)=S_{0}^{\mathrm{III}}\left(\tau \right)$.

The remaining task is to ensure the continuity requirements for fidelity temperature and fidelity internal energy. To this end, we need to determine $T_{0}^{\omega }\equiv T_{m}^{\omega }-T_{t}^{\omega }$ for the five principal regimes, with ω=I, II, III, IV, and V. Here, $T_{m}^{\omega }$ represents fidelity temperature, evaluated from a dominant control parameter x in one principal regime ω, at a chosen point on a principal part, which is a boundary in the principal regime ω, whereas $T_{t}^{\omega }$ represents fidelity temperature at the same point, but it is evaluated from a dominant control parameter x in the principal part itself. Specifically, in regime I, for a fixed τ=λ, we have $T_{m}^{I}\left(\tau \right)=T^{I}\left(\sqrt{1-\tau^{2}},\tau \right)$ and $T_{t}^{I}\left(\tau \right)=0$ on the disordered circle, labelled as v. In regime II, for a fixed τ=γ, we have $T_{m}^{\mathrm{II}}\left(\tau \right)=T^{\mathrm{II}}\left(\sqrt{1-\tau^{2}},\tau \right)$ and $T_{t}^{\mathrm{II}}\left(\tau \right)=0$ on the disordered circle, labelled as v. In regime III, for a fixed τ=γ, we have $T_{m}^{\mathrm{III}}\left(\tau \right)=T^{\mathrm{III}}\left(1,\tau \right)$ and $T_{t}^{\mathrm{III}}\left(\tau \right)=T_{f}^{\mathrm{iv}}\left(1/\tau \right)$ in the dual image part iv, located on the dual line (λ=0 with γ≥1). In regime IV, for a fixed τ=γ, we have $T_{m}^{\mathrm{IV}}\left(\tau \right)=T^{\mathrm{IV}}\left(1,\tau \right)$ and $T_{t}^{\mathrm{IV}}\left(\tau \right)=0$, when x=1; i.e., when λ is infinite in value. In regime V, for a fixed τ=γ, we have $T_{m}^{V}\left(\tau \right)=T^{V}\left(1,\tau \right)$ and $T_{t}^{V}\left(\tau \right)=0$, when x=1; i.e., when λ is infinite in value.

(a) In regime I (λ<1 and $0<\gamma <\sqrt{1-\lambda^{2}}$), for a fixed τ, in order to ensure the continuity requirement for fidelity temperature on the disordered circle, labelled as v, $T^{I}\left(x,\tau \right)$ is shifted to $T^{I}\left(x,\tau \right)-T_{0}^{I}\left(\tau \right)$, with $T_{0}^{I}\left(\tau \right)=T^{I}\left(\sqrt{1-\tau^{2}},\tau \right)$. Then, $U^{I}\left(x,\tau \right)$ is shifted to $U^{I}\left(x,\tau \right)-T_{0}^{I}\left(\tau \right)S^{I}\left(x,\tau \right)$, with $S^{I}\left(x,\tau \right)$ left intact. In addition, fidelity internal energy on the line of the Gaussian critical points (γ=0 with 0<λ<1) with $x=x_{c}=0$, for a fixed τ, must be zero, and fidelity internal energy satisfies the continuity requirement on the disordered circle, labelled as v, as discussed in Section 2: $U_{0}^{I}\left(\tau \right)=T_{0}^{I}\left(\tau \right)S_{0}^{I}\left(\tau \right)$ and $U^{I}\left(\sqrt{1-\tau^{2}},\tau \right)-T_{0}^{I}\left(\tau \right)S^{I}\left(\sqrt{1-\tau^{2}},\tau \right)=U_{m}$. Therefore, $V_{0}^{I}\left(\tau \right)$ is determined as follows

(A71) $$V_{0}^{I}\left(\tau \right)=\frac{U_{m}}{\ln\left(e^{I}\left(\sqrt{1-\tau^{2}},\tau \right)/e^{I}\left(0,\tau \right)\right)V_{1}^{I}\left(\sqrt{1-\tau^{2}},\tau \right)+\alpha^{I}\left(\sqrt{1-\tau^{2}},\tau \right)V_{1}^{I}\left(\sqrt{1-\tau^{2}},\tau \right)\left(S^{I}\left(\sqrt{1-\tau^{2}},\tau \right)-S_{0}^{I}\left(\tau \right)\right)}.$$

Once $V_{0}^{I}\left(\tau \right)$ and $U_{0}^{I}\left(\tau \right)$ are determined, fidelity temperature $T^{I}\left(x,\tau \right)$ and fidelity internal energy $U^{I}\left(x,\tau \right)$ follow from (A54) and (A57), respectively. Following from our discussions in Section 2, fidelity entropy $S_{f}^{I}\left(x,\tau \right)$, fidelity temperature $T_{f}^{I}\left(x,\tau \right)$, and fidelity internal energy $U_{f}^{I}\left(x,\tau \right)$ take the following form: $S_{f}^{I}\left(x,\tau \right)=S^{I}\left(x,\tau \right)$, $T_{f}^{I}\left(x,\tau \right)=T^{I}\left(x,\tau \right)-T_{0}^{I}\left(\tau \right)$ and $U_{f}^{I}\left(x,\tau \right)=U^{I}\left(x,\tau \right)-T_{0}^{I}\left(\tau \right)S^{I}\left(x,\tau \right)$, respectively.

(b) In regime II ($\sqrt{1-\lambda^{2}}<\gamma <1$ and 0<λ<1), for a fixed τ, in order to ensure the continuity requirement for fidelity temperature on the disordered circle, labelled as v, $T^{\mathrm{II}}\left(x,\tau \right)$ is shifted to $T^{\mathrm{II}}\left(x,\tau \right)-T_{0}^{\mathrm{II}}\left(\tau \right)$, with $T_{0}^{\mathrm{II}}\left(\tau \right)=T^{\mathrm{II}}\left(\sqrt{1-\tau^{2}},\tau \right)$. Then, $U^{\mathrm{II}}\left(x,\tau \right)$ is shifted to $U^{\mathrm{II}}\left(x,\tau \right)-T_{0}^{\mathrm{II}}\left(\tau \right)S^{\mathrm{II}}\left(x,\tau \right)$, with $S^{\mathrm{II}}\left(x,\tau \right)$ left intact. In addition, fidelity internal energy on the line of the Ising critical points (λ=1 with 0<γ≤1) with $x=x_{c}=0$, for a fixed τ, must be zero, and fidelity internal energy satisfies the continuity requirement on the disordered circle, labelled as v, as discussed in Section 2: $U_{0}^{\mathrm{II}}\left(\tau \right)=T_{0}^{\mathrm{II}}\left(\tau \right)S_{0}^{\mathrm{II}}\left(\tau \right)$ and $U^{\mathrm{II}}\left(\sqrt{1-\tau^{2}},\tau \right)-T_{0}^{\mathrm{II}}\left(\tau \right)S^{\mathrm{II}}\left(\sqrt{1-\tau^{2}},\tau \right)=U_{m}$. Therefore, $V_{0}^{\mathrm{II}}\left(\tau \right)$ is determined as follows

(A72) $$V_{0}^{\mathrm{II}}\left(\tau \right)\;=\;\frac{U_{m}}{\;-\;\ln\left(e^{\mathrm{II}}\left(\sqrt{1\;-\;\tau^{2}},\tau \right)/e^{\mathrm{II}}\left(0,\tau \right)\right)V_{1}^{\mathrm{II}}\left(\sqrt{1\;-\;\tau^{2}},\tau \right)\;+\;\alpha^{\mathrm{II}}\left(\sqrt{1\;-\;\tau^{2}},\tau \right)V_{1}^{\mathrm{II}}\left(\sqrt{1\;-\;\tau^{2}},\tau \right)\left(S^{\mathrm{II}}\left(\sqrt{1\;-\;\tau^{2}},\tau \right)\;-\;S_{0}^{\mathrm{II}}\left(\tau \right)\right)}.$$

Once $V_{0}^{\mathrm{II}}\left(\tau \right)$ and $U_{0}^{\mathrm{II}}\left(\tau \right)$ are determined, fidelity temperature $T^{\mathrm{II}}\left(x,\tau \right)$ and fidelity internal energy $U^{\mathrm{II}}\left(x,\tau \right)$ follow from (A58) and (A61), respectively. Following from our discussions in Section 2, fidelity entropy $S_{f}^{\mathrm{II}}\left(x,\tau \right)$, fidelity temperature $T_{f}^{\mathrm{II}}\left(x,\tau \right)$, and fidelity internal energy $U_{f}^{\mathrm{II}}\left(x,\tau \right)$ take the following form: $S_{f}^{\mathrm{II}}\left(x,\tau \right)=S^{\mathrm{II}}\left(x,\tau \right)$, $T_{f}^{\mathrm{II}}\left(x,\tau \right)=T^{\mathrm{II}}\left(x,\tau \right)-T_{0}^{\mathrm{II}}\left(\tau \right)$, and $U_{f}^{\mathrm{II}}\left(x,\tau \right)=U^{\mathrm{II}}\left(x,\tau \right)-T_{0}^{\mathrm{II}}\left(\tau \right)S^{\mathrm{II}}\left(x,\tau \right)$, respectively.

(c) In regime III ( 0<λ<1 and γ>1), for a fixed τ, in order to ensure the continuity requirement for fidelity temperature on the dual line (λ=0 with γ≥1), labelled as iv, $T^{\mathrm{III}}\left(x,\tau \right)$ is shifted to $T^{\mathrm{III}}\left(x,\tau \right)-T_{0}^{\mathrm{III}}\left(\tau \right)$, with $T_{0}^{\mathrm{III}}\left(\tau \right)=T^{\mathrm{III}}\left(1,\tau \right)-T_{f}^{\mathrm{iv}}\left(1/\tau \right)$. Then, $U^{\mathrm{III}}\left(x,\tau \right)$ is shifted to $U^{\mathrm{III}}\left(x,\tau \right)-T_{0}^{\mathrm{III}}\left(\tau \right)S^{\mathrm{III}}\left(x,\tau \right)$, with $S^{\mathrm{III}}\left(x,\tau \right)$ left intact. In addition, fidelity internal energy on the line of the Ising critical points (λ=1 with 0<γ<1), with $x=x_{c}=0$, for a fixed τ, must be zero, and fidelity internal energy satisfies the continuity requirement at the $Z_{2}$-symmetric and dual line (λ=0 with γ≥1), labelled as iv, as discussed in Section 2: $U_{0}^{\mathrm{III}}\left(\tau \right)=T_{0}^{\mathrm{III}}\left(\tau \right)S_{0}^{\mathrm{III}}\left(\tau \right)$ and $U^{\mathrm{III}}\left(1,\tau \right)-T_{0}^{\mathrm{III}}\left(\tau \right)S^{\mathrm{III}}\left(1,\tau \right)=U_{f}^{\mathrm{iv}}\left(1/\tau \right)$. Therefore, $V_{0}^{\mathrm{III}}\left(\tau \right)$ is determined as follows

(A73) $$V_{0}^{\mathrm{III}}\left(\tau \right)=\frac{U_{f}^{\mathrm{iv}}\left(1/\tau \right)-T_{f}^{\mathrm{iv}}\left(1/\tau \right)\left(S^{\mathrm{III}}\left(1,\tau \right)-S_{0}^{\mathrm{III}}\left(\tau \right)\right)}{-\ln\left(e^{\mathrm{III}}\left(1,\tau \right)/e^{\mathrm{III}}\left(0,\tau \right)\right)V_{1}^{\mathrm{III}}\left(1,\tau \right)+\alpha^{\mathrm{III}}\left(1,\tau \right)V_{1}^{\mathrm{III}}\left(1,\tau \right)\left(S^{\mathrm{III}}\left(1,\tau \right)-S_{0}^{\mathrm{III}}\left(\tau \right)\right)}.$$

Once $V_{0}^{\mathrm{III}}\left(\tau \right)$ and $U_{0}^{\mathrm{III}}\left(\tau \right)$ are determined, fidelity temperature $T^{\mathrm{III}}\left(x,\tau \right)$ and fidelity internal energy $U^{\mathrm{III}}\left(x,\tau \right)$ follow from their counterparts of (A58) and (A61), with the label being changed from II to III, respectively. Following from our discussions in Section 2, fidelity entropy $S_{f}^{\mathrm{III}}\left(x,\tau \right)$, fidelity temperature $T_{f}^{\mathrm{III}}\left(x,\tau \right)$, and fidelity internal energy $U_{f}^{\mathrm{III}}\left(x,\tau \right)$ take the following form: $S_{f}^{\mathrm{III}}\left(x,\tau \right)=S^{\mathrm{III}}\left(x,\tau \right)$, $T_{f}^{\mathrm{III}}\left(x,\tau \right)=T^{\mathrm{III}}\left(x,\tau \right)-T_{0}^{\mathrm{III}}\left(\tau \right)$ and $U_{f}^{\mathrm{III}}\left(x,\tau \right)=U^{\mathrm{III}}\left(x,\tau \right)-T_{0}^{\mathrm{III}}\left(\tau \right)S^{\mathrm{III}}\left(x,\tau \right)$, respectively.

(d) In regime IV (λ>1 and 0<γ<1), for a fixed τ, in order to ensure the continuity requirement for fidelity temperature on the characteristic line located at infinity, when λ is infinite in value, $T^{\mathrm{IV}}\left(x,\tau \right)$ is shifted to $T^{\mathrm{IV}}\left(x,\tau \right)-T_{0}^{\mathrm{IV}}\left(\tau \right)$, with $T_{0}^{\mathrm{IV}}\left(\tau \right)=T^{\mathrm{IV}}\left(1,\tau \right)$, accompanied by a shift in $U^{\mathrm{IV}}\left(x,\tau \right)$: $U^{\mathrm{IV}}\left(x,\tau \right)-T_{0}^{\mathrm{IV}}\left(\tau \right)S^{\mathrm{IV}}\left(x,\tau \right)$, with $S^{\mathrm{IV}}\left(x,\tau \right)$ left intact. In addition, fidelity internal energy on the line of the Ising critical points (λ=1 with 0<γ<1) with $x=x_{c}=0$, for a fixed τ, must be zero, and fidelity internal energy satisfies the continuity requirement on the characteristic line located at infinity, when λ is infinite in value, as discussed in Section 2: $U_{0}^{\mathrm{IV}}\left(\tau \right)=T_{0}^{\mathrm{IV}}\left(\tau \right)S_{0}^{\mathrm{IV}}\left(\tau \right)$ and $U^{\mathrm{IV}}\left(1,\tau \right)-T_{0}^{\mathrm{IV}}\left(\tau \right)S^{\mathrm{IV}}\left(1,\tau \right)=U_{m}$. Therefore, $V_{0}^{\mathrm{IV}}\left(\tau \right)$ is determined as follows

(A74) $$V_{0}^{\mathrm{IV}}\left(\tau \right)=\frac{U_{m}}{-\ln\left(e^{\mathrm{IV}}\left(1,\tau \right)\right)/\left(e^{\mathrm{IV}}\left(0,\tau \right)\right)V_{1}^{\mathrm{IV}}\left(1,\tau \right)+\alpha^{\mathrm{IV}}\left(1,\tau \right)V_{1}^{\mathrm{IV}}\left(1,\tau \right)\left(S^{\mathrm{IV}}\left(1,\tau \right)-S_{0}^{\mathrm{IV}}\left(\tau \right)\right)}.$$

Once $V_{0}^{\mathrm{IV}}\left(\tau \right)$ and $U_{0}^{\mathrm{IV}}\left(\tau \right)$ are determined, fidelity temperature $T^{\mathrm{IV}}\left(x,\tau \right)$ and fidelity internal energy $U^{\mathrm{IV}}\left(x,\tau \right)$ follow from their counterparts of (A61) and (A58), with the label being changed from II to IV, respectively, given that (λ,γ) and (x,τ) label the same point in the principal regime. Following from our discussions in Section 2, fidelity entropy $S_{f}^{\mathrm{IV}}\left(x,\tau \right)$, fidelity temperature $T_{f}^{\mathrm{IV}}\left(x,\tau \right)$, and fidelity internal energy $U_{f}^{\mathrm{IV}}\left(x,\tau \right)$ take the following form: $S_{f}^{\mathrm{IV}}\left(x,\tau \right)=S^{\mathrm{IV}}\left(x,\tau \right)$, $T_{f}^{\mathrm{IV}}\left(x,\tau \right)=T^{\mathrm{IV}}\left(x,\tau \right)-T_{0}^{\mathrm{IV}}\left(\tau \right)$, and $U_{f}^{\mathrm{IV}}\left(x,\tau \right)$ $=U^{\mathrm{IV}}\left(x,\tau \right)-T_{0}^{\mathrm{IV}}\left(\tau \right)S^{\mathrm{IV}}\left(x,\tau \right)$, respectively.

(e) In regime V ( λ>1 and γ>1), for a fixed γ, in order to ensure the continuity requirement for fidelity temperature on the characteristic line located at infinity, when λ is infinite in value, fidelity temperature $T^{V}\left(x,\tau \right)$ is shifted to $T^{V}\left(x,\tau \right)-T_{0}^{V}\left(\tau \right)$, with $T_{0}^{V}\left(\tau \right)=T^{V}\left(1,\tau \right)$, accompanied by a shift in $U^{V}\left(x,\tau \right)$: $U^{V}\left(x,\tau \right)-T_{0}^{V}\left(\tau \right)S^{V}\left(x,\tau \right)$, with $S^{V}\left(x,\tau \right)$ left intact. In addition, fidelity internal energy on the line of the Ising critical points (λ=1 with γ∈(1,∞)) with $x=x_{c}=0$, for a fixed τ, must be zero, and fidelity internal energy satisfies the continuity requirement on the characteristic line located at infinity, when λ is infinite in value, as discussed in Section 2: $U_{0}^{V}\left(\tau \right)=T_{0}^{V}\left(\tau \right)S_{0}^{V}\left(\tau \right)$ and $U^{V}\left(1,\tau \right)-T_{0}^{V}\left(\tau \right)S^{V}\left(1,\tau \right)=U_{m}$. Therefore, $V_{0}^{V}\left(\tau \right)$ is determined as follows

(A75) $$V_{0}^{V}\left(\tau \right)=\frac{U_{m}}{-\ln\left(e^{V}\left(1,\tau \right)\right)/\left(e^{V}\left(0,\tau \right)\right)V_{1}^{V}\left(1,\tau \right)+\alpha^{V}\left(1,\tau \right)V_{1}^{V}\left(1,\tau \right)\left(S^{V}\left(1,\tau \right)-S_{0}^{V}\left(\tau \right)\right)}.$$

Once $V_{0}^{V}\left(\tau \right)$ and $U_{0}^{V}\left(\tau \right)$ are determined, fidelity temperature $T^{V}\left(x,\tau \right)$ and fidelity internal energy $U^{V}\left(x,\tau \right)$ follow from their counterparts of (A58) and (A61), with the label being changed from II to V, respectively. Following from our discussions in Section 2, fidelity entropy $S_{f}^{V}\left(x,\tau \right)$, fidelity temperature $T_{f}^{V}\left(x,\tau \right)$, and fidelity internal energy $U_{f}^{V}\left(x,\tau \right)$ take the following form: $S_{f}^{V}\left(x,\tau \right)=S^{V}\left(x,\tau \right)$, $T_{f}^{V}\left(x,\tau \right)=T^{V}\left(x,\tau \right)-T_{0}^{V}\left(\tau \right)$, and $U_{f}^{V}\left(x,\tau \right)=U^{V}\left(x,\tau \right)-T_{0}^{V}\left(\tau \right)S^{V}\left(x,\tau \right)$, respectively.

Once fidelity entropy $S_{f}^{q}\left(x\right)$, fidelity temperature $T_{f}^{q}\left(x\right)$, and fidelity internal energy $U_{f}^{q}\left(x\right)$ (q=i, iii, and v) on the characteristic lines and fidelity entropy $S_{f}^{\omega }\left(x,\tau \right)$, fidelity temperature $T_{f}^{\omega }\left(x,\tau \right)$, and fidelity internal energy $U_{f}^{\omega }\left(x,\tau \right)$ (ω = I, II, III, IV, and V) in the five principal regimes are determined, fidelity entropy $S_{f}\left(\lambda ,\gamma \right)$, fidelity temperature $T_{f}\left(\lambda ,\gamma \right)$, and fidelity internal energy $U_{f}\left(\lambda ,\gamma \right)$ follow. That is, $S_{f}^{q}\left(\lambda ,\gamma \right)\equiv S_{f}^{q}\left(x\right)$ and $S_{f}^{\omega }\left(\lambda ,\gamma \right)\equiv S_{f}^{\omega }\left(x,\tau \right)$, $T_{f}^{q}\left(\lambda ,\gamma \right)\equiv T_{f}^{q}\left(x\right)$ and $T_{f}^{\omega }\left(\lambda ,\gamma \right)\equiv T_{f}^{\omega }\left(x,\tau \right)$, and $U_{f}^{q}\left(\lambda ,\gamma \right)\equiv U_{f}^{q}\left(x\right)$ and $U_{f}^{\omega }\left(\lambda ,\gamma \right)\equiv U_{f}^{\omega }\left(x,\tau \right)$, when we move from *x* and τ to λ and γ, meaning that *x* and τ are regarded as functions of λ and γ. This is due to the fact that (λ,γ) and (x,τ) label the same point on each characteristic line and in each principal regime.

Numerical simulation results for fidelity entropy $S_{f}\left(\lambda ,\gamma \right)$, fidelity temperature $T_{f}\left(\lambda ,\gamma \right)$, and fidelity internal energy $U_{f}\left(\lambda ,\gamma \right)$ for the quantum spin-1/2 XY model are shown in Figure 12a–c, respectively.

### Appendix K.2. Fidelity Entropy $S_{f}\left(1,\gamma \right)$, Fidelity Temperature $T_{f}\left(1,\gamma \right)$, and Fidelity Internal Energy $U_{f}\left(1,\gamma \right)$: An Interior Point of View

For the quantum spin-1/2 XY model, there are three lines of critical points: one line of the Gaussian critical points located at γ=0 with −1<λ<1, with central charge c=1, and the two lines of the Ising critical points located at λ=±1 with γ>0, with central charge c=1/2, if we restrict to the region: γ>0. However, the two lines of the Ising critical points located at λ=±1 with γ>0 are symmetrical with respect to γ→−γ. Therefore, we only need to consider the line of the Ising critical points (λ=1 with γ>0). We remark that the model is critical, with central charge c=1, when γ is infinite in value, with the symmetry group U(1) being generated by $\sum_{i}{\left(-1\right)}^{i}S_{i}^{z}$.

On the line of the Gaussian critical points located at γ=0 with −1<λ<1, which has been described as scenario II-1 in Section 6, any two ground states with two different values of λ are orthogonal to each other as a result of the level crossings, since $\sum_{i}S_{i}^{z}$ is conserved. As a consequence, the ground-state fidelity per lattice site between any two ground states vanishes. This implies that no dominant control parameter is available on the line of the Gaussian critical points for the quantum spin-1/2 XY model, unless it is embedded into the quantum spin-1/2 XYZ model in a magnetic field (cf. Appendix N).

On the line of the Ising critical points located at λ=1 with γ>0, which has been described as scenario I-1 in Section 6, there is a characteristic point γ=1, which is self-dual, in contrast to other points. Therefore, the line of the Ising critical points may be divided into two parts: part a is from γ=0 to γ=1 and part b is from γ=∞ to γ=1, both of which are principal parts. A dominate control parameter x is chosen to be γ in part a. However, the extent of γ is not finite in part b. Therefore, a re-parametrization operation needs to be performed in the ground-state energy density e(1,γ): $e\left(1,\gamma \right)=m^{b}\left(x\right)e^{b}\left(x\right)$, with $m^{b}\left(x\right)=1/x$. Accordingly, a dominant control parameter x is chosen to be x=1/γ in part b.

Let us start from fidelity entropy S(x) in part a and part b:

In part a, from Equation (9), fidelity entropy $S^{a}\left(x\right)$ takes the following form

(A76) $$S^{a}\left(x\right)=-2\int_{x_{c}}^{x}\ln d^{a}\left(x;y\right)\;dy+S_{0}^{a}.$$

Here, $d^{a}\left(x;y\right)$ denotes the ground-state fidelity per lattice site in part a, and $S_{0}^{a}$ is the residual fidelity entropy at the transition point $x_{c}=0$, labelled as PT. According to our convention (cf. Section 2), we have $S_{f}^{a}\left(x\right)=S^{a}\left(x\right)$.

In part b, a re-parametrization operation in the ground-state energy density e(1,γ): $e\left(1,\gamma \right)=m^{b}\left(x\right)e^{b}\left(x\right)$, with $m^{b}\left(x\right)=1/x$, is performed. As discussed in Section 2, $S_{f}^{b}\left(x\right)$ includes contributions from fidelity entropy $S^{b}\left(x\right)$ and from scaling entropy $S_{\sigma }^{b}\left(x\right)$, with $S_{\sigma }^{b}\left(x\right)=\ln x$. Thus, we have $S_{f}^{b}\left(x\right)=S^{b}\left(x\right)+S_{\sigma }^{b}\left(x\right)$, where fidelity entropy $S^{b}\left(x\right)$, as follows from Equation (9), takes the same form as Equation (A76) for part a, with the label being changed from a to b.

Once fidelity entropy $S_{f}\left(x\right)$ is determined, fidelity internal energy $U_{f}\left(x\right)$ and fidelity temperature $T_{f}\left(x\right)$ may be determined from solving the singular first-order differential equation, Equation (14), as discussed for continuous QPTs in Section 2.

In part a, for the chosen dominant control parameter x=γ, the ground-state energy density $e^{a}\left(x\right)$ monotonically decreases with x. Then, fidelity internal energy $U^{a}\left(x\right)$ takes the following form

(A77) $$U^{a}\left(x\right)=\ln\frac{e^{a}\left(x\right)}{e^{a}\left(0\right)}V^{a}\left(x\right)+U_{0}^{a}.$$

Here, $U_{0}^{a}$ is an additive constant, and $V^{a}\left(x\right)>0$ satisfies the singular first-order differential equation:

(A78) $$\frac{\partial V^{a}\left(x\right)}{\partial x}=\alpha^{a}\left(x\right)\;V^{a}\left(x\right),$$

with

(A79) $$\alpha^{a}\left(x\right)=-\frac{\partial \ln\left(e^{a}\left(x\right)/e^{a}\left(0\right)\right)/\partial x}{\partial S^{a}\left(x\right)/\partial x+\ln\left(e^{a}\left(x\right)/e^{a}\left(0\right)\right)}.$$

Accordingly, fidelity temperature $T^{a}\left(x\right)$ in this part follows from

(A80) $$T^{a}\left(x\right)=-\frac{\partial V^{a}\left(x\right)}{\partial x}.$$

In part b, fidelity internal energy $U^{b}\left(x\right)$, fidelity temperature $T^{b}\left(x\right)$, and $V^{b}\left(x\right)$ together with its singular first-order differential equation with $\alpha^{b}\left(x\right)$ take the same form as equations, Equations (A77)–(A80), for part a, with the label being changed from a to b.

In order to solve a singular first-order differential equation in each part, we perform a scaling analysis of $\alpha^{a}\left(x\right)$ and $\alpha^{b}\left(x\right)$ near a transition point.

Near the point $x_{c}=0$ for the two parts, fidelity entropy $S^{a}\left(x\right)$ and $S^{b}\left(x\right)$ scale as $S^{a}\left(x\right)∼x^{2}$ and $S^{b}\left(x\right)∼x^{2}$, respectively. Combined with a scaling analysis of the ground-state energy density $e^{a}\left(x\right)$ and $e^{b}\left(x\right)$ near $x_{c}=0$: $\ln\left(e^{a}\left(x\right)/e^{a}\left(0\right)\right)∼\ln{\mathrm{xx}}^{2}$ and $\ln\left(e^{b}\left(x\right)/e^{b}\left(x\right)\right)∼\ln{\mathrm{xx}}^{2}$, we have the following:

(A81) $$\alpha^{a}\left(x\right)∼\ln\left(1-x\right),$$

and

(A82) $$\alpha^{b}\left(x\right)∼\ln\left(1-x\right).$$

Our numerical simulations confirm this scaling analysis.

This enables us to solve a singular first-order differential equation, as shown below.

(a) In Part a: λ=1 and 0<γ<1, the singular first-order differential equation, Equation (A78), may be solved as follows:

(A83) $$V^{a}\left(x\right)=V_{0}^{a}V_{1}^{a}\left(x\right),$$

where $V_{0}^{a}$ is a constant, and $V_{1}^{a}\left(x\right)$ takes the following form.

(A84) $$V_{1}^{a}\left(x\right)=\exp\left(\int_{0}^{x}\alpha^{a}\left(y\right)dy\right).$$

(b) In part b, the solution $V^{b}\left(x\right)$ to the singular first-order differential equation takes the same form as Equations (A83) and (A84) for part a, with the label being changed from a to b.

The remaining task is to determine $S_{0}$, $U_{0}$, and $V_{0}$ in the two parts. In part a, we require that fidelity entropy from an interior point of view at the transition point, labelled as PT, is single-valued. That is, $S_{f}^{a}\left(x\right)$ from an interior point of view is equal to $S_{m}$, which is fidelity entropy from an exterior point of view at the transition point x=0, labelled as PT. Therefore, $S_{0}^{a}$ is determined: $S_{0}^{a}=S_{m}$. With this in mind, $S_{0}^{b}$ is determined from the continuity requirement for $S_{f}^{b}\left(x\right)$ at the self-dual point with x=1: $S_{f}^{b}\left(1\right)=S_{f}^{a}\left(1\right)$. In addition, at the self-dual point, we require fidelity temperature from an interior point of view to be zero, and fidelity internal energy from an interior point of view should be equal to fidelity internal energy from an exterior point of view at the U(1)-symmetric point (0,1) for the quantum spin-1/2 XY model, denoted as $U_{m}$. As argued in Section 2, for this purpose, a shift in fidelity temperature is performed: $T^{a}\left(x\right)-T_{0}^{a}$ and $T^{b}\left(x\right)-T_{0}^{b}$ accompanied by a shift in fidelity internal energy—$U^{a}\left(x\right)-T_{0}^{a}S^{a}\left(x\right)$ and $U^{b}\left(x\right)-T_{0}^{b}S^{b}\left(x\right)$, respectively. Therefore, we need to determine $T_{0}^{a}$ and $T_{0}^{b}$ for principal parts a and b, respectively. In part a, we have $T_{0}^{a}\equiv T^{a}\left(1\right)$, where $T^{a}\left(1\right)$ represents fidelity temperature $T^{a}\left(x\right)$ at the self-dual point x=1, evaluated from a dominant control parameter x in part a. In part b, we have $T_{0}^{b}\equiv T^{b}\left(1\right)$, where $T^{b}\left(1\right)$ represents fidelity temperature $T^{b}\left(x\right)$ at the self-dual point x=1, evaluated from a dominant control parameter x in part b. Specifically, in part a, in order to ensure the requirement for fidelity temperature at the self dual point with x=1, shifts in fidelity temperature from $T^{a}\left(x\right)$ to $T^{a}\left(x\right)-T_{0}^{a}$ and in fidelity internal energy from $U^{a}\left(x\right)$ to $U^{a}\left(x\right)-T_{0}^{a}S^{a}\left(x\right)$ are performed. In addition, fidelity internal energy at the transition point with x=0, labelled as PT, must be zero, and fidelity internal energy at the self-dual point with x=1 satisfies the continuity requirement, as discussed in Section 2: $U_{0}^{a}=T_{0}^{a}S_{0}^{a}$ and $U^{a}\left(1\right)-T_{0}^{a}S^{a}\left(1\right)=U_{m}$. Hence, $V_{0}^{a}$ is determined as follows

(A85) $$V_{0}^{a}=\frac{U_{m}}{\ln\left(e^{a}\left(1\right)/e^{a}\left(0\right)\right)V_{1}^{a}\left(1\right)+\alpha^{a}\left(1\right)V_{1}^{a}\left(1\right)\left(S^{a}\left(1\right)-S_{0}^{a}\right)}.$$

Once $V_{0}^{a}$ and $U_{0}^{a}$ are determined, fidelity temperature $T^{a}\left(x\right)$ and fidelity internal energy $U^{a}\left(x\right)$ follow from (A77) and (A80), respectively. We refer to $T^{a}\left(x\right)-T_{0}^{a}$ and $U^{a}\left(x\right)$ $-T_{0}^{a}S^{a}\left(x\right)$ as $T_{f}^{a}\left(x\right)$ and $U_{f}^{a}\left(x\right)$, respectively. That is, $T_{f}^{a}\left(x\right)\equiv T^{a}\left(x\right)-T_{0}^{a}$ and $U_{f}^{a}\left(x\right)$ $\equiv U^{a}\left(x\right)-T_{0}^{a}S^{a}\left(x\right)$.

In part b, in order to ensure the requirement for fidelity temperature at the self dual point with x=1, shifts in fidelity temperature from $T^{b}\left(x\right)$ to $T^{b}\left(x\right)-T_{0}^{b}$ and in fidelity internal energy from $U^{b}\left(x\right)$ to $U^{b}\left(x\right)-T_{0}^{b}S^{b}\left(x\right)$ are performed. In addition, fidelity internal energy, when x tends to 0, must be zero and fidelity internal energy at the self-dual point x=1, satisfies the continuity requirement, as discussed in Section 2: $U_{0}^{b}=T_{0}^{b}S_{0}^{b}$ and $U^{b}\left(1\right)-T_{0}^{b}S^{b}\left(1\right)=U_{m}$. Hence, $V_{0}^{b}$ is determined as follows

(A86) $$V_{0}^{b}=\frac{U_{m}}{\ln\left(e^{b}\left(1\right)/e^{b}\left(0\right)\right)V_{1}^{b}\left(1\right)+\alpha^{b}\left(1\right)V_{1}^{b}\left(1\right)\left(S^{b}\left(1\right)-S_{0}^{b}\right)}.$$

Once $V_{0}^{b}$ and $U_{0}^{b}$ are determined, fidelity temperature $T^{b}\left(x\right)$ and fidelity internal energy $U^{b}\left(x\right)$ follow from their counterparts of (A77) and (A80), with the label being changed from a to b, respectively. We refer to $T^{b}\left(x\right)-T_{0}^{b}$ and $U^{b}\left(x\right)-T_{0}^{b}S^{b}\left(x\right)$ as $T_{f}^{b}\left(x\right)$ and $U_{f}^{b}\left(x\right)$, respectively. That is, $T_{f}^{b}\left(x\right)\equiv T^{b}\left(x\right)-T_{0}^{b}$ and $U_{f}^{b}\left(x\right)\equiv U^{b}\left(x\right)-T_{0}^{b}S^{b}\left(x\right)$.

Once fidelity entropy $S_{f}^{q}\left(x\right)$, fidelity temperature $T_{f}^{q}\left(x\right)$, and fidelity internal energy $U_{f}^{q}\left(x\right)$ (*q*=a and b) are determined for the two parts, fidelity entropy $S_{f}\left(1,\gamma \right)$, fidelity temperature $T_{f}\left(1,\gamma \right)$, and fidelity internal energy $U_{f}\left(1,\gamma \right)$ follow, since they take the same values as $S_{f}^{q}\left(x\right)$, $T_{f}^{q}\left(x\right)$, and $U_{f}^{q}\left(x\right)$, respectively. That is, $S_{f}^{q}\left(1,\gamma \right)\equiv S_{f}^{q}\left(x\right)$, $T_{f}^{q}\left(1,\gamma \right)\equiv T_{f}^{q}\left(x\right)$, and $U_{f}^{q}\left(1,\gamma \right)\equiv U_{f}^{q}\left(x\right)$, when we move from *x* to γ, meaning that *x* is regarded as a function of γ.

Numerical simulation results for fidelity entropy $S_{f}\left(1,\gamma \right)$, fidelity temperature $T_{f}\left(1,\gamma \right)$, and fidelity internal energy $U_{f}\left(1,\gamma \right)$ on the line of the Ising critical points located at λ=1 with γ>0 are shown in Figure 14a–c, respectively.

## Appendix L. Fidelity Entropy $S_{f}\left(\lambda ,h\right)$, Fidelity Temperature $T_{f}\left(\lambda ,h\right)$, and Fidelity Internal Energy $U_{f}\left(\lambda ,h\right)$ for the Transverse-Field Quantum Ising Model in a Longitudinal Field

In this Appendix, we present mathematical details about fidelity entropy $S_{f}\left(\lambda ,h\right)$, fidelity temperature $T_{f}\left(\lambda ,h\right)$, and fidelity internal energy $U_{f}\left(\lambda ,h\right)$ for the transverse-field quantum Ising model in a longitudinal field.

For this model, the ground-state phase diagram is simple. A first-order QPT occurs at h=0, when 0≤λ<1, which ends at a critical point (1,0). At the first-order QPT points, the model is driven from a phase with spin polarization in −x to a phase with spin polarization in x, when h changes its sign. When h=0, the model becomes the transverse-field Ising model, which exhibits a second-order QPT at $\lambda_{c}=1$, characterized by the $Z_{2}$ symmetry-breaking order for λ<1. As mentioned in Appendix K, duality occurs for the transverse-field quantum Ising model. We remark that, when h=0, fidelity mechanical-state functions have been determined as a special case of the quantum spin-1/2 XY model, corresponding to γ=1. Therefore, we restrict ourselves to the region λ≥0 and h≥0.

With the symmetry and duality in mind, we may divide the region λ≥0 and h≥0 into two principal regimes, as shown in Section 2. They are labelled as regime I, defined as 0≤λ<1 and h≥0, and regime II, defined as λ≥1 and h≥0.

Now we turn to the explicit mathematical expressions for fidelity entropy $S_{f}\left(\lambda ,h\right)$, fidelity temperature $T_{f}\left(\lambda ,h\right)$, and fidelity internal energy $U_{f}\left(\lambda ,h\right)$ for the transverse-field quantum Ising model in a longitudinal field.

We recall the choices of a dominant control parameter in the two regimes:

(a) In regime I (0≤λ<1 and h≥0), a dominant control parameter x was chosen to be x=h/(1+h) and an auxiliary control parameter τ was chosen to be τ=λ. In this regime, a re-parametrization operation in the ground-state energy density e(λ,h): $e\left(\lambda ,h\right)=m^{I}\left(x,\tau \right)\;e^{I}\left(x,\tau \right)$ is performed, with $m^{I}\left(x,\tau \right)=1/\left(1-x\right)$, to ensure that $e^{I}\left(x,\tau \right)$ is monotonic with x. Note that if x=0 or, equivalently, h=0, then the model reduces to the transverse-field Ising model, which in turn is a special case of the quantum spin-1/2 XY model with γ=1 (cf. Section 7 and Appendix K). As a result, fidelity mechanical-state functions for x=0 may be taken from the quantum spin-1/2 XY model with γ=1 (cf. Appendix K). In this Appendix, fidelity mechanical-state functions for the transverse-field Ising model are denoted as $S^{\mathrm{TFI}}\left(\tau \right)$, $T^{\mathrm{TFI}}\left(\tau \right)$ and $U^{\mathrm{TFI}}\left(\tau \right)$, with τ=λ, respectively.
(b) In regime II (λ≥1 and h≥0), a dominant control parameter x was chosen to be $x=\sqrt{{\left(\lambda -1\right)}^{2}+h^{2}}/\left(1+\sqrt{{\left(\lambda -1\right)}^{2}+h^{2}}\right)$ and an auxiliary control parameter τ was chosen to be τ=arctan(h/(λ−1))∈(0,π/2). The ranges of x and τ are 0<x<1 and 0<τ<π/2, respectively. In this regime, a re-parametrization operation in the ground-state energy density e(λ,h): $e\left(\lambda ,h\right)=m^{\mathrm{II}}\left(x,\tau \right)\;e^{\mathrm{II}}\left(x,\tau \right)$, is performed, with $m^{\mathrm{II}}\left(x,\tau \right)=1/\left(1-x\right)$. This choice is consistent with the requirement from duality, which occurs when τ=0, corresponding to the transverse-field quantum Ising model.

Fidelity entropy $S_{f}\left(x,\tau \right)$, fidelity temperature $T_{f}\left(x,\tau \right)$, and fidelity internal energy $U_{f}\left(x,\tau \right)$ may be determined following from our prescriptions for discontinuous QPTs (regime I) and for continuous QPTs (regime II), as discussed in Section 2.

(a) In regime I (0≤λ<1 and h≥0), a re-parametrization operation in the ground-state energy density e(λ,h): $e\left(\lambda ,h\right)=m^{I}\left(x,\tau \right)\;e^{I}\left(x,\tau \right)$, with $m^{I}\left(x,\tau \right)=1/\left(1-x\right)$, is performed, and fidelity entropy $S_{f}^{I}\left(x,\tau \right)$ includes contributions from both fidelity entropy $S_{f}^{I}\left(x,\tau \right)$ and scaling entropy $S_{\sigma }^{I}\left(x,\tau \right)$, as discussed in Section 2. Thus, we have $S_{f}^{I}\left(x,\tau \right)\equiv S^{I}\left(x,\tau \right)+S_{\sigma }^{I}\left(x,\tau \right)$. Here, $S_{\sigma }^{I}\left(x,\tau \right)=\ln m^{I}\left(x,\tau \right)$, and $S^{I}\left(x,\tau \right)$ follows from definition (33). For a fixed τ, $S^{I}\left(x,\tau \right)$ takes the following form

(A87) $$S^{I}\left(x,\tau \right)=-2\int_{x_{d}}^{x}\ln d^{I}\left(x,\tau ;y,\tau \right)\;dy+S_{0}^{I}\left(\tau \right).$$

Here, $d^{I}\left(x,\tau ;y,\tau \right)$ denotes the ground-state fidelity per lattice site in regime I, and $S_{0}^{I}\left(\tau \right)$ is the residual fidelity entropy at a discontinuous QPT point $x_{d}$ for a fixed τ, with $x_{d}=0$. The continuity requirement for fidelity entropy $S_{f}\left(x,\tau \right)$ at $x_{d}=0$ implies that $S_{0}^{I}\left(\tau \right)=S^{\mathrm{TFI}}\left(\tau \right)$.

In regime I, for a fixed τ, the ground-state energy density $e^{I}\left(x,\tau \right)$ monotonically increases with x. Then, from Equation (35), fidelity internal energy $U^{I}\left(x,\tau \right)$ takes the following form

(A88) $$U^{I}\left(x,\tau \right)=-\left[\ln\kappa +\ln\frac{e^{I}\left(x,\tau \right)}{e^{I}\left(0,\tau \right)}\right]V^{I}\left(x,\tau \right)+U_{0}^{I}\left(\tau \right).$$

Here, $U_{0}^{I}\left(\tau \right)$ is a function of τ, and $V^{I}\left(x,\tau \right)>0$ satisfies the regular first-order differential equation

(A89) $$\frac{\partial V^{I}\left(x,\tau \right)}{\partial x}=\alpha^{I}\left(x,\tau \right)\;V^{I}\left(x,\tau \right),$$

with

(A90) $$\alpha^{I}\left(x,\tau \right)=\frac{\partial \ln\left(e^{I}\left(x,\tau \right)/e^{I}\left(0,\tau \right)\right)/\partial x}{\partial S^{I}\left(x,\tau \right)/\partial x-\left(\ln\kappa +\ln\left(e^{I}\left(x,\tau \right)/e^{I}\left(0,\tau \right)\right)\right)}.$$

Accordingly, fidelity temperature $T^{I}\left(x,\tau \right)$ in this regime is given by the following

(A91) $$T^{I}\left(x,\tau \right)=-\frac{\partial V^{I}\left(x,\tau \right)}{\partial x}.$$

In regime I, we have to ensure that fidelity mechanical-state functions are *continuous* at the boundaries. Indeed, we have already taken into account the continuity requirement for fidelity entropy, thus determining the residual fidelity entropy on the line of discontinuous QPT points and the critical point $x_{c}=0$ for a fixed τ=1. The remaining task is to ensure the continuity requirements for fidelity temperature and fidelity internal energy. In addition to the dual line (x=0), there is another characteristic line (x=1 with 0<τ<1), since a factorized ground state occurs at x=1, thus leading to zero-fidelity temperature, with fidelity internal energy being $U^{\mathrm{TFI}}\left(0\right)$. To this end, we need to determine $T_{0}^{I}\left(\tau \right)$ $\equiv T_{m}^{I}\left(\tau \right)-T_{t}^{I}\left(\tau \right)$ for the regime. For a fixed τ, we have $T_{m}^{I}\left(\tau \right)=T^{I}\left(1,\tau \right)$ and $T_{t}^{I}\left(\tau \right)=0$. Furthermore, in order to ensure the continuity requirement for fidelity temperature on the line of discontinuous QPT points and the requirement for fidelity temperature T(x,τ) to be zero when h is infinite in value, for a fixed τ, $T^{I}\left(x,\tau \right)$ is shifted to $T^{I}\left(x,\tau \right)-T_{0}^{I}\left(\tau \right)$ and $U^{I}\left(x,\tau \right)$ is shifted to $U^{I}\left(x,\tau \right)-T_{0}^{I}\left(\tau \right)S^{I}\left(x,\tau \right)$. At x=0, the continuity requirements for fidelity temperature and fidelity internal energy imply that $T^{I}\left(0,\tau \right)-T_{0}^{I}\left(\tau \right)=T^{\mathrm{TFI}}\left(\tau \right)$ and $U^{I}\left(0,\tau \right)-T_{0}^{I}\left(\tau \right)S^{\mathrm{TFI}}\left(\tau \right)=U^{\mathrm{TFI}}\left(\tau \right)$. On the other hand, at x=1, the continuity requirement for fidelity internal energy implies that $U^{I}\left(1,\tau \right)-T_{0}^{I}\left(\tau \right)S^{I}\left(1,\tau \right)=U^{\mathrm{TFI}}\left(0\right)$. Therefore, κ, $U_{0}^{I}\left(\tau \right)$, and $V_{0}^{I}\left(\tau \right)$ are determined from these continuity requirements for a fixed τ. We refer to $T^{I}\left(x,\tau \right)-T_{0}^{I}\left(\tau \right)$ and $U^{I}\left(x,\tau \right)-T_{0}^{I}\left(\tau \right)S^{I}\left(x,\tau \right)$ as $T_{f}^{I}\left(x,\tau \right)$ and $U_{f}^{I}\left(x,\tau \right)$. That is, $T_{f}^{I}\left(x,\tau \right)\equiv T^{I}\left(x,\tau \right)-T_{0}^{I}\left(\tau \right)$ and $U_{f}^{I}\left(x,\tau \right)\equiv U^{I}\left(x,\tau \right)-T_{0}^{I}\left(\tau \right)S^{I}\left(x,\tau \right)$.

The model is not exactly solvable when h≠0. Instead, we simulate it numerically to evaluate ground-state wave functions by exploiting the iTEBD algorithm [39,40,41,46,47,48] in the matrix-product state representation, with the bond dimension χ ranging from 20 to 200. This allows us to determine, among others, parameter lnκ, as plotted in Figure A10a. It approaches zero when τ→0 and τ→1 and exhibits a minimum around τ=0.33.

(b) In regime II (λ≥1 and h≥0), the critical point, located at λ=1 and h=0, controls the underlying physics. In this regime, fidelity mechanical-state functions are evaluated from our prescription for continuous QPTs in Section 2. For the chosen dominant control parameter x: $x=\sqrt{{\left(\lambda -1\right)}^{2}+h^{2}}$, the ground-state energy density $e^{\mathrm{II}}\left(x,\tau \right)$ is monotonic with x, for a fixed τ. As discussed in Section 2, $S_{f}^{\mathrm{II}}\left(x,\tau \right)$ includes contributions from both fidelity entropy $S_{f}^{\mathrm{II}}\left(x,\tau \right)$ and scaling entropy $S_{\sigma }^{\mathrm{II}}\left(x,\tau \right)$. Thus, we have $S_{f}^{\mathrm{II}}\left(x,\tau \right)\equiv S^{\mathrm{II}}\left(x,\tau \right)+S_{\sigma }^{\mathrm{II}}\left(x,\tau \right)$, with $S_{\sigma }^{\mathrm{II}}\left(x,\tau \right)=\ln m^{\mathrm{II}}\left(x,\tau \right)$, and $S_{f}^{\mathrm{II}}\left(x,\tau \right)$ follows from definition (28). For a fixed τ, $S^{\mathrm{II}}\left(x,\tau \right)$ takes the following form

(A92) $$S^{\mathrm{II}}\left(x,\tau \right)=-2\int_{x_{c}}^{x}\ln d^{\mathrm{II}}\left(x,\tau ;y,\tau \right)\;dy+S_{0}^{\mathrm{II}}\left(\tau \right).$$

Here, $d^{\mathrm{II}}\left(x,\tau ;y,\tau \right)$ denotes the ground-state fidelity per lattice site in regime II, and $S_{0}^{\mathrm{II}}\left(\tau \right)$ is the residual fidelity entropy at the critical point $x_{c}$ for a fixed τ, with $x_{c}=0$, or equivalently λ=1 and h=0. Therefore, $S_{0}^{\mathrm{II}}\left(\tau \right)$ is identified to be fidelity entropy for the transverse-field quantum Ising model at the same critical point. That is, $S_{0}^{\mathrm{II}}\left(\tau \right)=S^{\mathrm{TFI}}\left(1\right)$ for a fixed τ.

In regime II, for a fixed τ, $e^{\mathrm{II}}\left(x,\tau \right)$ monotonically increases with x. Then, from Equation (30), fidelity internal energy $U^{\mathrm{II}}\left(x,\tau \right)$ takes the following form

(A93) $$U^{\mathrm{II}}\left(x,\tau \right)=-\ln\frac{e^{\mathrm{II}}\left(x,\tau \right)}{e^{\mathrm{II}}\left(0,\tau \right)}V^{\mathrm{II}}\left(x,\tau \right)+U_{0}^{\mathrm{II}}\left(\tau \right).$$

Here, $U_{0}^{\mathrm{II}}\left(\tau \right)$ is a function of τ, and $V^{\mathrm{II}}\left(x,\tau \right)>0$ satisfies the singular first-order differential equation:

(A94) $$\frac{\partial V^{\mathrm{II}}\left(x,\tau \right)}{\partial x}=\alpha^{\mathrm{II}}\left(x,\tau \right)\;V^{\mathrm{II}}\left(x,\tau \right),$$

with

(A95) $$\alpha^{\mathrm{II}}\left(x,\tau \right)=\frac{\partial \ln\left(e^{\mathrm{II}}\left(x,\tau \right)/e^{\mathrm{II}}\left(0,\tau \right)\right)/\partial x}{\partial S^{\mathrm{II}}\left(x,\tau \right)/\partial x-\ln\left(e^{\mathrm{II}}\left(x,\tau \right)/e^{\mathrm{II}}\left(0,\tau \right)\right)}.$$

Accordingly, fidelity temperature $T^{\mathrm{II}}\left(x,\tau \right)$ in this regime is given by the following

(A96) $$T^{\mathrm{II}}\left(x,\tau \right)=-\frac{\partial V^{\mathrm{II}}\left(x,\tau \right)}{\partial x}.$$

In order to solve a singular first-order differential equation in the regime, we analyze the scaling behavior of $\alpha^{\mathrm{II}}\left(x,\tau \right)$ in the vicinity of the critical point. For τ≠0, we find that the critical exponent ν takes ν≃0.5, with τ=π/4 as an example, shown in Figure A10b. Thus, fidelity entropy $S^{\mathrm{II}}\left(x,\tau \right)$ scales as $x^{3/2}$. This is consistent with a general scaling analysis in Appendix H, which predicts that $S^{\mathrm{II}}\left(x,\tau \right)∼x^{\nu +1}$. Therefore, for a fixed τ, $\alpha^{\mathrm{II}}\left(x,\tau \right)$ diverges when x tends to $x_{c}$, with $x_{c}=0$, as follows

(A97) $$\alpha^{\mathrm{II}}\left(x,\tau \right)\propto \frac{1}{x^{1/2}}.$$

For a fixed τ, the singular first-order differential equation, Equation (A94), may be solved as follows:

(A98) $$V^{\mathrm{II}}\left(x,\tau \right)=V_{0}^{\mathrm{II}}\left(\tau \right)V_{1}^{\mathrm{II}}\left(x,\tau \right),$$

where $V_{0}^{\mathrm{II}}\left(\tau \right)>0$ is a function of τ, and $V_{1}^{\mathrm{II}}\left(x,\tau \right)$ is defined as follows

(A99) $$V_{1}^{\mathrm{II}}\left(x,\tau \right)=\exp\left(\int_{0}^{x}\alpha^{\mathrm{II}}\left(y,\tau \right)dy\right).$$

Therefore, fidelity internal energy $U^{\mathrm{II}}\left(x,\tau \right)$ and fidelity temperature $T^{\mathrm{II}}\left(x,\tau \right)$ follow from (A93) and (A96), respectively.

Now we have to ensure that fidelity mechanical-state functions are *continuous* at the boundaries. Indeed, we have already taken into account the continuity requirement for fidelity entropy, thus determining the residual fidelity entropy at the critical point $x_{c}$ for a fixed τ, with $x_{c}=0$. The remaining task is to ensure the continuity requirements for fidelity temperature and fidelity internal energy. In this regime, there is another characteristic line (x=1 with 0<τ<π/2), since a factorized ground state occurs there, thus leading to zero-fidelity temperature, with fidelity internal energy being $U^{\mathrm{TFI}}\left(0\right)$. To this end, we need to determine $T_{0}^{\mathrm{II}}\left(\tau \right)\equiv T_{m}^{\mathrm{II}}\left(\tau \right)-T_{t}^{\mathrm{II}}\left(\tau \right)$ for regime II. Here, we have $T_{m}^{\mathrm{II}}\left(\tau \right)=T^{\mathrm{II}}\left(1,\tau \right)$ and $T_{t}^{\mathrm{II}}\left(\tau \right)=0$.

In order to ensure that fidelity temperature $T_{f}\left(x,\tau \right)$ vanishes, when x proceeds to 1, for a fixed τ, $T^{\mathrm{II}}\left(x,\tau \right)$ is shifted to $T^{\mathrm{II}}\left(x,\tau \right)-T_{0}^{\mathrm{II}}\left(\tau \right)$, with $T_{0}^{\mathrm{II}}\left(\tau \right)=T^{\mathrm{II}}\left(1,\tau \right)$, accompanied by a shift in $U^{\mathrm{II}}\left(x,\tau \right)$: $U^{\mathrm{II}}\left(x,\tau \right)-T_{0}^{\mathrm{II}}\left(\tau \right)S^{\mathrm{II}}\left(x,\tau \right)$. In addition, fidelity internal energy at the critical point $x_{c}$ for a fixed τ, with $x_{c}=0$, must be zero, and fidelity internal energy at (1,τ) satisfies the continuity requirement, as discussed in Section 2: $U_{0}^{\mathrm{II}}\left(\tau \right)=T_{0}^{\mathrm{II}}\left(\tau \right)S_{0}^{\mathrm{II}}\left(\tau \right)$ and $U^{\mathrm{II}}\left(1,\tau \right)-T_{0}^{\mathrm{II}}\left(\tau \right)S^{\mathrm{II}}\left(1,\tau \right)=U^{\mathrm{TFI}}\left(0\right)$. Hence, $V_{0}^{\mathrm{II}}\left(\tau \right)$ is determined as follows

(A100) $$V_{0}^{\mathrm{II}}\left(\tau \right)=\frac{U^{\mathrm{TFI}}\left(0\right)}{-\ln\left(e^{\mathrm{II}}\left(1,\tau \right)/e^{\mathrm{II}}\left(0,\tau \right)\right)V_{1}^{\mathrm{II}}\left(1,\tau \right)+\alpha^{\mathrm{II}}\left(1,\tau \right)V_{1}^{\mathrm{II}}\left(1,\tau \right)\left(S^{\mathrm{II}}\left(1,\tau \right)-S^{\mathrm{TFI}}\left(1\right)\right)}.$$

After $V_{0}^{\mathrm{II}}\left(\tau \right)$ and $U_{0}^{\mathrm{II}}\left(\tau \right)$ are determined, fidelity temperature $T^{\mathrm{II}}\left(x,\tau \right)$ and fidelity internal energy $U^{\mathrm{II}}\left(x,\tau \right)$ follow from (A93) and (A96), respectively. We refer to $T^{\mathrm{II}}\left(x,\tau \right)-T_{0}^{\mathrm{II}}\left(\tau \right)$ and $U^{\mathrm{II}}\left(x,\tau \right)-T_{0}^{\mathrm{II}}\left(\tau \right)S^{\mathrm{II}}\left(x,\tau \right)$ as $T_{f}^{\mathrm{II}}\left(x,\tau \right)$ and $U_{f}^{\mathrm{II}}\left(x,\tau \right)$, respectively. That is, $T_{f}^{\mathrm{II}}\left(x,\tau \right)$ $\equiv T^{\mathrm{II}}\left(x,\tau \right)-T_{0}^{\mathrm{II}}\left(\tau \right)$ and $U_{f}^{\mathrm{II}}\left(x,\tau \right)\equiv U^{\mathrm{II}}\left(x,\tau \right)-T_{0}^{\mathrm{II}}\left(\tau \right)S^{\mathrm{II}}\left(x,\tau \right)$, respectively.

Once fidelity entropy $S_{f}^{\omega }\left(x,\tau \right)$, fidelity temperature $T_{f}^{\omega }\left(x,\tau \right)$, and fidelity internal energy $U_{f}^{\omega }\left(x,\tau \right)$ (ω = I and II) are determined for the two principal regimes, fidelity entropy $S_{f}\left(\lambda ,h\right)$, fidelity temperature $T_{f}\left(\lambda ,h\right)$, and fidelity internal energy $U_{f}\left(\lambda ,h\right)$ follow. That is, $S_{f}^{\omega }\left(\lambda ,h\right)\equiv S_{f}^{\omega }\left(x,\tau \right)$, $T_{f}^{\omega }\left(\lambda ,h\right)\equiv T_{f}^{\omega }\left(x,\tau \right)$, and $U_{f}^{\omega }\left(\lambda ,h\right)\equiv U_{f}^{\omega }\left(x,\tau \right)$ when we move from x and τ to λ and h, meaning that x and τ are regarded as functions of λ and h.

Numerical simulation results for fidelity entropy $S_{f}\left(\lambda ,h\right)$, fidelity temperature $T_{f}\left(\lambda ,h\right)$, and fidelity internal energy $U_{f}\left(\lambda ,h\right)$ for the transverse-field quantum Ising model in a longitudinal field are shown in Figure 16a–c, respectively.

## Appendix M. Fidelity Entropy $S_{f}\left(\Delta ,\gamma \right)$, Fidelity Temperature $T_{f}\left(\Delta ,\gamma \right)$, and Fidelity Internal Energy $U_{f}\left(\Delta ,\gamma \right)$ for the Quantum Spin-1/2 XYZ Model

In this Appendix, we present mathematical details about fidelity entropy $S_{f}\left(\Delta ,\gamma \right)$, fidelity temperature $T_{f}\left(\Delta ,\gamma \right)$, and fidelity internal energy $U_{f}\left(\Delta ,\gamma \right)$ for the spin-1/2 quantum XYZ model.

For this model, the Hamiltonian (3) is symmetrical under γ↔−γ. Therefore, we may restrict ourselves to the region γ≥0. As shown in Figure 17, there are three different phases, labelled as ${\mathrm{AF}}_{x}$, ${\mathrm{AF}}_{z}$, and ${\mathrm{FM}}_{z}$, representing an AF phase in the x direction, an AF phase in the z direction, and an FM phase in the z direction, respectively. There are three lines of critical points: γ=0 with −1<Δ≤1, γ=−1+Δ with Δ≥1 and γ=−1−Δ with Δ<−1. As demonstrated (cf. Appendix C), there are five different dualities. In addition, there is a characteristic line (γ=1+Δ with Δ>−1), representing factorizing fields [77,78,79].

Taking into account the symmetries, dualities, and factorizing fields, we may divide the region γ≥0 into twelve different regimes, with the characteristic lines, defined by γ=0, γ=1, and γ=±1±Δ, as the boundaries. The twelve regimes are separated into two groups, with six regimes in each group dual to each other. As shown in Section 2, regime I, regime III, regime V, regime VII, regime IX, and regime XI are dual to each other, whereas regime II, regime IV, regime VI, regime VIII, regime X, and regime XII are dual to each other. Therefore, there are only two principal regimes, representing the physics underlying the quantum spin-1/2 XYZ model (3). Here, we choose regime I (0<Δ<1 and 0<γ<1−Δ) and regime II (−1<Δ<0 and 0<γ<1+Δ) as two principal regimes.

Now we turn to the explicit mathematical expressions for fidelity entropy $S_{f}\left(\Delta ,\gamma \right)$, fidelity temperature $T_{f}\left(\Delta ,\gamma \right)$, and fidelity internal energy $U_{f}\left(\Delta ,\gamma \right)$ for the quantum spin-1/2 XYZ model.

### Appendix M.1. Fidelity Entropy $S_{f}\left(\Delta ,\gamma \right)$, Fidelity Temperature $T_{f}\left(\Delta ,\gamma \right)$, and Fidelity Internal Energy $U_{f}\left(\Delta ,\gamma \right)$: An Exterior Point of View

Let us determine fidelity entropy $S^{i}\left(x\right)$ in principal part i on the factorizing-field line (γ=1+Δ with −1<Δ<0), and fidelity entropy $S^{\mathrm{ii}}\left(x\right)$ in principal part ii on the U(1)-symmetric line (γ=1−Δ with 0<Δ<1):

(i) On the factorizing-field line (γ=1+Δ with −1<Δ<0), the same factorized state occurs as the ground-state wave function, with the ground-state energy density e(Δ,1+Δ)=−(Δ+2)/2. We recall that a dominant control parameter x was chosen to be x=Δ+1 for a fixed γ−Δ=1. Here, a re-parametrization operation in the ground-state energy density e(Δ,1+Δ): $e\left(\Delta ,1+\Delta \right)=m^{i}\left(x\right)e^{i}\left(x\right)$, with $m^{i}\left(x\right)=\left(x+1\right)/2$, is performed. Therefore, $e^{i}\left(x\right)$ is a constant as x varies. Note that the quantum spin-1/2 XYZ model becomes the quantum spin-1/2 XY model, when x=1. Therefore, fidelity mechanical-state functions for x=1 has been determined, as discussed in Appendix K. In particular, fidelity entropy $S^{i}\left(1\right)$ for x=1 is known. With this in mind, fidelity entropy $S^{i}\left(x\right)$ on the factorizing-field line (γ=1+Δ with 0<γ<1), is identical to $S^{i}\left(1\right)$ up to scaling entropy $S_{\sigma }^{i}\left(x\right)$. Thus, we have $S_{f}^{i}\left(x\right)=S^{i}\left(1\right)+S_{\sigma }^{i}\left(x\right)$. Here, $S_{\sigma }^{i}\left(x\right)=\ln m^{i}\left(x\right)$, with $m^{i}\left(x\right)=\left(x+1\right)/2$. According to our convention (cf. Section 2), we have $S_{f}^{i}\left(x\right)=S^{i}\left(x\right)$.
(ii) On the U(1)-symmetric line (γ=1−Δ with 0<Δ<1), we recall that a dominant control parameter x was chosen to be x=1−Δ/(2−Δ). Here, a re-parametrization operation in the ground-state energy density e(Δ,1−Δ): $e\left(\Delta ,1-\Delta \right)=m^{\mathrm{ii}}\left(x\right)e^{\mathrm{ii}}\left(x\right)$ is performed, with $m^{\mathrm{ii}}\left(x\right)=2/\left(x+1\right)$. It should be emphasized that the ground-state energy density e(Δ,1−Δ) is not monotonic as a function of Δ. However, a re-parametrization operation in the ground-state energy density ensures that both $m^{\mathrm{ii}}\left(x\right)$ and $e^{\mathrm{ii}}\left(x\right)$ are monotonically decreasing with x. In particular, x has been chosen to be consistent with duality between regime III and regime IV, (cf. Appendix C). As discussed in Section 2, $S_{f}^{\mathrm{ii}}\left(x\right)$ includes contributions from fidelity entropy $S^{\mathrm{ii}}\left(x\right)$ and from scaling entropy $S_{\sigma }^{\mathrm{ii}}\left(x\right)$. That is, $S_{f}^{\mathrm{ii}}\left(x\right)=S^{\mathrm{ii}}\left(x\right)+S_{\sigma }^{\mathrm{ii}}\left(x\right)$, with scaling entropy $S_{\sigma }^{\mathrm{ii}}\left(x\right)=-\ln m^{\mathrm{ii}}\left(x\right)$. Here, $S^{\mathrm{ii}}\left(x\right)$ takes the following form:
  (A101) $$S^{\mathrm{ii}}\left(x\right)=-2\int_{x_{c}}^{x}\ln d^{\mathrm{ii}}\left(x;y\right)\;\mathrm{dy}+S_{0}^{\mathrm{ii}},$$
  where $d^{\mathrm{ii}}\left(x;y\right)$ denotes the ground-state fidelity per lattice site in principal part ii, and $S_{0}^{\mathrm{ii}}$ is the residual fidelity entropy at the critical point $x_{c}=0$.

We move to the two principal regimes: regime I and regime II.

(a) In regime I (0<Δ<1 and γ<1−Δ), we recall that a dominant control parameter x was chosen to be x=γ, and an auxiliary control parameter τ was chosen to be τ=Δ. From Equation (28), for a fixed τ, fidelity entropy $S^{I}\left(x,\tau \right)$ takes the following form

(A102) $$S^{I}\left(x,\tau \right)=-2\int_{x_{c}}^{x}\ln d^{I}\left(x,\tau ;y,\tau \right)\;dy+S_{0}^{I}\left(\tau \right).$$

Here, $d^{I}\left(x,\tau ;y,\tau \right)$ denotes the ground-state fidelity per lattice site in regime I, and $S_{0}^{I}\left(\tau \right)$ is the residual fidelity entropy at a critical point $x_{c}$ for a fixed τ, with $x_{c}=0$.

(b) In regime II (−1<Δ<0 and γ<1+Δ), we recall that a dominant control parameter x was chosen to be x=γ, and an auxiliary control parameter τ was chosen to be τ=Δ. From Equation (28), fidelity entropy $S^{\mathrm{II}}\left(x,\tau \right)$ takes the same form as Equation (A102) for regime I, with the label being changed from I to II.

Once fidelity entropy $S_{f}^{q}\left(x\right)$ (q=i and ii) in the two principal parts and fidelity entropy $S_{f}^{\omega }\left(x,\tau \right)$ (ω = I and II) in the two principal regimes are determined, fidelity temperature $T_{f}^{q}\left(x\right)$ and fidelity internal energy $U_{f}^{q}\left(x\right)$ in the principal parts may be determined from solving the singular first-order differential equation, Equation (14), and fidelity temperature $T_{f}^{\omega }\left(x,\tau \right)$ and fidelity internal energy $U_{f}^{\omega }\left(x,\tau \right)$ in the principal regimes may be determined from solving the singular first-order differential equation, Equation (31), as discussed for continuous QPTs in Section 2.

We start our discussion from the two principal parts, labelled as i and ii.

(i) On the factorizing-field line (γ=1+Δ with −1<Δ<0), for the chosen dominant control parameter x: x=Δ+1, fidelity temperature $T^{i}\left(x\right)$ vanishes: $T^{i}\left(x\right)=0$. Meanwhile, fidelity internal energy $U^{i}\left(x\right)$ is a constant: $U^{i}\left(x\right)=U_{m}$, where $U_{m}$ has been determined in Appendix K, since the quantum spin-1/2 XYZ model becomes the quantum spin-1/2 XY model, when x=1. As discussed in Section 2, we have $T_{f}^{i}\left(x\right)=T^{i}\left(x\right)$ and $U_{f}^{i}\left(x\right)=U^{i}\left(x\right)$.

(ii) On the dual line (γ=1−Δ with 0<Δ<1), for the chosen dominant control parameter x: x=1−Δ/(2−Δ), a re-parametrization operation in the ground-state energy density e(Δ,1−Δ): $e\left(\Delta ,1-\Delta \right)=m^{\mathrm{ii}}\left(x\right)e^{\mathrm{ii}}\left(x\right)$ with $m^{\mathrm{ii}}\left(x\right)=2/\left(1+x\right)$, is performed to ensure that $e^{\mathrm{ii}}\left(x\right)$ monotonically decreases with x. Then, from Equation (10), $U^{\mathrm{ii}}\left(x\right)$ takes the form

(A103) $$U^{\mathrm{ii}}\left(x\right)=\ln\frac{e^{\mathrm{ii}}\left(x\right)}{e^{\mathrm{ii}}\left(0\right)}V^{\mathrm{ii}}\left(x\right)+U_{0}^{\mathrm{ii}}.$$

Here, $U_{0}^{\mathrm{ii}}$ is an additive constant, and $V^{\mathrm{ii}}\left(x\right)>0$ satisfies the singular first-order differential equation

(A104) $$\partial V^{\mathrm{ii}}\left(x\right)/\partial x=\alpha^{\mathrm{ii}}\left(x\right)\;V^{\mathrm{ii}}\left(x\right),$$

with

(A105) $$\alpha^{\mathrm{ii}}\left(x\right)=-\frac{\partial \ln\left(e^{\mathrm{ii}}\left(x\right)/e^{\mathrm{ii}}\left(0\right)\right)/\partial x}{\partial S^{\mathrm{ii}}\left(x\right)/\partial x+\ln\left(e^{\mathrm{ii}}\left(x\right)/e^{\mathrm{ii}}\left(0\right)\right)}.$$

Accordingly, fidelity temperature $T^{\mathrm{ii}}\left(x\right)$ follows from

(A106) $$T^{\mathrm{ii}}\left(x\right)=-\frac{\partial V^{\mathrm{ii}}\left(x\right)}{\partial x}.$$

Next, we move to fidelity temperature and fidelity internal energy in the two principal regimes:

(a) In regime I (0<Δ<1 and 0<γ<1−Δ), for the chosen dominant control parameter x: x=γ, the ground-state energy density $e^{I}\left(x,\tau \right)$ monotonically decreases with x, for a fixed τ=Δ. Then, from Equation (30), fidelity internal energy $U^{I}\left(x,\tau \right)$ takes the form
  (A107) $$U^{I}\left(x,\tau \right)=\ln\frac{e^{I}\left(x,\tau \right)}{e^{I}\left(0,\tau \right)}V^{I}\left(x,\tau \right)+U_{0}^{I}\left(\tau \right).$$
  Here, $U_{0}^{I}\left(\tau \right)$ is a function of τ, and $V^{I}\left(x,\tau \right)>0$ satisfies the singular first-order differential equation
  (A108) $$\frac{\partial V^{I}\left(x,\tau \right)}{\partial x}=\alpha^{I}\left(x,\tau \right)\;V^{I}\left(x,\tau \right),$$
  with
  (A109) $$\alpha^{I}\left(x,\tau \right)=-\frac{\partial \ln\left(e^{I}\left(x,\tau \right)/e^{I}\left(0,\tau \right)\right)/\partial x}{\partial S^{I}\left(x,\tau \right)/\partial x+\ln\left(e^{I}\left(x,\tau \right)/e^{I}\left(0,\tau \right)\right)}.$$
  Accordingly, fidelity temperature $T^{I}\left(x,\tau \right)$ in this regime is given by
  (A110) $$T^{I}\left(x,\tau \right)=-\frac{\partial V^{I}\left(x,\tau \right)}{\partial x}.$$
(b) In regime II (−1<Δ<0 and 0<γ<1+Δ), fidelity internal energy $U^{\mathrm{II}}\left(x,\tau \right)$, fidelity temperature $T^{\mathrm{II}}\left(x,\tau \right)$, and $V^{\mathrm{II}}\left(x,\tau \right)$, together with its singular first-order differential equation with $\alpha^{\mathrm{II}}\left(x,\tau \right)$, take the same form as Equations (A107)–(A110) for regime I, with the label being changed from I to II.

To solve the singular first-order differential equation, Equation (A108), and its counterparts, we analyze the scaling behavior of $\alpha^{I/\mathrm{II}}\left(x,\tau \right)$ in the vicinity of a critical point $x_{c}$, with $x_{c}=0$, for −1<τ<1. As discussed in Appendix H, fidelity entropy $S^{I/\mathrm{II}}\left(x,\tau \right)$ scales as $S^{I/\mathrm{II}}\left(x,\tau \right)∼x^{\nu (\tau )+1}$, with ν(τ), as a function of τ∈(−1,1), being the critical exponent for the correlation length. In addition, for a fixed τ∈(−1,1), our numerical simulation shows that the ground-state energy density $e^{I/\mathrm{II}}\left(x,\tau \right)$ near a critical point $x_{c}$, with $x_{c}=0$, scales as follows

(A111) $$\ln\frac{e^{I/\mathrm{II}}\left(x,\tau \right)}{e^{I/\mathrm{II}}\left(0,\tau \right)}∼x^{K(\tau )}\;\ln x.$$

In regime I and regime II, as long as ν(τ)<K(τ)≤ν(τ)+1, $\alpha^{I/\mathrm{II}}\left(x,\tau \right)$ scales as follows

(A112) $$\alpha^{I/\mathrm{II}}\left(x,\tau \right)\propto x^{K(\tau )-\nu (\tau )-1}\ln x.$$

The scaling behaviors for regime I and regime II are the same, as anticipated from the fact that they both belong to the Gaussian universality class. This is confirmed numerically, as shown in Figure A11. Actually, two different sets of the critical exponent ν(τ) are plotted as a function of τ∈(−1,1): one is ν(τ), which is extracted from the scaling behavior of fidelity entropy $S^{I/\mathrm{II}}\left(x,\tau \right)$, the other is $\nu_{b}\left(\tau \right)$, extracted from the leading singular term via the exact solution in Ref. [125] (see, also Ref. [130]). For −1<τ<0, ν(τ) and $\nu_{b}\left(\tau \right)$ matches, with an accuracy up to 5%. However, for τ>0, a significant discrepancy arises between ν(τ) and $\nu_{b}\left(\tau \right)$. One might attribute this discrepancy to the fact that only the leading singular term is taken into account to extract $\nu_{b}\left(\tau \right)$, which also neglects the presence of logarithmic factor lnx. Indeed, the necessity to include this logarithmic factor may be justified from a heuristic argument that it exists, since $\alpha^{I/\mathrm{II}}\left(x,\tau \right)$ should be smooth along a line of critical points with τ∈(−1,1), combined with the fact that it exists at an infinite number of discrete points between τ=0 and τ=1 if π/μ is an even integer, with cosμ=τ [125]. Note that our numerical result for ν(τ), as τ approaches 1, coincides with a previous observation that ν(1)≈2 [229].

Since the integration of $\alpha^{I/\mathrm{II}}\left(x,\tau \right)$ with respect to x is finite, the singular first-order differential equation, Equation (A108), for regime I and its counterpart for regime II may be solved in a straightforward manner.

Let us first determine $V^{i}\left(x\right)$ and $V^{\mathrm{ii}}\left(x\right)$ in the two principal parts, labelled as i and ii, respectively.

(i) In principal part i on the factorizing-field line (γ=1+Δ with −1<Δ<0), $V^{i}\left(x\right)$ vanishes: $V^{i}\left(x\right)=0$.

(ii) In principal part ii on the U(1)-symmetric line (γ=1−Δ with 0<Δ<1), when the KT critical point $x_{c}=0$ is approached, fidelity entropy $S^{\mathrm{ii}}\left(x\right)$ scales as $S^{\mathrm{ii}}\left(x\right)∼x^{3}$. Taking into account the fact that the first-order derivative of $\ln\left(e^{\mathrm{ii}}\left(x\right)/e^{\mathrm{ii}}\left(0\right)\right)$ with respect to x at the critical point $x_{c}$, with $x_{c}=0$, is nonzero, $\alpha^{\mathrm{ii}}\left(x\right)$ scales as follows

(A113) $$\alpha^{\mathrm{ii}}\left(x\right)∼\frac{1}{x}.$$

This is a situation similar to the Kramers–Wannier dual line (γ=1) for the quantum spin-1/2 XY model. Then, since the integration of $\alpha^{\mathrm{ii}}\left(x\right)$ with respect to x is finite, the singular first-order differential equation, Equation (A104), may be solved as follows:

(A114) $$V^{\mathrm{ii}}\left(x\right)=V_{0}^{\mathrm{ii}}V_{1}^{\mathrm{ii}}\left(x\right),$$

where $V_{0}^{\mathrm{ii}}$ is a constant to be determined, and $V_{1}^{\mathrm{ii}}\left(x\right)$ takes the following form

(A115) $$V_{1}^{\mathrm{ii}}\left(x\right)=\exp\left(\int_{0}^{x}\alpha^{\mathrm{ii}}\left(y\right)dy\right).$$

The singular first-order differential equations for regime I and regime II are solved in a similar manner.

(a) In regime I (0<Δ<1 and 0<γ<1−Δ), since the integration of $\alpha^{I}\left(x,\tau \right)$ with respect to x for a fixed τ is finite, the singular first-order differential equation, Equation (A108), may be solved as follows:

(A116) $$V^{I}\left(x,\tau \right)=V_{0}^{I}\left(\tau \right)V_{1}^{I}\left(x,\tau \right),$$

where $V_{0}^{I}\left(\tau \right)$ is a function of τ, and $V_{1}^{I}\left(x,\tau \right)$ is defined as follows

(A117) $$V_{1}^{I}\left(x,\tau \right)=\exp\left(\int_{0}^{x}\alpha^{I}\left(y,\tau \right)dy\right).$$

(b) In regime II (−1<Δ<0 and 0<γ<1+Δ), the solution $V^{\mathrm{II}}\left(x\right)$ to the singular first-order differential equation takes the same form as Equations (A116) and (A117) for regime I, with the label being changed from I to II.

We require that fidelity entropy takes the same value $S_{m}$ as that at the U(1)-symmetric point (0,1) for the quantum spin-1/2 XY model (cf. Section 7). Therefore, $S_{0}^{i}$ and $S_{0}^{\mathrm{ii}}$ are determined: $S^{i}\left(1\right)=S_{m}$ and $S^{\mathrm{ii}}\left(1\right)=S_{m}$. Hence, fidelity entropy $S^{i}\left(x\right)$ and $S^{\mathrm{ii}}\left(x\right)$ in the two principal parts are determined.

Now, let us determine $V_{0}$ and $U_{0}$ in the two principal parts, labelled as i and ii, and in the two principal regimes: regime I and regime II. As argued in Section 2, we need to determine $T_{0}^{q}$ for principal part q (q= i and ii). In principal part i, we have $T_{0}^{i}=0$. In principal part ii, we have $T_{0}^{\mathrm{ii}}=T^{\mathrm{ii}}\left(1\right)$.

In order to ensure that fidelity temperature vanishes at the characteristic point (λ=0 and γ=1), $T^{\mathrm{ii}}\left(x\right)$ is shifted to $T^{\mathrm{ii}}\left(x\right)-T_{0}^{\mathrm{ii}}$, with $T_{0}^{\mathrm{ii}}\equiv T^{\mathrm{ii}}\left(1\right)$, accompanied by a shift in $U^{\mathrm{ii}}\left(x\right)$: $U^{\mathrm{ii}}\left(x\right)-T_{0}^{\mathrm{ii}}S^{\mathrm{ii}}\left(x\right)$. In addition, fidelity internal energy at the critical point $x_{c}$, with $x_{c}=0$, must be zero, and fidelity internal energy at the characteristic point x=1 (λ=0 and γ=1) satisfies the continuity requirement, as discussed in Section 2: $U_{0}^{\mathrm{ii}}=T_{0}^{\mathrm{ii}}S_{0}^{\mathrm{ii}}$ and $U^{\mathrm{ii}}\left(1\right)-T_{0}^{\mathrm{ii}}S^{\mathrm{ii}}\left(1\right)=U_{m}$. Therefore, $V_{0}^{\mathrm{ii}}$ is determined as follows

(A118) $$V_{0}^{\mathrm{ii}}=\frac{U_{m}}{\ln\left(e^{\mathrm{ii}}\left(1\right)/e^{\mathrm{ii}}\left(0\right)\right)V_{1}^{\mathrm{ii}}\left(1\right)+\alpha^{\mathrm{ii}}\left(1\right)V_{1}^{\mathrm{ii}}\left(1\right)\left(S^{\mathrm{ii}}\left(1\right)-S_{0}^{\mathrm{ii}}\right)}.$$

Once $V_{0}^{\mathrm{ii}}$ and $U_{0}^{\mathrm{ii}}$ are determined, fidelity temperature $T^{\mathrm{ii}}\left(x\right)$ and fidelity internal energy $U^{\mathrm{ii}}\left(x\right)$ follow from (A103) and (A106), respectively. Then, following from our discussions in Section 2, fidelity temperature $T_{f}^{\mathrm{ii}}\left(x\right)$ and fidelity internal energy $U_{f}^{\mathrm{ii}}\left(x\right)$ take the following form: $T_{f}^{\mathrm{ii}}\left(x\right)=T^{\mathrm{ii}}\left(x\right)-T_{0}^{\mathrm{ii}}$ and $U_{f}^{\mathrm{ii}}\left(x\right)=U^{\mathrm{ii}}\left(x\right)-T_{0}^{\mathrm{ii}}\;S^{\mathrm{ii}}\left(x\right)$, respectively.

Now we turn to the residual fidelity entropy on the line of the Gaussian critical points. We are able to determine the residual fidelity entropy $S_{0}^{I}\left(\tau \right)$ and $S_{0}^{\mathrm{II}}\left(\tau \right)$, respectively, from the continuity requirements: $S^{I}\left(1-\tau ,\tau \right)=S^{\mathrm{ii}}\left(1-\tau /\left(2-\tau \right)\right)$ at a point in principal part ii, and $S^{\mathrm{II}}\left(1+\tau ,\tau \right)=S^{i}\left(\tau +1\right)$ at a point in principal part i. Here, the continuity requirement for fidelity entropy at a point in the two principal parts, labelled as i and ii, implies that fidelity entropies in regime I and regime II include contributions from scaling entropy $S_{\sigma }^{I}\left(x,\tau \right)$ and $S_{\sigma }^{\mathrm{II}}\left(x,\tau \right)$ due to a re-parametrization operation in the ground-state energy density in the two principal parts, respectively. Hence, we have $S_{f}^{I}\left(x,\tau \right)=S^{I}\left(x,\tau \right)+$ $S_{\sigma }^{I}\left(x,\tau \right)$ and $S_{f}^{\mathrm{II}}\left(x,\tau \right)=S^{\mathrm{II}}\left(x,\tau \right)+S_{\sigma }^{\mathrm{II}}\left(x,\tau \right)$, where $S_{\sigma }^{I}\left(x,\tau \right)=S_{\sigma }^{\mathrm{ii}}\left(1-\tau /\left(2-\tau \right)\right)$ and $S_{\sigma }^{\mathrm{II}}\left(x,\tau \right)=S_{\sigma }^{i}\left(\tau +1\right)$.

We have to ensure that fidelity mechanical-state functions are *continuous* at the boundaries for the two principal regimes. Indeed, we have already taken into account the continuity requirement for fidelity entropy, thus determining the residual fidelity entropy on the line of the Gaussian critical points. The remaining task is to ensure the continuity requirements for fidelity temperature and fidelity internal energy. To this end, we need to determine $T_{0}^{I}\equiv T_{m}^{I}-T_{t}^{I}$ and $T_{0}^{\mathrm{II}}\equiv T_{m}^{\mathrm{II}}-T_{t}^{\mathrm{II}}$ for the two principal regimes. In regime I, we have $T_{m}^{I}\left(\tau \right)=T^{I}\left(1-\tau ,\tau \right)$ and $T_{t}^{I}\left(\tau \right)=T^{\mathrm{ii}}\left(1-\tau /\left(2-\tau \right)\right)$ for a fixed τ. In regime II, we have $T_{m}^{\mathrm{II}}\left(\tau \right)=T^{\mathrm{II}}\left(1+\tau ,\tau \right)$ and $T_{t}^{\mathrm{II}}\left(\tau \right)=0$ for a fixed τ.

In regime I, for a fixed τ, in order to ensure the continuity requirement for fidelity temperature in principal part ii, $T^{I}\left(x,\tau \right)$ is shifted to $T^{I}\left(x,\tau \right)-T_{0}^{I}\left(\tau \right)$, with $T_{0}^{I}\left(\tau \right)$$=T^{I}\left(\sqrt{1-\tau^{2}},\tau \right)$. Then, $U^{I}\left(x,\tau \right)$ is shifted to $U^{I}\left(x,\tau \right)-T_{0}^{I}\left(\tau \right)S^{I}\left(x,\tau \right)$, with $S^{I}\left(x,\tau \right)$ left intact. Here, $T_{0}^{I}\left(\tau \right)\equiv T^{I}\left(1-\tau ,\tau \right)-T^{\mathrm{ii}}\left(1-\tau /\left(2-\tau \right)\right)$. In addition, fidelity internal energy on the line of the Gaussian critical points (γ=0 with 0<Δ<1), with $x=x_{c}=0$, for a fixed τ must be zero, and fidelity internal energy satisfies the continuity requirement in principal part ii, as discussed in Section 2: $U_{0}^{I}\left(\tau \right)=T_{0}^{I}\left(\tau \right)S_{0}^{I}\left(\tau \right)$ and $U^{I}\left(1-\tau ,\tau \right)$$-T_{0}^{I}\left(\tau \right)S^{I}\left(1-\tau ,\tau \right)=U_{f}^{\mathrm{ii}}\left(1-\tau /\left(2-\tau \right)\right)$. Therefore, $V_{0}^{I}\left(\tau \right)$ is determined as follows

(A119) $$V_{0}^{I}\left(\tau \right)=\frac{U_{f}^{\mathrm{ii}}\left(1-\tau /\left(2-\tau \right)\right)-T_{f}^{\mathrm{ii}}\left(1-\tau /\left(2-\tau \right)\right)\left(S^{I}\left(1-\tau ,\tau \right)-S_{0}^{I}\left(\tau \right)\right)}{\ln\left(e^{I}\left(1-\tau ,\tau \right)/e^{I}\left(0,\tau \right)\right)V_{1}^{I}\left(1-\tau ,\tau \right)+\alpha^{I}\left(1-\tau ,\tau \right)V_{1}^{I}\left(1-\tau ,\tau \right)\left(S^{I}\left(1-\tau ,\tau \right)-S_{0}^{I}\left(\tau \right)\right)}.$$

Once $V_{0}^{I}\left(\tau \right)$ and $U_{0}^{I}\left(\tau \right)$ are determined, fidelity temperature $T^{I}\left(x,\tau \right)$ and fidelity internal energy $U^{I}\left(x,\tau \right)$ follow from (A107) and (A110), respectively. Following from our discussions in Section 2, fidelity entropy $S_{f}^{I}\left(x,\tau \right)$, fidelity temperature $T_{f}^{I}\left(x,\tau \right)$, and fidelity internal energy $U_{f}^{I}\left(x,\tau \right)$ take the following form: $S_{f}^{I}\left(x,\tau \right)=S^{I}\left(x,\tau \right)$, $T_{f}^{I}\left(x,\tau \right)=T^{I}\left(x,\tau \right)-T_{0}^{I}\left(\tau \right)$, and $U_{f}^{I}\left(x,\tau \right)=U^{I}\left(x,\tau \right)-T_{0}^{I}\left(\tau \right)S^{I}\left(x,\tau \right)$, respectively.

In regime II, for a fixed τ, in order to ensure the continuity requirement for fidelity temperature in principal part i, $T^{\mathrm{II}}\left(x,\tau \right)$ is shifted to $T^{\mathrm{II}}\left(x,\tau \right)-T_{0}^{\mathrm{II}}\left(\tau \right)$, with $T_{0}^{\mathrm{II}}\left(\tau \right)$$=T^{\mathrm{II}}\left(\sqrt{1-\tau^{2}},\tau \right)$. Then, $U^{\mathrm{II}}\left(x,\tau \right)$ is shifted to $U^{\mathrm{II}}\left(x,\tau \right)-T_{0}^{\mathrm{II}}\left(\tau \right)S^{\mathrm{II}}\left(x,\tau \right)$, with $S^{\mathrm{II}}\left(x,\tau \right)$ left intact. Here, $T_{0}^{\mathrm{II}}\left(\tau \right)=T^{\mathrm{II}}\left(1+\tau ,\tau \right)$. In addition, fidelity internal energy on the line of the Gaussian critical points (γ=0 with 0<Δ<1) or, equivalently, $x=x_{c}$, with $x_{c}=0$, for a fixed τ must be zero, and fidelity internal energy satisfies the continuity requirement in principal part i, as discussed in Section 2: $U_{0}^{\mathrm{II}}\left(\tau \right)=T_{0}^{\mathrm{II}}\left(\tau \right)S_{0}^{\mathrm{II}}\left(\tau \right)$ and $U^{\mathrm{II}}\left(1+\tau ,\tau \right)-T_{0}^{\mathrm{II}}\left(\tau \right)S^{\mathrm{II}}\left(1+\tau ,\tau \right)=U_{m}$. Therefore, $V_{0}^{\mathrm{II}}\left(\tau \right)$ is determined as follows

(A120) $$V_{0}^{\mathrm{II}}\left(\tau \right)=\frac{U_{m}}{\ln\left(e^{\mathrm{II}}\left(1+\tau ,\tau \right)/e^{\mathrm{II}}\left(0,\tau \right)\right)V_{1}^{\mathrm{II}}\left(1+\tau ,\tau \right)+\alpha^{\mathrm{II}}\left(1+\tau ,\tau \right)V_{1}^{\mathrm{II}}\left(1+\tau ,\tau \right)\left(S^{\mathrm{II}}\left(1+\tau ,\tau \right)-S_{0}^{\mathrm{II}}\left(\tau \right)\right)}.$$

After $V_{0}^{\mathrm{II}}\left(\tau \right)$ and $U_{0}^{\mathrm{II}}\left(\tau \right)$ are determined, fidelity temperature $T^{\mathrm{II}}\left(x,\tau \right)$ and fidelity internal energy $U^{\mathrm{II}}\left(x,\tau \right)$ follow from their counterparts of (A107) and (A110), with the label being changed from I to II, respectively. Following from our discussions in Section 2, fidelity entropy $S_{f}^{\mathrm{II}}\left(x,\tau \right)$, fidelity temperature $T_{f}^{\mathrm{II}}\left(x,\tau \right)$, and fidelity internal energy $U_{f}^{\mathrm{II}}\left(x,\tau \right)$ take the following form: $S_{f}^{\mathrm{II}}\left(x,\tau \right)=S^{\mathrm{II}}\left(x,\tau \right)$, $T_{f}^{\mathrm{II}}\left(x,\tau \right)=T^{\mathrm{II}}\left(x,\tau \right)-T_{0}^{\mathrm{II}}\left(\tau \right)$, and $U_{f}^{\mathrm{II}}\left(x,\tau \right)=U^{\mathrm{II}}\left(x,\tau \right)-T_{0}^{\mathrm{II}}\left(\tau \right)S^{\mathrm{II}}\left(x,\tau \right)$, respectively.

Once fidelity entropy $S_{f}^{q}\left(x\right)$, fidelity temperature $T_{f}^{q}\left(x\right)$, and fidelity internal energy $U_{f}^{q}\left(x\right)$ (q=i, ii) in the two principal parts and fidelity entropy $S_{f}^{\omega }\left(x,\tau \right)$, fidelity temperature $T_{f}^{\omega }\left(x,\tau \right)$, and fidelity internal energy $U_{f}^{\omega }\left(x,\tau \right)$ in the two principal regimes (ω = I, II) are determined, fidelity entropy $S_{f}\left(\Delta ,\gamma \right)$, fidelity temperature $T_{f}\left(\Delta ,\gamma \right)$, and fidelity internal energy $U_{f}\left(\Delta ,\gamma \right)$ follow. That is, $S_{f}^{q}\left(\Delta ,\gamma \right)\equiv S_{f}^{q}\left(x\right)$ and $S_{f}^{\omega }\left(\Delta ,\gamma \right)\equiv S_{f}^{\omega }\left(x,\tau \right)$, $T_{f}^{q}\left(\Delta ,\gamma \right)$$\equiv T_{f}^{q}\left(x\right)$ and $T_{f}^{\omega }\left(\Delta ,\gamma \right)\equiv T_{f}^{\omega }\left(x,\tau \right)$, and $U_{f}^{q}\left(\Delta ,\gamma \right)\equiv U_{f}^{q}\left(x\right)$ and $U_{f}^{\omega }\left(\Delta ,\gamma \right)\equiv U_{f}^{\omega }\left(x,\tau \right)$, when we move from x and τ to Δ and γ, meaning that x and τ are regarded as functions of Δ and γ. This is due to the fact that (Δ,γ) and (x,τ) label the same point in the control parameter space.

If fidelity entropy $S_{f}\left(\Delta ,\gamma \right)$, fidelity temperature $T_{f}\left(\Delta ,\gamma \right)$, and fidelity internal energy $U_{f}\left(\Delta ,\gamma \right)$ are determined in regime I and regime II, then fidelity mechanical-state functions in the non-principal regimes are determined from dualities in Appendix C by taking into account a contribution from scaling entropy arising from dualities (cf. Appendix G).

Numerical simulations for fidelity entropy $S_{f}\left(\Delta ,\gamma \right)$, fidelity temperature $T_{f}\left(\Delta ,\gamma \right)$, and fidelity internal energy $U_{f}\left(\Delta ,\gamma \right)$ are shown in Figure 18a–c, respectively.

### Appendix M.2. Fidelity Entropy $S_{f}\left(\Delta ,0\right)$, Fidelity Temperature $T_{f}\left(\Delta ,0\right)$, and Fidelity Internal Energy $U_{f}\left(\Delta ,0\right)$: An Interior Point of View

We start from the complete line of critical points (γ=0 with −1<Δ<1), which has been labelled as scenario III-1 in Section 6. Here, a dominant control parameter x is chosen to be x=1−Δ. From Equation (9), fidelity entropy S(x) takes the following form

(A121) $$S\left(x\right)=-2\int_{x_{c}}^{x}\ln d(x;y)\;dy+S_{0}.$$

Here, d(x;y) denotes the ground-state fidelity per lattice site on the complete line, labelled as scenario III-1, and $S_{0}$ is the residual fidelity entropy at the KT transition point $x_{c}=0$. According to our convention (cf. Section 2), we have $S_{f}\left(x\right)=S\left(x\right)$.

On the complete line (γ=0 with −1<Δ<1), which constitutes a principal part as a whole (cf. scenario III-1), ground-state energy density e(x) monotonically increases with x. Then, from Equation (10), fidelity internal energy U(x) takes the following form

(A122) $$U\left(x\right)=-\ln\frac{e(x)}{e(0)}V\left(x\right)+U_{0}.$$

Here, $U_{0}$ is an additive constant, and V(x)>0 satisfies the singular first-order differential equation:

(A123) $$\frac{\partial V(x)}{\partial x}=\alpha \left(x\right)\;V\left(x\right),$$

with

(A124) $$\alpha \left(x\right)=\frac{\partial \ln\left(e(x)/e(0)\right)/\partial x}{\partial S(x)/\partial x-\ln\left(e(x)/e(0)\right)}.$$

Accordingly, fidelity temperature T(x) on the complete line is given by the following

(A125) $$T\left(x\right)=-\frac{\partial V(x)}{\partial x}.$$

To solve the singular first-order differential equation, Equation (A123), we analyze the scaling behavior of α(x) in the vicinity of the KT transition point $x_{c}=0$. As it turns out, fidelity entropy S(x) scales as $S\left(x\right)∼x^{3}$. In addition, our numerical simulation shows that the ground-state energy density e(x) near the KT transition point $x_{c}=0$ scales as ln(e(x)/e(0))∼x. Then, α(x) diverges as follows

(A126) $$\alpha \left(x\right)\propto \frac{1}{x}.$$

Our numerical simulations confirm this scaling analysis.

Since the integration of α(x) with respect to x is finite, the singular first-order differential equation, Equation (A123), may be solved in a straightforward manner:

(A127) $$V\left(x\right)=V_{0}V_{1}\left(x\right),$$

where $V_{0}$ is a positive constant, and $V_{1}\left(x\right)$ takes the following form

(A128) $$V_{1}\left(x\right)=\exp\left(\int_{0}^{x}\alpha (y)dy\right).$$

The remaining task is to determine $S_{0}$, $U_{0}$, and $V_{0}$. Here, we require that, at the FM transition point x=2 or equivalently Δ=−1, fidelity mechanical-state functions are equal to those from an exterior point of view. In addition, at the KT transition point x=0 or equivalently, Δ=1, fidelity internal energy must be zero. Hence, $S_{0}$ is determined from the requirement $S_{f}\left(2\right)=S_{m}$, with $S_{m}$ denoting fidelity entropy from an exterior point of view at the FM transition point x=2. That is, fidelity entropy $S_{f}\left(x\right)$ from an interior point of view matches that from an exterior point of view at the FM transition point; thus, it is single-valued there. In addition, fidelity temperature at the FM transition point must be zero, thus leading to $T_{0}=T\left(2\right)$. Furthermore, fidelity internal energy $U_{f}\left(x\right)$ at the KT transition point x=0 must be zero, and fidelity internal energy $U_{f}\left(x\right)$ is single-valued at the FM transition point x=2 when it is approached along the U(1)-symmetric line (γ=0), as discussed in Section 2: $U_{0}=T_{0}S_{0}$ and $U\left(2\right)-T_{0}S\left(2\right)=U_{m}$, with $U_{m}$ denoting fidelity internal energy from an exterior point of view at the FM transition point. Hence, $V_{0}$ is determined as follows

(A129) $$V_{0}=\frac{U_{m}}{-\ln\left(e(2)/e(0)\right)V_{1}\left(2\right)+\alpha \left(2\right)V_{1}\left(2\right)\left(S_{f}\left(2\right)-S_{0}\right)}.$$

After $V_{0}$ and $U_{0}$ are determined, fidelity temperature T(x) and fidelity internal energy U(x) follow from (A122) and (A125), respectively. We refer to $T\left(x\right)-T_{0}$ and $U\left(x\right)-T_{0}S\left(x\right)$ as $T_{f}\left(x\right)$ and $U_{f}\left(x\right)$, respectively. That is, $T_{f}\left(x\right)\equiv T\left(x\right)-T_{0}$ and $U_{f}\left(x\right)\equiv U\left(x\right)-T_{0}S\left(x\right)$.

Once fidelity entropy $S_{f}\left(x\right)$, fidelity temperature $T_{f}\left(x\right)$, and fidelity internal energy $U_{f}\left(x\right)$ are determined on the complete line of critical points (γ=0 with −1<Δ<1), fidelity entropy $S_{f}\left(\Delta ,0\right)$, fidelity temperature $T_{f}\left(\Delta ,0\right)$, and fidelity internal energy $U_{f}\left(\Delta ,0\right)$ follow. That is, $S_{f}\left(\Delta ,0\right)\equiv S_{f}\left(x\right)$, $T_{f}\left(\Delta ,0\right)\equiv T_{f}\left(x\right)$, and $U_{f}\left(\Delta ,0\right)\equiv U_{f}\left(x\right)$, when we move from x to Δ, meaning that x is regarded as a function of Δ. This is due to the fact that (Δ,0) (−1<Δ<1) and x label the same point on the complete line.

Numerical simulation results for fidelity entropy $S_{f}\left(\Delta ,0\right)$, fidelity temperature $T_{f}\left(\Delta ,0\right)$, and fidelity internal energy $U_{f}\left(\Delta ,0\right)$ are shown in Figure 19a–c, respectively.

## Appendix N. Fidelity Entropy, Fidelity Temperature, and Fidelity Internal Energy for the Quantum Spin-1/2 XXZ Model in a Magnetic Field

In this Appendix, we present mathematical details about fidelity entropy $S_{f}\left(\Delta ,h\right)$, fidelity temperature $T_{f}\left(\Delta ,h\right)$, and fidelity internal energy $U_{f}\left(\Delta ,h\right)$ for the quantum spin-1/2 XXZ model in a magnetic field.

As shown in Figure 20, there are four phases, labelled as AF, ${\mathrm{FM}}_{-}$, ${\mathrm{FM}}_{+}$, and XY, representing an AF phase, an FM phase with all spin down, an FM phase with all spin up, and a critical phase with central charge c=1, respectively. We may restrict ourselves to the region h≥0, since the Hamiltonian (4) is symmetrical with respect to h↔−h. Meanwhile, the consideration of the phase transition lines and characteristic lines allows us to separate the entire region with h≥0 into four principal regimes: regime I, regime II, regime III, and regime IV.

Now we turn to the explicit mathematical expressions for fidelity entropy $S_{f}\left(\Delta ,h\right)$, fidelity temperature $T_{f}\left(\Delta ,h\right)$, and fidelity internal energy $U_{f}\left(\Delta ,h\right)$ for the quantum spin-1/2 XXZ model in a magnetic field.

The choices of a dominant control parameter x in the four principal regimes are as follows: In regime I, a dominant control parameter x was chosen to be $x=\sqrt{{\left(\Delta +1\right)}^{2}+h^{2}}/$$\left(1+\sqrt{{\left(\Delta +1\right)}^{2}+h^{2}}\right)$, and an auxiliary control parameter τ was chosen to be τ=arctan(h/(Δ+1))∈(π/2,π]. Here, a re-parametrization operation in the ground-state energy density e(Δ,h): $e\left(\Delta ,h\right)=m^{I}\left(x,\tau \right)\;e^{I}\left(x,\tau \right)$, with $m^{I}\left(x,\tau \right)=\left(2\sin\tau -\cos\tau \right)x/\left(1-x\right)+1$, is performed to ensure that $e^{I}\left(x,\tau \right)$ is a constant: $e^{I}\left(x,\tau \right)=-1$. In regime II, a dominant control parameter x was chosen to be x=1−1/(h−Δ), and an auxiliary control parameter τ was chosen to be τ=Δ∈[−1,∞). Here, a re-parametrization operation in the ground-state energy density e(Δ,h): $e\left(\Delta ,h\right)=m^{\mathrm{II}}\left(x,\tau \right)\;e^{\mathrm{II}}\left(x,\tau \right)$, with $m^{\mathrm{II}}\left(x,\tau \right)=\tau +2/\left(1-x\right)$, is performed to ensure that $e^{\mathrm{II}}\left(x,\tau \right)$ is a constant: $e^{\mathrm{II}}\left(x,\tau \right)=-1$. In regime III, a dominant control parameter x was chosen to be x=−Δ, and an auxiliary control parameter τ was chosen to be τ=h. In regime IV, a dominant control parameter x was chosen to be $x=h_{c}-h$, and an auxiliary control parameter τ was chosen to be τ=Δ.

We remark that fidelity mechanical-state functions on the line of the PT transition points (h=1+Δ with Δ>−1) for the quantum spin-1/2 XXZ model in a magnetic field (4) may be determined from those on the factorizing-field line (γ=1+Δ with Δ>−1) for the quantum spin-1/2 XYZ model (3), since we have to regard both of them as a sub-model of the quantum spin-1/2 XYZ model in a magnetic field. That is, the quantum spin-1/2 XXZ model in a magnetic field (4) consitutes a U(1)-symmetric plane (γ=0) and the quantum spin-1/2 XYZ model (3) constitutes a dual plane (h=0) for the quantum spin-1/2 XYZ model in a magnetic field.

In fact, the line of the PT transition points (h=1+Δ with Δ>−1) and the factorizing-field line (γ=1+Δ with Δ>−1) are located on the factorizing-field cone surface [77,78,79,80,81,82] (cf. scenario II-2 and scenario III-2 in Section 6). The presence of the factorizing-field cone surface divides the three-dimensional region, defined by h>0 and γ>0, into three-dimensional principal regimes in the control parameter space. In a three-dimensional principal regime, the three coupling parameters Δ, γ and h may be parametrized in the following form: γ=rsinθ and h=rcosθ, with Δ left intact. Hence, if we restrict to the factorizing-field cone surface, then a dominant control parameter x is chosen to be θ and two auxiliary control parameters $\tau_{1}$ and $\tau_{2}$ are chosen to be $\tau_{1}=\Delta$ and $\tau_{2}=1+\Delta$, respectively. In addition, an important observation is that the ground state remains to be the same for a fixed θ on the factorizing-field cone surface, implying that any circle on the factorizing-field cone surface with a fixed Δ accommodates the same set of factorized ground states as that on the disordered circle for the spin-1/2 XY model (1), with an identification of its coupling parameter λ with the coupling parameter h for the quantum spin-1/2 XYZ model in a magnetic field. In fact, the spin-1/2 XY model (1) is also a sub-model of the quantum spin-1/2 XYZ model in a magnetic field, with Δ=0. Given that the factorizing-field line (γ=1+Δ with Δ>−1) for the quantum spin-1/2 XYZ model (3) is divided into two parts that are dual to each other (cf. Appendix M), we need to partition the factorizing-field cone surface with Δ>−1 into two regimes: −1<Δ<0 and Δ>0. In regime −1<Δ<0, a re-parametrization operation is performed: $e\left(\gamma ,\Delta ,h\right)=m\left(x,\tau_{1},1+\tau_{1}\right)e\left(x,\tau_{1},1+\tau_{1}\right)$, with $m\left(x,\tau_{1},1+\tau_{1}\right)=\left(2+\tau_{1}\right)/2$. This results in a contribution to scaling entropy from the re-parametrization operation: $S_{\sigma }\left(\Delta ,\gamma ,h\right)=\ln\left(2+\Delta \right)-\ln2$. In regime Δ>0, one has to combine the re-parametrization operation with the duality transformation connecting the two parts on the factorizing-field line (γ=1+Δ with Δ>−1), both of which induce a contribution to scaling entropy: $S_{\sigma }\left(\Delta ,\gamma ,h\right)=\ln\left(2+\Delta \right)-2\ln\left(1+\Delta \right)-\ln2$ for Δ>0.

Hence, for the quantum spin-1/2 XXZ model in a magnetic field (4), fidelity temperature $T^{p}\left(\Delta \right)$ on the line of the PT transition points (h=1+Δ with Δ>−1) is zero, and fidelity internal energy $U^{p}\left(\Delta \right)$ on the line of the PT transition points (h=1+Δ with Δ>−1) takes the maximum value $U_{m}$, which is identical to that at all stable fixed points for the quantum spin-1/2 XYZ model (cf. Appendix M). Meanwhile, $S^{p}\left(\Delta \right)$ on the line of the PT transition points (h=1+Δ with Δ>−1) takes the following form:

(A130) $$S^{p}\left(\Delta \right)=S^{\mathrm{XYZ}}\left(\Delta ,1+\Delta \right)-S^{\mathrm{XY}}\left(0,1\right),$$

Here, $S^{\mathrm{XYZ}}\left(\Delta ,1+\Delta \right)$ is fidelity entropy on the factorizing-field line (γ=1+Δ with Δ>−1) for the quantum spin-1/2 XYZ model (3): $S^{\mathrm{XYZ}}\left(\Delta ,1+\Delta \right)=\ln\left(2+\Delta \right)-\ln2+S_{m}$ for −1<Δ<0 and $S^{\mathrm{XYZ}}\left(\Delta ,1+\Delta \right)=\ln\left(2+\Delta \right)-2\ln\left(1+\Delta \right)-\ln2+S_{m}$ for Δ>0, where $S_{m}$ denotes fidelity entropy at the U(1)-symmetric point (Δ=0 and γ=1) for the quantum spin-1/2 XYZ model (cf. Appendix M), and $S^{\mathrm{XY}}\left(0,1\right)$ denotes $S^{\mathrm{XY}}\left(\cos\theta ,\sin\theta \right)$ at θ=π/2, where $S^{\mathrm{XY}}\left(\cos\theta ,\sin\theta \right)$ is fidelity entropy at (cosθ, sinθ), evaluated from θ=0, on the disordered circle: $\lambda^{2}+\gamma^{2}=1$ for the quantum spin-1/2 XY model (1): $S^{\mathrm{XY}}\left(\cos\theta ,\sin\theta \right)\;=\;-2\int_{0}^{\theta }\ln d(\cos\theta ,\sin\theta ;\cos\eta ,\sin\eta )\;d\eta$, where d(cosθ,sinθ;cosη,sinη) is the ground-state fidelity per lattice site on the disordered circle (cf. Appendix A).

In regime I, the ground state remains the same: a spin-polarized state with all spins down for h>0. Therefore, $S_{f}^{I}\left(x,\tau \right)$ includes contributions from the residual fidelity entropy $S_{0}^{I}\left(\tau \right)$ and scaling entropy $S_{\sigma }^{I}\left(x,\tau \right)$, as discussed in Section 2: $S_{f}^{I}\left(x,\tau \right)=S_{0}^{I}\left(\tau \right)+S_{\sigma }^{I}\left(x,\tau \right)$, with scaling entropy $S_{\sigma }^{I}\left(x,\tau \right)=\ln\left(2\sin\tau x-\cos\tau x+1-x\right)-\ln\left(1-x\right)$. Here, $S_{0}^{I}\left(\tau \right)=S_{m}-\ln2$ is determined by the continuity requirement for fidelity entropy at the FM transition point: $S_{0}^{I}\left(\pi /2\right)=S^{p}\left(-1\right)$. In addition, fidelity temperature $T_{f}^{I}\left(x,\tau \right)$ is zero, and fidelity internal energy $U_{f}^{I}\left(x,\tau \right)$ is the maximum: $U_{f}^{I}\left(x,\tau \right)=U_{m}$.

In regime II, the ground state remains the same: a spin-polarized state with all spins down for h>0. Therefore, $S_{f}^{\mathrm{II}}\left(x,\tau \right)$ includes contributions from the residual fidelity entropy $S_{0}^{\mathrm{II}}\left(\tau \right)$ and scaling entropy $S_{\sigma }^{\mathrm{II}}\left(x,\tau \right)$, as discussed in Section 2: $S_{f}^{\mathrm{II}}\left(x,\tau \right)=S_{0}^{\mathrm{II}}\left(\tau \right)+S_{\sigma }^{\mathrm{II}}\left(x,\tau \right)$, with scaling entropy $S_{\sigma }^{\mathrm{II}}\left(x,\tau \right)=\ln\left(2+\tau -\tau x\right)-\ln\left(1-x\right)$. Here, $S_{0}^{\mathrm{II}}\left(\tau \right)$ is determined by the continuity requirement for fidelity entropy on the line of the PT transition points (h=1+Δ with Δ>−1): $S_{0}^{\mathrm{II}}\left(\tau \right)=S^{p}\left(\tau \right)$. In addition, fidelity temperature $T_{f}^{\mathrm{II}}\left(x,\tau \right)$ is zero, and fidelity internal energy $U_{f}^{\mathrm{II}}\left(x,\tau \right)$ is the maximum: $U_{f}^{\mathrm{II}}\left(x,\tau \right)=U_{m}$.

In regime III, from an exterior point of view, fidelity temperature $T_{f}^{\mathrm{III}}\left(x,\tau \right)$ diverges, indicating strong quantum fluctuations. Fidelity internal energy $U_{f}^{\mathrm{III}}\left(x,\tau \right)$ is, by convention, zero, and the only contribution to fidelity entropy is the residual fidelity entropy $S_{0}$, which is known on the line of the PT transition points. However, it is time-consuming to determine the residual fidelity entropy $S_{0}$ in regime III (cf. scenario II-2 and scenario III-2 in Section 6). Instead, we mainly focus on fidelity mechanical-state functions from an interior point of view in regime III.

In regime IV, for $\Delta =\Delta_{c}$, an IC transition occurs at $\left(\Delta_{c},h_{c}\right)$. The ground-state wave functions do not vary with *h* and the ground-state energy density does not depend on *h*. For the chosen control parameter *x*, the ground-state energy density e(x,τ) is a constant for a fixed τ. Therefore, fidelity entropy $S_{f}^{\mathrm{IV}}\left(x,\tau \right)$, fidelity temperature $T_{f}^{\mathrm{IV}}\left(x,\tau \right)$, and fidelity internal energy $U_{f}^{\mathrm{IV}}\left(x,\tau \right)$ do not depend on τ. That is, we only need to determine fidelity entropy, fidelity temperature, and fidelity internal energy on the $Z_{2}$-symmetric line (h=0 with Δ>1). We remark that fidelity mechanical-state functions for h=0 with Δ>1 are identical to those for the quantum spin-1/2 XYZ model, when γ=0 with Δ>1 (cf. Section 9). Therefore, we have $S_{f}^{\mathrm{IV}}\left(x,\tau \right)=S_{f}^{\mathrm{XYZ}}\left(\tau ,0\right)$, $S_{f}^{\mathrm{IV}}\left(x,\tau \right)=T_{f}^{\mathrm{XYZ}}\left(\tau ,0\right)$, and $S_{f}^{\mathrm{IV}}\left(x,\tau \right)=U_{f}^{\mathrm{XYZ}}\left(\tau ,0\right)$, respectively.

Now we move to fidelity entropy $S_{f}\left(x,\tau \right)$, fidelity temperature $T_{f}\left(x,\tau \right)$, and fidelity internal energy $U_{f}\left(x,\tau \right)$ in regime III from an interior point of view.

Principal regime III is enclosed by the boundaries consisting of a line of the Gaussian critical points, a line of the PT transition points, and a line of the IC transition points. Therefore, it is a two-dimensional critical XY regime. The critical points on a vertical line in regime III result from the level crossings. Therefore, a dominant control parameter *x* is, by definition, not available, since the ground-state fidelity per lattice site is zero. In contrast, the ground-state fidelity per lattice site is well-defined on a horizontal line in regime III. Therefore, a dominant control parameter *x* may be chosen. For the chosen dominant control parameter *x*, with x=−Δ, the ground-state energy density e(Δ,h) is not monotonic with Δ, for a fixed *h*. Therefore, a re-parametrization operation in the ground-state energy density e(Δ,h): $e\left(\Delta ,h\right)=m^{\mathrm{III}}\left(x,\tau \right)\;e^{\mathrm{III}}\left(x,\tau \right)$, with $m^{\mathrm{III}}\left(x,\tau \right)=1/\left(\Delta_{c}+x+1\right)$, is performed to ensure that $e^{\mathrm{III}}\left(x,\tau \right)$ is monotonic with *x*. As discussed in Section 2, $S_{f}^{\mathrm{III}}\left(x,\tau \right)$ includes contributions from both fidelity entropy $S^{\mathrm{III}}\left(x,\tau \right)$ and scaling entropy $S_{\sigma }^{\mathrm{III}}\left(x,\tau \right)$. That is, $S_{f}^{\mathrm{III}}\left(x,\tau \right)=S^{\mathrm{III}}\left(x,\tau \right)+S_{\sigma }^{\mathrm{III}}\left(x,\tau \right)$, with scaling entropy $S_{\sigma }^{\mathrm{III}}\left(x,\tau \right)=-\ln m^{\mathrm{III}}\left(x,\tau \right)$. For a fixed τ, $S^{\mathrm{III}}\left(x,\tau \right)$ takes the following form

(A131) $$S^{\mathrm{III}}\left(x,\tau \right)=-2\int_{x_{c}}^{x}\ln d^{\mathrm{III}}\left(x,\tau ;y,\tau \right)\;dy+S_{0}^{\mathrm{III}}\left(\tau \right).$$

Here, $d^{\mathrm{III}}\left(x,\tau ;y,\tau \right)$ denotes the ground-state fidelity per lattice site in regime III, and $S_{0}^{\mathrm{III}}\left(\tau \right)$ is the residual fidelity entropy at a critical point $x_{c}$ for a fixed τ.

In regime III, the ground-state energy density $e^{\mathrm{III}}\left(x,\tau \right)$ monotonically decreases with *x*. Then, from Equation (30), fidelity internal energy $U^{\mathrm{III}}\left(x,\tau \right)$ takes the following form

(A132) $$U^{\mathrm{III}}\left(x,\tau \right)=\ln\frac{e^{\mathrm{III}}\left(x,\tau \right)}{e^{\mathrm{III}}\left(0,\tau \right)}V^{\mathrm{III}}\left(x,\tau \right)+U_{0}^{\mathrm{III}}\left(\tau \right).$$

Here, $U_{0}^{\mathrm{III}}\left(\tau \right)$ is a function of τ, and $V^{\mathrm{III}}\left(x,\tau \right)>0$ satisfies the following differential equation:

(A133) $$\frac{\partial V^{\mathrm{III}}\left(x,\tau \right)}{\partial x}=\alpha^{\mathrm{III}}\left(x,\tau \right)\;V^{\mathrm{III}}\left(x,\tau \right),$$

with

(A134) $$\alpha^{\mathrm{III}}\left(x,\tau \right)=-\frac{\partial \ln\left(e^{\mathrm{III}}\left(x,\tau \right)/e^{\mathrm{III}}\left(0,\tau \right)\right)/\partial x}{\partial S^{\mathrm{III}}\left(x,\tau \right)/\partial x+\ln e^{\mathrm{III}}\left(x,\tau \right)/e^{\mathrm{III}}\left(0,\tau \right)}.$$

Accordingly, fidelity temperature $T^{\mathrm{III}}\left(x,\tau \right)$ in this regime is given by the following

(A135) $$T^{\mathrm{III}}\left(x,\tau \right)=-\frac{\partial V^{\mathrm{III}}\left(x,\tau \right)}{\partial x}.$$

To solve the singular first-order differential equation, Equation (A133), we analyze the scaling behavior of $\alpha^{\mathrm{III}}\left(x,\tau \right)$ in the vicinity of the IC transition point $x_{c}=0$. Fidelity entropy $S^{\mathrm{III}}\left(x,\tau \right)$ scales as $S^{\mathrm{III}}\left(x,\tau \right)∼x^{3}$. In addition, our numerical simulation shows that the ground-state energy density $e^{\mathrm{III}}\left(x,\tau \right)$ in the vicinity of the IC transition point $x_{c}=0$ scales as $\ln(e^{\mathrm{III}}\left(x,\tau \right)/e^{\mathrm{III}}\left(0,\tau \right))∼x$. Hence, $\alpha^{\mathrm{III}}\left(x,\tau \right)$ scales as follows

(A136) $$\alpha^{\mathrm{III}}\left(x,\tau \right)\propto \frac{1}{x}.$$

This scaling analysis is confirmed in our numerical simulation.

Since the integration of $\alpha^{\mathrm{III}}\left(x\right)$ with respect to *x* is finite, the singular first-order differential equation, Equation (A133), may be solved in a straightforward manner

(A137) $$V^{\mathrm{III}}\left(x,\tau \right)=V_{0}^{\mathrm{III}}\left(\tau \right)V_{1}^{\mathrm{III}}\left(x,\tau \right),$$

where $V_{0}^{\mathrm{III}}\left(\tau \right)$ is a function of τ, and $V_{1}^{\mathrm{III}}\left(x,\tau \right)$ is defined as follows

(A138) $$V_{1}^{\mathrm{III}}\left(x,\tau \right)=\exp\left(\int_{x_{c}}^{x}\alpha^{\mathrm{III}}\left(y,\tau \right)dy\right).$$

For a fixed τ, a PT transition point occurs at $x_{p}$, and an IC transition occurs at $x_{c}$. The continuity requirement for fidelity entropy at the PT transition point $x=x_{p}$ demands that $S^{\mathrm{III}}\left(x_{p},\tau \right)=S^{p}\left(\tau -1\right)$. Hence, $S_{0}^{\mathrm{III}}\left(\tau \right)$ is determined. In order to ensure the continuity requirement for fidelity temperature at the PT point $\left(x_{p},\;\tau \right)$, $T^{\mathrm{III}}\left(x,\tau \right)$ is shifted to $T^{\mathrm{III}}\left(x,\tau \right)-T_{0}^{\mathrm{III}}\left(\tau \right)$, accompanied by a shift in $U^{\mathrm{III}}\left(x,\tau \right)$: $U^{\mathrm{III}}\left(x,\tau \right)-T_{0}^{\mathrm{III}}\left(\tau \right)S^{\mathrm{III}}\left(x,\tau \right)$, with $S^{\mathrm{III}}\left(\Delta ,h\right)$ left intact. Here, $T_{0}^{\mathrm{III}}\left(\tau \right)\equiv T^{\mathrm{III}}\left(x_{p},\tau \right)$. In addition, fidelity internal energy at the IC transition point $\left(x_{c},\tau \right)$ must be zero, and fidelity internal energy satisfies the continuity requirement at the PT transition point $\left(x_{p},\;\tau \right)$, as discussed in Section 2: $U_{0}^{\mathrm{III}}\left(\tau \right)=T_{0}^{\mathrm{III}}\left(\tau \right)S_{0}^{\mathrm{III}}\left(\tau \right)$ and $U^{\mathrm{III}}\left(x_{p},\;\tau \right)-T_{0}^{\mathrm{III}}\left(\tau \right)S^{\mathrm{III}}\left(x_{p},\;\tau \right)=U_{m}$. Therefore, $V_{0}^{\mathrm{III}}\left(\tau \right)$ is determined as follows

(A139) $$V_{0}^{\mathrm{III}}\left(\tau \right)=\frac{U_{m}}{\ln\left(e^{\mathrm{III}}\left(x_{p},\;\tau \right)/e^{\mathrm{III}}\left(x_{c},\tau \right)\right)V^{\mathrm{III}}\left(x_{p},\;\tau \right)+\alpha^{\mathrm{III}}\left(x_{p},\;\tau \right)V^{\mathrm{III}}\left(x_{p},\;\tau \right)\left(S^{\mathrm{III}}\left(x_{p},\;\tau \right)-S_{0}^{\mathrm{III}}\left(\tau \right)\right)}.$$

Once $V_{0}^{\mathrm{III}}$ and $U_{0}^{\mathrm{III}}$ are determined, fidelity temperature $T^{\mathrm{III}}\left(x,\;\tau \right)$ and fidelity internal energy $U^{\mathrm{III}}\left(x,\;\tau \right)$ follow from (A132) and (A135), respectively. Following from our discussions in Section 2, fidelity entropy $S_{f}^{\mathrm{III}}\left(x,\tau \right)$, fidelity temperature $T_{f}^{\mathrm{III}}\left(x,\tau \right)$, and fidelity internal energy $U_{f}^{\mathrm{III}}\left(x,\tau \right)$ take the following form: $S_{f}^{\mathrm{III}}\left(x,\tau \right)=S^{\mathrm{III}}\left(x,\tau \right)$, $T_{f}^{\mathrm{III}}\left(x,\tau \right)=T^{\mathrm{III}}\left(x,\tau \right)-T_{0}^{\mathrm{III}}\left(\tau \right)$, and $U_{f}^{\mathrm{III}}\left(x,\tau \right)=U^{\mathrm{III}}\left(x,\tau \right)-T_{0}^{\mathrm{III}}\left(\tau \right)S^{\mathrm{III}}\left(x,\tau \right)$, respectively.

Once fidelity entropy $S_{f}^{\mathrm{III}}\left(x,\tau \right)$, fidelity temperature $T_{f}^{\mathrm{III}}\left(x,\tau \right)$, and fidelity internal energy $U_{f}^{\mathrm{III}}\left(x,\tau \right)$ are determined in this principal regime, fidelity entropy $S_{f}^{\mathrm{III}}\left(\Delta ,h\right)$, fidelity temperature $T_{f}^{\mathrm{III}}\left(\Delta ,h\right)$ and fidelity internal energy $U_{f}^{\mathrm{III}}\left(\Delta ,h\right)$ follow. That is, $S_{f}^{\mathrm{III}}\left(\Delta ,h\right)$$\equiv S_{f}^{\mathrm{III}}\left(x,\tau \right)$, $T_{f}^{\mathrm{III}}\left(\Delta ,h\right)\equiv T_{f}^{\mathrm{III}}\left(x,\tau \right)$, and $U_{f}^{\mathrm{III}}\left(\Delta ,h\right)\equiv U_{f}^{\mathrm{III}}\left(x,\tau \right)$, when we move from (x,τ) to (Δ,h), meaning that *x* and τ are regarded as functions of Δ and *h*. This is due to the fact that (Δ,h) and (x,τ) label the same point in the control parameter space.

Numerical simulations for fidelity entropy $S_{f}\left(\Delta ,h\right)$, fidelity temperature $T_{f}\left(\Delta ,h\right)$, and fidelity internal energy $U_{f}\left(\Delta ,h\right)$ are shown as a function of Δ in Figure 21a–c, respectively, for h=0.25, 0.45, and 1.

## Appendix O. Fidelity Entropy $S_{f}\left(\Delta ,\gamma \right)$, Fidelity Temperature $T_{f}\left(\Delta ,\gamma \right)$, and Fidelity Internal Energy $U_{f}\left(\Delta ,\gamma \right)$ for the Quantum Spin-1 XYZ Model

In this Appendix, we present mathematical details about fidelity entropy $S_{f}\left(\Delta ,\gamma \right)$, fidelity temperature $T_{f}\left(\Delta ,\gamma \right)$ and fidelity internal energy $U_{f}\left(\Delta ,\gamma \right)$ for the quantum spin-1 XYZ model.

We restrict ourselves to the region γ≥0 due to the fact that the Hamiltonian (5) is symmetrical with respect to γ↔−γ. The ground-state phase diagram is shown in Figure 17 with four distinct phases, labelled as ${\mathrm{AF}}_{x}$, ${\mathrm{AF}}_{z}$, ${\mathrm{FM}}_{z}$, and the Haldane phase—a typical example for the SPT phases. Our simulation results from the iTEBD algorithm, with the bond dimension χ=60, yield the Ising transition point from the ${\mathrm{AF}}_{x}$ phase to the Haldane phase on the U(1)-symmetric line (γ=1−Δ with 0<Δ<1) is located at $\left(\Delta_{c0},1-\Delta_{c0}\right)$, with $\Delta_{c0}\approx 0.915$, and the KT transition point from the critical XY phase to the Haldane phase on the U(1)-symmetric line (γ=0) is located at $\left(\Delta_{c1},0\right)$, with $\Delta_{c1}\approx 0.29$. The three lines of the Gaussian critical points are located on γ=0 with $-1<\Delta \leq \Delta_{c1}$ and its two dual images. In addition, there exist six lines of the Ising critical points, dual to each other, with one of them connecting $\left(\Delta_{c1},0\right)$ and $\left(\Delta_{c0},1-\Delta_{c0}\right)$. Moreover, the FM transition points are located at (−1,0) and its dual image points, and the SU(2)-symmetric points are located at (1,0) and its dual image points.

As demonstrated in Appendix C, there are five different dualities, which are identical to those for the quantum spin-1/2 XYZ chain, thus leading to four self-dual lines (γ=±1±Δ), and two semi-self-dual lines (γ=1 and Δ=0). The factorizing-field line is located at γ=1+Δ, with Δ>−1 [77,78,79].

Taking into account the symmetries, dualities, and factorizing fields, together with one soft line and the phase boundaries between different phases, we may divide the region γ≥0 into twenty-four different regimes. The twenty-four regimes fall into four groups, with six regimes in each group that are dual to each other. As shown in Section 2, regime I, regime V, regime IX, regime XIII, regime XVII, and regime XXI are dual to each other; regime II, regime VI, regime X, regime XIV, regime XVIII, and regime XXII are dual to each other; regime III, regime VII, regime XI, regime XV, regime XIX, and regime XXIII are dual to each other, whereas regime IV, regime VIII, regime XII, regime XVI, regime XX, and regime XXIV are dual to each other. Therefore, there are only four principal regimes, representing the physics underlying the model. We chose regime I ($0<\Delta <\Delta_{c0}$ and 0<γ<1), regime II (−1<Δ<0 and 0<γ<1+Δ), regime III ($\Delta_{c1}<\Delta <1$ and $0<\gamma <1-\Delta_{c0}$), and regime IV ($0<\Delta <\Delta_{c1}$ and $0<\gamma <1-\Delta_{c1}$) as four principal regimes.

As it turns out, the SU(2)-symmetric point (1,0) is a metastable fixed point, which is located on the SU(2) characteristic line—the spin-1 bilinear–biquadratic model—if we embed the Hamiltonian (5) into a more general model including an anisotropic extension of the biquadratic interactions. Fidelity internal energy and fidelity temperature at the metastable fixed point (1,0) follow from the continuity requirements by taking into account the fact that they are determined from the Takhtajan–Babujian critical point to the AKLT point on the SU(2) characteristic line. That is, the AKLT point is regarded as the stable fixed point. Therefore, we demand that fidelity temperature is zero and fidelity internal energy is equal to the maximum $U_{m}$ at the AKLT point. In this Appendix, fidelity mechanical-state functions at the metastable fixed point (1,0) is denoted as $S_{H}$, $T_{H}$, and $U_{H}$, as discussed in Appendix O.3.

Now we turn to the explicit mathematical expressions for fidelity entropy $S_{f}\left(\Delta ,\gamma \right)$, fidelity temperature $T_{f}\left(\Delta ,\gamma \right)$, and fidelity internal energy $U_{f}\left(\Delta ,\gamma \right)$.

### Appendix O.1. Fidelity Entropy $S_{f}\left(\Delta ,\gamma \right)$, Fidelity Temperature $T_{f}\left(\Delta ,\gamma \right)$, and Fidelity Internal Energy $U_{f}\left(\Delta ,\gamma \right)$: An Exterior Point of View

There are five principal parts on the characteristic lines: (i) the U(1)-symmetric line (γ=0 with $\Delta_{c1}<\Delta <1$), which is also a dual line; (ii) the U(1)-symmetric line (γ=1−Δ with $0<\Delta <\Delta_{c0}$), which is also a dual line; (iii) the U(1)-symmetric line (γ=1−Δ with $\Delta_{c0}<\Delta <1$), which is also a dual line; (iv) the factorizing-field line (γ=1+Δ with −1<Δ<0); (v) the semi-self-dual line (Δ=0 with 0<γ<1).

Let us determine fidelity entropy S(Δ,γ) in the five principal parts.

(i) In principal part i on the U(1)-symmetric line (γ=0 with $\Delta_{c1}<\Delta <1$), we recall that a dominant control parameter x was chosen to be $x=\Delta -\Delta_{c1}$. As follows from definition (9), fidelity entropy $S^{i}\left(x\right)$ takes the following form

(A140) $$S^{i}\left(x\right)=-2\int_{x_{c}}^{x}\ln d^{i}\left(x;y\right)\;dy+S_{0}^{i}.$$

Here, $d^{i}\left(x;y\right)$ denotes the ground-state fidelity per lattice site in principal part i, and $S_{0}^{i}$ is the residual fidelity entropy at the KT transition point $x_{c}=0$. According to our convention (cf. Section 2), we have $S_{f}^{i}\left(x\right)=S^{i}\left(x\right)$.

(ii) In principal part ii on the U(1)-symmetric line (γ=1−Δ with $0<\Delta <\Delta_{c0}$), we recall that a dominant control parameter x was chosen to be $x=\Delta_{c0}/\left(2-\Delta_{c0}\right)-\Delta /\left(2-\Delta \right)$. The Ising transition occurs at $x_{c}=0$ or equivalently $\left(\Delta_{c0},1-\Delta_{c0}\right)$. Here, a re-parametrization operation in the ground-state energy density e(Δ,1−Δ): $e\left(\Delta ,1-\Delta \right)=m^{\mathrm{ii}}\left(x\right)e^{\mathrm{ii}}\left(x\right)$, with $m^{\mathrm{ii}}\left(x\right)=\left(\Delta_{c0}/\left(2-\Delta_{c0}\right)+1\right)/\left(x+1\right)$, is performed. It should be emphasized that the ground-state energy density e(Δ,1−Δ) is not monotonic as a function of Δ on the U(1)-symmetric line (γ=1−Δ). In contrast, both $m^{\mathrm{ii}}\left(x\right)$ and $e^{\mathrm{ii}}\left(x\right)$ monotonically decreases with x. In particular, x has been chosen to retain consistency with duality between regime V and regime IX (cf. Appendix C). As discussed in Section 2, $S_{f}^{\mathrm{ii}}\left(x\right)$ includes contributions from both fidelity entropy $S^{\mathrm{ii}}\left(x\right)$ and scaling entropy $S_{\sigma }^{\mathrm{ii}}\left(x\right)$. That is, $S_{f}^{\mathrm{ii}}\left(x\right)=S^{\mathrm{ii}}\left(x\right)+S_{\sigma }^{\mathrm{ii}}\left(x\right)$, with scaling entropy $S_{\sigma }^{\mathrm{ii}}\left(x\right)=-\ln m^{\mathrm{ii}}\left(x\right)$. From Equation (9), fidelity entropy $S^{\mathrm{ii}}\left(x\right)$ takes the same form as Equation (A140) for part i, with the label being changed from i to ii.

(iii) In principal part iii on the U(1)-symmetric line (γ=1−Δ with $\Delta_{c0}<\Delta <1$), we recall that a dominant control parameter x was chosen to be $x=\Delta /\left(2-\Delta \right)-\Delta_{c0}/\left(2-\Delta_{c0}\right)$. The Ising transition occurs at $x_{c}=0$ or equivalently, $\left(\Delta_{c0},1-\Delta_{c0}\right)$. It should be emphasized that the ground-state energy density e(Δ,1−Δ) is monotonically decreasing with x, along the U(1)-symmetric line (γ=1−Δ with $\Delta_{c0}<\Delta <1$). Fidelity entropy $S^{\mathrm{iii}}\left(x\right)$, as follows from Equation (9), takes the same form as Equation (A140) for part i, with the label being changed from i to iii. According to our convention (cf. Section 2), we have $S_{f}^{\mathrm{iii}}\left(x\right)=S^{\mathrm{iii}}\left(x\right)$.

(iv) In principal part iv on the factorizing-field line (γ=1+Δ with 0<γ<1), the same factorized state occurs as ground-state wave functions, with the ground-state energy density e(Δ,1+Δ) being e(Δ,1+Δ)=−(Δ+2)/2. We recall that a dominant control parameter x was chosen to be x=Δ+1 for a fixed γ−Δ. Here, a re-parametrization operation in the ground-state energy density e(Δ,1+Δ): $e\left(\Delta ,1+\Delta \right)=m^{\mathrm{iv}}\left(x\right)e^{\mathrm{iv}}\left(x\right)$, with $m^{\mathrm{iv}}\left(x\right)=\left(x+1\right)/2$, is performed. Therefore, $e^{\mathrm{iv}}\left(x\right)$ is a constant as x varies. With this in mind, fidelity entropy $S^{\mathrm{iv}}\left(x\right)$ in principal part iv is identical to $S^{\mathrm{iv}}\left(1\right)$, apart from scaling entropy $S_{\sigma }^{\mathrm{iv}}\left(x\right)$. Thus, we have $S_{f}^{\mathrm{iv}}\left(x\right)=S^{\mathrm{iv}}\left(1\right)+S_{\sigma }^{\mathrm{iv}}\left(x\right)$, with $S_{\sigma }^{\mathrm{iv}}\left(x\right)=\ln m^{\mathrm{ii}}\left(x\right)$.

(v) In principal part v on the semi-self-dual line (Δ=0 with 0<γ<1), we recall that a dominant control parameter x was chosen to be x=γ. From Equation (9), fidelity entropy $S^{v}\left(x\right)$ takes the same form as Equation (A140) for part i, with the label being changed from i to v. According to our convention (cf. Section 2), we have $S_{f}^{v}\left(x\right)=S^{v}\left(x\right)$.

We move to the four principal regimes: regime I, regime II, regime III, and regime IV.

(a) In regime I ($0<\Delta <\Delta_{c0}$ and 0<γ<1), we recall that a dominant control parameter x was chosen to be $x=\Delta_{c}/\left(2-\Delta_{c}\right)-\Delta /\left(2-\Delta \right)$ and an auxiliary control parameter τ was chosen to be τ=γ+Δ. The Ising transition occurs at $x_{c}=0$ or equivalently, $\left(\Delta_{c},\gamma_{c}\right)$. Here, a re-parametrization operation in the ground-state energy density e(Δ,γ): $e\left(\Delta ,\gamma \right)=m^{I}\left(x,\tau \right)e^{I}\left(x,\tau \right)$, with $m^{I}\left(x,\tau \right)=\left(\Delta_{c}/\left(2-\Delta_{c}\right)+1\right)/\left(x+1\right)$, is performed. It should be emphasized that the ground-state energy density e(Δ,γ) is not monotonic as a function of Δ on the line (γ=τ−Δ). In contrast, both $m^{I}\left(x,\tau \right)$ and $e^{I}\left(x,\tau \right)$ monotonically decreased with x for a fixed τ. As discussed in Section 2, $S_{f}^{I}\left(x,\tau \right)$ includes contributions from both fidelity entropy $S^{I}\left(x,\tau \right)$ and scaling entropy $S_{\sigma }^{I}\left(x,\tau \right)$. That is, $S_{f}^{I}\left(x,\tau \right)=S^{I}\left(x,\tau \right)+S_{\sigma }^{I}\left(x,\tau \right)$, where scaling entropy $S_{\sigma }^{I}\left(x,\tau \right)=-\ln m^{I}\left(x,\tau \right)$, and for a fixed τ, $S^{I}\left(x,\tau \right)$ takes the following form

(A141) $$S^{I}\left(x,\tau \right)=-2\int_{x_{c}}^{x}\ln d^{I}\left(x,\tau ;y,\tau \right)\;dy+S_{0}^{I}\left(\tau \right).$$

Here, $d^{I}\left(x,\tau ;y,\tau \right)$ denotes the ground-state fidelity per lattice site in regime I, and $S_{0}^{I}\left(\tau \right)$ is the residual fidelity entropy at a critical point $x_{c}$ for a fixed τ, with $x_{c}=0$.

(b) In regime II (−1<Δ<0 and γ<1+Δ), we recall that a dominant control parameter x was chosen to be x=γ and an auxiliary control parameter τ was chosen to be τ=λ. Following from Equation (28), fidelity entropy $S^{\mathrm{II}}\left(x,\tau \right)$ takes the same form as Equation (A141) for regime I, with the label being changed from I to II. According to our convention (cf. Section 2), we have $S_{f}^{\mathrm{II}}\left(x,\tau \right)=S^{\mathrm{II}}\left(x,\tau \right)$.

(c) In regime III ($\Delta_{c1}<\Delta <1$ and $0<\gamma <1-\Delta_{c0}$), we recall that a dominant control parameter x was chosen to be $x=\Delta /\left(2-\Delta \right)-\Delta_{c}/\left(2-\Delta_{c}\right)$ and an auxiliary control parameter τ was chosen to be τ=γ+Δ. The Ising transition occurs at $x_{c}=0$ or equivalently $\left(\Delta_{c},\gamma_{c}\right)$. Following from Equation (28), fidelity entropy $S^{\mathrm{III}}\left(x,\tau \right)$ takes the same form as Equation (A141) for regime I, with the label being changed from I to III. According to our convention (cf. Section 2), we have $S_{f}^{\mathrm{III}}\left(x,\tau \right)=S^{\mathrm{III}}\left(x,\tau \right)$.

(d) In regime IV ($0<\Delta <\Delta_{c1}$ and $0<\gamma <\Delta_{c1}$), we recall that a dominant control parameter x was chosen to be x=(γ+Δ)/(2−γ−Δ)−Δ/(2−Δ) and an auxiliary control parameter τ was chosen to be τ=γ+Δ. The Gaussian transition occurs at $x_{c}=0$ for a fixed τ or equivalently (τ,0). Here, a re-parametrization operation in the ground-state energy density e(Δ,γ): $e\left(\Delta ,\gamma \right)=m^{\mathrm{IV}}\left(x,\tau \right)e^{\mathrm{IV}}\left(x,\tau \right)$, with $m^{\mathrm{IV}}\left(x,\tau \right)=\left(\tau /\left(2-\tau \right)+1\right)/\left(x+1\right)$, is performed. It should be emphasized that the ground-state energy density e(Δ,γ) is not monotonic as a function of Δ on the line (γ=τ−Δ). In contrast, both $m^{\mathrm{IV}}\left(x,\tau \right)$ and $e^{\mathrm{IV}}\left(x,\tau \right)$ monotonically decrease with x for a fixed τ. As discussed in Section 2, $S_{f}^{\mathrm{IV}}\left(x,\tau \right)$ includes contributions from both fidelity entropy $S^{\mathrm{IV}}\left(x,\tau \right)$ and scaling entropy $S_{\sigma }^{\mathrm{IV}}\left(x,\tau \right)$. That is, $S_{f}^{\mathrm{IV}}\left(x,\tau \right)=S^{\mathrm{IV}}\left(x,\tau \right)+S_{\sigma }^{\mathrm{IV}}\left(x,\tau \right)$, where scaling entropy $S_{\sigma }^{\mathrm{IV}}\left(x,\tau \right)=-\ln m^{\mathrm{IV}}\left(x,\tau \right)$, and fidelity entropy $S^{\mathrm{IV}}\left(x,\tau \right)$, as follows from Equation (28), takes the same form as Equation (A141) for regime I, with the label being changed from I to IV.

Once fidelity entropy $S_{f}^{q}\left(x\right)$ (q=i, ii, iii, iv, and v) in the five principal parts and fidelity entropy $S_{f}^{\omega }\left(x,\tau \right)$ (ω = I, II, III and IV) in the four principal regimes are determined, fidelity temperature $T_{f}^{q}\left(x\right)$ and fidelity internal energy $U_{f}^{q}\left(x\right)$ in the principal parts may be determined from solving the singular first-order differential equation, Equation (14), and fidelity temperature $T_{f}^{\omega }\left(x,\tau \right)$ and fidelity internal energy $U_{f}^{\omega }\left(x,\tau \right)$ in the principal regimes may be determined by solving the singular first-order differential equation, Equation (31), according to our prescription for continuous QPTs in Section 2.

Now we turn to fidelity temperature $T_{f}\left(x,\tau \right)$ and fidelity internal energy $U_{f}\left(x,\tau \right)$ in the five principal parts.

(i) In principal part i on the U(1)-symmetric line (γ=0 with $\Delta_{c1}<\Delta <1$), for the chosen dominant control parameter $x=\Delta -\Delta_{c1}$, the ground-state energy density $e^{i}\left(x\right)$ monotonically decreases with x. Then, from Equation (10), fidelity internal energy $U^{i}\left(x\right)$ takes the following form

(A142) $$U^{i}\left(x\right)=\ln\frac{e^{i}\left(x\right)}{e^{i}\left(0\right)}V^{i}\left(x\right)+U_{0}^{i}.$$

Here, $U_{0}^{i}$ is an additive constant, and $V^{i}\left(x\right)>0$ satisfies the singular first-order differential equation

(A143) $$\partial V^{i}\left(x\right)/\partial x=\alpha^{i}\left(x\right)\;V^{i}\left(x\right),$$

with

(A144) $$\alpha^{i}\left(x\right)=-\frac{\partial \ln\left(e^{i}\left(x\right)/e^{i}\left(0\right)\right)/\partial x}{\partial S^{i}\left(x\right)/\partial x+\ln\left(e^{i}\left(x\right)/e^{i}\left(0\right)\right)}.$$

Accordingly, fidelity temperature $T^{i}\left(x\right)$ follows from

(A145) $$T^{i}\left(x\right)=-\frac{\partial V^{i}\left(x\right)}{\partial x}.$$

(ii) In principal part ii on the U(1)-symmetric line (γ=1−Δ with $0<\Delta <\Delta_{c0}$), fidelity internal energy $U^{\mathrm{ii}}\left(x\right)$, fidelity temperature $T^{\mathrm{ii}}\left(x\right)$, and $V^{\mathrm{ii}}\left(x\right)$, together with its singular first-order differential equation with $\alpha^{\mathrm{ii}}\left(x\right)$, take the same form as Equations (A142)–(A145) for part i, with the label being changed from i to ii, respectively.

(iii) In principal part iii on the U(1)-symmetric line (γ=1−Δ with $\Delta_{c0}<\Delta <1$), fidelity internal energy $U^{\mathrm{iii}}\left(x\right)$, fidelity temperature $T^{\mathrm{iii}}\left(x\right)$, and $V^{\mathrm{iii}}\left(x\right)$, together with its singular first-order differential equation with $\alpha^{\mathrm{iii}}\left(x\right)$, take the same form as Equations (A142)–(A145) for part i, with the label being changed from i to iii, respectively.

(iv) In principal part iv on the factorizing-field line (γ=1+Δ with 0<γ<1), for the chosen dominant control parameter x=Δ+1, fidelity temperature $T^{\mathrm{iv}}\left(x\right)$ vanishes: $T^{\mathrm{iv}}\left(x\right)=0$. Meanwhile, fidelity internal energy $U^{\mathrm{iv}}\left(x\right)$ is a constant: $U^{\mathrm{iv}}\left(x\right)=U_{m}$, with $U_{m}$ being the maximum yet to be determined. As discussed in Section 2, we have $T_{f}^{\mathrm{iv}}\left(x\right)=T^{\mathrm{iv}}\left(x\right)$ and $U_{f}^{\mathrm{iv}}\left(x\right)=U^{\mathrm{iv}}\left(x\right)$.

(v) In principal part v, fidelity internal energy $U^{v}\left(x\right)$, fidelity temperature $T^{v}\left(x\right)$, and $V^{v}\left(x\right)$, together with its singular first-order differential equation with $\alpha^{v}\left(x\right)$, take the same form as Equations (A142)–(A145) for part i, with the label being changed from i to v.

We move to fidelity temperature and fidelity internal energy in four principal regimes: regime I, regime II, regime III, and regime IV.

(a) In regime I ($0<\Delta <\Delta_{c0}$ and 0<γ<1), for a fixed τ=γ+Δ, the Ising transition occurs at $\left(\Delta_{c},\gamma_{c}\right)$ or, equivalently, $\left(x_{c},\tau \right)$, with $x=\Delta_{c}/\left(2-\Delta_{c}\right)-\Delta /\left(2-\Delta \right)$. For a fixed τ, the ground-state energy density $e^{I}\left(x,\tau \right)$ monotonically decreases with x. Hence, from Equation (30), fidelity internal energy $U^{I}\left(x,\tau \right)$ takes the following form

(A146) $$U^{I}\left(x,\tau \right)=\ln\frac{e^{I}\left(x,\tau \right)}{e^{I}\left(0,\tau \right)}V^{I}\left(x,\tau \right)+U_{0}^{I}\left(\tau \right).$$

Here, $U_{0}^{I}\left(\tau \right)$ is a function of τ, and $V^{I}\left(x,\tau \right)>0$ satisfies the singular first-order differential equation:

(A147) $$\frac{\partial V^{I}\left(x,\tau \right)}{\partial x}=\alpha^{I}\left(x,\tau \right)\;V^{I}\left(x,\tau \right),$$

with

(A148) $$\alpha^{I}\left(x,\tau \right)=-\frac{\partial \ln\left(e^{I}\left(x,\tau \right)/e^{I}\left(0,\tau \right)\right)/\partial x}{\partial S^{I}\left(x,\tau \right)/\partial x+\ln\left(e^{I}\left(x,\tau \right)/e^{I}\left(0,\tau \right)\right)}.$$

Accordingly, fidelity temperature $T^{I}\left(x,\tau \right)$ in this regime is given by the following

(A149) $$T^{I}\left(x,\tau \right)=-\frac{\partial V^{I}\left(x,\tau \right)}{\partial x}.$$

(b) In regime II (−1<Δ<0 and 0<γ<1+Δ), fidelity internal energy $U^{\mathrm{II}}\left(x,\tau \right)$, fidelity temperature $T^{\mathrm{II}}\left(x,\tau \right)$, and $V^{\mathrm{II}}\left(x,\tau \right)$, together with its singular first-order differential equation with $\alpha^{\mathrm{II}}\left(x,\tau \right)$, take the same form as Equations (A146)–(A149) for regime I, with the label being changed from I to II, respectively.

(c) In regime III ($\Delta_{c1}<\Delta <1$ and $0<\gamma <1-\Delta_{c0}$), fidelity internal energy $U^{\mathrm{III}}\left(x,\tau \right)$, fidelity temperature $T^{\mathrm{III}}\left(x,\tau \right)$, and $V^{\mathrm{III}}\left(x,\tau \right)$, together with its singular first-order differential equation with $\alpha^{\mathrm{III}}\left(x,\tau \right)$, take the same form as Equations (A146)–(A149) for regime I, with the label being changed from I to III, respectively.

(d) In regime IV ($0<\Delta <\Delta_{c1}$ and $0<\gamma <1-\Delta_{c1}$), fidelity internal energy $U^{\mathrm{IV}}\left(x,\tau \right)$, fidelity temperature $T^{\mathrm{IV}}\left(x,\tau \right)$, and $V^{\mathrm{IV}}\left(x,\tau \right)$, together with its singular first-order differential equation with $\alpha^{\mathrm{IV}}\left(x,\tau \right)$, take the same form as Equations (A146)–(A149) for regime I, with the label being changed from I to IV, respectively.

In order to solve a singular first-order differential equation in each principal part and in each principal regime, we perform a scaling analysis of $\alpha^{q}\left(x\right)$ (q= i, ii, iii, and v) and $\alpha^{\omega }\left(x,\tau \right)$ (ω = I, II, III, and IV) in the vicinity of a critical point $x_{c}=0$, which falls into three universality classes: (A) the Gaussian universality class for part v, regime II, and regime IV; (B) the Ising universality class for part ii and part iii and regime I and regime III; (C) the KT universality class for part i.

(A) If a Gaussian critical point $x_{c}=0$ is approached, fidelity entropy $S^{v}\left(x\right)$ scales as $S^{v}\left(x\right)∼x^{\nu (0)+1}$ in part v, fidelity entropy $S^{\mathrm{II}}\left(x,\tau \right)$ and fidelity entropy $S^{\mathrm{IV}}\left(x,\tau \right)$ scale as $S^{\mathrm{II}}\left(x,\tau \right)∼x^{\nu (\tau )+1}$ in regime II, and $S^{\mathrm{IV}}\left(x,\tau \right)∼x^{\nu (\tau )+1}$ in regime IV, respectively, with ν(τ), as a function of τ, being the critical exponent for the correlation length (cf. Appendix H). In addition, our numerical simulation shows that the ground-state energy density $e^{v}\left(x\right)$ near a critical point $x_{c}=0$ scales as follows:

(A150) $$\ln\frac{e^{v}\left(x\right)}{e^{v}\left(0\right)}∼x^{K(0)}\;\ln x,$$

and, for a fixed τ∈(−1,0), our numerical simulation shows that the ground-state energy density $e^{\mathrm{II}/\mathrm{IV}}\left(x,\tau \right)$ near a critical point $x_{c}=0$ scales as follows

(A151) $$\ln\frac{e^{\mathrm{II}/\mathrm{IV}}\left(x,\tau \right)}{e^{\mathrm{II}/\mathrm{IV}}\left(0,\tau \right)}∼x^{K(\tau )}\;\ln x.$$

In part v, with ν(0)<K(0)≤ν(0)+1, $\alpha^{v}\left(x\right)$ scales as follows:

(A152) $$\alpha^{v}\left(x\right)\propto x^{K(0)-\nu (0)-1}\ln x,$$

and in regime II and regime IV, as long as ν(τ)<K(τ)≤ν(τ)+1, $\alpha^{\mathrm{II}/\mathrm{IV}}\left(x,\tau \right)$ scales as follows

(A153) $$\alpha^{\mathrm{II}/\mathrm{IV}}\left(x,\tau \right)\propto x^{K(\tau )-\nu (\tau )-1}\ln x.$$

Our numerical simulations confirm this analysis, as shown in Figure A12 for regime II and part v. For regime IV, we have K(τ)≃1.13 and ν(τ)≃0.78 for τ=0.1, and K(τ)≃1.14 and ν(τ)≃0.78 for τ≃0.2, respectively.

(B) If a Ising critical point $x_{c}=0$ is approached, fidelity entropy $S^{q}\left(x\right)$ scales as $S^{q}\left(x\right)∼x^{2}$ in part q, with q= ii and iii, and $S^{\omega }\left(x,\tau \right)$ scales as $S^{\omega }\left(x,\tau \right)∼x^{2}$ in regime ω, with q= I and III. This indicates that the critical exponent is ν=1 (cf. Appendix H for details), consistent with the previous work on the Ising transition between the Haldane phase and the ${\mathrm{AF}}_{x}$ phase [163,230]. Taking into account the fact that the first-order derivative of $\ln\left(e^{q}\left(x\right)/e^{q}\left(0\right)\right)$ and $\ln\left(e^{\omega }\left(x,\tau \right)/e^{\omega }\left(0,\tau \right)\right)$ with respect to x at a critical point $x_{c}=0$ is nonzero [230], $\alpha^{q}\left(x\right)$ and $\alpha^{\omega }\left(x,\tau \right)$ scale as

(A154) $$\alpha^{q}\left(x\right)∼\frac{1}{x},$$

and

(A155) $$\alpha^{\omega }\left(x,\tau \right)∼\frac{1}{x},$$

respectively. The scaling behaviors for part ii and part iii and for regime I and regime III are the same, as anticipated from the fact that all the phase transitions belong to the Ising universality class. Our numerical simulations confirm this analysis.

(C) In part i, when a KT transition point $x_{c}=0$ is approached, fidelity entropy $S^{i}\left(x\right)$ scale as $S^{i}\left(x\right)∼x^{3}$. Taking into account the fact that the first-order derivative of $\ln\left(e^{i}\left(x\right)/e^{i}\left(0\right)\right)$ with respect to x, at a critical point $x_{c}=0$, is nonzero, $\alpha^{i}\left(x\right)$ scales as follows

(A156) $$\alpha^{i}\left(x\right)∼\frac{1}{x}.$$

Our numerical simulations confirm this analysis.

Since the integration of $\alpha^{q}\left(x\right)$ and $\alpha^{\omega }\left(x,\tau \right)$ with respect to x is finite, the singular first-order differential equations may be solved in a straightforward manner.

Let us first determine V(x,τ) in the five principal parts.

(a) In principal part i on the U(1)-symmetric line (γ=0 with $\Delta_{c1}<\Delta <1$), since the integration of $\alpha^{i}\left(x\right)$ with respect to x is finite, the singular first-order differential equation, Equation (A143), may be solved as follows:

(A157) $$V^{i}\left(x\right)=V_{0}^{i}V_{1}^{i}\left(x\right),$$

where $V_{0}^{i}$ is a constant, and $V_{1}^{i}\left(\Delta ,0\right)$ is defined as follows

(A158) $$V_{1}^{i}\left(x\right)=\exp\left(\int_{0}^{x}\alpha^{i}\left(y\right)dy\right).$$

(b) In principal parts ii, iii, and v, the solutions $V^{\mathrm{ii}}\left(x\right)$, $V^{\mathrm{iii}}\left(x\right)$, and $V^{v}\left(x\right)$ to the singular first-order differential equations take the same form as Equations (A157) and (A158) for part i, with the label being changed from i to ii, iii, and v, respectively.

(c) In principal part iv on the factorizing-field line (γ=1+Δ with 0<γ<1), $V^{i}\left(x\right)$ vanishes: $V^{i}\left(x\right)=0$.

The singular first-order differential equation, Equation (A147), for regime I, and their counterparts for regime III, regime II and regime IV, may be solved in a similar manner.

(a) In regime I ($0<\Delta <\Delta_{c0}$ and 0<γ<1), since the integration of $\alpha^{I}\left(x,\tau \right)$ with respect to x, for a fixed τ, is finite, the singular first-order differential equation, Equation (A147), may be solved as follows:

(A159) $$V^{I}\left(x,\tau \right)=V_{0}^{I}\left(\tau \right)V_{1}^{I}\left(x,\tau \right),$$

where $V_{0}^{I}\left(\tau \right)$ is a function of τ, and $V_{1}^{I}\left(x,\tau \right)$ is defined as follows

(A160) $$V_{1}^{I}\left(x,\tau \right)=\exp\left(\int_{0}^{x}\alpha^{I}\left(y,\tau \right)dy\right).$$

(b) In regime II (−1<Δ<0 and 0<γ<1+Δ), regime III (0<Δ<1, $0<\gamma <1-\Delta_{c0}$), and regime IV ($0<\Delta <\Delta_{c1}$ and $0<\gamma <1-\Delta_{c1}$), the solutions $V^{\mathrm{II}}\left(x,\tau \right)$, $V^{\mathrm{III}}\left(x,\tau \right)$, and $V^{\mathrm{IV}}\left(x,\tau \right)$ to the singular first-order differential equations take the same form as Equations (A159) and (A160) for regime I, with the label being changed from I to II, III, IV, and V, respectively.

Now, we turn to the residual fidelity entropy $S_{0}^{q}$ for the five principal parts, with q=i, ii, iii, iv and v. We remark that fidelity entropy $S_{f}\left(x,\tau \right)$, generically, is *relative* in the sense that it is *only* determined up to a constant. Here, as a convention, we chose fidelity entropy $S_{f}\left(x,\tau \right)$ to be zero at the critical point $x_{c}$, with $x_{c}=0$, in part v. Hence, we have $S_{0}^{v}=0$. Therefore, fidelity entropy $S_{f}^{v}\left(x\right)$ in part v follows from its definition, which takes the same form as Equation (A140), with the label being changed from i to v. Here, we denote fidelity entropy $S_{f}^{v}\left(1\right)$ at the characteristic point x=1 or equivalently Δ=0 and γ=1, as $S_{m}$. Then, the residual fidelity entropy $S_{0}^{\mathrm{ii}}$ and $S_{0}^{\mathrm{iv}}$ are determined from the continuity requirement for fidelity entropy at the U(1)-symmetric point (0,1): $S_{f}^{\mathrm{ii}}\left(1\right)=S_{m}$ and $S_{f}^{\mathrm{iv}}\left(1\right)=S_{m}$. Following from the continuity requirement at the Ising transition point, the residual fidelity entropy $S_{0}^{\mathrm{iii}}$ in part iii is determined: $S_{0}^{\mathrm{iii}}=S_{0}^{\mathrm{ii}}$. In addition, the residual fidelity entropy $S_{0}^{i}$ on the U(1)-symmetric line (γ=0 with $\Delta_{c1}<\Delta <1$), labelled as i, is determined from the continuity requirement for fidelity entropy at the KT transition point ($\Delta_{c1}$, 0), which is identical to the residual fidelity entropy $S_{0}$ at the KT transiton point on the complete line of critical points (γ=0 with $-1<\Delta <\Delta_{c1}$) from an interior point of view (cf. Appendix O.2), as a result of an analogue of the Hawking radiation (cf. scenario III-1).

We are free to choose $V_{0}$ on one of the characteristic lines, since fidelity internal energy $U_{f}\left(x,\tau \right)$ is only determined up to a constant factor. Here, we set $V_{0}^{v}=1$ on the semi-self-dual line (Δ=0 with 0<γ<1), labelled as v. To ensure that fidelity temperature $T_{f}^{v}\left(x\right)$ at the characteristic point x=1, or equivalently Δ=0 and γ=1, is zero, a shift from $T^{v}\left(x\right)$ to $T^{v}\left(x\right)-T_{0}^{v}$ is performed in part v, which induces a shift in fidelity internal energy: $U^{v}\left(x\right)-T_{0}^{v}S^{v}\left(x\right)$. Here, $T_{0}^{v}\equiv T^{v}\left(1\right)$. We refer to $T^{v}\left(x\right)-T_{0}^{v}$ and $U^{v}\left(x\right)-T_{0}^{v}S^{v}\left(x\right)$ as $T_{f}^{v}\left(x\right)$ and $U_{f}^{v}\left(x\right)$, respectively. That is, $T_{f}^{v}\left(x\right)\equiv T^{v}\left(x\right)-T_{0}^{v}$ and $U_{f}^{v}\left(x\right)\equiv U^{v}\left(x\right)-T_{0}^{v}S^{v}\left(x\right)$. Therefore, fidelity internal energy $U_{f}^{v}\left(x\right)$ on the semi-self-dual line (Δ=0 with 0<γ<1), labelled as v, is determined. Here, we denote fidelity internal energy at the characteristic point x=1, or equivalently Δ=0 and γ=1, as $U_{m}$. Then, we require that fidelity internal energy at the AKLT point takes a maximum value, $U_{m}$. Therefore, fidelity temperature $T_{H}$ and fidelity internal energy $U_{H}$ at the SU(2)-symmetric point (Δ=1 and γ=0) are determined by taking the same values as those for the spin-1 bilinear–biquadratic model at the same point, with D=0 (cf. Appendix O.3). We are able to determine $T_{0}^{q}$ for the other principal parts, labelled as q, with q=i, ii, iii, and iv. In part i, we have $T_{0}^{i}=T^{i}\left(1-\Delta_{c1}\right)-T_{H}$. In part ii, we have $T_{0}^{\mathrm{ii}}=T^{\mathrm{ii}}\left(\Delta_{c0}/\left(2-\Delta_{c0}\right)\right)$. In part iii, we have $T_{0}^{\mathrm{iii}}=T^{\mathrm{iii}}\left(1-\Delta_{c}/\left(2-\Delta_{c}\right)\right)-T_{H}$. In part iv, we have $T_{0}^{\mathrm{iv}}=0$.

Now we need to determine $V_{0}$ and $U_{0}$ in the remaining four principal parts: part i, part ii, part iii, and part iv.

(i) In principal part i on the U(1)-symmetric line (γ=0 with $\Delta_{c1}<\Delta <1$), in order to ensure the continuity requirement for fidelity temperature at the SU(2)-symmetric point (Δ=1 and γ=0), $T^{i}\left(x\right)$ is shifted to $T^{i}\left(x\right)-T_{0}^{i}$, accompanied by a shift in $U^{i}\left(x\right)$: $U^{i}\left(x\right)-T_{0}^{i}S^{i}\left(x\right)$, with $S^{i}\left(x\right)$ left intact. In addition, fidelity internal energy $U^{i}\left(x\right)$ at a critical point $x_{c}=0$ must be zero, and fidelity internal energy $U^{i}\left(x\right)$ satisfies the continuity requirement at the SU(2)-symmetric point (Δ=1 and γ=0), with $x=1-\Delta_{c1}$, as discussed in Section 2: $U_{0}^{i}=T_{0}^{i}S_{0}^{i}$ and $U^{i}\left(1-\Delta_{c1}\right)-T_{0}^{i}S^{i}\left(1-\Delta_{c1}\right)=U_{H}$. Therefore, $V_{0}^{i}$ is determined as follows

(A161) $$V_{0}^{i}=\frac{U_{H}-T_{H}\left(S^{i}\left(1-\Delta_{c1}\right)-S_{0}^{i}\right)}{\ln\left(e^{i}\left(1-\Delta_{c1}\right)/e^{i}\left(0\right)\right)V_{1}^{i}\left(1-\Delta_{c1}\right)+\alpha^{i}\left(1-\Delta_{c1}\right)V_{1}^{i}\left(1-\Delta_{c1}\right)\left(S^{i}\left(1-\Delta_{c1}\right)-S_{0}^{i}\right)}.$$

Once $V_{0}^{i}$ and $U_{0}^{i}$ are determined, fidelity temperature $T^{i}\left(x\right)$ and fidelity internal energy $U^{i}\left(x\right)$ follow from (A142) and (A145), respectively. We refer to $T^{i}\left(x\right)-T_{0}^{i}$ and $U^{i}\left(x\right)$$-T_{0}^{i}S^{i}\left(x\right)$ as $T_{f}^{i}\left(x\right)$ and $U_{f}^{i}\left(x\right)$, respectively. That is, $T_{f}^{i}\left(x\right)\equiv T^{i}\left(x\right)-T_{0}^{i}$ and $U_{f}^{i}\left(x\right)$$\equiv U^{i}\left(x\right)-T_{0}^{i}S^{i}\left(x\right)$.

(ii) In principal part ii on the U(1)-symmetric line (γ=1−Δ with $0<\Delta <\Delta_{c}$), in order to ensure that fidelity temperature vanishes at the characteristic point (Δ=0 and γ=1), $T^{\mathrm{ii}}\left(x\right)$ is shifted to $T^{\mathrm{ii}}\left(x\right)-T_{0}^{\mathrm{ii}}$, accompanied by a shift in $U^{\mathrm{ii}}\left(x\right)$: $U^{\mathrm{ii}}\left(x\right)-T_{0}^{\mathrm{ii}}S^{\mathrm{ii}}\left(x\right)$, with $S^{\mathrm{ii}}\left(x\right)$ left intact. In addition, fidelity internal energy $U^{\mathrm{ii}}\left(x\right)$ at a critical point $x_{c}$ must be zero, and fidelity internal energy $U^{\mathrm{ii}}\left(x\right)$ at the U(1)-symmetric point (0,1), or equivalently $x=\Delta_{c0}/\left(2-\Delta_{c0}\right)$, satisfies the continuity requirement, as discussed in Section 2: $U_{0}^{\mathrm{ii}}=T_{0}^{\mathrm{ii}}S_{0}^{\mathrm{ii}}$ and $U^{\mathrm{ii}}\left(\Delta_{c0}/\left(2-\Delta_{c0}\right)\right)-T_{0}^{\mathrm{ii}}S^{\mathrm{ii}}\left(\Delta_{c0}/\left(2-\Delta_{c0}\right)\right)=U_{m}$. Therefore, $V_{0}^{\mathrm{ii}}$ is determined as follows

(A162) $$V_{0}^{\mathrm{ii}}=\frac{U_{m}}{\ln\left(e^{\mathrm{ii}}\left(\Delta_{c0}/\left(2-\Delta_{c0}\right)\right)/e^{\mathrm{ii}}\left(0\right)\right)V_{1}^{\mathrm{ii}}\left(\Delta_{c0}/\left(2-\Delta_{c0}\right)\right)+\alpha^{\mathrm{ii}}\left(\Delta_{c0}/\left(2-\Delta_{c0}\right)\right)V_{1}^{\mathrm{ii}}\left(\Delta_{c0}/\left(2-\Delta_{c0}\right)\right)\left(S^{\mathrm{ii}}\left(\Delta_{c0}/\left(2-\Delta_{c0}\right)\right)-S_{0}^{\mathrm{ii}}\right)}.$$

Once $V_{0}^{\mathrm{ii}}$ and $U_{0}^{\mathrm{ii}}$ are determined, fidelity temperature $T^{\mathrm{ii}}\left(\Delta ,1-\Delta \right)$ and fidelity internal energy $U^{\mathrm{ii}}\left(\Delta ,1-\Delta \right)$ follow from their counterparts of (A142) and (A145), with the label being changed from i to ii, respectively. We refer to $T^{\mathrm{ii}}\left(x\right)-T_{0}^{\mathrm{ii}}$ and $U^{\mathrm{ii}}\left(x\right)-T_{0}^{\mathrm{ii}}S^{\mathrm{ii}}\left(x\right)$ as $T_{f}^{\mathrm{ii}}\left(x\right)$ and $U_{f}^{\mathrm{ii}}\left(x\right)$, respectively. That is, $T_{f}^{\mathrm{ii}}\left(x\right)\equiv T^{\mathrm{ii}}\left(x\right)-T_{0}^{\mathrm{ii}}$ and $U_{f}^{\mathrm{ii}}\left(x\right)\equiv U^{\mathrm{ii}}\left(x\right)-T_{0}^{\mathrm{ii}}S^{\mathrm{ii}}\left(x\right)$.

(iii) In principal part iii on the U(1)-symmetric line (γ=1−Δ with $\Delta_{c}<\Delta <1$), in order to ensure that fidelity temperature is continuous at the SU(2)-symmetric point Δ=1 and γ=0, $T^{\mathrm{iii}}\left(x\right)$ is shifted to $T^{\mathrm{iii}}\left(x\right)-T_{0}^{\mathrm{iii}}$, accompanied by a shift in $U^{\mathrm{iii}}\left(x\right)$: $U^{\mathrm{iii}}\left(x\right)$$-T_{0}^{\mathrm{iii}}S^{\mathrm{iii}}\left(x\right)$, with $S^{\mathrm{iii}}\left(x\right)$ left intact. In addition, fidelity internal energy $U^{\mathrm{iii}}\left(x\right)$ at a critical point $x_{c}$, with $x_{c}=0$, must be zero, and fidelity internal energy $U^{\mathrm{iii}}\left(x\right)$ satisfies the continuity requirement at the SU(2)-symmetric point (Δ=1 and γ=0, or equivalently $x_{m}$$=1-\Delta_{c0}/\left(2-\Delta_{c0}\right)$, as discussed in Section 2: $U_{0}^{\mathrm{iii}}=T_{0}^{\mathrm{iii}}S_{0}^{\mathrm{iii}}$ and $U^{\mathrm{iii}}\left(x_{m}\right)-T_{0}^{\mathrm{iii}}S^{\mathrm{iii}}\left(x_{m}\right)$$=U_{H}$, with $U_{H}$ denoting fidelity internal energy at the same point for the spin-1 bilinear–biquadratic model. Therefore, $V_{0}^{\mathrm{iii}}$ is determined as follows

(A163) $$V_{0}^{\mathrm{iii}}=\frac{U_{H}-T_{H}\left(S^{\mathrm{iii}}\left(x_{m}\right)-S_{0}^{\mathrm{iii}}\right)}{\ln\left(e^{\mathrm{iii}}\left(x_{m}\right)/e^{\mathrm{iii}}\left(0\right)\right)V_{1}^{\mathrm{iii}}\left(x_{m}\right)+\alpha^{\mathrm{iii}}\left(x_{m}\right)V_{1}^{\mathrm{iii}}\left(x_{m}\right)\left(S^{\mathrm{iii}}\left(x_{m}\right)-S_{0}^{\mathrm{iii}}\right)}.$$

Once $V_{0}^{\mathrm{iii}}$ and $U_{0}^{\mathrm{iii}}$ are determined, fidelity temperature $T^{\mathrm{iii}}\left(x\right)$ and fidelity internal energy $U^{\mathrm{iii}}\left(x\right)$ follow from their counterparts of (A142) and (A145), with the label being changed from i to iii, respectively. We refer to $T^{\mathrm{iii}}\left(x\right)-T_{0}^{\mathrm{iii}}$ and $U^{\mathrm{iii}}\left(x\right)-T_{0}^{\mathrm{iii}}S^{\mathrm{iii}}\left(x\right)$ as $T_{f}^{\mathrm{iii}}\left(x\right)$ and $U_{f}^{\mathrm{iii}}\left(x\right)$, respectively. That is, $T_{f}^{\mathrm{iii}}\left(x\right)\equiv T^{\mathrm{iii}}\left(x\right)-T_{0}^{\mathrm{iii}}$ and $U_{f}^{\mathrm{iii}}\left(x\right)\equiv U^{\mathrm{iii}}\left(x\right)-T_{0}^{\mathrm{iii}}S^{\mathrm{iii}}\left(x\right)$.

(iv) In principal part iv on the factorizing-field line (γ=1+Δ with −1<Δ<0), $V^{\mathrm{iv}}\left(x\right)$ vanishes; thus, $V_{0}^{\mathrm{iv}}=0$ and $U_{0}^{\mathrm{iv}}=0$.

Now we turn to the residual fidelity entropy on the line of the Ising critical points between the ${\mathrm{AF}}_{x}$ phase and the Haldane phase in regime I or regime III, denoted as $S_{0}^{I}\left(\tau \right)$ or $S_{0}^{\mathrm{III}}\left(\tau \right)$, respectively, and the residual fidelity entropy on the line of the Gaussian critical points: γ=0, with $-1<\Delta \leq \Delta_{c1}$ in regime II and regime IV, denoted as $S_{0}^{\mathrm{II}}\left(\tau \right)$ and $S_{0}^{\mathrm{IV}}\left(\tau \right)$, respectively. In regime I, the residual fidelity entropy $S_{0}^{I}\left(\tau \right)$ is determined from the continuity requirement on the semi-self-dual line (Δ=0 with 0<γ<1), labelled as v: $S^{I}\left(\tau \right)=S_{f}^{v}\left(\tau \right)$. Then, the residual fidelity entropy $S_{0}^{\mathrm{III}}\left(\tau \right)$ is determined from the continuity requirement on the line of the Ising critical points between the ${\mathrm{AF}}_{x}$ phase and the Haldane phase: $S_{0}^{\mathrm{III}}\left(\tau \right)=S_{0}^{I}\left(\tau \right)$. In regime II, the residual fidelity entropy $S_{0}^{\mathrm{II}}\left(\tau \right)$ is determined from the continuity requirement on the factorizing-field line (γ=1+Δ with −1<Δ<0), labelled as iv. Note that, the continuity requirement for fidelity entropy at a point on the factorizing-field line (γ=1+Δ with −1<Δ<0), labelled as iv, implies that fidelity entropy in regime II includes contributions from scaling entropy $S_{\sigma }^{\mathrm{II}}\left(x,\tau \right)$ due to a re-parametrization operation in the ground-state energy density on the factorizing-field line (γ=1+Δ with −1<Δ<0), lablled as iv. Hence, we have $S_{f}^{\mathrm{II}}\left(x,\tau \right)=S^{\mathrm{II}}\left(x,\tau \right)+S_{\sigma }^{\mathrm{II}}\left(x,\tau \right)$, where $S_{\sigma }^{\mathrm{II}}\left(x,\tau \right)=S_{\sigma }^{\mathrm{iv}}\left(\tau +1\right)$. In regime IV, the residual fidelity entropy $S_{0}^{\mathrm{IV}}\left(\tau \right)$ is determined from the continuity requirement on the semi-self-dual line (Δ=0 with 0<γ<1), labelled as v: $S^{\mathrm{IV}}\left(\tau \right)=S_{f}^{v}\left(\tau \right)$. Then, $S_{0}^{\mathrm{III}}\left(\tau \right)$ is determined from the continuity requirement on the line of the Ising critical points between the ${\mathrm{AF}}_{x}$ phase and the Haldane phase: $S_{0}^{\mathrm{III}}\left(\tau \right)=S_{0}^{I}\left(\tau \right)$. Moreover, the continuity requirement for $S_{f}\left(x,\tau \right)$ on the factorizing-field line, labelled as iv, leads to a contribution to $S_{f}^{\mathrm{II}}\left(x,\tau \right)$ in regime II from scaling entropy $S_{\sigma }^{\mathrm{II}}\left(\tau \right)$ due to a re-parametrization operation in the ground-state energy density. Hence, we have $S_{f}^{\mathrm{II}}\left(x,\tau \right)=S^{\mathrm{II}}\left(x,\tau \right)+S_{\sigma }^{\mathrm{II}}\left(\tau \right)$. Here, $S_{\sigma }^{\mathrm{II}}\left(\tau \right)=S_{\sigma }^{\mathrm{iv}}\left(\tau +1\right)=\ln\left(2+\tau \right)-\ln\left(2\right)$.

We have to ensure that fidelity mechanical-state functions are *continuous* at the boundaries of the four principal regimes. Indeed, we have already taken into account the continuity requirement for fidelity entropy; the remaining task is to ensure continuity requirements for fidelity temperature and fidelity internal energy. As discussed in Section 2, we need to determine $T_{0}^{\omega }\equiv T_{m}^{\omega }-T_{t}^{\omega }$ for the four principal regimes, with ω=I, II, III, and IV. Here, $T_{m}^{\omega }$ represents fidelity temperature, evaluated from a dominant control parameter x in one principal regime ω, at a chosen point in a principal part, which is a boundary in a principal regime ω, whereas $T_{t}^{\omega }$ represents fidelity temperature at the same point, but it is evaluated from a dominant control parameter x in the principal part itself. Specifically, in regime I, for a fixed τ∈(0,1), we have $T_{m}^{I}\left(\tau \right)=T^{I}\left(\Delta_{c}/\left(2-\Delta_{c}\right),\tau \right)$ and $T_{t}^{I}\left(\tau \right)=T^{v}\left(\tau \right)$. Here, $T^{I}\left(\Delta_{c}/\left(2-\Delta_{c}\right),\tau \right)$ is evaluated from the chosen dominant control parameter x in the principal regime for a fixed τ, which is along the straight line (γ=τ−Δ), and $T^{v}\left(\tau \right)$ is evaluated from the semi-self-dual line (Δ=0 with 0<γ<1), labelled as v, for the same chosen point. $\Delta =\Delta_{c}$ and $\gamma =\tau -\Delta_{c}$ is the Ising transition point between the Haldane phase and the ${\mathrm{AF}}_{x}$ phase for a fixed τ. In regime II, for a fixed τ∈(−1,0), we have $T_{m}^{\mathrm{II}}\left(\tau \right)=T^{\mathrm{II}}\left(1+\tau ,\tau \right)$ and $T_{t}^{\mathrm{II}}\left(\tau \right)=T^{\mathrm{iv}}\left(\tau +1\right)$, with $T^{\mathrm{iv}}\left(\tau +1\right)$ denoting fidelity temperature on the factorizing-field line (γ=1+Δ with −1<Δ<0), labelled as iv. In regime III, for a fixed τ∈(0,1), we have $T_{m}^{\mathrm{III}}\left(\tau \right)=T^{\mathrm{III}}\left(\tau /\left(2-\tau \right)-\Delta_{c}/\left(2-\Delta_{c}\right),\tau \right)$ and $T_{t}^{\mathrm{III}}\left(\tau \right)=T_{f}^{i}\left(\tau \right)$. Here, $T^{\mathrm{III}}\left(\tau /\left(2-\tau \right)-\Delta_{c}/\left(2-\Delta_{c}\right),\tau \right)$ is evaluated from a dominant control parameter on the line (γ=τ−Δ), and $T_{f}^{i}\left(\tau \right)$ is evaluated from a dominant control parameter on the U(1)-symmetric line (γ=0 with 0<Δ<1), labelled as i. In regime IV, for a fixed $\tau \in (0,\Delta_{c1})$, we have $T_{m}^{\mathrm{IV}}\left(\tau \right)=T^{\mathrm{IV}}\left(\tau /\left(2-\tau \right),\tau \right)$ and $T_{t}^{I}\left(\tau \right)=T^{v}\left(\tau \right)$. Here, $T^{\mathrm{IV}}\left(\tau /\left(2-\tau \right),\tau \right)$ is evaluated along the line (γ=τ−Δ), and $T_{f}^{i}\left(\tau \right)$ is evaluated from a dominant control parameter on the semi-self-dual line (Δ=0 with 0<γ<1), labelled as v.

Now, let us determine $V_{0}$ and $U_{0}$ for the four principal regimes.

(1) In regime I ($0<\Delta <\Delta_{c0}$ and 0<γ<1), in order to ensure the continuity requirement for fidelity temperature on the semi-self-dual line (Δ=0 with 0<γ<1), labelled as v, $T^{I}\left(x,\tau \right)$ is shifted to $T^{I}\left(x,\tau \right)-T_{0}^{I}\left(\tau \right)$, with $T_{0}^{I}\left(\tau \right)=T^{I}\left(\Delta_{c}/\left(2-\Delta_{c}\right),\tau \right)-T^{v}\left(\tau \right)$. Here, $\left(\Delta_{c},\tau -\Delta_{c}\right)$ is the Ising transition point between the Haldane phase and the ${\mathrm{AF}}_{x}$ phase. Then, $U^{I}\left(x,\tau \right)$ is shifted to $U^{I}\left(x,\tau \right)-T_{0}^{I}\left(\tau \right)S^{I}\left(x,\tau \right)$, with $S^{I}\left(x,\tau \right)$ left intact. In addition, fidelity internal energy $U^{I}\left(x,\tau \right)$ on the line of the Ising critical points must be zero, and fidelity internal energy $U^{I}\left(x,\tau \right)$ satisfies the continuity requirement on the semi-self-dual line (Δ=0 with 0<γ<1), labelled as v, as discussed in Section 2: $U_{0}^{I}\left(\tau \right)=T_{0}^{I}\left(\tau \right)S_{0}^{I}\left(\tau \right)$ and $U^{I}\left(\Delta_{c}/\left(2-\Delta_{c}\right),\tau \right)-T_{0}^{I}\left(\Delta_{c}/\left(2-\Delta_{c}\right),\tau \right)S^{I}\left(\Delta_{c}/\left(2-\Delta_{c}\right),\tau \right)=U_{f}^{v}\left(\tau \right)$. Therefore, $V_{0}^{I}\left(\tau \right)$ is determined as follows

(A164) $$V_{0}^{I}\left(\tau \right)=\frac{U_{f}^{v}\left(\tau \right)-T_{f}^{v}\left(\tau \right)\left(S^{I}\left(\Delta_{c}/\left(2-\Delta_{c}\right),\tau \right)-S_{0}^{I}\left(\tau \right)\right)}{\ln\left(e^{I}\left(\Delta_{c}/\left(2-\Delta_{c}\right),\tau \right)/e^{I}\left(0,\tau \right)\right)V_{1}^{I}\left(x_{0},\tau \right)+\alpha^{I}\left(\Delta_{c}/\left(2-\Delta_{c}\right),\tau \right)V_{1}^{I}\left(\Delta_{c}/\left(2-\Delta_{c}\right),\tau \right)\left(S^{I}\left(\Delta_{c}/\left(2-\Delta_{c}\right),\tau \right)-S_{0}^{I}\left(\tau \right)\right)}.$$

Once $V_{0}^{I}\left(\tau \right)$ and $U_{0}^{I}\left(\tau \right)$ are determined, fidelity temperature $T^{I}\left(x,\tau \right)$ and fidelity internal energy $U^{I}\left(x,\tau \right)$ follow from (A146) and (A149), respectively. Following from our discussions in Section 2, fidelity entropy $S_{f}^{I}\left(x,\tau \right)$, fidelity temperature $T_{f}^{I}\left(x,\tau \right)$, and fidelity internal energy $U_{f}^{I}\left(x,\tau \right)$ take the following form: $S_{f}^{I}\left(x,\tau \right)=S^{I}\left(x,\tau \right)$, $T_{f}^{I}\left(x,\tau \right)=T^{I}\left(x,\tau \right)-T_{0}^{I}\left(\tau \right)$ and $U_{f}^{I}\left(x,\tau \right)=U^{I}\left(x,\tau \right)-T_{0}^{I}\left(\tau \right)S^{I}\left(x,\tau \right)$, respectively.

(2) In regime II (−1<Δ<0 and 0<γ<1+Δ), in order to ensure the continuity requirement for fidelity temperature on the U(1)-symmetric line (γ=1−Δ with $0<\Delta <\Delta_{c0}$), $T^{\mathrm{II}}\left(x,\tau \right)$ is shifted to $T^{\mathrm{II}}\left(x,\tau \right)-T_{0}^{\mathrm{II}}\left(\tau \right)$, with $T_{0}^{\mathrm{II}}\left(\tau \right)=T^{\mathrm{II}}\left(1+\tau ,\tau \right)$. Then, $U^{\mathrm{II}}\left(x,\tau \right)$ is shifted to $U^{\mathrm{II}}\left(x,\tau \right)-T_{0}^{\mathrm{II}}\left(\tau \right)S^{\mathrm{II}}\left(x,\tau \right)$, with $S^{\mathrm{II}}\left(x,\tau \right)$ left intact. Here, $T_{0}^{\mathrm{II}}\left(\Delta \right)=T^{\mathrm{II}}\left(\Delta ,1+\Delta \right)$. In addition, fidelity internal energy $U^{\mathrm{II}}\left(x,\tau \right)$ on the line of the Gaussian critical points (γ=0 with $-1<\Delta <\Delta_{c1}$), or equivalently, $x=x_{c}=0$, for a fixed τ, must be zero, and fidelity internal energy $U^{\mathrm{II}}\left(x,\tau \right)$ satisfies the continuity requirement in principal part iv, as discussed in Section 2: $U_{0}^{\mathrm{II}}\left(\tau \right)=T_{0}^{\mathrm{II}}\left(\tau \right)S_{0}^{\mathrm{II}}\left(\tau \right)$ and $U^{\mathrm{II}}\left(1+\tau ,\tau \right)-T_{0}^{\mathrm{II}}\left(\tau \right)S^{\mathrm{II}}\left(1+\tau ,\tau \right)=U_{m}$. Therefore, $V_{0}^{\mathrm{II}}\left(\tau \right)$ is determined as follows

(A165) $$V_{0}^{\mathrm{II}}\left(\tau \right)=\frac{U_{m}}{\ln\left(e^{\mathrm{II}}\left(1+\tau ,\tau \right)/e^{\mathrm{II}}\left(0,\tau \right)\right)V_{1}^{\mathrm{II}}\left(1+\tau ,\tau \right)+\alpha^{\mathrm{II}}\left(1+\tau ,\tau \right)V_{1}^{\mathrm{II}}\left(1+\tau ,\tau \right)\left(S^{\mathrm{II}}\left(1+\tau ,\tau \right)-S_{0}^{\mathrm{II}}\left(\tau \right)\right)}.$$

Once $V_{0}^{\mathrm{II}}\left(\tau \right)$ and $U_{0}^{\mathrm{II}}\left(\tau \right)$ are determined, fidelity temperature $T^{\mathrm{II}}\left(x,\tau \right)$ and fidelity internal energy $U^{\mathrm{II}}\left(x,\tau \right)$ follow from their counterparts of (A146) and (A149), with the label being changed from I to II. Following from our discussions in Section 2, fidelity entropy $S_{f}^{\mathrm{II}}\left(x,\tau \right)$, fidelity temperature $T_{f}^{\mathrm{II}}\left(x,\tau \right)$, and fidelity internal energy $U_{f}^{\mathrm{II}}\left(x,\tau \right)$ take the following form: $S_{f}^{\mathrm{II}}\left(x,\tau \right)=S^{\mathrm{II}}\left(x,\tau \right)$, $T_{f}^{\mathrm{II}}\left(x,\tau \right)=T^{\mathrm{II}}\left(x,\tau \right)-T_{0}^{\mathrm{II}}\left(\tau \right)$, and $U_{f}^{\mathrm{II}}\left(x,\tau \right)=U^{\mathrm{II}}\left(x,\tau \right)-T_{0}^{\mathrm{II}}\left(\tau \right)S^{\mathrm{II}}\left(x,\tau \right)$, respectively.

(3) In regime III (0<Δ<1, $0<\gamma <1-\Delta_{c0}$), in order to ensure the continuity requirement for fidelity temperature on the U(1)-symmetric line (γ=0 with 0<Δ<1), $T^{\mathrm{III}}\left(x,\tau \right)$ is shifted to $T^{\mathrm{III}}\left(x,\tau \right)-T_{0}^{\mathrm{III}}\left(\tau \right)$, with $T_{0}^{\mathrm{III}}\left(\tau \right)=T^{\mathrm{III}}\left(\tau /\left(2-\tau \right)-\Delta_{c}/\left(2-\Delta_{c}\right),\tau \right)$$-T_{f}^{\mathrm{iv}}\left(\tau \right)$. Here, $\left(\Delta_{c},\tau -\Delta_{c}\right)$ is the Ising transition point between the Haldane phase and the ${\mathrm{AF}}_{x}$ phase, for a fixed τ. Then, $U^{\mathrm{III}}\left(x,\tau \right)$ is shifted to $U^{\mathrm{III}}\left(x,\tau \right)-T_{0}^{\mathrm{III}}\left(\tau \right)S^{\mathrm{III}}\left(x,\tau \right)$, with $S^{\mathrm{III}}\left(x,\tau \right)$ left intact. In addition, fidelity internal energy $U^{\mathrm{III}}\left(x,\tau \right)$ on the line of the Ising critical points must be zero, and fidelity internal energy $U^{\mathrm{III}}\left(x_{m},\tau \right)$ satisfies the continuity requirement on the U(1)-symmetric line, labelled as i, as discussed in Section 2: $U_{0}^{\mathrm{III}}\left(\tau \right)=T_{0}^{I}\left(\tau \right)S_{0}^{\mathrm{III}}\left(\tau \right)$ and $U^{\mathrm{III}}\left(x_{m},\tau \right)-T_{0}^{\mathrm{III}}\left(\tau \right)S^{\mathrm{III}}\left(x_{m},\tau \right)=U_{f}^{i}\left(\tau \right)$. Here, $x_{m}$$=\tau /\left(2-\tau \right)-\Delta_{c}/\left(2-\Delta_{c}\right)$. Therefore, $V_{0}^{\mathrm{III}}\left(\tau \right)$ is determined as follows

(A166) $$V_{0}^{\mathrm{III}}\left(\tau \right)=\frac{U_{f}^{i}\left(\tau \right)-T_{f}^{i}\left(\tau \right)\left(S^{\mathrm{III}}\left(x_{m},\tau \right)-S_{0}^{\mathrm{III}}\left(\tau \right)\right)}{\ln\left(e^{\mathrm{III}}\left(x_{m},\tau \right)/e^{\mathrm{III}}\left(0,\tau \right)\right)V_{1}^{\mathrm{III}}\left(x_{m},\tau \right)+\alpha^{\mathrm{III}}\left(x_{m},\tau \right)V_{1}^{I}\left(x_{m},\tau \right)\left(S^{\mathrm{III}}\left(x_{m},\tau \right)-S_{0}^{\mathrm{III}}\left(\tau \right)\right)}.$$

Once $V_{0}^{\mathrm{III}}\left(\tau \right)$ and $U_{0}^{\mathrm{III}}\left(\tau \right)$ are determined, fidelity temperature $T^{\mathrm{III}}\left(x,\tau \right)$ and fidelity internal energy $U^{\mathrm{III}}\left(x,\tau \right)$ follow from their counterparts of (A146) and (A149), with the label being changed from I to III. Following from our discussions in Section 2, fidelity entropy $S_{f}^{\mathrm{III}}\left(x,\tau \right)$, fidelity temperature $T_{f}^{\mathrm{III}}\left(x,\tau \right)$, and fidelity internal energy $U_{f}^{\mathrm{III}}\left(x,\tau \right)$ take the following form: $S_{f}^{\mathrm{III}}\left(x,\tau \right)=S^{\mathrm{III}}\left(x,\tau \right)$, $T_{f}^{\mathrm{III}}\left(x,\tau \right)=T^{\mathrm{III}}\left(x,\tau \right)-T_{0}^{\mathrm{III}}\left(\tau \right)$, and $U_{f}^{\mathrm{III}}\left(x,\tau \right)=U^{\mathrm{III}}\left(x,\tau \right)-T_{0}^{\mathrm{III}}\left(\tau \right)S^{\mathrm{III}}\left(x,\tau \right)$, respectively.

(4) In regime IV ($0<\Delta <\Delta_{c1}$ and $0<\gamma <\Delta_{c1}$), in order to ensure the continuity requirement for fidelity temperature on the semi-self-dual line (Δ=0 with 0<γ<1), labelled as v, $T^{\mathrm{IV}}\left(x,\tau \right)$ is shifted to $T^{\mathrm{IV}}\left(x,\tau \right)-T_{0}^{\mathrm{IV}}\left(\tau \right)$, with $T_{0}^{\mathrm{IV}}\left(\tau \right)=T^{\mathrm{IV}}\left(\tau /\left(\tau -2\right),\tau \right)-T^{v}\left(\tau \right)$. Here, (τ/(2−τ),τ) is the Gaussian transition point. Then, $U^{\mathrm{IV}}\left(x,\tau \right)$ is shifted to $U^{\mathrm{IV}}\left(x,\tau \right)-T_{0}^{\mathrm{IV}}\left(\tau \right)S^{\mathrm{IV}}\left(x,\tau \right)$, with $S^{\mathrm{IV}}\left(x,\tau \right)$ left intact. In addition, fidelity internal energy $U^{\mathrm{IV}}\left(x,\tau \right)$ on the line of the Gaussian critical points with $x_{c}=0$ for a fixed τ, must be zero, and fidelity internal energy $U^{\mathrm{IV}}\left(x,\tau \right)$ satisfies the continuity requirement on the semi-dual line (Δ=0 with 0<γ<1), labelled as v, as discussed in Section 2: $U_{0}^{\mathrm{IV}}\left(\tau \right)=T_{0}^{\mathrm{IV}}\left(\tau \right)S_{0}^{\mathrm{IV}}\left(\tau \right)$ and $U^{\mathrm{IV}}\left(\tau /\left(2-\tau \right),\tau \right)-T_{0}^{\mathrm{IV}}\left(\tau /\left(2-\tau \right),\tau \right)S^{I}\left(\tau /\left(2-\tau \right),\tau \right)=U_{f}^{v}\left(\tau \right)$. Therefore, $V_{0}^{I}\left(\tau \right)$ is determined as follows

(A167) $$V_{0}^{\mathrm{IV}}\left(\tau \right)=\frac{U_{f}^{v}\left(\tau \right)-T_{f}^{v}\left(\tau \right)\left(S^{\mathrm{IV}}\left(\tau /\left(2-\tau \right),\tau \right)-S_{0}^{\mathrm{IV}}\left(\tau \right)\right)}{\ln\left(e^{\mathrm{IV}}\left(\tau /\left(2-\tau \right),\tau \right)/e^{\mathrm{IV}}\left(0,\tau \right)\right)V_{1}^{\mathrm{IV}}\left(x_{0},\tau \right)+\alpha^{\mathrm{IV}}\left(\tau /\left(2-\tau \right),\tau \right)V_{1}^{\mathrm{IV}}\left(\tau /\left(2-\tau \right),\tau \right)\left(S^{\mathrm{IV}}\left(\tau /\left(2-\tau \right),\tau \right)-S_{0}^{\mathrm{IV}}\left(\tau \right)\right)}.$$

After $V_{0}^{\mathrm{IV}}\left(\tau \right)$ and $U_{0}^{\mathrm{IV}}\left(\tau \right)$ are determined, fidelity temperature $T^{\mathrm{IV}}\left(x,\tau \right)$ and fidelity internal energy $U^{\mathrm{IV}}\left(x,\tau \right)$ follow from their counterparts of (A146) and (A149), with the label being changed from I to IV, respectively. Following from our discussions in Section 2, fidelity entropy $S_{f}^{\mathrm{IV}}\left(x,\tau \right)$, fidelity temperature $T_{f}^{\mathrm{IV}}\left(x,\tau \right)$, and fidelity internal energy $U_{f}^{\mathrm{IV}}\left(x,\tau \right)$ take the form: $S_{f}^{\mathrm{IV}}\left(x,\tau \right)=S^{\mathrm{IV}}\left(x,\tau \right)$, $T_{f}^{\mathrm{IV}}\left(x,\tau \right)=T^{\mathrm{IV}}\left(x,\tau \right)-T_{0}^{\mathrm{IV}}\left(\tau \right)$, and $U_{f}^{\mathrm{IV}}\left(x,\tau \right)$$=U^{\mathrm{IV}}\left(x,\tau \right)-T_{0}^{\mathrm{IV}}\left(\tau \right)S^{\mathrm{IV}}\left(x,\tau \right)$, respectively.

Once fidelity entropy $S_{f}^{q}\left(x\right)$, fidelity temperature $T_{f}^{q}\left(x\right)$, and fidelity internal energy $U_{f}^{q}\left(x\right)$ (q=i, ii, iii, iv and v) in the five principal parts and fidelity entropy $S_{f}^{\omega }\left(x,\tau \right)$, fidelity temperature $T_{f}^{\omega }\left(x,\tau \right)$, and fidelity internal energy $U_{f}^{\omega }\left(x,\tau \right)$ in the four principal regimes (ω = I, II, III and IV) are determined, fidelity entropy $S_{f}\left(\Delta ,\gamma \right)$, fidelity temperature $T_{f}\left(\Delta ,\gamma \right)$, and fidelity internal energy $U_{f}\left(\Delta ,\gamma \right)$ follow. That is, $S_{f}^{q}\left(\Delta ,\gamma \right)\equiv S_{f}^{q}\left(x\right)$ and $S_{f}^{\omega }\left(\Delta ,\gamma \right)\equiv S_{f}^{\omega }\left(x,\tau \right)$, $T_{f}^{q}\left(\Delta ,\gamma \right)\equiv T_{f}^{q}\left(x\right)$ and $T_{f}^{\omega }\left(\Delta ,\gamma \right)\equiv T_{f}^{\omega }\left(x,\tau \right)$, and $U_{f}^{q}\left(\Delta ,\gamma \right)\equiv U_{f}^{q}\left(x\right)$ and $U_{f}^{\omega }\left(\Delta ,\gamma \right)\equiv U_{f}^{\omega }\left(x,\tau \right)$, when we move from *x* and τ to Δ and γ, meaning that *x* and τ are regarded as functions of Δ and γ. This is due to the fact that (Δ,γ) and (x,τ) label the same point in the control parameter space.

If fidelity entropy $S_{f}\left(\Delta ,\gamma \right)$, fidelity temperature $T_{f}\left(\Delta ,\gamma \right)$, and fidelity internal energy $U_{f}\left(\Delta ,\gamma \right)$ are determined in regime I, regime II, regime III, and regime IV, then fidelity mechanical-state functions in the non-principal regimes are determined from dualities in Appendix C by taking into account a contribution from scaling entropy arising from dualities (cf. Appendix G).

Numerical simulations for fidelity entropy $S_{f}\left(\Delta ,\gamma \right)$, fidelity temperature $T_{f}\left(\Delta ,\gamma \right)$, and fidelity internal energy $U_{f}\left(\Delta ,\gamma \right)$ are shown in Figure 23a–c, respectively.

### Appendix O.2. Fidelity Entropy $S_{f}\left(\Delta ,0\right)$, Fidelity Temperature $T_{f}\left(\Delta ,0\right)$, and Fidelity Internal Energy $U_{f}\left(\Delta ,0\right)$: An Interior Point of View

We start from the complete line of critical points (γ=0 with $-1<\Delta <\Delta_{c1}$), which has been labelled as scenario III-1 in Section 6. Here, a dominant control parameter x is chosen to be $x=\Delta_{c1}-\Delta$. From Equation (9), fidelity entropy S(x) takes the following form

(A168) $$S\left(x\right)=-2\int_{x_{c}}^{x}\ln d(x;y)\;dy+S_{0}.$$

Here, d(x;y) denotes the ground-state fidelity per lattice site on the complete line, labelled as scenario III-1, and $S_{0}$ is the residual fidelity entropy at the KT transition point $x_{c}=0$. According to our convention (cf. Section 2), we have $S_{f}\left(x\right)=S\left(x\right)$.

On the complete line (γ=0 with $-1<\Delta <\Delta_{c1}$), which constitutes a principal part as a whole (cf. scenario III-1), the ground-state energy density e(x) monotonically increases with x. Then, from Equation (10), fidelity internal energy U(x) takes the following form

(A169) $$U\left(x\right)=-\ln\frac{e(x)}{e(0)}V\left(x\right)+U_{0}.$$

Here, $U_{0}$ is an additive constant, and V(x)>0 satisfies the singular first-order differential equation:

(A170) $$\frac{\partial V(x)}{\partial x}=\alpha \left(x\right)\;V\left(x\right),$$

with

(A171) $$\alpha \left(x\right)=\frac{\partial \ln\left(e(x)/e(0)\right)/\partial x}{\partial S(x)/\partial x-\ln\left(e(x)/e(0)\right)}.$$

Accordingly, fidelity temperature T(x) on the complete line is provided by the following

(A172) $$T\left(x\right)=-\frac{\partial V(x)}{\partial x}.$$

To solve the singular first-order differential equation, Equation (A123), we analyze the scaling behavior of α(x) in the vicinity of the KT transition point $x_{c}=0$. As it turns out, fidelity entropy S(x) scales as $S\left(x\right)∼x^{3}$. In addition, our numerical simulation shows that the ground-state energy density e(x), near the KT transition point $x_{c}=0$, scales as ln(e(x)/e(0))∼x. Then, α(x) scales as follows

(A173) $$\alpha \left(x\right)\propto \frac{1}{x}.$$

Our numerical simulations confirm this scaling analysis.

Since the integration of α(x) with respect to x is finite, the singular first-order differential equation, Equation (A170), may be solved in a straightforward manner:

(A174) $$V\left(x\right)=V_{0}V_{1}\left(x\right),$$

where $V_{0}$ is a positive constant, and $V_{1}\left(x\right)$ takes the following form

(A175) $$V_{1}\left(x\right)=\exp\left(\int_{0}^{x}\alpha (y)dy\right).$$

The remaining task is to determine $S_{0}$, $U_{0}$, and $V_{0}$. Here, we require that, at the FM transition point $x=1+\Delta_{c1}$, or equivalently Δ=−1, fidelity mechanical-state functions are equal to those from an exterior point of view. In addition, at the KT transition point x=0, or equivalently $\Delta =\Delta_{c1}$, fidelity internal energy must be zero. Hence, $S_{0}$ is determined from the following requirement: $S_{f}\left(1\right)=S_{m}$, with $S_{m}$ denoting fidelity entropy from an exterior point of view at the FM transition point x=1. That is, fidelity entropy $S_{f}\left(x\right)$ from an interior point of view matches that from an exterior point of view at the FM transition point, so it is single-valued there. In addition, fidelity temperature at the FM transition point must be zero, thus leading to $T_{0}=T\left(1\right)$. Furthermore, fidelity internal energy $U_{f}\left(x\right)$ at the KT transition point x=0 must be zero, and fidelity internal energy $U_{f}\left(x\right)$ is single-valued at the FM transition point x=1, as discussed in Section 2: $U_{0}=T_{0}S_{0}$ and $U\left(1\right)-T_{0}S\left(1\right)=U_{m}$, with $U_{m}$ being fidelity internal energy from an exterior point of view at the FM transition point. Hence, $V_{0}$ is determined as follows

(A176) $$V_{0}=\frac{U_{m}}{\ln\left(e(1)/e(0)\right)V_{1}\left(1\right)+\alpha \left(1\right)V_{1}\left(1\right)\left(S_{f}\left(1\right)-S_{0}\right)}.$$

After $V_{0}$ and $U_{0}$ are determined, fidelity temperature T(x) and fidelity internal energy U(x) follow from (A169) and (A172), respectively. We refer to $T\left(x\right)-T_{0}$ and $U\left(x\right)-T_{0}S\left(x\right)$ as $T_{f}\left(x\right)$ and $U_{f}\left(x\right)$, respectively. That is, $T_{f}\left(x\right)\equiv T\left(x\right)-T_{0}$ and $U_{f}\left(x\right)\equiv U\left(x\right)-T_{0}S\left(x\right)$.

Once fidelity entropy $S_{f}\left(x\right)$, fidelity temperature $T_{f}\left(x\right)$ and fidelity internal energy $U_{f}\left(x\right)$ are determined on the complete line, fidelity entropy $S_{f}\left(\Delta ,0\right)$, fidelity temperature $T_{f}\left(\Delta ,0\right)$ and fidelity internal energy $U_{f}\left(\Delta ,0\right)$ follows. That is, $S_{f}\left(\Delta ,0\right)\equiv S_{f}\left(x\right)$, $T_{f}\left(\Delta ,0\right)\equiv T_{f}\left(x\right)$, and $U_{f}\left(\Delta ,0\right)\equiv U_{f}\left(x\right)$, when we move from x to Δ, meaning that x is regarded as a function of Δ. This is due to the fact that (Δ,0) ($-1<\Delta <\Delta_{c1}$) and x label the same point on the complete line.

Numerical simulations for fidelity entropy $S_{f}\left(\Delta ,0\right)$, fidelity temperature $T_{f}\left(\Delta ,0\right)$, and fidelity internal energy $U_{f}\left(\Delta ,0\right)$ are shown in Figure 24a–c, respectively.

### Appendix O.3. Fidelity Entropy $S_{f}\left(D\right)$, Fidelity Temperature $T_{f}\left(D\right)$, and Fidelity Internal Energy $U_{f}\left(D\right)$ for the Spin-1 Bilinear-Biquadratic Model

The spin-1 bilinear–biquadratic model is described by the following Hamiltonian:

(A177) $$H\left(D\right)=\sum_{i}\left(S_{i}\cdot S_{i+1}+D{\left(S_{i}\cdot S_{i+1}\right)}^{2}\right),$$

where $S_{i}=\left(S_{i}^{x},S_{i}^{y},S_{i}^{z}\right)$, and $S_{i}^{x}$, $S_{i}^{y}$, and $S_{i}^{z}$ represent the spin-1 matrices at site i.

As argued in Ref. [231], the AKLT point is characterized by the onset of short-range incommensurate spin correlations in the spin-1 bilinear–biquadratic model. In addition, there is a hidden $Z_{2}\times Z_{2}$ symmetry-breaking order in the Haldane phase [160,161], which results in a four-fold degenerate ground states after a non-local unitary transformation [160,161]. Therefore, it is plausible to regard the AKLT point as a characteristic point, which is identified as a stable fixed point in the Haldane phase. Here, we remark that the spin-1 bilinear–biquadratic model constitutes the SU(2) characteristic line, if we embed both the Hamiltonian (5) and the Hamiltonian (A177) into a more general model, which includes an anisotropic extension of the biquadratic interactions. As a consequence, the AF spin-1 Heisenberg model turns out to be a metastable fixed point. This means that we have to determine fidelity mechanical-state functions for the AF spin-1 Heisenberg model via the spin-1 bilinear–biquadratic model.

In this Appendix, we present mathematical details about fidelity entropy $S_{f}\left(D\right)$, fidelity temperature $T_{f}\left(D\right)$, and fidelity internal energy $U_{f}\left(D\right)$ from the Babujian–Takhtajan critical point $D_{c}=-1$ [152,153] to the AKLT point $D_{\mathrm{AKLT}}=1/3$ [133,134].

We choose a dominant control parameter x to be x=D+1. From Equation (9), fidelity entropy S(x) takes the following form

(A178) $$S\left(x\right)=-2\int_{x_{c}}^{x}\ln d(x;y)\;dy+S_{0}.$$

Here, d(x;y) denotes the ground-state fidelity per lattice site, and $S_{0}$ is the residual fidelity entropy at the critical point $x_{c}=0$. According to our convention (cf. Section 2), we have $S_{f}\left(x\right)=S\left(x\right)$.

Once fidelity entropy $S_{f}\left(x\right)$ is determined, fidelity temperature $T_{f}\left(x\right)$ and fidelity internal energy $U_{f}\left(x\right)$ may be determined from solving the singular first-order differential Equation (14), as discussed for continuous QPTs in Section 2. Here, the ground-state energy density e(x) monotonically increases with x. Then, from Equation (10), fidelity internal energy U(x) takes the following form

(A179) $$U\left(x\right)=-\ln\frac{e(x)}{e(0)}V\left(x\right)+U_{0}.$$

Here, $U_{0}$ is an additive constant, and V(x)>0 satisfies the singular first-order differential equation

(A180) $$\frac{\partial V(x)}{\partial x}=\alpha \left(x\right)\;V\left(x\right),$$

with

(A181) $$\alpha \left(x\right)=\frac{\partial \ln\left(e(x)/e(0)\right)/\partial x}{\partial S(x)/\partial x-\ln\left(e(x)/e(0)\right)}.$$

Accordingly, fidelity temperature T(x) is given by the following

(A182) $$T\left(x\right)=-\frac{\partial V(x)}{\partial x}.$$

To solve the singular first-order differential equation, Equation (A123), we analyze the scaling behavior of α(x) near point $x_{c}=0$. Fidelity entropy S(x) scales as $S\left(x\right)∼x^{3}$. In addition, our numerical simulation shows that the ground-state energy density e(x) near point $x_{c}=0$ scales as ln(e(x)/e(0))∼x [232]. Then, α(x) diverges as follows

(A183) $$\alpha \left(x\right)\propto \frac{1}{x}.$$

Our numerics confirm this scaling analysis.

Since the integration of α(x) with respect to x is finite, the singular first-order differential equation, Equation (A123), may be solved in a straightforward manner:

(A184) $$V\left(x\right)=V_{0}\left(x\right)V_{1}\left(x\right),$$

where $V_{0}\left(x\right)$ is a function of x, and $V_{1}\left(x\right)$ is defined as follows

(A185) $$V_{1}\left(x\right)=\exp\left(\int_{0}^{x}\alpha (y)dy\right).$$

A shift in fidelity temperature is performed: $T\left(x\right)-T_{0}$, accompanied by a shift in fidelity internal energy: $U\left(x\right)-T_{0}S\left(x\right)$. We refer to $T\left(x\right)-T_{0}$ and $U\left(x\right)-T_{0}S\left(x\right)$ as $T_{f}\left(x\right)$ and $U_{f}\left(x\right)$, respectively. That is, $T_{f}\left(x\right)\equiv T\left(x\right)-T_{0}$ and $U_{f}\left(x\right)\equiv U\left(x\right)-T_{0}S\left(x\right)$.

The continuity requirement for fidelity entropy demands that $S\left(1\right)=S_{H}$, where S(1) is fidelity entropy for the spin-1 bilinear–biquadratic model (A177) with x=1, or equivalently, D=0, and $S_{H}$ is fidelity entropy at the metastable fixed point (1, 0) on the (Δ, γ) plane for the quantum spin-1 XYZ model (5). We emphasize that fidelity entropy $S_{f}\left(\Delta ,\gamma \right)$ is not single-valued at the metastable fixed point (1, 0) for the quantum spin-1 XYZ model (5) (cf. Section 6). Here, we choose $S_{H}$ to represent fidelity entropy at the metastable fixed point (1, 0), evaluated from the U(1)-symmetric line (γ=0 with $\Delta_{c1}<\Delta <1$), labelled as i. Therefore, the residual fidelity entropy $S_{0}$ at the Babujian–Takhtajan critical point is determined. In order to ensure that fidelity temperature is zero at the AKLT point, with x=4/3, T(x) is shifted to $T\left(x\right)-T_{0}$, accompanied by a shift in U(x): $U\left(x\right)-T_{0}S\left(x\right)$, with S(x) left intact. In addition, fidelity internal energy U(x) at a critical point $x_{c}$, with $x_{c}=0$, must be zero, and fidelity internal energy U(x) at the AKLT point, with x=4/3, takes the maximum value $U_{m}$, as discussed in Section 2: $U_{0}=T_{0}S_{0}$ and $U\left(4/3\right)-T_{0}S\left(4/3\right)=U_{m}$. Here, $U_{m}$ is identical to fidelity internal energy at the stable fixed point for the quantum spin-1 XYZ model (5). Therefore, $V_{0}$ is determined as follows

(A186) $$V_{0}=\frac{U_{m}}{-\ln\left(e(4/3)/e(0)\right)V_{1}\left(4/3\right)+\alpha \left(4/3\right)V_{1}\left(4/3\right)\left(S_{f}\left(4/3\right)-S_{0}\right)}.$$

After $V_{0}$ and $U_{0}$ are determined, fidelity temperature T(x) and fidelity internal energy U(x) follow from (A179) and (A182), respectively. We refer to $T\left(x\right)-T_{0}$ and $U\left(x\right)-T_{0}S\left(x\right)$ as $T_{f}\left(x\right)$ and $U_{f}\left(x\right)$, respectively. That is, $T_{f}\left(x\right)\equiv T\left(x\right)-T_{0}$ and $U_{f}\left(x\right)\equiv U\left(x\right)-T_{0}S\left(x\right)$.

Once fidelity entropy $S_{f}\left(x\right)$, fidelity temperature $T_{f}\left(x\right)$, and fidelity internal energy $U_{f}\left(x\right)$ are determined, fidelity entropy $S_{f}\left(D\right)$, fidelity temperature $T_{f}\left(D\right)$, and fidelity internal energy $U_{f}\left(D\right)$ follow. That is, $S_{f}\left(D\right)\equiv S_{f}\left(x\right)$, $T_{f}\left(D\right)\equiv T_{f}\left(x\right)$, and $U_{f}\left(D\right)\equiv U_{f}\left(x\right)$, when we move from x to D, meaning that x is regarded as a function of D. This is due to the fact that D and x label the same point on the SU(2)-symmetric line.

Our numerical simulation results for fidelity entropy $S_{f}\left(D\right)$, fidelity temperature $T_{f}\left(D\right)$, and fidelity internal energy $U_{f}\left(D\right)$, with $D_{c}<D<D_{\mathrm{AKLT}}$, are shown in Figure A13a–c, respectively.

## Appendix P. Fidelity Entropy $S_{f}\left(J_{x},J_{y}\right)$, Fidelity Temperature $T_{f}\left(J_{x},J_{y}\right)$, and Fidelity Internal Energy $U_{f}\left(J_{x},J_{y}\right)$ for the Spin-1/2 Kitaev Model on a Honeycomb Lattice

In this Appendix, we present mathematical details about fidelity entropy $S_{f}\left(J_{x},J_{y}\right)$, fidelity temperature $T_{f}\left(J_{x},J_{y}\right)$, and fidelity internal energy $U_{f}\left(J_{x},J_{y}\right)$ for the spin-1/2 Kitaev model on a honeycomb lattice. Here, we set $J_{z}$ as an energy scale, $J_{z}=1$, for the sake of brevity.

We restrict ourselves to the region $J_{x}\geq 0$ and $J_{y}\geq 0$. As shown in Figure 25, there are four distinct phases: three gapped $Z_{2}$ quantum spin liquid phases, and one gapless $Z_{2}$ spin liquid phase. There are three lines of critical points ($J_{y}=1+J_{x}$ with $J_{x}\geq 0$, $J_{y}=1-J_{x}$ with $0\leq J_{x}\leq 1$, and $J_{y}=-1+J_{x}$ with $J_{x}>1$), that are dual to each other. A remarkable feature of the spin-1/2 Kitaev model on a honeycomb lattice is that there are three different dualities (cf. Appendix C).

Taking the symmetries and dualities into account, we may divide the region, defined by $J_{x}\geq 0$ and $J_{y}\geq 0$, into twelve distinct regimes, enclosed by the boundaries consisting of the $Z_{2}$-symmetric line ($J_{x}=J_{y}$), the two self-dual lines ($J_{x}=1$ and $J_{y}=1$), and the two semi-self dual lines ($J_{x}=0$ and $J_{y}=0$), together with the three lines of critical points. The twelve regimes are separated into two groups, with six regimes in each group that are dual to each other. As shown in Section 2, the first group includes regime I, regime III, regime V, regime VII, regime IX, and regime XI, whereas the second group includes regime II, regime IV, regime VI, regime VIII, regime X, and regime XII. Therefore, there are only two principal regimes representing the physics underlying the model. Here, we chose regime I ($0<J_{x}<1/2$ and $J_{x}<J_{y}<1-J_{x}$) and regime II ($0<J_{x}<1$, $1-J_{x}<J_{y}<1$ and $J_{y}>J_{x}$) as the two principal regimes. Hence, all other regimes are symmetric or dual image regimes.

Now, we turn to the explicit mathematical expressions for fidelity entropy $S_{f}\left(J_{x},J_{y}\right)$, fidelity temperature $T_{f}\left(J_{x},J_{y}\right)$, and fidelity internal energy $U_{f}\left(J_{x},J_{y}\right)$ for the spin-1/2 Kitaev model on a honeycomb lattice. We recall the choices of a dominant control parameter x in the two principal regimes:

(i) Regime I ($0<J_{x}<1/2$ and $J_{x}<J_{y}<1-J_{x}$): A dominant control parameter x was chosen to be $x=\sqrt{J_{x}^{2}+J_{y}^{2}}\left(1-J_{x}-J_{y}\right)/\left(J_{x}+J_{y}\right)$, and an auxiliary control parameter τ was chosen to be $\tau =J_{y}/J_{x}\in \left(1,\infty \right)$.
(ii) Regime II ($0<J_{x}<1$, $1-J_{x}<J_{y}<1$ and $J_{y}>J_{x}$): A dominant control parameter x was chosen to be $x=\sqrt{{\left(J_{x}-1\right)}^{2}+{\left(J_{y}-1\right)}^{2}}\left(J_{x}+J_{y}-1\right)/\left(2-J_{x}-J_{y}\right)$, and an auxiliary control parameter τ was chosen to be $\tau =\left(J_{y}-1\right)/\left(J_{x}-1\right)\in \left(0,1\right)$.

### Appendix P.1. Fidelity Entropy $S_{f}$ ($J_{x}$, $J_{y}$), Fidelity Temperature $T_{f}$ ($J_{x}$, $J_{y}$), and Fidelity Internal Energy $U_{f}$ ($J_{x}$, $J_{y}$) for the Spin-1/2 Kitaev Model on a Honeycomb Lattice: An Exterior Point of View

Let us determine fidelity entropy $S^{i}\left(x\right)$ in the principal part on the $Z_{2}$-symmetric line ($J_{x}=0$ with $0<J_{y}<1$), labelled as i. We recall that a dominant control parameter x was chosen to be $x=1-J_{y}$. As follows from (9), fidelity entropy $S^{i}\left(x\right)$ takes the following form

(A187) $$S^{i}\left(x\right)=-2\int_{x_{c}}^{x}\ln d^{i}\left(x;y\right)\;dy+S_{0}^{i}.$$

Here, $d^{i}\left(x;y\right)$ denotes the ground-state fidelity per lattice site in the principal part, and $S_{0}^{i}$ is the residual fidelity entropy at the critical point $x_{c}=0$ between the gapped $Z_{2}$ spin liquid phases. According to our convention (cf. Section 2), we have $S_{f}^{i}\left(x\right)=S^{i}\left(x\right)$.

Now we move to fidelity entropy $S_{f}\left(J_{x},J_{y}\right)$ in two principal regimes.

(a) In regime I ($0<J_{x}<1/2$ and $J_{x}<J_{y}<1-J_{x}$), for the chosen dominant control parameter x: $x=\sqrt{J_{x}^{2}+J_{y}^{2}}\left(1-J_{x}-J_{y}\right)/\left(J_{x}+J_{y}\right)$, fidelity entropy $S^{I}\left(x,\tau \right)$ takes the following form

(A188) $$S^{I}\left(x,\tau \right)=-2\int_{x_{c}}^{x}\ln d^{I}\left(x,\tau ;y,\tau \right)\;dy+S_{0}^{I}\left(\tau \right).$$

Here, $d^{I}\left(x,\tau ;y,\tau \right)$ denotes the ground-state fidelity per lattice site in regime I, and $S_{0}^{I}\left(\tau \right)$ is the residual fidelity entropy at a critical point $x_{c}$ for a fixed τ, with $x_{c}=0$. According to our convention (cf. Section 2), we have $S_{f}^{I}\left(x,\tau \right)=S^{I}\left(x,\tau \right)$.

(b) In regime II ($0<J_{x}<1$, $1-J_{x}<J_{y}<1$ and $J_{y}>J_{x}$), for the chosen dominant control parameter x: $x=\sqrt{{\left(J_{x}-1\right)}^{2}+{\left(J_{y}-1\right)}^{2}}\left(J_{x}+J_{y}-1\right)/\left(2-J_{x}-J_{y}\right)$, fidelity entropy $S^{\mathrm{II}}\left(x,\tau \right)$, as follows from Equation (28), takes the same form as Equation (A188) for regime I, with the label being changed from I to II. According to our convention (cf. Section 2), we have $S_{f}^{\mathrm{II}}\left(x,\tau \right)=S^{\mathrm{II}}\left(x,\tau \right)$.

Once fidelity entropy $S_{f}^{i}\left(x\right)$ in principal part i and fidelity entropy $S_{f}^{\omega }\left(x,\tau \right)$ (ω = I and II) in the two principal regimes are determined, fidelity temperature $T_{f}^{i}\left(x\right)$ and fidelity internal energy $U_{f}^{i}\left(x\right)$ in principal part i may be determined from solving the singular first-order differential equation, Equation (14), and fidelity temperature $T_{f}^{\omega }\left(x,\tau \right)$ and fidelity internal energy $U_{f}^{\omega }\left(x,\tau \right)$ in the principal regime ω may be determined from solving the singular first-order differential equation, Equation (31), according to our prescription for continuous QPTs in Section 2.

We start our discussions from fidelity temperature and fidelity internal energy in principal part i on the $Z_{2}$-symmetric line ($J_{x}=0$ with $0<J_{y}<1$). For the chosen dominant control parameter $x=1-J_{y}$, the ground-state energy density $e^{i}\left(x\right)$ increases with x. Thus, fidelity internal energy $U^{i}\left(x\right)$ takes the following form

(A189) $$U^{i}\left(x\right)=-\ln\frac{e^{i}\left(x\right)}{e^{i}\left(0\right)}V^{i}\left(x\right)+U_{0}^{i}.$$

Here, $U_{0}^{i}$ is an additive constant, and $V^{i}\left(x\right)>0$ satisfies the singular first-order differential equation:

(A190) $$\partial V^{i}\left(x\right)/\partial x=\alpha^{i}\left(x\right)\;V^{i}\left(x\right),$$

with

(A191) $$\alpha^{i}\left(x\right)=\frac{\partial \ln\left(e^{i}\left(x\right)/e^{i}\left(0\right)\right)/\partial x}{\partial S^{i}\left(x\right)/\partial x-\ln\left(e^{i}\left(x\right)/e^{i}\left(0\right)\right)}.$$

Accordingly, fidelity temperature $T^{i}\left(x\right)$ in this part follows from

(A192) $$T^{i}\left(x\right)=-\frac{\partial V^{i}\left(x\right)}{\partial x}.$$

Now, we move to the two principal regimes: regime I and regime II.

(a) In regime I ($0<J_{x}<1$ and $J_{x}<J_{y}<1-J_{x}$), for a fixed $\tau =J_{y}/J_{x}$, the ground-state energy density $e^{I}\left(x,\tau \right)$ monotonically increases with x, with $x=\sqrt{J_{x}^{2}+J_{y}^{2}}$$\left(1-J_{x}-J_{y}\right)/\left(J_{x}+J_{y}\right)$. Thus, fidelity internal energy $U^{I}\left(x,\tau \right)$ takes the following form

(A193) $$U^{I}\left(x,\tau \right)=-\ln\frac{e^{I}\left(x,\tau \right)}{e^{I}\left(0,\tau \right)}V^{I}\left(x,\tau \right)+U_{0}^{I}\left(\tau \right).$$

Here, $U_{0}^{I}\left(\tau \right)$ is a function of τ, and $V^{I}\left(x,\tau \right)>0$ satisfies the singular first-order differential equation

(A194) $$\frac{\partial V^{I}\left(x,\tau \right)}{\partial x}=\alpha^{I}\left(x,\tau \right)\;V^{I}\left(x,\tau \right),$$

with

(A195) $$\alpha^{I}\left(x,\tau \right)=\frac{\partial \ln\left(e^{I}\left(x,\tau \right)/e^{I}\left(0,\tau \right)\right)/\partial x}{\partial S^{I}\left(x,\tau \right)/\partial x-\ln\left(e^{I}\left(x,\tau \right)/e^{I}\left(0,\tau \right)\right)}.$$

Accordingly, fidelity temperature $T^{I}\left(x,\tau \right)$ in this regime is given by the following

(A196) $$T^{I}\left(x,\tau \right)=-\frac{\partial V^{I}\left(x,\tau \right)}{\partial x}.$$

(b) In regime II ($0<J_{x}<1$, $1-J_{x}<J_{y}<1$, and $J_{y}>J_{x}$), for a fixed $\tau =(J_{y}-1)/$$\left(J_{x}-1\right)\in \left(0,1\right)$, the ground-state energy density $e^{\mathrm{II}}\left(x,\tau \right)$ monotonically decreases with x, with $x=\sqrt{{\left(J_{x}-1\right)}^{2}+{\left(J_{y}-1\right)}^{2}}\left(J_{x}+J_{y}-1\right)/\left(2-J_{x}-J_{y}\right)$. Thus, from Equation (30), fidelity internal energy $U^{\mathrm{II}}\left(x,\tau \right)$ takes the following form

(A197) $$U^{\mathrm{II}}\left(x,\tau \right)=\ln\frac{e^{\mathrm{II}}\left(x,\tau \right)}{e^{\mathrm{II}}\left(0,\tau \right)}V^{\mathrm{II}}\left(x,\tau \right)+U_{0}^{\mathrm{II}}\left(\tau \right).$$

Here, $U_{0}^{\mathrm{II}}\left(\tau \right)$ is a function of τ, and $V^{\mathrm{II}}\left(x,\tau \right)>0$ satisfies the singular first-order differential equation:

(A198) $$\frac{\partial V^{\mathrm{II}}\left(x,\tau \right)}{\partial x}=\alpha^{\mathrm{II}}\left(x,\tau \right)\;V^{\mathrm{II}}\left(x,\tau \right),$$

with

(A199) $$\alpha^{\mathrm{II}}\left(x,\tau \right)=-\frac{\partial \ln\left(e^{\mathrm{II}}\left(x,\tau \right)/e^{\mathrm{II}}\left(0,\tau \right)\right)/\partial x}{\partial S^{\mathrm{II}}\left(x,\tau \right)/\partial x+\ln e^{\mathrm{II}}\left(x,\tau \right)/e\left(0,\tau \right)}.$$

Accordingly, fidelity temperature $T^{\mathrm{II}}\left(x,\tau \right)$ in this regime is provided by the following

(A200) $$T^{\mathrm{II}}\left(x,\tau \right)=-\frac{\partial V^{\mathrm{II}}\left(x,\tau \right)}{\partial x}.$$

To solve the singular first-order differential equations, Equations (A194) and (A198), we analyze the scaling behavior of $\alpha^{I}\left(x,\tau \right)$ and $\alpha^{\mathrm{II}}\left(x,\tau \right)$ in the vicinity of a critical point $x_{c}=0$ for a fixed τ. When a critical point $x_{c}=0$ is approached, fidelity entropy $S^{I}\left(x,\tau \right)$ and $S^{\mathrm{II}}\left(x,\tau \right)$ scale as $S^{I}\left(x,\tau \right)∼x^{5/2}$ and $S^{\mathrm{II}}\left(x,\tau \right)∼x^{5/2}$, respectively, consistent with the fact that d=2, m=1, $\nu_{\|}=1/2$, and $\nu_{⊥}=1$ [227,233]. Here, $\nu_{\|}$ and $\nu_{⊥}$ stand for the critical exponent for the correlation length in two perpendicular directions, with m and d−m being the effective dimensions, respectively (cf. Appendix H). Combined with a scaling analysis of the ground-state energy density $e^{I}\left(x,\tau \right)$ and $e^{\mathrm{II}}\left(x,\tau \right)$ near a critical point $x_{c}=0$: $\ln e^{I}\left(x,\tau \right)∼x$ and $\ln e^{\mathrm{II}}\left(0,\tau \right)∼x$, we have the following:

(A201) $$\alpha^{I}\left(x,\tau \right)∼\frac{1}{x},$$

and

(A202) $$\alpha^{\mathrm{II}}\left(x,\tau \right)∼\frac{1}{x}.$$

This scaling analysis is confirmed in our numerical simulations.

Since the integration of $\alpha^{I}\left(x,\tau \right)$/$\alpha^{\mathrm{II}}\left(x,\tau \right)$ with respect to x is finite, the singular first-order differential equation, Equation (A194), for regime I and regime II may be solved in a straightforward manner.

(a) In regime I ($0<J_{x}<1$ and $J_{x}<J_{y}<1-J_{x}$), for a fixed τ, the singular first-order differential equation, Equation (A194), may be solved as follows:

(A203) $$V^{V}\left(x,\tau \right)=V_{0}^{V}\left(\tau \right)V_{1}^{V}\left(x,\tau \right),$$

where $V_{0}^{I}\left(\tau \right)$ is a function of τ, and $V_{1}^{I}\left(x,\tau \right)$ is defined as follows

(A204) $$V_{1}^{I}\left(x,\tau \right)=\exp\left(\int_{0}^{x}\alpha^{I}\left(y,\tau \right)dy\right),$$

(b) In regime II, the solution $V^{\mathrm{II}}\left(x,\tau \right)$ to the singular first-order differential equation takes the same form as Equations (A203) and (A204) for regime I, with the label being changed from I to II.

We dictate that fidelity entropy should be zero at the critical point $x=x_{c}$, with $x_{c}=0$ or equivalently, $J_{x}=0$ and $J_{y}=1$, in principal part i. That is, $S_{0}^{i}=0$. As argued in Section 2, we need to determine $T_{0}^{i}$ for principal part i. Fidelity temperature at the U(1)-symmetric point x=1, or equivalently $J_{x}=J_{y}=0$, in principal part i is zero. Then, we have $T_{0}^{i}\equiv T^{i}\left(1\right)$.

We are free to choose $V_{0}$ on one of the characteristic lines, since fidelity internal energy $U_{f}\left(x,\tau \right)$ is only determined up to a constant factor. Here, we set $V_{0}^{i}=1$ for the $Z_{2}$-symmetric line ($J_{x}=0$ with $0<J_{y}<1$), labelled as i. Then, fidelity internal energy $U^{i}\left(x\right)$ in part i is determined from (A189). A shift from $T^{i}\left(x\right)$ to $T^{i}\left(x\right)-T_{0}^{i}$ induces a shift in fidelity internal energy in part i: $U^{i}\left(x\right)-T_{0}^{i}S^{i}\left(x\right)$. We refer to $T^{i}\left(x\right)-T_{0}^{i}$ and $U^{i}\left(x\right)-T_{0}^{i}S^{i}\left(x\right)$ as $T_{f}^{i}\left(x\right)$ and $U_{f}^{i}\left(x\right)$, respectively. That is, $T_{f}^{i}\left(x\right)\equiv T^{i}\left(x\right)-T_{0}^{i}$ and $U_{f}^{i}\left(x\right)\equiv U^{i}\left(x\right)-T_{0}^{i}S^{i}\left(x\right)$.

Now we turn to the residual fidelity entropy $S_{0}^{I}\left(\tau \right)$ on the line of critical points ($J_{y}$$=1-J_{x}$ with $0<J_{x}<1/2$). $S_{0}^{I}\left(\tau \right)$ is determined from the continuity requirement for fidelity entropy $S_{f}^{I}\left(x,\tau \right)$ at a characteristic point ($x_{0},\;\tau$), with $x_{0}=\sqrt{1+\tau^{2}}/\left(1+\tau \right)$, which turns out to be the U(1)-symmetric point ($J_{x}=0$ and $J_{y}=0$) for any τ: $S_{0}^{I}\left(\tau \right)=S^{i}\left(1\right)-S^{I}\left(x_{0},\tau \right)$. In addition, the continuity requirement for fidelity entropy demands that the residual fidelity entropy $S_{0}^{\mathrm{II}}\left(\tau \right)$ is identical to the residual fidelity entropy $S_{0}^{I}\left(\tau \right)$ on the line of critical points ($J_{y}=1-J_{x}$ with $0\leq J_{x}\leq 1$). That is, we have $S_{0}^{\mathrm{II}}\left(\tau \right)=S_{0}^{I}\left(1/\tau \right)$. With this in mind, $S_{f}^{I}\left(x,\tau \right)$ and $S_{f}^{\mathrm{II}}\left(x,\tau \right)$ are determined in the two principal regimes, respectively.

The remaining task is to ensure the continuity requirements for fidelity temperature and fidelity internal energy. To this end, we need to determine $T_{0}^{\omega }\equiv T_{m}^{\omega }-T_{t}^{\omega }$ for the two principal regimes, with ω=I and II. Specifically, in regime I, for a fixed τ, we have $T_{m}^{I}\left(\tau \right)=T^{I}\left(x_{0},\tau \right)$ and $T_{t}^{I}\left(\tau \right)=0$. Here, $T^{I}\left(x_{0},\tau \right)$ denotes fidelity temperature, evaluated from a dominant control parameter x for a fixed τ in regime I, at $\left(x_{0},\tau \right)$ or equivalently $J_{x}=0$ and $J_{y}=0$. In regime II, $T^{\mathrm{II}}\left(x_{s},\tau \right)$ denotes fidelity temperature, evaluated from a dominant control parameter x for a fixed τ in principal regime II, at a characteristic point $\left(x_{s},\tau \right)$, with $x_{s}=\sqrt{1+\tau^{2}}/\left(1+\tau \right)$, which turns out to be the $S_{3}$-symmetric point, located at $J_{x}=1$ and $J_{y}=1$, for any τ. Then, we have $T_{m}^{\mathrm{II}}\left(\tau \right)=T^{\mathrm{II}}\left(x_{s},\tau \right)$ and $T_{t}^{\mathrm{II}}\left(\tau \right)=0$.

In regime I ($0<J_{x}<1/2$ and $J_{x}<J_{y}<1-J_{x}$), in order to ensure the continuity requirement for fidelity temperature at the U(1)-symmetric point $\left(x_{0},\tau \right)$ for a fixed τ, or equivalently $J_{x}=0$ and $J_{y}=0$, $T^{I}\left(x,\tau \right)$ is shifted to $T^{I}\left(x,\tau \right)-T_{0}^{I}\left(\tau \right)$, with $T_{0}^{I}\left(\tau \right)=T^{I}\left(x_{0},\tau \right)$. Then, $U^{I}\left(x,\tau \right)$ is shifted to $U^{I}\left(x,\tau \right)-T_{0}^{I}\left(\tau \right)S^{I}\left(x,\tau \right)$, with $S^{I}\left(x,\tau \right)$ left intact. In addition, fidelity internal energy $U^{I}\left(x,\tau \right)$ on the line of critical points (0,τ) must be zero, and fidelity internal energy $U^{I}\left(x,\tau \right)$ satisfies the continuity requirement at the U(1)-symmetric point $\left(x_{0},\tau \right)$ or equivalently $J_{x}=0$ and $J_{y}=0$, as discussed in Section 2: $U_{0}^{I}\left(\tau \right)=T_{0}^{I}\left(\tau \right)S_{0}^{I}\left(\tau \right)$ and $U^{I}\left(x_{0},\tau \right)-T_{0}^{I}\left(\tau \right)S^{I}\left(x_{0},\tau \right)=U_{f}^{i}\left(1\right)$. Therefore, $V_{0}^{I}\left(\tau \right)$ is determined as follows

(A205) $$V_{0}^{I}\left(\tau \right)=\frac{U_{f}^{i}\left(1\right)}{-\ln e^{I}\left(x_{0},\tau \right)/e^{I}\left(0,\tau \right)V_{1}^{I}\left(x_{0},\tau \right)+\alpha^{I}\left(x_{0},\tau \right)V_{1}^{I}\left(x_{0},\tau \right)\left(S^{I}\left(x_{0},\tau \right)-S_{0}^{I}\left(\tau \right)\right)}.$$

After $V_{0}^{I}\left(\tau \right)$ and $U_{0}^{I}\left(\tau \right)$ are determined, fidelity temperature $T^{I}\left(x,\tau \right)$ and fidelity internal energy $U^{I}\left(x,\tau \right)$ follow from (A193) and (A196), respectively. Following from our discussions in Section 2, fidelity entropy $S_{f}^{I}\left(x,\tau \right)$, fidelity temperature $T_{f}^{I}\left(x,\tau \right)$, and fidelity internal energy $U_{f}^{I}\left(x,\tau \right)$ take the following form: $S_{f}^{I}\left(x,\tau \right)=S^{I}\left(x,\tau \right)$, $T_{f}^{I}\left(x,\tau \right)=T^{I}\left(x,\tau \right)-T_{0}^{I}\left(\tau \right)$, and $U_{f}^{I}\left(x,\tau \right)=U^{I}\left(x,\tau \right)-T_{0}^{I}\left(\tau \right)S^{I}\left(x,\tau \right)$, respectively.

In regime II ($0<J_{x}<1$, $1-J_{x}<J_{y}<1$ and $J_{y}>J_{x}$), in order to ensure that fidelity temperature vanishes at the $S_{3}$-symmetric point $\left(x_{s},\tau \right)$ for a fixed τ, or equivalently $J_{x}=1$ and $J_{y}=1$, $T^{\mathrm{II}}\left(x,\tau \right)$ is shifted to $T^{\mathrm{II}}\left(x,\tau \right)-T_{0}^{\mathrm{II}}\left(\tau \right)$, with $T_{0}^{\mathrm{II}}\left(\tau \right)=T^{\mathrm{II}}\left(x_{s},\tau \right)$. Then, $U^{\mathrm{II}}\left(x,\tau \right)$ is shifted to $U^{\mathrm{II}}\left(x,\tau \right)-T_{0}^{\mathrm{II}}\left(\tau \right)S^{\mathrm{II}}\left(x,\tau \right)$, with $S^{\mathrm{II}}\left(x,\tau \right)$ left intact. In addition, fidelity internal energy $U^{\mathrm{II}}\left(x,\tau \right)$ on the line of critical points (0,τ) must be zero, and fidelity internal energy $U^{\mathrm{II}}\left(J_{x},J_{y}\right)$ satisfies the continuity requirement at the $S_{3}$-symmetric point $\left(x_{s},\tau \right)$, or equivalently, $J_{x}=1$ and $J_{y}=1$, as discussed in Section 2: $U_{0}^{\mathrm{II}}\left(\tau \right)=T_{0}^{\mathrm{II}}\left(\tau \right)S_{0}^{\mathrm{II}}\left(\tau \right)$ and $U^{\mathrm{II}}\left(x_{0},\tau \right)-T_{0}^{\mathrm{II}}\left(\tau \right)S^{\mathrm{II}}\left(x_{0},\tau \right)=U_{f}^{i}\left(1\right)$. Therefore, $V_{0}^{\mathrm{II}}\left(\tau \right)$ is determined as follows

(A206) $$V_{0}^{\mathrm{II}}\left(\tau \right)=\frac{U_{f}^{i}\left(1\right)}{\ln e^{\mathrm{II}}\left(x_{s},\tau \right)/e^{\mathrm{II}}\left(0,\tau \right)V_{1}^{\mathrm{II}}\left(x_{s},\tau \right)+\alpha^{\mathrm{II}}\left(x_{s},\tau \right)V_{1}^{\mathrm{II}}\left(x_{s},\tau \right)\left(S^{\mathrm{II}}\left(x_{s},\tau \right)-S_{0}^{\mathrm{II}}\left(\tau \right)\right)}.$$

Once $V_{0}^{\mathrm{II}}\left(\tau \right)$ and $U_{0}^{\mathrm{II}}\left(\tau \right)$ are determined, fidelity temperature $T^{\mathrm{II}}\left(x,\tau \right)$ and fidelity internal energy $U^{\mathrm{II}}\left(x,\tau \right)$ follow from their counterparts of (A193) and (A196), with the label being changed from I to II, respectively. Following from our discussions in Section 2, fidelity entropy $S_{f}^{\mathrm{II}}\left(x,\tau \right)$, fidelity temperature $T_{f}^{\mathrm{II}}\left(x,\tau \right)$, and fidelity internal energy $U_{f}^{\mathrm{II}}\left(x,\tau \right)$ take the following form: $S_{f}^{\mathrm{II}}\left(x,\tau \right)=S^{\mathrm{II}}\left(x,\tau \right)$, $T_{f}^{\mathrm{II}}\left(x,\tau \right)=T^{\mathrm{II}}\left(x,\tau \right)-T_{0}^{\mathrm{II}}\left(\tau \right)$, and $U_{f}^{\mathrm{II}}\left(x,\tau \right)=U^{\mathrm{II}}\left(x,\tau \right)-T_{0}^{\mathrm{II}}\left(\tau \right)S^{\mathrm{II}}\left(x,\tau \right)$, respectively.

Once fidelity entropy $S_{f}^{q}\left(x\right)$, fidelity temperature $T_{f}^{i}\left(x\right)$, and fidelity internal energy $U_{f}^{i}\left(x\right)$ on the characteristic line, labelled as i, and fidelity entropy $S_{f}^{\omega }\left(x,\tau \right)$, fidelity temperature $T_{f}^{\omega }\left(x,\tau \right)$ and fidelity internal energy $U_{f}^{\omega }\left(x,\tau \right)$ (ω = I and II) are determined for the two principal regimes, fidelity entropy $S_{f}\left(J_{x},J_{y}\right)$, fidelity temperature $T_{f}\left(J_{x},J_{y}\right)$, and fidelity internal energy $U_{f}\left(J_{x},J_{y}\right)$ follow. That is, $S_{f}^{q}\left(J_{x},J_{y}\right)\equiv S_{f}^{q}\left(x\right)$ and $S_{f}^{\omega }\left(J_{x},J_{y}\right)\equiv S_{f}^{\omega }\left(x,\tau \right)$, $T_{f}^{q}\left(J_{x},J_{y}\right)\equiv T_{f}^{q}\left(x\right)$ and $T_{f}^{\omega }\left(J_{x},J_{y}\right)\equiv T_{f}^{\omega }\left(x,\tau \right)$, and $U_{f}^{q}\left(J_{x},J_{y}\right)\equiv U_{f}^{q}\left(x\right)$ and $U_{f}^{\omega }\left(J_{x},J_{y}\right)\equiv U_{f}^{\omega }\left(x,\tau \right)$, when we move from x and τ to $J_{x}$ and $J_{y}$, meaning that x and τ are regarded as functions of $J_{x}$ and $J_{y}$. This is due to the fact that $\left(J_{x},\;J_{y}\right)$ and (x,τ) label the same point on each characteristic line and in each principal regime.

If fidelity entropy $S_{f}\left(J_{x},J_{y}\right)$, fidelity temperature $T_{f}\left(J_{x},J_{y}\right)$, and fidelity internal energy $U_{f}\left(J_{x},J_{y}\right)$ are determined in regime I and regime II, then fidelity mechanical-state functions in the non-principal regimes are determined from dualities in Appendix C by taking into account a contribution from scaling entropy arising from dualities (cf. Appendix G).

Numerical simulations for fidelity entropy $S_{f}\left(J_{x},J_{y}\right)$, fidelity temperature $T_{f}\left(J_{x},J_{y}\right)$, and fidelity internal energy $U_{f}\left(J_{x},J_{y}\right)$ are shown in Figure 26a–c, respectively.

### Appendix P.2. Fidelity Entropy $S_{f}\left(J_{x},1-J_{x}\right)$, Fidelity Temperature $T_{f}\left(J_{x},1-J_{x}\right)$, and Fidelity Internal Energy $U_{f}\left(J_{x},1-J_{x}\right)$ for the Spin-1/2 Kitaev Model on a Honeycomb Lattice: An Interior Point of View

There are three complete lines of critical points: $J_{y}=1+J_{x}$ with $J_{x}\geq 0$, $J_{y}=1-J_{x}$ with $0\leq J_{x}\leq 1$, and $J_{y}=-1+J_{x}$ with $J_{x}>1$. Since they are dual to each other, we only need to focus on the complete line of critical points ($J_{y}=1-J_{x}$ with $0<J_{x}<1$) labelled as scenario I-3 in Section 6. The complete line is divided into two symmetric parts, with the principal part being the line of critical points ($J_{y}=1-J_{x}$ with $0<J_{x}<1/2$). Here, a dominant control parameter x is chosen to be $x=\sqrt{J_{x}^{2}+{\left(J_{y}-1\right)}^{2}}$. From Equation (9), fidelity entropy S(x) takes the following form

(A207) $$S\left(x\right)=-2\int_{x_{c}}^{x}\ln d(x;y)\;dy+S_{0}.$$

Here, d(x;y) denotes the ground-state fidelity per lattice site in the principal part, and $S_{0}$ is the residual fidelity entropy at the critical point $x_{c}=0$. According to our convention (cf. Section 2), we have $S_{f}\left(x\right)=S\left(x\right)$.

In the principal part ($J_{y}=1-J_{x}$ and $0<J_{x}<1/2$), the ground-state energy density e(x) monotonically increases with x. Then, from Equation (10), fidelity internal energy U(x) takes the following form

(A208) $$U\left(x\right)=-\ln\frac{e(x)}{e(0)}V\left(x\right)+U_{0}.$$

Here, $U_{0}$ is an additive constant, and V(x)>0 satisfies the singular first-order differential equation:

(A209) $$\frac{\partial V(x)}{\partial x}=\alpha \left(x\right)\;V\left(x\right),$$

with

(A210) $$\alpha \left(x\right)=\frac{\partial \ln\left(e(x)/e(0)\right)/\partial x}{\partial S(x)/\partial x-\ln\left(e(x)/e(0)\right)}.$$

Accordingly, fidelity temperature T(x) in the principal part is provided by the following

(A211) $$T\left(x\right)=-\frac{\partial V(x)}{\partial x}.$$

To solve the singular first-order differential equation, Equation (A209), we analyze the scaling behavior of α(x) in the vicinity of the critical point $x_{c}=0$. Fidelity entropy S(x) scales as $S\left(x\right)∼x^{3}$. In addition, our numerical simulation shows that the ground-state energy density e(x), near $x_{c}=0$, scales as ln(e(x)/e(0))∼x. Then, α(x) scales as follows

(A212) $$\alpha \left(x\right)\propto \frac{1}{x}.$$

This scaling analysis is confirmed in our numerical simulations.

Since the integration of α(x) with respect to x is finite, the singular first-order differential equation, Equation (A209), may be solved in a straightforward manner:

(A213) $$V\left(x\right)=V_{0}V_{1}\left(x\right),$$

where $V_{0}$ is a positive constant, and $V_{1}\left(x\right)$ takes the following form

(A214) $$V_{1}\left(x\right)=\exp\left(\int_{0}^{x}\alpha (y)dy\right).$$

The remaining task is to determine $S_{0}$, $U_{0}$, and $V_{0}$. We choose $S_{0}=0$, as follows from the requirement that fidelity entropy is single-valued at the critical point x=0 or, equivalently, $J_{x}=0$ and $J_{y}=1$. We require that, at the $Z_{2}$-symmetric point $x=\sqrt{2}/2$, or equivalently $J_{x}=J_{y}=1/2$, fidelity temperature is zero, thus leading to $T_{0}=T\left(\sqrt{2}/2\right)$. Furthermore, fidelity internal energy at the $Z_{2}$-symmetric point $x=\sqrt{2}/2$, or equivalently $J_{x}=J_{y}=1/2$, takes the maximum value and fidelity internal energy at the critical point x=0, or equivalently $J_{x}=0$ and $J_{y}=1$, is zero: $U_{0}=T_{0}S_{0}$ and $U\left(\sqrt{2}/2\right)-T_{0}S\left(\sqrt{2}/2\right)=U_{m}$, with $U_{m}$ being the maximum value for fidelity internal energy from an exterior point of view. Hence, $V_{0}$ is determined as follows

(A215) $$V_{0}=\frac{U_{m}}{-\ln\left(e\left(\sqrt{2}/2\right)/e\left(0\right)\right)V\left(\sqrt{2}/2\right)+\alpha \left(\sqrt{2}/2\right)V\left(\sqrt{2}/2\right)\left(S\left(\sqrt{2}/2\right)-S_{0}\right)}.$$

After $V_{0}$ and $U_{0}$ are determined, fidelity temperature T(x) and fidelity internal energy U(x) follow from (A208) and (A211). We refer to $T\left(x\right)-T_{0}$ and $U\left(x\right)-T_{0}S\left(x\right)$ as $T_{f}\left(x\right)$ and $U_{f}\left(x\right)$, respectively. That is, $T_{f}\left(x\right)\equiv T\left(x\right)-T_{0}$ and $U_{f}\left(x\right)\equiv U\left(x\right)-T_{0}S\left(x\right)$.

Once fidelity entropy $S_{f}\left(x\right)$, fidelity temperature $T_{f}\left(x\right)$, and fidelity internal energy $U_{f}\left(x\right)$ are determined in the principal part, fidelity entropy $S_{f}\left(J_{x},1-J_{x}\right)$, fidelity temperature $T_{f}\left(J_{x},1-J_{x}\right)$, and fidelity internal energy $U_{f}\left(J_{x},1-J_{x}\right)$ follow. That is, $S_{f}\left(J_{x},1-J_{x}\right)\equiv S_{f}\left(x\right)$, $T_{f}\left(J_{x},1-J_{x}\right)\equiv T_{f}\left(x\right)$, and $U_{f}\left(J_{x},1-J_{x}\right)\equiv U_{f}\left(x\right)$, when we move from *x* to $\left(J_{x},\;1-J_{x}\right)$, meaning that *x* is regarded as a function of $J_{x}$. This is due to the fact that $\left(J_{x},\;1-J_{x}\right)$ and *x* label the same point in the control parameter space. Fidelity mechanical-state functions on the other two lines of critical points follow from dualities. Note that *x*, as a function of $J_{x}$, is monotonic. Hence, it is proper to present the final results as a function of $J_{x}$.

Numerical simulations for fidelity entropy $S_{f}\left(J_{x},1-J_{x}\right)$, fidelity temperature $T_{f}\left(J_{x},1-J_{x}\right)$, and fidelity internal energy $U_{f}\left(J_{x},1-J_{x}\right)$ are shown in Figure 27a–c, respectively.

## Appendix Q. Zamolodchikov RG Flows vs. Real-Space RG Flows

In the main text, we restrict ourselves to formalize fidelity flows mimicking real-space RG flows and do not touch upon Zamolodchikov RG flows. However, as it turns out, it is necessary to make a distinction between real-space RG flows and Zamolodchikov RG flows.

For this purpose, we delve into fidelity flows for the quantum spin-1/2 XY model, discussed in Section 13, which mimic real-space RG flows. For convenience, we reproduce them in the guise of real-space RG flows in Figure A14a. It follows that real-space RG flows proceed from an unstable fixed point to a stable fixed point. Note that there are two lines of critical points: One is the line of the Gaussian critical points (γ=0 with −1<λ<1), with central charge c being 1, and the other is the Ising critical line (λ=1 with γ>0), with central charge c being 1/2. In addition, a critical point with central charge c being 1 is located at infinity, when γ is infinite in value. Therefore, Zamolodchikov RG flows must be drastically different from real-space RG flows. Actually, Zamolodchikov RG flows proceed from an unstable fixed point to another unstable fixed point before they eventually end at a stable fixed point, with a c-function that is non-increasing along any RG trajectory [52]. Note that the c-function becomes central charge c at an unstable fixed point. A sketch of Zamolodchikov RG flows is plotted in Figure A14b for the quantum spin-1/2 XY model. As a consequence, it is necessary to extend the notion of fidelity flows in order to understand Zamolodchikov RG flows in fidelity mechanics.

An observation is that we have to negate our choice of a dominant control parameter x in some principal regimes in order to accommodate fidelity flows mimicking Zamolodchikov RG flows in the context of fidelity mechanics and in the sense that a chosen dominant control parameter x in such a principal regime must be replaced by −x (up to an additive constant). Specifically, for the quantum spin-1/2 XY model, we have to negate our choices for a dominant control parameter x in regime II and regime III (cf. Section 7).

According to our prescription in Section 2, fidelity internal energy U(x), fidelity entropy S(x), and fidelity temperature T(x) follow once a dominant control parameter x is chosen in a given regime. Mathematically, we have ΔU(x)=T(x)ΔS(x). Now, we negate our dominant control parameter choice, $\bar{x}=2x_{c}-x$, as indicated in Figure A15. Then, we anticipate that fidelity temperature T(x) is left intact ($\bar{T}\left(\tilde{x}\right)=T\left(x\right)$), and fidelity internal energy U(x) becomes $\bar{U}\left(\bar{x}\right)={\bar{U}}_{0}-U\left(x\right)$, with ${\bar{U}}_{0}$ being an undetermined (additive) constant. However, fidelity entropy S(x) needs to be replaced by $\bar{S}\left(\bar{x}\right)$: $\bar{S}\left(\bar{x}\right)=-2\int_{{\bar{x}}_{c}}^{\bar{x}}\ln d(\bar{x},\bar{y})d\bar{y}+{\bar{S}}_{b0}$, with ${\bar{S}}_{b0}$ being an additive constant. Hence, we have the following

(A216) $$\bar{\Delta }\left(\bar{U}\left(\bar{x}\right)\right)=\bar{T}\left(\bar{x}\right)\bar{\Delta }\bar{S}\left(\bar{x}\right)+\bar{\Delta }\bar{W}\left(\bar{x}\right).$$

Here, fidelity work $\bar{\Delta }\bar{W}\left(\bar{x}\right)$ takes the following form: $\bar{\Delta }\bar{W}\left(\bar{x}\right)=-\bar{T}\left(\bar{x}\right)\left(\bar{\Delta }\bar{S}\left(\bar{x}\right)-\Delta S\left(x\right)\right)$. Physically, this amounts to stating that $\bar{\Delta }\bar{S}\left(\bar{x}\right)$ bits of information is created in the information storage media associated with a dominant control parameter $\bar{x}$, whereas $\Delta S_{f}\left(x\right)$ bits of information is erased from the information storage media associated with a dominant control parameter x. We emphasize that it is necessary to erase all information recorded in the information storage media associated with a dominant control parameter x. Otherwise, we would be able to remember the future instead of the past, a subtle problem to be dealt with when a dominant control parameter x is negated, resulting in $\bar{x}$.

With the above discussion in mind, we are ready to be back to the quantum spin-1/2 XY model. In regime II, a dominant control parameter x was chosen to be $\lambda -\sqrt{1-\gamma^{2}}$, starting from $\lambda =\sqrt{1-\gamma^{2}}$ on the disorder line and ending at λ=1, for each γ∈(0,1). In regime III, a dominant control parameter x was chosen to be λ, starting from λ=0 and ending at λ=1, for each γ>1. Once the chosen dominant control parameter x is negated in regime II and regime III, we are able to determine fidelity mechanical-state functions in the two regimes. Combined with fidelity mechanical-state functions in regime I, regime IV, and regime V (cf. Section 7), we are able to piece together all regimes by imposing the continuity requirements on the boundaries, thus resulting in fidelity internal energy $U_{f}\left(\lambda ,\gamma \right)$, fidelity entropy $S_{f}\left(\lambda ,\gamma \right)$, and fidelity temperature $T_{f}\left(\lambda ,\gamma \right)$ tailored to Zamolodchikov RG flows in fidelity mechanics. It is during this last step that we transform them back to the original control parameters λ and γ, with a subscript “f” added and the tilde removed from fidelity mechanical-state functions.

We plot fidelity entropy $S_{f}\left(\lambda ,\gamma \right)$, fidelity temperature $T_{f}\left(\lambda ,\gamma \right)$, and fidelity internal energy $U_{f}\left(\lambda ,\gamma \right)$ for the quantum spin-1/2 XY model in Figure A16a–c, respectively. Fidelity entropy $S_{f}\left(\lambda ,\gamma \right)$ exhibits singularities on the two dual lines (γ=1 and λ=0), and on the disordered circle: $\lambda^{2}+\gamma^{2}=1$, in addition to the two lines of critical points, located at γ=0 with −1<λ<1 and λ=1 with γ≠0. One might view such a singularity as a “phase transition” in fidelity mechanics. Note that fidelity entropy $S_{f}\left(\lambda ,\gamma \right)$ reaches its maximum on the dual line (γ=1), when λ is infinite in value. In addition, fidelity temperature $T_{f}\left(\lambda ,\gamma \right)$ diverges at the two lines of critical points (γ=0 with −1<λ<1 and λ=1 with γ≠0), but it is zero on the disordered circle: $\lambda^{2}+\gamma^{2}=1$, and at a characteristic line, representing a factorizing field when λ is infinite in value. Meanwhile, fidelity internal energy $U_{f}\left(\lambda ,\gamma \right)$ takes the same value on the disordered circle: $\lambda^{2}+\gamma^{2}=1$, and it reaches its maximum when λ is infinite in value.

Although we restrict our discussion to the quantum spin-1/2 XY model, it is applicable to any quantum many-body systems undergoing QPTs regardless of dimensionality. As such, it sheds new light on Zamolodhikov’s c-theorem [52,53] and Cardy’s a-theorem [55,56].
